# New Method for Identifying Fungal Kingdom Enzyme Hotspots from Genome Sequences

**DOI:** 10.3390/jof7030207

**Published:** 2021-03-11

**Authors:** Lene Lange, Kristian Barrett, Anne S. Meyer

**Affiliations:** 1 BioEconomy, Research & Advisory, Copenhagen, 2500 Valby, Denmark; lene.lange2@gmail.com; 2 Section for Protein Chemistry and Enzyme Technology, Department of Biotechnology and Biomedicine, Building 221, Technical University of Denmark, DK-2800 Kgs. Lyngby, Denmark; kbaka@dtu.dk

**Keywords:** taxonomic enzyme hotspots, eco-physiological enzyme hotspot, peptide-based functional annotation, CUPP, enzyme function specificity diversity, enzyme profiles

## Abstract

Fungal genome sequencing data represent an enormous pool of information for enzyme discovery. Here, we report a new approach to identify and quantitatively compare biomass-degrading capacity and diversity of fungal genomes via integrated function-family annotation of carbohydrate-active enzymes (CAZymes) encoded by the genomes. Based on analyses of 1932 fungal genomes the most potent hotspots of fungal biomass processing CAZymes are identified and ranked according to substrate degradation capacity. The analysis is achieved by a new bioinformatics approach, Conserved Unique Peptide Patterns (CUPP), providing for CAZyme-family annotation and robust prediction of molecular function followed by conversion of the CUPP output to lists of integrated “Function;Family” (e.g., EC 3.2.1.4;GH5) enzyme observations. An EC-function found in several protein families counts as different observations. Summing up such observations allows for ranking of all analyzed genome sequenced fungal species according to richness in CAZyme function diversity and degrading capacity. Identifying fungal CAZyme hotspots provides for identification of fungal species richest in cellulolytic, xylanolytic, pectinolytic, and lignin modifying enzymes. The fungal enzyme hotspots are found in fungi having very different lifestyle, ecology, physiology and substrate/host affinity. Surprisingly, most CAZyme hotspots are found in enzymatically understudied and unexploited species. In contrast, the most well-known fungal enzyme producers, from where many industrially exploited enzymes are derived, are ranking unexpectedly low. The results contribute to elucidating the evolution of fungal substrate-digestive CAZyme profiles, ecophysiology, and habitat adaptations, and expand the knowledge base for novel and improved biomass resource utilization.

## 1. Introduction

Fungi play an important role in the recirculation of organic resources in nature, and their ability to decompose, modify, and essentially thrive on natural biomass is enabled by an arsenal of carbohydrate-active enzymes and accessory enzymes, CAZymes (www.CAZy.org (accessed on 1 February 2021)), of which many are brought to use by an enzyme secretion mechanism, specifically well developed in filamentous fungi [1]. To unlock the full potential of plant biomass and agro-industrial feed stocks in the development of the new circular, bio-based economy, we need to identify new enzymes and microbes for improved bioprocessing of a wide spectrum of biomasses resources, processing side-streams and wastes [2,3]. Fungi and specifically fungal genomes represent huge reservoirs of biotechnologically useful enzymes for such processes [3,4,5]. The current portfolio of enzymes used in industrial biotechnology and in biomass conversion is derived from a minute part of the Fungal (and Bacterial) Kingdoms [5]. Clearly, the provision of new insight into the biomass degrading potential of fungi and comprehension of their enzyme profiles is imperative in the pursuit of new enzymes, improved resource efficiency, and biomass utilization [4,5]. The focus of the present study is to explore the exponentially growing space of un-annotated fungal genome data to identify promising fungal species and eco-physiological specializations with particularly rich and interesting CAZyme gene pools. We use a new bioinformatics-based approach to mine the immense amounts of available fungal genomic data to identify fungi whose genomes harbor genes that encode particularly high numbers of biomass processing enzymes associated with conversion of different groups of the key biomass substrates, cellulose, xylan, pectin, and lignin. Our aim is to contribute a new approach for efficiently using genome data to identify fungal species that feature exceptionally interesting CAZyme gene pools. This approach provides a novel basis for selecting new enzyme systems to develop more efficient biomass conversion and processing strategies for upgrading of a wide spectrum of biomass resources, side-streams and wastes.

The genomic era has focused on revealing genomics of individual organisms, species or strains seen in isolation. Now, the time is ripe to tackle the next step, focusing on revealing evolutionary patterns of fungal interaction with their substrates. The initial step in this endeavor is to have methods for robust and fast functional annotation of genomes, exerting both high precision and sensitivity. The CAZymes are particularly diverse and highly specific due to the wealth of structural and chemical diversity of their substrates, and the proteins in focus for interaction and substrate conversion are the digestive carbohydrate degrading enzymes. Annotation to family and subfamily of such CAZymes is highly efficient and robust [6,7]. New approaches to functional annotation have been published recently, namely eCAMI [8], dbCAN2 [9], SACCHARIS [10] and CUPP [11,12] that all represent alternatives to the more general, widely used hmm-based functional annotation provided by Pfam and HMMer3 [13]. The breakthroughs in the robust prediction of enzyme function directly from genome sequence, facilitate the use of the ample amount of available genomic data for improved biological understanding of the evolution of the substrate interaction of fungi. Digital prediction methods furthermore provide a basis for intelligent and targeted enzyme discovery across all parts of the Fungal Kingdom. CUPP, for example, facilitates a comparison between the activity profiles of polysaccharide degrading enzymes across all taxonomic ranks, from species to phyla [14]. This is done by using peptide-based functional annotation to construct a new type of integrated “Function;Family” observation, first described in [14], and used for sub-genus grouping of species based on enzyme profile relatedness.

The current study uses the concept of “Function;Family” observations to identify fungal CAZyme hotspots by making “Function;Family” observation-based calculations, suitable for cross taxonomy comparisons, enabling ranking of species according to degrading capacity and diversity in enzymatic substrate specificity. This ranking is then transformed into an evolutionary, biological and ecological context by integrating the bio-informatics-based prediction of function with the mycological knowledge of phenotype, lifestyle, habitat, and ecophysiology. The hypothesis guiding this study is that “Function; Family” observations reflect fitness-relevant, evolutionarily important characters for fungal speciation and competitiveness. The core tenet is that such analysis and ranking provide valuable guidance for innovative and targeted enzyme discovery as well as significant new insight into evolution of fungal digestive CAZymes across eco-physiological specializations and lifeforms. Thus, this study contributes to shed light on the evolution of fungal digestive enzyme portfolio, in a broad spectrum of fungal lifestyles, ecological patterns, physiological specializations and substrate affinities among the genome sequenced fungi now available. The results include several surprising findings of where the richest, most optimized enzyme portfolio are found in nature; and also shows that the fungal species, from where most of the industrially used enzymes are derived, display a rather low ranking among the total of 1932 fungal genomes included in the analysis.

## 2. Materials and Methods

### 2.1. Genomes, Selection, and Filtering

6775 fungal genomic assemblies available in the NCBI GenBank were downloaded, and statistics and filtering were assessed in the following way: For each fungal species, the assembly having the greatest sequencing coverage times the Contig N-50 value [15] was selected as representative of the species. This selection resulted in inclusion of 1932 non-redundant, genomic assemblies each representing one fungal species (or strain), according to the NCBI taxid (see Tables S1 and S2 for list of genomes analyzed, including accession numbers and acknowledgement of source of genome sequencing data). We are aware that the genomes in the NCBI GenBank have been deposited by different research groups and may have been sequenced by different techniques. For certain genera where strains have received a NCBI taxid, multiple representatives of the same species may have been included. Some genomes may even be incomplete or may suffer from incomplete annotation. The data presented therefore carry the uncertainty that certain CAZymes may have been underestimated in the annotation of those genomes. The described filtering of genomes made, including selection according to high N-50 is diminishing this problem, but cannot eliminate it entirely. The study includes genomes of all fungal phyla, but members of the fungal yeast class, Saccharomycetes, are not included.

### 2.2. Prediction of Proteins and Assembly

In order to make the comparison of the assemblies more systematic and less biased we predicted all the proteins on the same basis using Augustus software [16,17]. Genomic proteins were ab initio predicted using Augustus 2.5.5 versus a selected model organism based on the taxonomical phylum origin of the genome as follows: Assemblies of Ascomycota were predicted using the model organism *Aspergillus oryzae*, whereas Basidiomycota members were predicted using the model organism *Phanerochaete chrysosporium*, and species belonging to any other phylum were predicted using *Rhizopus oryzae* as model organism. The resulting protein lists were annotated using CUPP.INFO (v2020) [12] for annotation of CAZy family, EC number and CAZy family subgroups (and CUPP groups) [11]. In brief, for each of the groups, the proteins and their taxonomical origin can be browsed using CUPP.INFO (https://www.cupp.info/browse (accessed on 1 February 2021)), e.g., the 2nd group of GH7 can be found (https://www.cupp.info/cupp/GH7:2.1 (accessed on 1 February 2021)) and the group members examined (limited to genomes in the CAZy database). Based on the EC numbers and, to some extent, the general substrate specificity of the CAZy families, the likely target plant cell wall polysaccharide substrate of the individual enzymes was determined, given as “Function;Family” observations as explained in [14]. The polysaccharide substrates and lignin associated with each of the “Function;Family” observations are listed in Table S3.

The annotation by CUPP provides functional annotation in addition to general CAZy family annotation. The use of CUPP does not reduce the sensitivity for CAZy family annotation relative to the sensitivity of the HMMer-based dbCAN. The original validation of the CUPP method thus showed that the F-score for CUPP annotation was 0.97–0.98 whereas the F-score of dbCAN was 0.94–0.97 [11,12].

**Figure 1:**
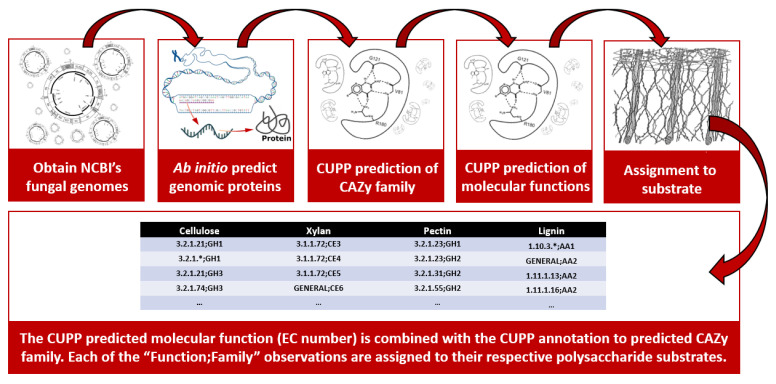
Flow diagram describing the steps from downloading the full fungal genomes from NCBI and prediction of genomic proteins using AUGUSTUS. The carbohydrate-active enzymes were assigned a CAZy family and molecular function prediction via CUPP (see text). The predicted Function;Family observations were then assigned to their respective target substrates. The * indication added as fourht number in some EC numbers (molecular function numbers) indicate that no full EC number is available in the CAZy database. A similar molecular function (same EC number) from two different CAZy families are considered as two different observations by combining the EC number and the CAZy family into a combined string.

Basically, the CUPP method first involves obtainment of all carbohydrate-active enzymes from CAZy.org (accessed on 1 February 2021); these are then organized into groups of proteins each with unique patterns of peptides. The CUPP method relies on that the signatures of peptides differ for each group, which in turn allows for rapid annotation of genomic proteins. In case one or more members of a group may have a known molecular function, this allows for extrapolation of information to the newly annotated proteins. The predicted molecular function is given as EC number. In this work, the molecular function is used in the contest of the CAZy family from which it originates (see Figure 1).

Flow diagram of genomic based “Function;Family” prediction of CAZymes by CUPP.

### 2.3. Functional Annotation and Definition of “Function;Family” Observation

The method for identifying the fungal genomes, richest in substrate specificity diversity or most potent with regard to degrading capacity includes three steps: Step 1, Peptide-based functional CAZyme annotation of 1932 fungal genomes, using the CUPP method [11,12]; Step 2, converting such separated family and EC-function annotations (EC-function is understood here as functional classification or prediction, according to EC number) into a list of integrated “Function;Family” observations, obtained by combining the CUPP predicted EC-function of the enzyme with the type of CAZyme protein family it belongs to. Step 3, the method is completed by accumulating the “Function;Family” observations into one score for each species of total number of observations for each specific substrate. The result of this 3-step calculation is a ranking, reflecting the enzyme substrate specificity diversity as well as the accumulated enzyme biomass degradation capacity of the individual species. Please note, the LPMOs are not included in this survey, as current state of knowledge does not allow for prediction of function within this group of CAZymes.

### 2.4. “Function;Family” Observation-Based Ranking

For the hotspot ranking results, the term “Unique observation” means that each “Function;Family” observation only counts once. This means that multiple “Function;Family” observations found in the same fungus are not counted more than once. The tables listing these hotspot rankings indicate “enzyme function specificity diversity”. In contrast, the tables summarizing all occurrences of each observation, meaning that all the CAZymes annotated to the individual substrates were summarized regardless of the particular “Function;Family”, provide “enzyme degrading capacity”. We are aware that certain fungal genome assemblies are deposited in the NCBI GenBank with diploid allele contigs in addition to the haploid representation; this problem can result in too high number estimates of encoded enzymes. The inclusion of both the total and the unique observation counts and rankings partly attune to this problem that that there is currently no direct way to adjust for. In case a single EC number is associated with two plant cell wall substrates, the enzymes count as half for both substrates.

### 2.5. Substrate Association Analysis Included in “Function;Family” Observation-Based Ranking

The scores of “Function;Family” observations on each CAZyme-targeted substrate is supplemented by analysis of ranking according to relevant combinations of the substrate groups, i.e., cellulose + xylan, cellulose + xylan + lignin; and lignin + pectin, respectively.

### 2.6. Definition of Redundancy Multiplication score

By calculating the ratio between total number of “Function;Family” observations as compared to the number of unique observations a measure is achieved, which quantify the boosting of enzyme degrading capacity due to an organism having several enzymes of the same type of “Function;Family” observation. It is called Redundancy Multiplication Score.

## 3. Results

### 3.1. Total CAZyme Cell Wall Polymer Degrading Capacity-All Observations Included

The results reported in Table 1 include lists of the ten top-ranked species, based on all accumulated (“redundant”) CAZyme “Function;Family” observations for four plant cell wall substrates, listed separately for Cellulose, Xylan, Pectin and Lignin, and summed up as “Total”.

These accumulated “Function;Family” observations thus identify the fungal species, which encode for particularly rich sets of carbohydrate processing enzymes with affinity for the major plant cell wall components. (CAZymes, www.cazy.org (accessed on 1 February 2021), except glycosyl transferases). The ranking of all 1932 fungal species/strains analyzed is given in Tables S1 and S2. Table S1 lists number of observations, including redundancy, reflecting the overall CAZyme degrading capacity of the species. It includes both specificity diversity and number of copies of each type of “Function;Family” observation, where several copies of same “Function;Family” observation are found in the fungal genome. Table S2 includes only the number of unique “Function;Family” observations, reflecting the enzyme function specificity diversity of the fungal species analyzed. Taxonomy and phylogeny follow the recently reported advances in fungal systematics [18,19,20].

*Total CAZyme cell wall polymer degrading capacity*. As appears from the ranking according to the column “Total” in Table 1, the strongest cell wall degrading capacities are found in a surprisingly diverse spectrum of species with regard to taxonomy, lifestyle, substrate affinities and ecophysiology. This finding substantiates that the evolutionary development towards a rich cell wall polymer degrading capacity has taken place in many habitats, among many fungal life forms, many types of substrates and ecophysiological specialisations and in several Fungal Kingdom phyla: the two top hotspot species are early lineage, zoosporic anaerobic rumen fungi, *Pecoramyces ruminatium* and *Neocallimastix californiae* (both Neocallimastigomycetes, Chytridiomycota).

The exceptionally high “Function;Family” observation scoring of rumen fungi (*P. ruminatium* and *N. californiae* (Neocallimastigomycetes)) reflects that the genomes of these organisms encode for a very high number of cellulose and xylan degrading enzymes in accord with the types of substrates the fungi encounter in the cow’s rumen [21]. In addition, the high scores may also reflect that these highly specialized early diverging, anaerobic fungi have a unique genome organization (including numerous duplications) and a similarly unique enzyme cellulosome structure [22,23].

The third on the list of fungal Top10 cell wall polymer-degrading capacity species (see Table 1, Column “Total”) is the Basidiomycete, *Mycena citricolor* (Agaricomycetes). The astonishingly potent CAZyme portfolio of *M. citricolor*, dominated by a very high number of pectin degrading and lignin modifying enzyme activities is unique and surprising for a Basidiomycete. From Table S1 it appears that only three basidiomycetes are found among the top ranking 100 fungal species, placed as 3, 35 and 71, respectively.

**Figure 2:**
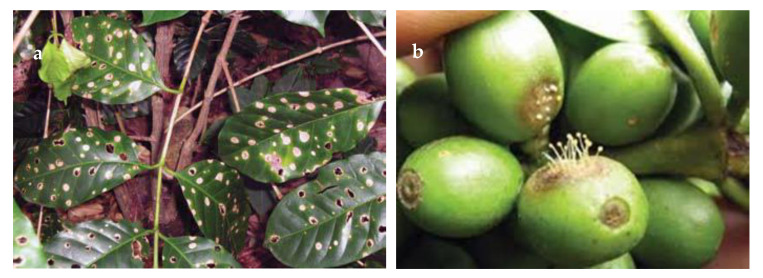
Attack of American Coffee Spot, by *Mycena citricolor.* (**a**) on coffee leaves, leading to total degradation of attacked areas, forming conspicuous holes in the leaves. (**b**) Minute fruiting bodies (caps) of *M. citricolor* invading coffee berries. The berry is strongly affected by the fungal infection, causing brown necrotic tissue. The entire infected area is sunken in, flattening the shape of the infected coffee fruit. In short, the figure hints the effect of a plant pathogen with a powerful, pectinolytic as well as ligninolytic secretome of biomass degrading enzymes. Photos: Andrew Dominick. University of Wisconsin-La Crosse and Direcction General de Sanidad Vegetal, Sagarpa (Secretaria de Agricultura Ganaderia Desarrollo Rural Pesca y Alimentacion), Mexico.

The rich enzyme capacity profile found in *M. citricolor* mirrors that it is an aggressive plant pathogen (causing American Coffee Spot disease), being able to enzymatically invade all parts of the coffee plant, leaves, stems and fruits (see Figure 2). Furthermore, *M. citricolor* also grows as a saprotroph on dead coffee plant biomass. Its enzymatic degrading capacity is evident in the field as the coffee leaves infected are so strongly affected by the fungal attack, that conspicuous holes in the leaves are formed (see Figure 2a). Further, the fruiting bodies of this minute cap fungus are visible by the naked eye and occur close to brown spots on leaves, stems and fruits (Figure 2b). Interestingly, *M. citricolor* also has a strong metabolite profile, e.g., its fruiting bodies are visible at night, as it shows bioluminescence [24]. One other *Mycena* species is included in the rankings achieved in the current study (Table 1), *M. chlorophos*, listed as 696 on the “Total”-ranked list of species, including redundant enzyme “Function;Family” observations (Table S2). The specific comparison of the predicted enzyme observation profile of *M. citricolor/M. chlorophos* is consistent with the high pectin degrading enzyme capacity of *M. citricolor*: Cellulose 91/40; Xylan 50/27; Pectin 204/35; Lignin 149/64.

Number four to ten of the richest biomass degrading enzyme capacity species are all belonging to Ascomycota (Table 1). Among those, the species with the strongest biomass degrading capacity is *Verticillium longisporum* (Hypocreales, Sordariomycetes). Similar to most *Verticillium* species, *V. longisporum* primarily lives in the soil. However, *V. longisporum*, is also a plant pathogen of dicot plants, most commonly infecting canola, here causing *Verticillium* Wilt. It can attack other brassica plant species as well as woody ornamentals. The fungus invades all parts of the plant also spreading efficiently through the vascular system. Besides its high CAZyme degrading capacity, with particularly high numbers of pectin and cellulose degrading enzyme “Function;Family” observations encoded by its genome, it also secretes mycotoxins, and exerts chemotactic response in the soil, growing towards host root systems [25]. The fifth species on biomass degrading enzyme capacity is *Coniochaeta* sp. 2T2.1, (Ascomycota, Sordariomycetes, Coniochaetales). The genus *Coniochaeta* includes species of pleomorphic, sapro-and bio-trophic yeasts and are primarily described as tree pathogens [26]. In accordance with its diverse ecophysiological specialization and pleomorphic life form, Table 1 shows that this fungus encodes a high number of CAZyme “Function;Family” observations for each of the four types of plant cell wall substrates, cellulose, pectin, xylan, and lignin. The CUPP analyzed genome sequenced strain *Coniochaeta* sp. 2T2.1 was isolated from a wheat straw-degrading microbial consortium. The strain *Coniochaeta* 2T2.1 is also described to be a key Eukaryote member of a soil-derived microbial consortium along with bacteria [27].

The sixth species on the “Total” list in Table 1 is *Paramyrothecium roridum*, Hypocreales, Sordariomycetes, Ascomycota (synonym *Myrothecium roridum*). It is a soil inhabiting, facultative plant parasite with a large host range and worldwide distribution. In agriculture, it is recognized as an important pathogen of vegetable (dicot) crops, which agrees well with its high capacity for pectin and cellulose degradation, according to its encoded CAZymes attacking these substrates [28]. It is a strong producer of bioactive metabolites such as Mycotoxin B. The seventh species on the Total list, Table 1 is the endophytic filamentous *Cadophora* sp. DSE1049, (Helotiales, Leotiomycetes, Ascomycota), which has an enzyme profile for the four target plant substrates almost similar to that of *P. roridum*, but just slightly lower observation counts for pectin degrading enzymes and slightly higher counts of xylan and lignin modifying enzyme observations (Table 1). This isolate may represent a new species in the genus [29]. *Cadophora* is associated to the ubiquitous fungal grouping of “dark septate endophyte” community. It is common on semi-arid grasslands, observed to colonize both grass and non-grass hosts. The functional role of the “dark septate endophyte” (DSE) fungi in the ecosystem is still elusive. Characterization of its digestive enzyme profile in the present study can contribute to shed light on DSE interaction with their host plants [30].

Number eight to ten in the “Total” column in Table 1 are three species of the *Diaporthe* (Sordariales). *Diaporthe* is a genus of endophytic, saprotroph and pathogenic fungi. *Diaporthe* spp. produce the mycotoxin Phomopsins, causing liver damage. In grapevines *D. ampelina* causes two different diseases, *Phomopsis* cane and leaf spot and *Phomopsis* dieback (notably, *Phomopsis* is an earlier genus name of *D. ampelina). D. ampelina* directly attacks all green tissues of the vine, causing necrotic lesions on the leaves, green stems, and fruit [31]. The habitat is clearly reflected in the high numbers of cellulose and lignin degrading enzymes of *Diaporthe* sp., but it is also important to note that all three “top 10” *Diaporthe* spp. have surprisingly good pectin degrading capacities according to the number of pectin degraded CAZyme observations identified (Table 1). Next on the Top 10 list is another *Diaporthe* species, *D. longicolla* (syn. *Phomopsis longicolla*). It is a seed-borne fungus causing “Phomopsis seed decay” in soybean, *Glycine max* [32].

### 3.2. Redundant Observations Included: Fungal Hotspots of Degradation Capacity for Specific Substrates

*Cellulose*. A striking similarity is found between the Cellulose ranked list and the Total degradation capacity list above. As it appears from Table 1, the ranking of cellulose active enzyme observations has a high impact on the total ranking: eight of the top ten fungal species, scoring highest on the “Cellulose” active enzyme observations are the same as for “Total” cell wall degrading. Notably, *M. citricola* and *Cadophora* sp. DSE1049 are not on the cellulose ranked list of species richest in degradation capacity, as they primarily have high pectin degrading capacity (Table 1). Further, the Cellulose-ranked list includes a third rumen fungus, *Piromyces* (*P.* sp. E2) and one more species of *Diaporthe* (*D. capsica*); all *Diaporthe* species analyzed have similarly rich CAZyme profiles.

*Xylan*. The four highest scoring species, with the richest xylan degradation capacity among the 1932 genome sequenced fungal species are rumen fungi (Chytridiomycota, see Table 2, Xylan, # 1,2,3 and 8). Despite this, the xylan-ranked list, in contrast to the cellulose-ranked list, holds several surprises in including species not on the Total list and not earlier described as potent, high enzyme capacity degraders: The saprotrophic wood degrader *Exidia glandulosa*, (Agaricomycetes, Basidiomycota); the “rock fungus”, an extremophile ascomycetous black yeast, *Rachicladosporium antarcticum*; and an understudied coniferous needle degrader, *Chalara longipes* (Leotiomycetes, Ascomycota). Furthermore, *Coniochaeta* sp., *Cadophora* sp. and *V. longispora*, which all are placed among the top 10 on the Total-list (Table 1) are also top-scoring on xylan.

*Pectin*. The absolute highest scoring (in all 204 “Function;Family” pectin-associated observations) is *M. citricolor*. A broad selection of CAZymes, degrading also the pectin rich coffee fruits is part of the pathogenicity profile of *M. citricolor*. However, most unique for the Pectin-ranked listing in Table 1 is that out of the top ten species, *Colletotrichum* sp. are ranked four to eight. Species of *Colletotrichum* are well-known plant pathogens, invading all part of their host plants, found to grow also as endophytes. Unique for the Pectin-ranked listing is further an *Aspergillus species*, *A. latus*, *V. longisporum*, *P. roridum* and *Cadophora* sp. DSE1049 on the Pectin-ranked list are all listed also on the Total degrading capacity list (Table 1). This indicates that these latter three species have an overall rich CAZyme cell wall degrading enzyme portfolio but also being highly specialized in degrading pectin. As expected, yet noteworthy, no rumen fungi are represented among the pectin-ranked top ten species; highest placed rumen fungus is *N. californiae*, placed as #30 among pectin-ranked species (see Table S2).

*Lignin*. The list of fungal species with the most diverse and strongest lignin degrading capacity is different from all of the above list: It is dominated by Agaricomycetes (Basidiomycota). No chytridiomycetous rumen fungi and only three ascomycetous species are on the top 10 list ranked according to number of lignin enzyme observations. Among the Basidiomycota, as also for pectin degradation, *M. citricolor* again stands out as number one in lignin degrading capacity (having in all 149 “Function; Family” lignin-associated observations). A similar profile as *M. citricolor*, being specialized in both pectin and lignin degradation, is also found for the three Ascomycetes ranked highest among lignin degrading fungi, *Coniochaeta* sp., *D. ampelina* and *V. longisporum* (Table 1 Lignin, ranked as number 6, 8 and 10). The habitat and ecophysiological specialization of the five lignin-degrading genome-sequenced Basidiomycetous species, placed as numbers 1–5, 7 and 9 are highly diverse: *M. citricolor; Exidia glandulosa* (wood degrading typically growing on dead attached branches of oak); *Hymenopellis chiangmaiae* and *H. radicata* (soil inhabiting, deep rooted; known as edible Black Termite Mushroom in China) [33]; *Ganoderma boninense* and *G.* sp. BRIUMSc (the *Ganoderma* genus being well known for around the world causing decay in a wide range of tree species, including oil palm); and *Neonothopanus nambi* (a poisonous, bioluminescent mushroom in the family Marasmiaceae) [34,35].

### 3.3. Top 10 Fungal Species, Richest in Function Specificity Diversity-Unique Observations Only

*Total*. The evolutionary development in enzyme cell wall substrate specificity has one outstanding taxonomic and lifestyle hotspot: Nine out of the ten topmost enzyme specificity rich fungal species/strains belong to the genus *Colletotrichum*, (Sordariomycetes, Ascomycota) (see Table 2 and Table S2).

*Colletotrichum* species can invade all part of their host plants and can live both as symbionts, as endophytes and as plant pathogens; some species even have a mutualistic relationship with their hosts. Notably, enzymes from *Colletotricum* are only sparsely studied experimentally, as they are known as producers of highly biologically active mycotoxins (also against humans). Notably, #7 on the “Total”-ranked, unique/non-redundant list is another well-known mycotoxin producer *P. roridum*, from the same taxonomic group as *Colletotrichum*. The number of unique, non-redundant CAZyme “Function;Family” observations in *Colletotrichum* sp. and *P. roridum* are as high as 109–112. 

### 3.4. Unique Observations, Listing Top Ranking Species on Specific Substrates

*Cellulose.* The ranking according to number of *unique* cellulolytic “Function;Family” observations (not including redundant observations) is astonishing in that all the top-scoring species are understudied (and totally unexploited) with regard to their enzyme portfolio. The ranking is also remarkably different from the top ten species of the “Total” list above as only one species of *Colletotrichum* is included on the Cellulose-ranked list (Table 2). Specifically pinpointing number 1–10 separately is not possible as it includes six top-scoring species, which all have 18 unique cellulose active “Function;Family” observations. Followed by a long list of species having 17 unique observations. The group of six species, all having 18 observations is dominated by Sordariomycetes (four species, see Table 2). The lifestyles and substrate and host affinity among these six species are very diverse: *Gliomastix tumulicola* (*Gliomastix* spp. are saprotrophs; *G. tumulicola* part of the *Gliomastix/Bionectria* clade of *Acremonium*-complex [36]); *P. roridum* (see above); *Aaosphaeria arxii* (*A. arxii* was originally isolated from corn (*Zea maydis*) in Columbia and it has since been found on various plants) [37]; *Clavariopsis aquatica*, (a marine fungus, belonging to a less studied part of the Pleosporales (Dothideomycetes [38]); *Hymenoschyphus herbarum*, (a saprotroph on twigs and branches, belonging to Helotiales, Leotiomycetes)*;* and *Memnoniella echinata*, an indoor mold, very similar to *Stachybotrys chartarum*; was previously named *S. echinata*; it has a world-wide distribution, mainly isolated from soil and from cellulose containing materials such as paper, wallpaper, textiles and dead plant material). *M. echinata* produces toxic metabolites similar to those of *S. chartarum* [39]. As can be seen in Table S2 there is a high number of species having 17 unique “Function;Family” observations. This long list is dominated by Ascomycetes but also including three species of the Basidiomycetous *Auricularia* spp. The Ascomycete diversity is broader when looking on top 70, including, e.g., *Morchella eximia* (Pezizocomycetes), (a globally occurring fire-associated species of Morels) [40], and one species of *Aspergillus* (*A. versicolor*), belonging to Eurotiomycetes.

*Xylan*. *Cadophora* (Leotiomycetes, Ascomycota) has the richest xylan “Function;Family” observation score (26). Six species have xylan function diversity score of 25: The previously introduced species *C. tropicale* and *C. aenigma* and *P. roridum*, and also, *Clonostachys rosea*, (a mycoparasite, ubiquitously found and isolated from many types of habitats, most often from soil). Mycoparasitism of *C. rosea* has been exploited for biological control of numerous fungal plant pathogens, insects and nematodes. This biological activity is enabled by multiple mechanisms: a secretome rich in both cell wall degrading enzymes and biologically active secondary metabolites, and induction of plant defense. *C. rosea* has also been described to have enzyme functions of relevance for biodegradation of plastic waste [41]. *Xylaria striata*, having a secretome composed of both biomass degrading enzymes and biologically active metabolites, shown to promote plants biomass growth, is also highly ranked for xylan associated CAZymes (Table 2). Chinese culture, CCTCC NO: M2012101, *X. striata*, strain RK1-1 has been associated with anti-aging activity in nematodes [42]. Notably, a high number of other Ascomycetous species have a xylan “Function;Family” diversity score of at least 24 (Table S2).

*Pectin*. Species of *Colletotrichum* fill all the ten top-ranking positions with regard to their pectin-active enzyme “Function;Family” observation portfolio (range 51–53). Notably, among the species of *Colletotrichum* here ranked highest with respect to pectin specificity diversity is *C. gloeosporioides*, a tea pathogen with a highly bio-active metabolite profile. Since most of the fungi having high numbers of CAZyme observations are indeed plant pathogens, it is noted that strong cell wall degrading capacity often is co-occurring with a rich mycotoxin profile, indicating that the evolutionary development of a strong CAZymes battery to support efficient growth is often combined with a biologically active secreted metabolome.

*Lignin*. In all, sixteen species have the highest scoring of unique “Function;Family” lignin-active observations (19) (Table 2 and Table S2). This list holds 13 Ascomycetes (belonging to Sordario-, Leotio- and Dothideomycetes); and three Basidiomycete Agaricomycetess, two species of *Auricularia* and one *Hymenopellis* species (Table S2) (for lifestyle and substrate affinity, see text above).

### 3.5. Substrate Association Analyses, Unique Observations

Substrate association analyses were made for unique observation data, including Cellulose + Xylan, Cellulose + Xylan + Lignin, and Lignin + Pectin. Interestingly, among the top scoring species on Cellulose + Xylan + Lignin is the wood inhabiting *Xylaria striata* (data calculated from Table S2). Notably, with the important exception of *Mycena citricolor*, among all Top50 scoring species in the Pectin + Lignin substrate association analysis are Ascomycota. Among these, Sordariomycetous species are dominating, specially, high specificity diversity is found (beyond *Colletotrichum* sp) in *Verticillium longisporum*, *Paramyrothecium*, *Pestalotiopsis*, *Fusarium oxysporum*, and *Diaporthe*; but also, very rich diversity in pectin and lignin function specificity diversity are found in ascomycetous species outside *Sordariomycetes*, e.g., *Staganospora* (Dothideomycetes), *Morchella esculenta* (Pezizomycetes, most famous edible morel).

### 3.6. Substrate Association Analyses, Including Redundant Observations

For redundant observations, similar substrate association analyses were made (data calculated from Table S1). Interestingly, linkage analysis revealed the high enzyme degrading capacity of several new species, as, e.g., by linking Cellulose + Xylan: *Aspergillus* and *Byssochlamus* (Eurotiomycetes); *Zopfia* (Dothideomycetes), a root inhabiting plant pathogen; *Hortaea*, a black yeast; and *Coprinellus*, a soil inhabiting saprotrophic Basidiomycete. Of special interest is also the list of species, top scoring on substrate association of “Function;Family” observation scoring on pectin plus lignin (see Table 3).

### 3.7. Analysis of Variation of Biomass Degrading Capacity among Species of Same Genus

The enzyme biomass degrading profile among the high number of genome sequenced species of *Aspergillus, Penicillium* and *Fusarium* is highly varying. All three genera include species with strong and medium strong enzyme profiles, as well as species with only meager biomass degrading capacity. In contrast to this the genus *Diaporthe* and *Colletotricum* appear to be more uniform in total degrading capacity across genome sequenced species included in the study. In numbers: The species of *Aspergillus* included (in all 103) have a diversified ranking from top *A. latus* (5) to bottom ranked *A. cejpii* (1109). The 48 *Penicillium* species included have ranking positions varying between *P.* sp. 61, *P. janthinellum 203*, and the lowest ranking in biomass degrading capacity *P. decumbens* 1258. The genome sequences included from *Fusarium* species (in all 180 species) have a ranking, ranging from *F.* sp. (NRRL 22101) 54 to bottom *F. ventricosum* 1201. Albeit fewer species sequenced the diversity of enzyme profiles of the genus *Colletotrichum* and *Diaporthe* appear to be much more potent and much narrower: *Colletotricum*, 34 sequenced species included, *C. cameliae* 14; and bottom ranked species *C. falcatum* at 324. *Diaporthe*, 7 genome sequenced species, ranking as high as 8–12, 42 and 94, respectively, in Total degrading capacity. 

### 3.8. Redundancy Multiplication Score: Ratio of Redundant vs. Unique “Function;Family” Observations

The level of “Function;Family” observations including redundant observations as compared to the level of unique non-redundant observations is given as ratios in Table 4. This table includes calculation for the ten species of each of the four types of enzyme portfolios, cellulolytic, xylanolytic, pectinolytic and ligninolytic; plus, a “Total” cell wall polymer degrading capacity, summed up across the four types of enzymes. For each of such sets of 10 species, the number of non-redundant “Function;Family” observations is also listed. To the right in Table 4 is the calculated redundancy multiplication; describing the boosting in degrading capacity, possibly caused by higher redundancy in number of “Function;Family” observations.

For three groupings, “Total”, “Cellulose” and “Xylan” it appears that the two rumen fungi, *P. ruminatium* and *N. californiae* (both Neocallimastigomycetes, Chytridiomycota) have the highest redundancy multiplication score (marked by *); similary, *Piromyces* E2 is excelling in Redundancy Multiplication Score on Xylan; to be noted, this type of specialized rumen fungi have their cell wall degrading enzymes placed in a specialized cellulosome structure and a high duplication genome structure. The highest Redundancy Multiplication Score after the three rumen fungi are found for the Basidiomycete *M. citricolor*, for both pectin and lignin, as well as for total; closely followed by *V. longisporum, Coniochaeta* (Cellulose), *Diaporthe ampelina, Piromyces E2* and *P. finis* (Xylan), and *Exidia glandulosa* (Lignin). The lowest Redundancy Multiplication Score is found among Ascomycetous hits of pectin-active as well as cellulose-active enzyme observations.

### 3.9. Taxonomic Distribution of Fungal Phyla According to Enzyme Biomass Degrading Capacity

**Figure 3:**
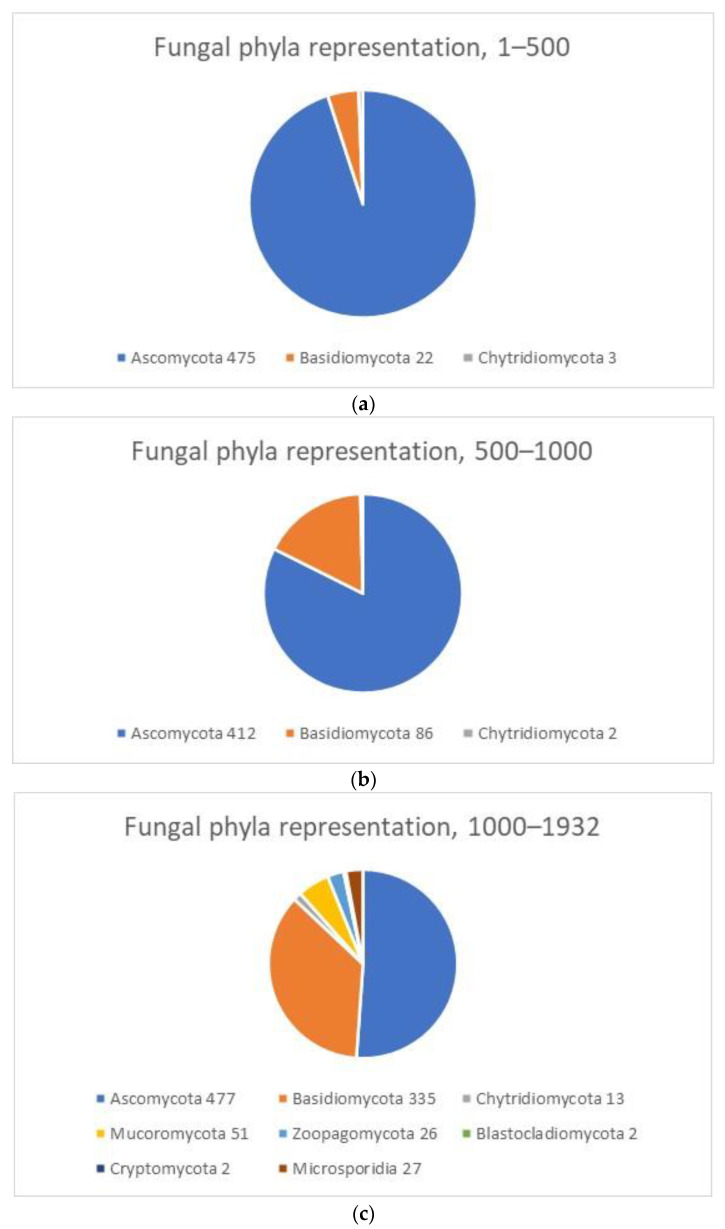
Pie chart representation of representation (in number of species) of fungal phyla in the upper most part of the list of Total biomass-degrading capacity: (**a**): 1–500, (**b**): 500–1000 and (**c**): 1000–1932. Ascomycota species dominates from 1–1000; Basidiomycota grow from 22 to 86 to 335 from upper part to lower part of the ranking list. All 8 fungal phyla are represented in the second half of the ranked listing of in all 1932 species, although some are in such low numbers that values are not visible in the charts, so numbers have been added with the phylum name. Sequencing projects are still biased towards over-representation of Ascomycota and under-representation of especially Chytridiomycota, and Blastocladiomycota.

A pie-chart visualization of ratio of species belonging to the various taxonomic fungal phyla on the listing of biomass degrading capacity is shown in Figure 3. The first 500 species are heavily dominated by ascomycetous species, with a few anaerobic chytrids (rumen fungi) prominently placed on top of the list; Basidiomycetes represented by only 22 species. In position 500–1000 the number of Basidiomycetes is growing. However, among the genome-sequenced species, listed from 1000 to 1932 in ranking of biomass degrading enzyme capacity the other non-ascomycota phyla starts to be more prominently represented. Overall enzyme degrading capacity of fungal phyla (beyond the Chytridio-, Asco- and Basidiomycota described above) are all low-ranking: Mucoromycota, highest ranking, *Rhizopus oryzae* 1329; Zoopagomycota, highest, *Basidiobolus meristosporus* 1419; Blastocladiomycota, highest, *Allomyces macrogynus* 1662; Cryptomycota, highest, *Rozella allomyces*, 1880; Microsporidia highest ranking, 1897; all out of a total of 1932 species analyzed.

### 3.10. Biomass Degrading Capacity of Iconic Species and Life Forms

*Fungi and fungal enzymes used industrially.* It is surprising to see that the species most frequently used in industrial biomass conversion are not found among the fungal species we here found to have the highest level of biomass conversion enzyme capacity: Species included of *Trichoderma* rank according to total biomass degrading capacity as numbers 916, 1072, 1112, 1113, and 1232 (respectively, for *T. virens*, *T. harzianum*, *T. viride*, *T. asperellum and T. reesei).* Species of *Aspergillus* rank higher, *A. oryzae* 424, *A. hancockii* 447, *A. flavus* 475, *A. terreus* 647 and *A. nidulans* 716, *A. niger* 795 and *A. fumigatus* 859. Therefore, do also the two basidiomycetes, *Ganoderma lucidum* 678 and *Trametes versicolor* 735. Notably, the industrial use of, e.g., species of *Aspergillus* or *Trichoderma* for producing blends of enzymes are chosen just as much for their high-performance growth characteristics in upscale fermenters as well as for their highly efficient enzyme secretion machinery. Not for their efficiency in biomass conversion by their native enzymes.

*White rot fungi.* Similarly, it is also surprising that some of the widely studied white rot fungi, known to produce, e.g., specific lignin-cellulose degrading and complex xylan degrading CAZymes are not among the topscoring in biomass degrading capacity: *Pleurotus ostreatus* 798, *Phanerochate chrysosporium* 632, *Armillaria mellea* 660. *Brown rot fungi.* As expected brown rot fungi are ranked low in enzymatic biomass degrading capacity: *Serpula lacrymans* 1471, *Coniophora puteana* 1039, *Fomitopsis pinicola* 1139.

*Wood degraders as compared to dung fungi and indoor fungi.* The most potent among the wood degraders is the inconspicuous species *Exidia glandulosa* 35 and *Xylaria striata* 88. Next level in enzymatic biomass conversion capacity, among wood degraders are species of *Hypoxylon* and *Calonectria* here ranked as 540 and 546. For comparison the dung fungi *Podospora comata* and *P. anserina* are ranked 587 and 588; while the indoor fungi *Stachybotrys chartarum* is ranked as 141, hereby being among the top biomass degrading capacity species.

*Edible fungi.* The enzyme biomass conversion capacity among edible fungi, calculated as total number of “Function;Family” observations deserves special attention. They are all possible candidates for being used for fermentation as they have a long track record of being consumed as food, neither posing risk to workers health nor representing a regulatory and safety obstacle for being used for food and feed. (Notably, allergenicity cannot a priori be ruled out). A selection of iconic edible fungi, all ranked among the upper half is here listed, indicating their ranking according to biomass conversion capacity: Chinese mushroom, *Auricularia auriculaejudae* (synonym *Hirneola*) 509; *Hiericium coralloides* 796, *Pleurotus ostreatus* 875, *Flammulina velutipes* 889, *Volvariella volvata* 949. In addition to such species, a large proportion of the edible mushrooms are placed in the second half of the ranking list, to be regarded as low in biomass conversion capacity. The late fall mushroom *Sarcomyxa edulis* 1136, *Cantharellus cibarius* 1221, *Fistulina hepatica* 1296, *Boletus edulis* 1276, *Agaricus bisporus* 1.335, *Sparassis crispa* 1404, *Tuber* sp., 1422 to 1531, *Lactarius deliciosus* 1470 and *Ustilago maydis* (corn smut) 1623.

*Insect cultivated fungi.* Among the most highly optimized (enzymatic digestive) biological systems in Nature is the co-evolution of termites and leaf cutter ants with basidiomycetous fungi. The termites and the leaf cutter ants are cultivating specific species of Basidiomycetous fungi to produce enzymes, which in two steps convert the collected plant biomass to nutritious fungal-derived protein and sugar; or seen from the opposite perspective: a biological system where the fungus has lured the termites or ants to provide it with continuous food supply, protected humid underground shelter and meticulous weeding, actively defending the fungal colony from intruding microbial infections. Leaf cutter ants cultivate species of *Leucoagaricus* [43]; the termites cultivate species of *Termitomyces*, both genera of Agaricomycetes. Surprisingly, a preferred species, *L. gongylophorus* rank as low as 1570 in total biomass degrading capacity, while other genome sequenced *Leucoagaricus* sp. included in this study rank as 903 and 758. Similarly, surprisingly, *Termitomyces eurrhizus* ranks as low as 1196; other, *T.* spp. rank even lower in enzyme degrading profile (e.g., 1302) in the current study. The fungi co-evolved with termites and leaf cutter ants may to a large extent have been optimized not only in enzyme profile but maybe more in growth characteristics (e.g., formation of highly structured gongylidiae). Which, together with the enzymes have led to the high biomass conversion, from plant to nutritious larval feed for the larvae so successfully sustaining the colony development.

*Thermophilic fungi, industrially exploited.* Enzymes from a number of thermophilic fungal species have been industrially exploited, explicitly for their high temperature stability. Selected thermophilic species included in this study: *Thermothelomyces thermophilum* here ranks 891; *Thermoascaceaca* sp. 1098; *Chaetomium thermophilum* 1135, *Thermoascus crustaceus* 1263; and *Thermomyces lanuginosus* 1646; *Thermomucor indicae-seudaticae* 1666. Even though these fungi do not have a very high biomass degrading capacity, their industrial exploitation has been very successful due to their thermostability.

*Iconic fungal plant pathogens*. Enzymatic degradation of plant cell wall components is seen as a part of the pathogenicity of many (or most) of the fungal species infecting and invading living plants. As here exemplified, their enzyme biomass degrading armaments are quite varying: *Mycena citricolor* are on the top of the overall total redundant listing, as third only after two anaerobic cow rumen fungi (see Table 1); *Ilyonectria mors-panaci* 97; *Eutypa lata* 119; *Stenocarpella maydis* 127; *Rice blast*, *Pyricularia oryzae* 570; *Pyrenophora tritici-repentes* 620; *Gauemannomyces tritici* 635; *Pyricularia grisea* 675; *Puccinia arachidis* 711; *Botrytis cinerea* 718; *Rhizoctonia solani* 775; *Venturia oleagina* 785; *Monilinia fructigena* 982; *Heterobasidion annosum* 1017; *Claviceps purpurea* 1018; *Tilletia indica* 1607; *Synchytrium endobioticum* (potato wart disease; *Chytridiomycota*) 1745; *Taphrina betulina* (witches broom of birch) 1786. Two groups of plant pathogens deserve special attention, the rusts and the smuts. Two plant pathogenic rust-species are among the fungi with the absolute lowest biomass degrading capacity: *Melampsora occidentalis*, ranking 1891, has two cellulose-active observations only and no other types of biomass degrading enzyme; *Endocronartium harknessii*, ranking 1892 has two pectin-active enzymes only; *Uromyces vicia-fabae* ranking 1893 has two cellulose-active observations only; and *Melampsora larici-populina*, ranking 1901 has one lignin-active “Function;Family” observation. However, other species of rust fungi have a richer enzyme capacity for breaking down plant cell walls, e.g., *Uromyces transversalis* ranking 665; *Puccinia arachidis* as 771; while *Puccinia graminis* ranks significantly lower, 1744; *Puccinia striiformis* 1828. Notably, no genome sequenced species of smut fungi have high capacity for biomass degradation, all are ranking very low with regard to biomass degrading capacity: *Ustilago trichophera* 1285; *Ustilago tritici* 1486; *Violaceomyces palustris* 1487; and *Ustilago esculenta* 1638. For species with such extremely low capacity for biomass degradation, elucidating the roles of the few enzyme observations present could give valuable information, elucidating the enzymatic substrate interaction between rust and smut fungi and their hosts.

*Mycorrhiza-forming fungi.* Through the ectomycorrhizal symbiosis, primarily with tree species, Basidiomycetous fungi do not need to live only on absorptive growth; however, the mycorrhizal fungi have been shown also to have a basic set of digestive plant cell wall degrading enzymes. Based on this conceptual understanding the following ranking of species on degradation capacity make good sense: *Boletus edulis* 1276; *Suillus luteus* 1307; *Lactarius indigo* 1331; *Paxillus involutus* 1379; *Russula* sp. 1469; *Xerocomus* sp. 1472; *Amanita* 1521; *Russula* 1568, 1638; *Amanita inopinata* 1849 *and A. jacksonii* 1852. The last two species have an extremely reduced portfolio of biomass degrading enzymes, *A. inopinata* has two enzyme “Function;Family” observations active on cellulose and seven on lignin; and for the latter, *A. jacksonii*, one on cellulose and eight, active on lignin.

*Endomycorrhiza-forming fungi.* Only rather few of this system-biology-wise highly important life form has been genome sequenced and included in this study. The sequenced species all rank rather closely, following the same pattern as ectomycorrhizal fungi: *Gigaspora margarita* 1378, *G. rosea* 1451, *Glomus cerebriforme* 1590, *Oehlia diaphana 1711.* Studies revealing the specific role of these very few enzyme “Function;Family” observations found in Endomycorrhizal fungi could be highly interesting; especially seen in the light this life form being so ecologically essential for entire habitats.

*Fungi used in food processing.* A few fungal species used in food processing, maturation and fermentation are included in this study. Among those are the following iconic species: *Penicillium camemberti* having a total of 919 “Function;Family” observations and *Penicillium roqueforti* 1156; *Aspergillus oryzae* 423, *Aspergillus sojae* 304 *and Rhizopus oryzae* 1329; the first two are used for highly optimized French cheese making; the latter three used in Japanese cuisine, misu, tempeh and soy sauce.

Fungi used in biological control of plant diseases. Against insects: *Metarhizium majus* 1264; *M. robertsii* 1273; *M.* sp. 1558; *Beauveria pseudobassiana* 1344; *B.* sp. 1371, 1372, 1398, 1399. Against fungal pathogens: *Clonostachys rosea*, being both an endophyte and a mycoparasite has a good armament also of plant cell wall degrading enzymes, ranking as high as 31 on the total biomass degrading capacity “Function;Family” observation list.

Fungal species as models used in mycological and genetic research. None of the fungal research model species are ranking as having an outstanding biomass degrading capacity: *Schizophyllum commune* 849; *Coprinopsis cinerea* 869; *Neurospora crassa* 1096; *Trametes (Pycnoporus) cinnabarina* 928; *Rhizophlyctis rosea* 935; *Arthrobotrys oligospora* 957; *Ustilago maydis* 1548; *Allomyces macrogynus* 1662; *Entomophthora muscae* 1701; *Rozella allomyces* 1880; *Catenaria anguillulae* 1863; *Batrachochytridum dendrabatidis* 1865; *B. salamandrivorans* 1874. However, one could also in this grouping include the rather high ranking of enzyme biomass degrading capacity and specificity diversity of species of Aspergillus (*A. oryzae*, *A. nidulans* etc.) used for biological production in industry, being species ranking high with regard to biomass conversion capacity (see above, Industrial used fungi).

*Black yeasts, halophiles and xerophiles.* Extremophilic species of black yeasts and molds, being both halophilic and xerophilic have not yet been exploited industrially to their full potential. One reason is that many of them are human pathogens and therefore neither safe nor permissibly to use in industry. For the non-pathogenic species of black yeasts, their industrial relevance is not primarily for a rich enzyme diversity of “Function;Family” observations. Their industrial relevance is connected to special molecular characteristics of tolerating extremely low water activity and also (related to) tolerating high concentration of salt. A selection of Black yeast species, included in this survey are here listed: *Aureobasidium pullulans* 679; *Hortaea thailandica* 1132; and the halophilic molds *Wallemia hederae* 1790; *W. mellicola* 1881.

*Human pathogens.* A high number of fungal human pathogens have been genome sequenced. As human cells do not have walls, enzymatic lignocellulosic biomass degrading capacity is not per se expected to be significant for their pathogenicity. Still, interesting observations, also of relevance for understanding their pathogenesis fully, can be achieved by analyzing their portfolio of CAZymes (see Table S1). Below is a selection of human pathogens, each of them ranked among the 1932 fungal genomes analyzed: *Aspergillus fumigatus* 859; *Trichotecium roseum* 987; *Sporothrix brasiliensis* 1007; *Trichosporon coremiiforme* 1267; *Cutaneotrichosporon dermatis* 1327; *Cryptococcus amylolentus* 1599; *Cryptococcus wingfieldii* 1656; *Cryptococcus gattii* 1789. Further several species included in this study are animal pathogens, e.g., *Raffaelea lauricola* 1006 pathogen of Beetles; *Piedraia hortae* 1750 on human and animal hair; *Malessezia* spp. 1791 a human and animal dermatophyte. *Halocinogenic fungus*, *Psilocybe* spp. rank as 417, 1057; the highly toxic fungus *Amanita phalloides* has ranking 1805. 

*Fungal production of pharmaceutical drugs*. Species of *Monascus* (e.g., *M. purpureus* ranking as 1514) and species of *Aspergillus* (especially *A. terreus* ranking as 647) have dominated the biological production of drugs to control human hyper-tension.

## 4. Discussion

### 4.1. Discussion and Conclusions

The fungal digestive enzyme battery is an integrated part of fungal evolutionary speciation, as the organism gains fitness advantage for growth and reproduction by efficiently mobilizing accessible substrates [14]. Efficiency in substrate mobilization depends on having the right type of enzyme proteins (stable and substrate-accessible), combined with having optimized portfolio of enzyme functions, suitable for the substrates, available in the ecosystem of the fungus. This combination of enzyme key features, type of protein and molecular function is the basis for the new methodological approach presented here for comparative ranking of fungi according to their biomass-digestive CAZyme profiles: The integrated “Function;Family” observation is the fitness feature that evolutionary pressure is targeting; having the right type of enzymes for the functions needed is a competitive advantage for absorptive metabolism of the heterotrophic fungal life-form. By accumulating “Function;Family” observations for each of the major cell wall components, in two modes, with and without observation redundancy, we can describe each genome by one number for unique observations and one number for total degradation capacity (incl redundant observations). This, both informative and evolutionary relevant as well as reductionistic approach enables comparative ranking of enzyme degrading capacity as well as richness in enzyme function specificities, hereby identifying enzyme hotspots (taxonomic and eco-physiological), across a high number of available fungal genome sequences. Notably, the time is right for such across Fungal Kingdom genome analysis, as species from all phyla, together representing most of the larger segments and life forms of the Fungal Kingdom by now have been sequenced and made available for the international scientific community, 1000 fungal genome community project by the US Department of Energy Joint Genome Institute and Mycocosm [6,44,45].

In the present study, the CAZyme “Function;Family” observation-based analysis was developed into two different types of ranking: Differentiating between richness in enzyme function specificity diversity, counting only unique observations; and the organismal total CAZyme degrading capacity including also redundant “Function;Family” observations. Using this in a Fungal-Kingdom-wide analysis, including comparative analysis of enzyme activities, targeting many substrates opens up for three additional types of conceptually new findings: (i) The factor by which the enzyme capacity is enhanced by organismal evolutionary development of several redundant “Function;Family” observations, varies between fungal phyla as well as between the different types of substrate targeted (here quantified as a Redundancy Multiplication Score). (ii) Substrate association analysis revealed new ranking of species, especially interesting for elucidating which species are excelling when summing up the pectin plus lignin number of observations. (iii) Analysis of intergeneric variation of CAZyme richness (made possible where multiple genome sequenced species were included) enabled the description of two different patterns. In some fungal genera, the enzyme profiles of the species rank quite closely with regard to richness in degrading capacity as well as in function diversity. While in other genera, the species are found to vary significantly with regard to enzyme profile richness, being spread almost from top to bottom of the ranking list.

The results obtained demonstrate that the “Function;Family” observation-based methodological approach opens for new and surprising insight into the richest fungal enzyme hotspots in Nature: It also provides significant new enzyme knowledge about fungal roles in degradation of substrates and in host interaction and pathogenicity. It stands out prominently that a dominant characteristic among the species identified to be richest in enzyme specificities and biomass degrading capacity is a strong and broad eco-physiological profile: Typically including ability to be both biotrophic and saprotrophic, able to attack all parts of the plants and several types of substrates: *M. citricolor* (Basidiomycota) a devastating pathogen on coffee, attacking all parts of the plant, not the least the berries; *V. longisporum* (Ascomycota) both soil-inhabiting and a devastating plant pathogen; *Coniochaeta* (Ascomycota) a pleomorphic yeast, both living as saprotroph and as tree pathogen, as well as human pathogen; *P. roridum* (Ascomycota), soil inhabiting and a facultative plant pathogen. *Diaporthe* sp. (Ascomycota), a devastating pathogen of a wide spectrum of species, invading all parts of the plants, green, wooden and seeds; biotrophic and necrotrophic die back. Notably, all of the above-mentioned species degrade dicots. A hypothesis based on the results here reported could be that among the richest enzyme profile species, the dicot-invading (or both dicot- and monocot-invading) fungi have a high capacity CAZyme portfolio dominated by a very large variety of pectin degrading enzymes as compared to monocot invading species.

Interestingly, one more essential character is prominent for the majority of the especially rich CAZyme producing species here identified. A surprisingly large fraction of the richest and most versatile, talented species with regard to enzyme specificity diversity as well as total enzyme degrading capacity is also characterized by being talented in production of bioactive metabolites, of which many are recognized as mycotoxins. This is the case for eight out of the ten top-ranking species of redundant observations (Table 1) and for all the ten unique observations top-ranking species (Table 2). One interpretation of this phenomenon is straight forward: Being extremely efficient in degrading (plant) biomass, goes hand in hand evolutionary with having a strong line of defense, in order to safeguard the mobilized nutritional elements for own use. Hereby creating increased fitness (competitiveness) by protecting the pool of monomeric and short sugar oligos formed as a result of the enzyme degradation. A new line of research, making integrated studies of digestive enzymes and metabolite profiles of fungi from genomes is emerging [46,47]. It seems as we are approaching having sufficient evidence for formulating hypothesis of examples co-development of the two major elements of the fungal secretome, enzyme proteins and biologically active secondary metabolites.

Another interesting result of this study is that several of Nature’s enzyme Hotspots are found in unexpected life forms. *Cadophora* sp. (Ascomycota), an endophyte belonging to the dark septate endophyte group (DSE) is among the highest ranked species (on both xylan and pectin). The reporting here is the first to give evidence that a member of the DSE ranks among the strongest species with regard to overall enzyme capacity and biomass specificity diversity. It is assumed that the DSE root-endophytes represent a special plant-fungus interaction; comparing the DSE profile of enzyme “Function;Family” observations with the profile of other types of symbiotic fungi might help us to better understand their system biology role, e.g., in carbon sequestration and cycling and ecosystem functioning [29,30]. Based on the current study we propose a possible new role for the DSE fungi, also serving as biomass degraders in the rhizosphere soil surrounding the host roots; and suggest that richness in enzyme function diversity could reflect adaptation and specialization of endophytes, broadly invading many types of plant tissue without causing devastating harm to their host plant.

Additionally, other species (here shown to have both the highest capacity and the broadest enzyme function specificity diversity), live in habitats and have ecophysiological specializations not earlier recognized as being hotspot for enzyme activities. It is highly surprising to find a black yeast, *Rachicladosporium antarcticum*, genome sequenced and annotated in 2017 [48], generally characterized as an endolithic “rock fungus”, to globally being among the most highly talented enzyme portfolio species. It could be suggested that living in such organic substrate-meager environments as this speacies does makes it highly essential to be able to efficiently degrade whatever become available. Other surprises among the hotspots are the basidiomycetes *Exidia glandulosa*, especially excelling on xylan and lignin living on wood and *Chalara*, (Ascomycota) a specialized xylan-degrader of coniferous needles. In contrast to this, the rather low ranking of all the to date industrially enzymatically exploited fungal species is truly surprising (*Trichoderma* sp. rank between 916 and 1232; *Aspergillus* sp. between 424 (*A. oryzae*) and 745 *(A. niger*). Notably, the industrial use of fungi for fermentation and for production of enzyme blends as well as single enzymes rely on much more than enzymes, such as strong growth performance in submerged fermentation and a highly efficient enzyme secretion mechanism. On the other hand, a reason that the highest enzyme performing species are understudied and almost totally unexploited is that many of them are known for posing risk of workers health when handled in the laboratory and even more facing regulatory issues in approval of upscaled production facilities. This significant inherent bias is, e.g., true for species of *Colletotrichum* and for species of *Paramyrothecium* [28], but recombinant enzyme production in approved workhorse hosts overcomes issues of simultaneous mycotoxin production.

Notably, the two species with the highest number of “Function;Family” observations belong to the early lineage, anaerobic, zoosporic rumen fungi (Neocallimagistomycota). The molecular organization of biomass degrading enzymes in the rumen microbiome (of both bacteria and fungi) known as the cellulosome, is unique in Nature. The cellulosome is fascinating in its complexity, highly optimized and specialized, through horizontal gene transfer evolution [49]. Furthermore, high number of “Function;Family” observations in the Neocallimastigomycota species may reflect not only high biomass degrading capacity but probably also a specific genome organization with a very high number of gene copies. Unfortunately, the rumen fungi have been left out from much of the recent rumen microbiome studies as only the prokaryotes have been included in the metagenome sequence analysis. However, new and interesting attempts have been made for developing methods for including also the anaerobic rumen fungi in the rumen microbiome analysis [50]. Based on the results of the current study, showing rumen fungi to be top ranking among all fungal species analyzed we hypothesize that the rumen fungi play a role as Key Stone species, playing a more prominent functional role than the ratio of fungal biomass and fungal DNA constitute as compared to bacterial biomass and DNA present in the rumen (fungi estimated to be between 8 and 12% of rumen microbiome) [51]. Thus far, none of the enzymes from rumen fungi or rumen bacteria have been industrially exploited. One reason being their anaerobic adaptation contrasting the predominantly aerobic industrial processing, another, that the complexity of the cellulosome structure is difficult to take advantage of in industrial fermentations and biorefinery processing. Most promising could be to use rumen fungi in a probiotic manner or possibly more advanced by combining it with special feed components (substrate oligoes and/or selected enzymes) to strengthen feed conversion and digestion at the same time as methane emission is reduced.

Recent studies have investigated different biomass degrading capacity among closely related species [52]. The results of the current study open for studying the variation among species, belonging to the same genus in both biomass-degrading capacity and function specificity diversity. More specifically, to elucidate this issue we analyzed comparatively the high number of species by ranking separately all species of the following five genera, *Aspergillus* (103), *Penicillium* (48), *Fusarium* (180), *Colletotrichum* (34). The preliminary conclusion from this is that variation among species within one genus apparently follows two different patterns. *Aspergillus*, *Fusarium* and *Penicillium* all have some species which have a very rich enzyme portfolio, in both total capacity and function diversity, ranked among the top 100. However, these genera also have species ranked in the middle part and even in the lower part of the total list of 1932 species. This result coincides with [14], where an enzyme profile relatedness analysis was made (by binary intergeneric comparison of “Function;Family” observations), dividing *Aspergillus* and *Penicillium* into sections. These sections were shown to be held together by the observations they shared to have as well as by the observations they shared not having. In contrast to this stands the ranking pattern of species of *Colletotricum*. This genus exhibits a pattern where the sequenced species are ranked rather close to each other, within a narrower part of the ranking list. Similar pattern is seen for species of *Diaporthe*, where in all seven species are included in this study, of which as much as four are placed among the top10 in biomass degrading capacity. Thus, the genus *Diaporthe* as such appears to be a real hotspot in enzyme biomass degrading capacity (see Table 1 and Table S1).

The term Redundancy Multiplication Score, RMS, is here introduced as a quantitative measure for characterizing different evolutionary trends in optimizing biomass degrading capacity (see Table 4): Highest RMS was found among anaerobic fungi and in the exceptional Basidiomycete, *M. citricolor*. The lowest RMS was found in species of the ascomycetous genera *Colletotricum, Diaporthe*, and *Coniochaeta*; notably, equally low RMS found for species of these three genera, both among enzymes active on the highly complex pectin substrate as well as on the much simpler cellulose. A preliminary conclusion is that RMS is primarily varying between fungal genera; and exceptionally high RMS is found in anaerobic fungi.

### 4.2. Applied Perspectives

Improved use of the global biological resources is urgently needed. We currently lose as much as approximately 35% as food waste [53] and estimated 15% more by not upgrading the food and non-food processing side-streams to their full potential [5]. Hereby all in all loosing, about half of all food and feed produced. This is a scary fact but most importantly, it provides a perspective of hope for improved food security by upgrading what was hitherto wasted; and it accentuates the need for new research in fungal carbohydrate active enzymes. Biotechnology, including use of enzymes to accomplish a range of conversions, is an integral part of accelerating the transition to a more circular economy, including improved use of the biological resources through increased resource circularity and production of bio-based substitutes for fossil-based materials and chemicals [54]. Plant biomass, bio- and agro-industrial waste streams, and even aquatic biomass and certain synthetic materials (e.g., plastic) must be utilized and/or recycled more efficiently to support the development of a sustainable bioeconomy. The target aim is increased “biologization” of the economy to help transform waste and materials into new products with defined, desirable properties [55]. Conscientious development of a sustainable bioeconomy provides an opportunity to decouple increased resource consumption from economic growth. Since the complexity and diversity of different biological substrates is reciprocated by the plethora of enzymes evolved to convert and degrade them, the diversity of enzymes is poised to help transform waste and materials into new products with defined, desirable properties. The new method presented in the present report for identifying the species, richest in biomass digestive enzymes can open for a wide spectrum of new inventions and new applications. A summary overview can be achieved by not distinguishing the specific ranking 1–10, but simply including all species with Top10 scorings on more than one substrate. Based on this principle the following Hotspot species overview is achieved (see Table 5).

As shown in Table 5, *P. roridum* has amazing industrial enzyme potential, excelling on all four substrates; if it was not for its toxicity profile. Would a knock-out or block of the mycotoxin synthesis pathways be a feasible, regulatory-acceptable strategy to pursue? Would this strategy be workable also for, e.g., *Colletotricum*?

In a broader perspective the results presented in the present study hint at a major avenue of new research for identification of novel fungal enzymes, function specificity diversity and degrading capacity hotspots: The data (i) provide an expanded basis for identifying new candidates for non-GMO production hosts for new types of biomass degrading enzyme blends, optimized by nature. The findings presented clearly outline that species for non-GMO enzyme blend production organisms should be chosen, depending on which primary cell wall components that are the main targets for biomass upgrading. (ii) help identifying candidate species for new whole-cell biocatalyst systems, suitable for biorefinery processing. (iii) provide a new foundation for identifying candidates suitable as new production host systems for recombinant production of mono-component enzymes and enzyme blends: blends inspired by linkage analysis, learning from Nature, how to optimize enzyme blends for specific types of biomass, see, e.g., *M. citricolor*, exceptional strong in pectin and lignin degradation but also efficient in cellulose and hemicellulose (symptoms in Figure 1). (iv) contribute to improved, evidence-based and intelligent strategies for understanding for discovery of new and improved enzymes and enzyme blends for efficient and value adding biomass conversion.

Increased understanding of the fungal CAZyme portfolio provides insight into the role of biomass degrading enzymes for fungi of different lifeforms, eco-physiological specializations and taxonomic affiliation; exemplified by the spectrum of iconic species identified as enzyme hotspots. The result of this study can therefore be used as basis for studying evolution of the fungal digestive enzyme system and for improving the understanding of possible co-evolution of enzymes and metabolite profiles in fungi and directly for providing a new conceptual understanding of the role of fungi in biomass conversion and carbon-recycling. Additionally, for finding new hotspots of biologically active secondary metabolites, e.g., for drug discovery. Analogously, whether there is any possible connection between fungal CAZyme profiles and mycotoxin production calls for further study. Experience from antimicrobial metabolite discovery (unpublished), revealed that fungi with a strong capacity for plant cell wall degradation is a hotspot also for finding antimicrobial metabolites. Studies next in line for learning from Nature is to further compare enzymes originating from different species and to look deeper into the different enzyme systems (e.g., different CAZyme profiles) employed by fungi to degrade biomass. Specifically, such additional work involves analyzing the LPMO profiles and elucidating the composition of the sets of “Function;Family” observations of the species here revealed to be global hotspots; and obviously to make a similar “Function;Family” observation-based analysis, for identifying bacterial hotspots with regard to enzyme function specificity diversity and overall degrading capacity.

## Figures and Tables

**Table 1:** Listing of the ten top-ranked species with regard to biomass degrading capacity, (all observations included), specifying the number of “Function;Family” observations. The table includes ranking according to “Total” (A), followed by ranking according to enzyme observations with affinity for each of the target substrates, Cellulose (B), Xylan (C), Pectin (D) and Lignin (E). The total list of observations for the analyzed 1932 genomes is given in Table S1.

A Ranking: Total	Taxonomy		Target Substrate of Encoded CAZymes				
Species	Class	Phylum	Cellulose	Pectin	Xylan	Lignin	Total
*Pecoramyces ruminatium*	Neocallimastigomycetes	Chytridiomycota	248	85	208	0	541
*Neocallimastix californiae*	Neocallimastigomycetes	Chytridiomycota	232	122	172	0	526
*Mycena citricolor*	Agaricomycetes	Basidiomycota	91	204	50	149	494
*Verticillium longisporum*	Sordariomycetes	Ascomycota	139	176	74	95	484
*Coniochaeta* sp. *2T2.1*	Sordariomycetes	Ascomycota	117	102	108	98	425
*Paramyrothecium roridum*	Sordariomycetes	Ascomycota	106	163	63	79	411
*Cadophora* sp. *DSE1049*	Leotiomycetes	Ascomycota	105	138	75	91	409
*Diaporthe ampelina*	Sordariomycetes	Ascomycota	116	129	58	97	400
*Diaporthe longicolla*	Sordariomycetes	Ascomycota	111	128	56	90	385
*Diaporthe* sp. *NJD1*	Sordariomycetes	Ascomycota	111	118	58	94	381
**B Ranking: Cellulose**	**Taxonomy**		**Target Substrate of Encoded CAZymes**				
**Species**	**Class**	**Phylum**	**Cellulose**	**Pectin**	**Xylan**	**Lignin**	**Total**
*Pecoramyces ruminatium*	Neocallimastigomycetes	Chytridiomycota	248	85	208	0	541
*Neocallimastix californiae*	Neocallimastigomycetes	Chytridiomycota	232	122	172	0	526
*Verticillium longisporum*	Sordariomycetes	Ascomycota	139	176	74	95	484
*Piromyces* sp. *E2*	Neocallimastigomycetes	Chytridiomycota	128	47	117	0	292
*Coniochaeta* sp. *2T2.1*	Sordariomycetes	Ascomycota	117	102	108	98	425
*Diaporthe ampelina*	Sordariomycetes	Ascomycota	116	129	58	97	400
*Diaporthe longicolla*	Sordariomycetes	Ascomycota	111	128	56	90	385
*Diaporthe* sp. *NJD1*	Sordariomycetes	Ascomycota	111	118	58	94	381
*Diaporthe capsici*	Sordariomycetes	Ascomycota	107	118	58	93	376
*Paramyrothecium roridum*	Sordariomycetes	Ascomycota	106	163	63	79	411
**C Ranking: Xylan**	**Taxonomy**		**Target Substrate of Encoded CAZymes**				
**Species**	**Class**	**Phylum**	**Cellulose**	**Pectin**	**Xylan**	**Lignin**	**Total**
*Pecoramyces ruminatium*	Neocallimastigomycetes	Chytridiomycota	248	85	208	0	541
*Neocallimastix californiae*	Neocallimastigomycetes	Chytridiomycota	232	122	172	0	526
*Piromyces* sp. *E2*	Neocallimastigomycetes	Chytridiomycota	128	47	117	0	292
*Coniochaeta* sp. *2T2.1*	Sordariomycetes	Ascomycota	117	102	108	98	425
*Cadophora* sp. *DSE1049*	Leotiomycetes	Ascomycota	105	138	75	91	409
*Verticillium longisporum*	Sordariomycetes	Ascomycota	139	176	74	95	484
*Rachicladosporium antarcticum*	Dothideomycetes	Ascomycota	68	32	72	56	228
*Piromyces finnis*	Neocallimastigo	Chytridiomycota	91	30	71	0	192
*Exidia glandulosa*	Agaricomycetes	Basidiomycota	82	61	70	114	327
*Chalara longipes*	Leotiomycetes	Ascomycota	94	82	69	79	324
**D Ranking: Pectin**	**Taxonomy**		**Target Substrate of Encoded CAZymes**				
**Species**	**Class**	**Phylum**	**Cellulose**	**Pectin**	**Xylan**	**Lignin**	**Total**
*Mycena citricolor*	Agaricomycetes	Basidiomycota	91	204	50	149	494
*Verticillium longisporum*	Sordariomycetes	Ascomycota	139	176	74	95	484
*Paramyrothecium roridum*	Sordariomycetes	Ascomycota	106	163	63	79	411
*Colletotrichum truncatum*	Sordariomycetes	Ascomycota	90	150	59	72	371
*Colletotrichum camelliae*	Sordariomycetes	Ascomycota	90	139	65	77	371
*Colletotrichum* sp. *COLG25*	Sordariomycetes	Ascomycota	90	139	63	76	368
*Colletotrichum karsti*	Sordariomycetes	Ascomycota	90	139	57	71	357
*Colletotrichum tropicale*	Sordariomycetes	Ascomycota	89	139	63	77	368
*Cadophora* sp. *DSE1049*	Leotiomycetes	Ascomycota	105	138	75	91	409
*Aspergillus latus*	Eurotiomycetes	Ascomycota	95	137	53	58	343
**E Ranking: Lignin**	**Taxonomy**		**Target Substrate of Encoded CAZymes**				
**Species**	**Class**	**Phylum**	**Cellulose**	**Pectin**	**Xylan**	**Lignin**	**Total**
*Mycena citricolor*	Agaricomycetes	Basidiomycota	91	204	50	149	494
*Exidia glandulosa*	Agaricomycetes	Basidiomycota	82	61	70	114	327
*Hymenopellis chiangmaiae*	Agaricomycetes	Basidiomycota	77	70	44	104	295
*Ganoderma boninense*	Agaricomycetes	Basidiomycota	77	36	44	104	261
*Neonothopanus nambi*	Agaricomycetes	Basidiomycota	58	18	33	102	211
*Coniochaeta* sp. *2T2.1*	Sordariomycetes	Ascomycota	117	102	108	98	425
*Ganoderma* sp. *BRIUMSc*	Agaricomycetes	Basidiomycota	62	31	34	98	225
*Diaporthe ampelina*	Sordariomycetes	Ascomycota	116	129	58	97	400
*Hymenopellis radicata*	Agaricomycetes	Basidiomycota	58	55	34	96	243
*Verticillium longisporum*	Sordariomycetes	Ascomycota	139	176	74	95	484

**Table 2:** Listing of the ten top-ranked species with regard to enzyme function specificity diversity (including only unique observations), specifying the number of “Function;Family” observations. The table includes ranking according to “Total” (A), followed by ranking according to enzyme observations with affinity for each of the target substrates, Cellulose (B), Xylan (C), Pectin (D) and lignin (E). (*) indicates more species with same number of observations. The total list of observations for the analyzed 1932 genomes is given in Table S1.

A Ranked: Total	Taxonomy		Target Substrate of Encoded CAZymes				
Species	Class	Phylum	Cellulose	Pectin	Xylan	Lignin	Total
*Colletotrichum* sp. *COLG25*	Sordariomycetes	Ascomycota	17	53	24	18	112
*Colletotrichum tropicale*	Sordariomycetes	Ascomycota	16	53	25	18	112
*Colletotrichum aenigma*	Sordariomycetes	Ascomycota	16	53	25	18	112
*Colletotrichum asianum*	Sordariomycetes	Ascomycota	16	53	24	18	111
*Colletotrichum* sp. *COLG31*	Sordariomycetes	Ascomycota	16	53	24	18	111
*Colletotrichum siamense*	Sordariomycetes	Ascomycota	16	53	23	18	110
*Paramyrothecium roridum*	Sordariomycetes	Ascomycota	18	49	25	17	109 *
*Colletotrichum viniferum*	Sordariomycetes	Ascomycota	16	51	23	19	109 *
*Colletotrichum fructicola*	Sordariomycetes	Ascomycota	15	52	24	18	109 *
*Colletotrichum gloeosporioides*	Sordariomycetes	Ascomycota	15	52	24	18	109 *
**B Ranked Cellulose**	**Taxonomy**		**Target substrate of encoded CAZymes**				
**Species**	**Class**	**Phylum**	**Cellulose**	**Pectin**	**Xylan**	**Lignin**	**Total**
*Paramyrothecium roridum*	Sordariomycetes	Ascomycota	18	49	25	17	109
*Gliomastix tumulicola*	Sordariomycetes	Ascomycota	18	40	24	15	97
*Hymenoscyphus herbarum*	Leotiomycetes	Ascomycota	18	43	22	19	102
*Aaosphaeria arxii*	Dothideomycetes	Ascomycota	18	41	22	16	97
*Memnoniella echinata*	Sordariomycetes	Ascomycota	18	41	21	17	97
*Clavariopsis aquatica*	Sordariomycetes	Ascomycota	18	35	19	18	90
*Xylaria striata*	Sordariomycetes	Ascomycota	17 *	43	25	17	102
*Colletotrichum* sp. *COLG25*	Sordariomycetes	Ascomycota	17 *	53	24	18	112
*Paraphaeosphaeria sporulosa*	Dothideomycetes	Ascomycota	17 *	41	24	17	99
*Stagonosporopsis tanaceti*	Dothideomycetes	Ascomycota	17 *	46	23	18	104
**C Ranked: Xylan**	**Taxonomy**		**Target substrate of encoded CAZymes**				
**Species**	**Class**	**Phylum**	**Cellulose**	**Pectin**	**Xylan**	**Lignin**	**Total**
*Cadophora* sp. *DSE1049*	Leotiomycetes	Ascomycota	16	45	26	16	103
*Paramyrothecium roridum*	Sordariomycetes	Ascomycota	18	49	25	17	109
*Xylaria striata*	Sordariomycetes	Ascomycota	17	43	25	17	102
*Colletotrichum tropicale*	Sordariomycetes	Ascomycota	16	53	25	18	112
*Colletotrichum aenigma*	Sordariomycetes	Ascomycota	16	53	25	18	112
*Clonostachys rosea*	Sordariomycetes	Ascomycota	16	45	25	15	101
*Pleosporales* sp. *UM 1110 2012*	Dothideomycetes	Ascomycota	16	39	25	17	97
*Gliomastix tumulicola*	Sordariomycetes	Ascomycota	18	40	24 *	15	97
*Colletotrichum* sp. *COLG25*	Sordariomycetes	Ascomycota	17	53	24 *	18	112
*Paraphaeosphaeria sporulosa*	Dothideomycetes	Ascomycota	17	41	24 *	17	99
**D Ranked: Pectin**	**Taxonomy**		**Target substrate of encoded CAZymes**				
**Species**	**Class**	**Phylum**	**Cellulose**	**Pectin**	**Xylan**	**Lignin**	**Total**
*Colletotrichum tropicale*	Sordariomycetes	Ascomycota	16	53	25	18	112
*Colletotrichum aenigma*	Sordariomycetes	Ascomycota	16	53	25	18	112
*Colletotrichum* sp. *COLG25*	Sordariomycetes	Ascomycota	17	53	24	18	112
*Colletotrichum asianum*	Sordariomycetes	Ascomycota	16	53	24	18	111
*Colletotrichum* sp. *COLG31*	Sordariomycetes	Ascomycota	16	53	24	18	111
*Colletotrichum siamense*	Sordariomycetes	Ascomycota	16	53	23	18	110
*Colletotrichum fructicola*	Sordariomycetes	Ascomycota	15	52	24	18	109
*Colletotrichum gloeosporioides*	Sordariomycetes	Ascomycota	15	52	24	18	109
*Colletotrichum viniferum*	Sordariomycetes	Ascomycota	16	51 *	23	19	109
*Colletotrichum camelliae*	Sordariomycetes	Ascomycota	16	51 *	23	18	108
**E Ranked: Lignin**	**Taxonomy**		**Target substrate of encoded CAZymes**				
**Species**	**Class**	**Phylum**	**Cellulose**	**Pectin**	**Xylan**	**Lignin**	**Total**
*Colletotrichum viniferum*	Sordariomycetes	Ascomycota	16	51	23	19 *	109
*Colletotrichum musae*	Sordariomycetes	Ascomycota	16	47	22	19 *	104
*Fusarium oxysporum*	Sordariomycetes	Ascomycota	16	44	22	19 *	101
*Hymenoscyphus herbarum*	Leotiomycetes	Ascomycota	18	43	22	19 *	102
*Fusarium oxysporum*	Sordariomycetes	Ascomycota	15	42	21	19 *	97
*Eutypa lata*	Sordariomycetes	Ascomycota	16	39	21	19 *	95
*Auricularia subglabra*	Agaricomycetes	Basidiomycota	16	23	21	19 *	79
*Hymenoscyphus salicellus*	Leotiomycetes	Ascomycota	16	42	20	19 *	97
*Hymenoscyphus infarciens*	Leotiomycetes	Ascomycota	16	40	20	19 *	95
*Auricularia cornea*	Agaricomycetes	Basidiomycota	17	25	20	19 *	81

**Table 3:** Enzyme profile of the 10 species with highest “Pectin+Lignin” biomass degrading capacity. Number of Pectin plus lignin observations is listed in right hand column. One basidiomycete (*M. citricolor*) and one Ascomycete (*V. longisporum*) stands out to be unique among all 1932 species analyzed. The heatmap shows the highly varying enzyme profile found in the species with highest total degrading capacity on pectin and lignin.

Species	Class	Phylum	Cellulose	Pectin	Xylan	Lignin	Total	Pectin + Lignin
*Mycena citricolor*	Agaricomycetes	Basidiomycota	91	204	50	149	494	353
*Verticillium longisporum*	Sordariomycetes	Ascomycota	139	176	74	95	484	271
*Paramyrothecium roridum*	Sordariomycetes	Ascomycota	106	163	63	79	411	242
*Cadophora* sp. *DSE1049*	Leotiomycetes	Ascomycota	105	138	75	91	409	229
*Diaporthe ampelina*	Sordariomycetes	Ascomycota	116	129	58	97	400	226
*Colletotrichum truncatum*	Sordariomycetes	Ascomycota	90	150	59	72	371	222
*Lachnum nothofagi*	Leotiomycetes	Ascomycota	92	124	60	95	371	219
*Diaporthe longicolla*	Sordariomycetes	Ascomycota	111	128	56	90	385	218
*Colletotrichum camelliae*	Sordariomycetes	Ascomycota	90	139	65	77	371	217
*Colletotrichum tropicale*	Sordariomycetes	Ascomycota	89	139	63	77	368	217

**Table 4:** Left column is names of top scoring sets of species with total-ranked observations (including redundant observations); the scores are given in second column. The third column from the left is the number of unique “Function;Family” observations of the same sets of degrading capacity Top ranking species. The right-hand column is the ratio, Redundant over Unique observations, named as Redundancy Multiplication Score. * marks the species/substrate with the strongest (>8) Redundancy Multiplication factor; ¤ marks the species with the lowest (<3) Redundancy Multiplication Score.

		All Observations	Only Unique Observations		Redundancy Multiplication Score
**TOTAL**					
*Pecoramyces ruminatium*		208		16	13.0 *
*Neocallimastix californiae*		172		15	11.5 *
*Mycena citricolor*		117		15	7.8
*Verticillium longisporum*		108		22	4.9
*Coniochaeta* sp. *2T2.1*		75		26	2.9
*Paramyrothecium roridum*		74		18	4.1
*Cadophora* sp. *DSE1049*		72		18	4.0
*Diaporthe ampelina*		71		10	7.1
*Diaporthe longicolla*		70		20	3.5
*Diaporthe* sp. *NJDP1*		69		23	3.0
**Cellulose**					
*Pecoramyces ruminatium*		204		24	8.5 *
*Neocallimastix californiae*		176		37	4.8
*Verticillium longisporum*		163		49	3.3
*Piromyces* sp. *E2*		150		45	3.3
*Coniochaeta* sp. *2T2.1*		139		51	2.7¤
*Diaporthe ampelina*		139		53	2.6¤
*Diaporthe longicola*		139		49	2.8¤
*Diaporthe* sp. *NJD1*		139		53	2.6¤
*Diaporthe capsici*		138		45	3.1
*Paramyrothecium roridum*		137		39	3.5
**Xylan**					
*Pecoramyces ruminatium*		149		18	8.3 *
*Neocallimastix californiae*		114		16	7.1
*Piromyces* sp. *E2*		104		17	6.1
*Coniochaeta* sp. *2T2.1*		104		15	6.9
*Cadophora* sp. *DSE1049*		102		16	6.4
*Verticillium longisporum*		98		13	7.5
*Rachicladosporium antarcticum*		98		14	7.0
*Piromyces finnis*		97		18	5.4
*Exidia glandulosa*		96		19	5.1
*Chalara longipes*		95		18	5.3
**Pectin**					
*Mycena citricolor*		204		24	8.5 *
*Verticillium longisporum*		176		37	4.8
*Paramyrothecium roridum*		163		49	3.3
*Colletotrichum truncatum*		150		45	3.3
*Colletotrichum camelliae*		139		51	2.7¤
*Colletotrichum* sp. *COLG25*		139		53	2.6¤
*Colletotrichum karsti*		139		49	2.8¤
*Colletotrichum tropicale*		139		53	2.6¤
*Cadophora* sp. *DSE1049*		138		45	3.1
*Aspergillus latus*		137		39	3.5
**Lignin**					
*Mycena citricolor*		149		18	8.3 *
*Exidia glandulosa*		114		16	7.1
*Hymenopellis chiangmaiae*		104		17	6.1
*Ganoderma boninense*		104		15	6.9
*Neonothopanus nambi*		102		16	6.4
*Coniochaeta* sp. *2T2.1*		98		13	7.5
*Ganoderma* sp. *BRIUMSc*		98		14	7.0
*Diaporthe ampelina*		97		18	5.4
*Hymenopellis radicata*		96		19	5.1
*Verticillium longisporum*		95		18	5.3

**Table 5:** Overview of the fungal species, which on two or more substrates rank among Top10 of all 1932 fungal genomes analyzed; Total carbohydrate biomass degrading capacity: According to total degrading capacity, including all observations;Function specificity diversity: according to richness in function specificity diversity of CAZymes. Two species stands out, *V. longisporum* in total degrading capacity on all four substrates; and *Colletotrichum COLG25* in enzyme function specificity diversity.

Total Carbohydrate Biomass Degrading Capacity					
		Target Substrate of Encoded CAZymes			
*Verticillium longisporum*	Ascomycota	Cellulose	Xylan	Lignin	Pectin
*Coniochaeta* sp.	Ascomycota	Cellulose	Xylan	Lignin	
*Pecaromyces ruminatum*	Neocallimastigo	Cellulose	Xylan		
*Neocallimastix california*	Neocallimastigo	Cellulose	Xylan		
*Mycena citricolor*	Basidiomycota	Pectin	Lignin		
*Paramyrothecium roridum*	Ascomycota	Cellulose	Xylan		
*Cadophora* sp.	Ascomycota	Xylan	Pectin		
*Diaporthe ampelina*	Ascomycota	Cellulose	Lignin		
*Exidia glandulosa*	Basidiomycota	Xylan	Lignin		
**Function specificity diversity**					
		**Target substrate of encoded CAZymes**			
*Colletotricum COLG25*	Ascomycota	Cellulose	Xylan	Pectin	
*Paramyrothecium roridum*	Ascomycota	Cellulose	Xylan		
*Gliomastix tumulicola*	Ascomycota	Cellulose	Xylan		
*Colletotrichum viniferum*	Ascomycota	Pectin	Lignin		
*Colletotrichum tropicale*	Ascomycota	Xylan	Lignin		
*Colletotrichum aenigma*	Ascomycota	Xylan	Pectin		
*Hymenoschuphus herbarum*	Ascomycota	Cellulose	Lignin		
*Xylaria stricta*	Ascomycota	Cellulose	Xylan		
*Paraphaerosphaeria sporulosa*	Ascomycota	Cellulose	Xylan		

## Data Availability

Not applicable.

## Supplementary Materials

The following are available online at https://www.mdpi.com/2309-608X/7/3/207/s1, Table S1: List of the rank of all 1932 fungal species/strains analyzed with regard to biomass degrading capacity, i.e., total number of “Function;Family” observations based on bioinformatics analysis of the fungal genome sequences (data supporting Table 1); Table S2: List of the rank of all 1932 fungal species/strains with regard to total number of unique “Function;Family” observations, reflecting the enzyme function specificity diversity of all the fungal genomes analyzed. Table S3: Categorization of “Family;Function” of enzymes in relation to substrate association.
