# Supplementary material for: New Method for Identifying Fungal Kingdom Enzyme Hotspots from Genome Sequences
Source: J Fungi (Basel). 2021 Mar 11;7(3):207. doi: 10.3390/jof7030207 (PMC8000046; doi:10.3390/jof7030207)
Supplement: Supplementary file 1 [file jof-07-00207-s001.pdf]

Supplementary Materials

Journal of Fungi

New Method for identifying Fungal Kingdom Enzyme Hotspots from Genome Sequences

Lene Lange[1], Kristian Barrett[2], and Anne S. Meyer[2]

[1] BioEconomy, *Research & Advisory*, Copenhagen, 2500 Valby, Denmark; lene.lange2@gmail.com (L.L)
[2] Section for Protein Chemistry and Enzyme Technology, Department of Biotechnology and Biomedicine, Building 221, Technical University of Denmark, DK-2800 Kgs. Lyngby, Denmark; kbaka@dtu.dk (KB), asme@dtu.dk (ASM)

Table S1. List of the rank of all 1.932 fungal species/strains with regard to biomass degrading capacity based on bioinformatics analysis of their genome sequence. The species are ranked with regard to the Total number of "Function;Family" observations, yet specifying the total number of "Function;Family" observations in each substrate category. The data shown is the full list of data supporting Table 1 in the main manuscript.

No	Species	Class	Phylum	Cellulose	Pectin	Xylan	Lignin	Total	ACC	Submitter
1	*Pecoramyces ruminatium*	Neocallimastigo	Chytridio	248	85	208	0	541	GCA_000412615.1	Oklahoma State University
2	*Neocallimastix californiae*	Neocallimastigo	Chytridio	232	122	172	0	526	GCA_002104975.1	DOE Joint Genome Institute
3	*Mycena citricolor*	Agarico	Basidio	91	204	50	149	494	GCA_003987915.1	Universidade de Sao Paulo
4	*Verticillium longisporum*	Sordario	Asco	139	176	74	95	484	GCA_001268165.1	SLU
5	*Coniochaeta sp. 2T2.1*	Sordario	Asco	117	102	108	98	425	GCA_009194965.1	DOE Joint Genome Institute
6	*Paramyrothecium roridum*	Sordario	Asco	106	163	63	79	411	GCA_003012165.1	USDA, ARS, NCAUR
7	*Cadophora sp. DSE1049*	Leotio	Asco	105	138	75	91	409	GCA_003073865.1	DOE Joint Genome Institute
8	*Diaporthe ampelina*	Sordario	Asco	116	129	58	97	400	GCA_001630405.1	Bangalore University
9	*Diaporthe longicolla*	Sordario	Asco	111	128	56	90	385	GCA_000800745.1	Purdue University
10	*Diaporthe sp. NJD1*	Sordario	Asco	111	118	58	94	381	GCA_013842865.1	Anhui Agricultural University
11	*Diaporthe capsici*	Sordario	Asco	107	118	58	93	376	GCA_013364905.1	Sichuan agricultural university
12	*Diaporthe sp. HANT25*	Sordario	Asco	106	123	57	88	374	GCA_013435955.1	Faculty of Science, Mahidol University
13	*Lachnum nothofagi*	Leotio	Asco	92	124	60	95	371	GCA_003988875.1	Manaaki Whenua Landcare Research
14	*Colletotrichum camelliae*	Sordario	Asco	90	139	65	77	371	GCA_011947485.1	Central South University of Forestry and Technology

15	*Colletotrichum truncatum*	Sordario	Asco	90	150	59	72	371	GCA_014235925.1	University of Salamanca
16	*Colletotrichum sp. COLG25*	Sordario	Asco	90	139	63	76	368	GCA_009801095.1	University of Agriculture, Faisalabad Pakistan.
17	*Colletotrichum tropicale*	Sordario	Asco	89	139	63	77	368	GCA_013201785.1	RIKEN
18	*Colletotrichum fructicola*	Sordario	Asco	89	137	62	77	365	GCA_000319635.2	RIKEN Plant Science Center
19	*Colletotrichum asianum*	Sordario	Asco	89	136	61	78	364	GCA_009806415.1	Northwest A&F University
20	*Phialocephala subalpina*	Leotio	Asco	96	118	62	87	363	GCA_900073065.1	Institute of Bioinformatics and Systems Biology
21	*Pestalotiopsis fici*	Sordario	Asco	92	128	63	80	363	GCA_000516985.1	Institute of microbiology, chinese academy of sciences
22	*Colletotrichum siamense*	Sordario	Asco	87	137	62	76	362	GCA_013390195.1	RIKEN
23	*Colletotrichum gloeosporioides*	Sordario	Asco	90	134	59	76	359	GCA_003243855.1	Nanjing Forestry University
24	*Colletotrichum viniferum*	Sordario	Asco	88	135	59	77	359	GCA_013201765.1	RIKEN
25	*Neopestalotiopsis sp. 37M*	Sordario	Asco	96	118	54	89	357	GCA_011058875.1	University of Campinas
26	*Colletotrichum sp. COLG31*	Sordario	Asco	92	132	55	78	357	GCA_009800995.1	University of Agriculture, Faisalabad Pakistan.
27	*Colletotrichum karsti*	Sordario	Asco	90	139	57	71	357	GCA_011947395.1	Central South University of Forestry and Technology
28	*Colletotrichum aenigma*	Sordario	Asco	86	131	62	75	354	GCA_013390185.1	RIKEN
29	*Memnoniella echinata*	Sordario	Asco	99	123	60	67	349	GCA_001599555.1	RIKEN Center for Life Science Technologies, Division of Genomic Technologies
30	*Aspergillus latus*	Eurotio	Asco	95	137	53	58	343	GCA_013306195.1	Vanderbilt University
31	*Clonostachys rosea*	Sordario	Asco	95	121	67	56	339	GCA_000963775.2	Chinese Academy of Agricultural Sciences
32	*Colletotrichum tofieldiae*	Sordario	Asco	89	116	57	71	333	GCA_001618725.1	Max Planck Genome Center Cologne
33	*Colletotrichum musicola*	Sordario	Asco	86	125	53	67	331	GCA_014235935.1	University of Salamanca
34	*Colletotrichum musae*	Sordario	Asco	82	118	52	77	329	GCA_002814275.1	CAPES
35	*Exidia glandulosa*	Agarico	Basidio	82	61	70	114	327	GCA_001632375.1	DOE Joint Genome Institute
36	*Nectria sp. B-13*	Sordario	Asco	94	124	59	50	327	GCA_002682825.1	Anhui University
37	*Colletotrichum scovillei*	Sordario	Asco	83	118	57	69	327	GCA_011075155.1	Tianjin Institute of Plant Protection
38	*Colletotrichum simmondsii*	Sordario	Asco	81	118	59	66	324	GCA_001563135.1	Universidad de Salamanca
39	*Chalara longipes*	Leotio	Asco	94	82	69	79	324	GCA_009732865.1	DOE Joint Genome Institute
40	*Colletotrichum plurivorum*	Sordario	Asco	82	125	49	67	323	GCA_014235945.1	University of Salamanca
41	*Colletotrichum acutatum*	Sordario	Asco	82	115	57	69	323	GCA_001593745.1	Seoul National University
42	*Diaporthe aspalathi*	Sordario	Asco	90	108	47	78	323	GCA_001447215.1	Beltsville Agricultural Research Center
43	*Pestalotiopsis sp. JCM 9685*	Sordario	Asco	89	104	49	81	323	GCA_001599175.1	RIKEN Center for Life Science Technologies, Division of Genomic Technologies
44	*Colletotrichum incanum*	Sordario	Asco	85	115	54	68	322	GCA_001625285.1	Max Planck Genome Center Cologne

45	*Verticillium zaregamsianum*	Sordario	Asco	100	124	49	47	320	GCA_002851755.1	Wageningen University & Research
46	*Colletotrichum nymphaeae*	Sordario	Asco	80	116	57	66	319	GCA_001563115.1	Universidad de Salamanca
47	*Colletotrichum coccodes*	Sordario	Asco	85	114	52	67	318	GCA_002249775.1	USDA-ARS
48	*Colletotrichum fioriniae*	Sordario	Asco	79	117	55	66	317	GCA_002930455.1	VIB
49	*Colletotrichum sojae*	Sordario	Asco	79	121	51	65	316	GCA_014235955.1	University of Salamanca
50	*Calonectria naviculata*	Sordario	Asco	86	117	53	58	314	GCA_003031705.1	USDA-ARS
51	*Ilyonectria destructans*	Sordario	Asco	92	120	40	62	314	GCA_001913115.1	Northeast Normal University
52	*Hyaloscypha variabilis*	Leotio	Asco	91	85	64	73	313	GCA_002865655.1	DOE Joint Genome Institute
53	*Byssochlamys sp. IMV 00045*	Eurotio	Asco	88	103	54	66	311	GCA_001931905.2	Jet Propulsion Laboratory, California Institute of Technology
54	*Fusarium sp. NRRL 22101*	Sordario	Asco	92	104	46	67	309	GCA_013010345.1	US Department of Agriculture, Agriculture Research Service
55	*Colletotrichum salicis*	Sordario	Asco	77	114	48	68	307	GCA_001563125.1	Universidad de Salamanca
56	*Gliomastix tumulicola*	Sordario	Asco	83	108	63	53	307	GCA_001599755.1	RIKEN Center for Life Science Technologies, Division of Genomic Technologies
57	*Fusarium decemcellulare*	Sordario	Asco	76	119	50	62	307	GCA_013266205.1	US Department of Agriculture, Agriculture Research Service
58	*Dactylonectria torresensis*	Sordario	Asco	78	128	48	52	306	GCA_011426275.1	AIT-Austrian Institute of Technology
59	*Calonectria pseudoturangicola*	Sordario	Asco	85	110	52	58	305	GCA_013403825.1	FABI
60	*Colletotrichum sp. JS-367*	Sordario	Asco	80	109	52	64	305	GCA_003122705.1	National Institute of Biological Resources
61	*Penicillium sp. CF01*	Eurotio	Asco	88	110	37	67	302	GCA_005250745.1	INAIL-Research Area
62	*Verticillium klebahnii*	Sordario	Asco	90	116	49	46	301	GCA_002851715.1	Wageningen University & Research
63	*Colletotrichum capsici*	Sordario	Asco	74	120	52	55	301	GCA_011947275.1	KLEF Deemed to be University
64	*Corynespora cassiicola*	Dothideo	Asco	81	100	44	76	301	GCA_900169545.1	UNIVERSITY OF BRISTOL
65	*Articulospora tetracladia*	Leotio	Asco	75	122	46	58	301	GCA_003415645.1	Nakdonggang National Institute of Biological Resources
66	*Dactylonectria macrodidyma*	Sordario	Asco	76	123	49	52	300	GCA_000935225.1	USDA-ARS
67	*Hortaea werneckii*	Dothideo	Asco	80	98	62	59	299	GCA_002127715.1	University of California, Riverside
68	*Verticillium albo-atrum*	Sordario	Asco	92	114	46	47	299	GCA_002851705.1	Wageningen University & Research
69	*Colletotrichum trifolii*	Sordario	Asco	74	116	49	60	299	GCA_004367215.1	RIKEN
70	*Phaeoacremonium minimum*	Sordario	Asco	84	94	63	57	298	GCA_000392275.1	UC Davis
71	*Hymenopellis chiangmaiae*	Agarico	Basidio	77	70	44	104	295	GCA_003314085.1	Kunming University of Science and Technology
72	*Fusarium sp. JS1030*	Sordario	Asco	72	110	53	60	295	GCA_000966855.1	Seoul National University
73	*Verticillium tricorpus*	Sordario	Asco	86	114	50	45	295	GCA_002851695.1	Wageningen University & Research

74	*Pezicula radicicola*	Leotio	Asco	78	103	30	83	294	GCA_003008705.1	The University of Texas Health Science Center at Houston
75	*Colletotrichum destructivum*	Sordario	Asco	77	110	46	61	294	GCA_009900065.1	Chinese Academy of Agricultural Sciences
76	*Verticillium isaacii*	Sordario	Asco	87	112	50	45	294	GCA_002851775.1	Wageningen University & Research
77	*Colletotrichum sidae*	Sordario	Asco	73	114	47	59	293	GCA_004367935.1	RIKEN
78	*Colletotrichum spinosum*	Sordario	Asco	74	113	47	59	293	GCA_004366825.1	RIKEN
79	*Zopfia rhizophila*	Dothideo	Asco	85	78	56	74	293	GCA_010093925.1	DOE Joint Genome Institute
80	*Aspergillus sp. MA 6041*	Eurotio	Asco	78	104	46	64	292	GCA_003138005.1	University of Natural Resources and Life Sciences, Vienna
81	*Ophiognomonia clavigignenti-juglandacearum*	Sordario	Asco	77	94	46	75	292	GCA_003671545.1	Colorado State U
82	*Piromyces sp. E2*	Neocallimastigo	Chytridio	128	47	117	0	292	GCA_002157105.1	JGI
83	*Calonectria leucothoes*	Sordario	Asco	77	105	51	58	291	GCA_002179835.1	USDA-ARS
84	*Colletotrichum orbiculare*	Sordario	Asco	71	114	47	59	291	GCA_000350065.2	RIKEN Plant Science Center
85	*Phialocephala scopiformis*	Leotio	Asco	80	76	52	80	288	GCA_001500285.1	DOE Joint Genome Institute
86	*Pseudopyrenochaeta lycopersici*	Dothideo	Asco	86	90	43	67	286	GCA_003313425.1	University of Verona
87	*Colletotrichum higginsianum*	Sordario	Asco	74	108	42	60	284	GCA_001672515.1	GATC Biotech AG
88	*Xylaria striata*	Sordario	Asco	76	81	53	72	282	GCA_002749545.1	Zhejiang University
89	*Calonectria fujianensis*	Sordario	Asco	77	105	44	56	282	GCA_013406965.1	FABI
90	*Nigrograna mackinnonii*	Dothideo	Asco	87	63	55	77	282	GCA_001007845.1	Yale University
91	*Pseudogymnoascus sp. VKM F-4519 (FW-2642)*	Leotio	Asco	77	102	62	40	281	GCA_000750935.1	Moscow State University
92	*Fusarium sp. NRRL 66182*	Sordario	Asco	67	110	53	50	280	GCA_013266265.1	US Department of Agriculture, Agriculture Research Service
93	*Calonectria honghensis*	Sordario	Asco	77	104	45	54	280	GCA_013403855.1	FABI
94	*Diaporthe helianthi*	Sordario	Asco	77	96	45	62	280	GCA_001702395.2	University of Western Brittany
95	*Fusarium oxysporum*	Sordario	Asco	74	101	49	55	279	GCA_002233775.1	University of Amsterdam
96	*Calonectria aciculata*	Sordario	Asco	76	103	46	54	279	GCA_013406995.1	FABI
97	*Ilyonectria mors-panacis*	Sordario	Asco	80	108	40	51	279	GCA_002991585.1	Zhejiang Sci-Tech University
98	*Calonectria pseudoreteaudii*	Sordario	Asco	78	101	43	56	278	GCA_001879505.1	Fujian Agriculture and Forestry University
99	*Fusarium nematophilum*	Sordario	Asco	75	107	44	51	277	GCA_013623595.1	US Department of Agriculture, Agriculture Research Service
100	*Colletotrichum chlorophyti*	Sordario	Asco	67	111	42	57	277	GCA_001937105.1	RIKEN
101	*Fusarium oxysporum*	Sordario	Asco	72	98	51	54	275	GCA_001652425.1	CSIRO
102	*Cairneyella variabilis*	Leotio	Asco	76	88	39	71	274	GCA_001625345.1	CSIRO
103	*Coleophoma cylindrospora*	Leotio	Asco	79	103	27	64	273	GCA_003369665.1	The University of Texas Health Science Center at Houston
104	*Stagonosporopsis tanaceti*	Dothideo	Asco	60	111	46	55	272	GCA_000812845.1	The University of Melbourne
105	*Fusarium oxysporum*	Sordario	Asco	66	97	49	58	270	GCA_005930515.1	RIKEN
106	*Paraphaeosphaeria sporulosa*	Dothideo	Asco	74	80	52	64	270	GCA_001642045.1	DOE Joint Genome Institute

107	*Fusarium oxysporum*	Sordario	Asco	70	93	48	58	269	GCA_012610815.1	Contact; Kurt. A. Zeller, USDA-APHIS-PPQ-S&T
108	*[Nectria] haematococca*	Sordario	Asco	79	97	44	49	269	GCA_010015875.1	Lishui University
109	*Pseudogymnoascus sp.* VKM F-4520 *(FW-2644)*	Leotio	Asco	80	94	51	44	269	GCA_000750995.1	Moscow State University
110	*Colletotrichum sansevieriae*	Sordario	Asco	69	100	43	57	269	GCA_002749775.1	Kagoshima University
111	*Oidiodendron maius*	Leotio	Asco	66	100	51	51	268	GCA_000827325.1	JGI
112	*Fusarium oxysporum*	Sordario	Asco	71	100	48	49	268	GCA_001702785.1	University of Amsterdam
113	*Fusarium sp. NRRL 62610*	Sordario	Asco	82	92	40	54	268	GCA_013186425.2	US Department of Agriculture, Agriculture Research Service
114	*Fusarium oxysporum*	Sordario	Asco	75	91	46	55	267	GCA_000260075.2	Broad Institute
115	*Fusarium oxysporum*	Sordario	Asco	68	99	51	49	267	GCA_003615085.1	NIAB-EMR
116	*Cudoniella acicularis*	Leotio	Asco	77	70	46	74	267	GCA_013054445.1	TU Dresden - IHI Zittau
117	*Beverwykella pulmonaria*	Dothideo	Asco	87	60	44	76	267	GCA_001599595.1	RIKEN Center for Life Science Technologies, Division of Genomic Technologies
118	*Margaritispora aquatica*	Leotio	Asco	63	112	42	50	267	GCA_007644065.1	Nakdonggang National Institute of Biological Resources
119	*Eutypa lata*	Sordario	Asco	82	68	44	72	266	GCA_000349385.1	UC Davis
120	*Pleosporales sp. UM 1110 2012*	Dothideo	Asco	80	64	57	65	266	GCA_000263175.2	UNIVERSITY OF MALAYA
121	*Fusarium oxysporum*	Sordario	Asco	71	93	46	55	265	GCA_001703215.1	University of Amsterdam
122	*Pyrenochaeta sp. DS3sAY3a*	Dothideo	Asco	80	81	43	61	265	GCA_001644535.1	DOE Joint Genome Institute
123	*Fusarium oxysporum*	Sordario	Asco	68	92	50	54	264	GCA_002234045.1	University of Amsterdam
124	*Calonectria crousiana*	Sordario	Asco	69	97	46	52	264	GCA_013406985.1	FABI
125	*Paraphoma sp. B47-9*	Dothideo	Asco	76	84	38	66	264	GCA_001748405.1	National Institute for Agro-Environmental Sciences (NIAES)
126	*Fusarium oxysporum*	Sordario	Asco	66	94	52	51	263	GCA_000260155.3	Broad Institute
127	*Stenocarpella maydis*	Sordario	Asco	80	71	50	62	263	GCA_002270565.1	University of Arkansas
128	*Fusarium metavorans*	Sordario	Asco	78	94	39	52	263	GCA_001633045.1	USDA-ARS
129	*Fusarium oxysporum*	Sordario	Asco	68	94	49	51	262	GCA_001702645.1	University of Amsterdam
130	*Fusarium sp. NRRL 62957*	Sordario	Asco	75	92	39	56	262	GCA_012978535.1	US Department of Agriculture, Agriculture Research Service
131	*Pseudogymnoascus sp.* VKM F-4518 *(FW-2643)*	Leotio	Asco	82	94	43	43	262	GCA_000750925.1	Moscow State University
132	*Ganoderma boninense*	Agarico	Basidio	77	36	44	104	261	GCA_002900995.2	PT SMART Tbk
133	*Periconia macrospinosa*	Dothideo	Asco	89	47	48	77	261	GCA_003073855.1	DOE Joint Genome Institute
134	*Fusarium phaseoli*	Sordario	Asco	75	94	42	50	261	GCA_013364925.1	US Department of Agriculture, Agriculture Research Service
135	*Hymenoscyphus salicellus*	Leotio	Asco	75	76	41	69	261	GCA_001414355.1	University of Exeter
136	*Fusarium oxysporum*	Sordario	Asco	68	93	48	51	260	GCA_002233895.1	University of Amsterdam
137	*Fusarium oxysporum*	Sordario	Asco	65	94	48	53	260	GCA_003977725.1	University of California at Davis
138	*Fusarium oxysporum*	Sordario	Asco	67	97	45	51	260	GCA_000260235.2	Broad Institute
139	*Fusarium albosuccineum*	Sordario	Asco	64	102	42	52	260	GCA_012931995.1	US Department of Agriculture, Agriculture Research Service
140	*Fusarium oxysporum*	Sordario	Asco	67	92	49	51	259	GCA_001702695.2	University of Amsterdam
141	*Stachybotrys chartarum*	Sordario	Asco	86	69	47	57	259	GCA_000732565.1	UT Southwestern

142	*Fusarium oxysporum*	Sordario	Asco	67	94	44	53	258	GCA_013423245.1	St. Petersburg University
143	*Fusarium neocosmosporiellum*	Sordario	Asco	76	88	42	52	258	GCA_006518225.1	US Department of Agriculture, Agriculture Research Service
144	*Colletotrichum lindemuthianum*	Sordario	Asco	68	96	38	56	258	GCA_001693025.2	Universidade Federal de Vicosa
145	*Fusarium oxysporum*	Sordario	Asco	67	91	49	50	257	GCA_002233805.1	University of Amsterdam
146	*Coprinellus micaceus*	Agarico	Basidio	74	26	68	89	257	GCA_004369175.1	DOE Joint Genome Institute
147	*Fusarium oxysporum*	Sordario	Asco	64	92	47	53	256	GCA_009602545.1	Auburn University
148	*Fusarium vanettenii*	Sordario	Asco	74	90	42	50	256	GCA_000151355.1	DOE Joint Genome Institute
149	*Fusarium oxysporum*	Sordario	Asco	67	92	46	50	255	GCA_002234115.1	University of Amsterdam
150	*Fusarium oxysporum*	Sordario	Asco	64	93	46	52	255	GCA_002233865.1	University of Amsterdam
151	*Aspergillus calidoustus*	Eurotio	Asco	74	95	40	46	255	GCA_001511075.1	HKI JENA
152	*Pezicula neosporulosa*	Leotio	Asco	73	91	27	64	255	GCA_009805495.1	Chinese Academy of Forestry
153	*Fusarium oxysporum*	Sordario	Asco	61	95	50	48	254	GCA_001888865.1	ZhongKai University of Agriculture and Engineering
154	*Lophiotrema nucula*	Dothideo	Asco	72	68	40	74	254	GCA_010015825.1	DOE Joint Genome Institute
155	*Colletotrichum shisoi*	Sordario	Asco	62	98	42	52	254	GCA_006783085.1	RIKEN
156	*Endocalyx cinctus*	0	Asco	81	60	48	64	253	GCA_001600455.1	RIKEN Center for Life Science Technologies, Division of Genomic Technologies
157	*Fusarium protoensiforme*	Sordario	Asco	78	89	38	48	253	GCA_011320165.1	US Department of Agriculture, Agriculture Research Service
158	*Stagonospora sp. SRC1lsM3a*	Dothideo	Asco	80	52	57	64	253	GCA_001644525.1	DOE Joint Genome Institute
159	*Hymenoscyphus herbarum*	Leotio	Asco	74	70	42	67	253	GCA_001414485.1	University of Exeter
160	*Coleophoma crateriformis*	Leotio	Asco	73	93	25	61	252	GCA_003369635.1	The University of Texas Health Science Center at Houston
161	*Pseudogymnoascus sp. VKM F-103*	Leotio	Asco	72	88	53	39	252	GCA_000750895.1	Moscow State University
162	*Fusarium oxysporum*	Sordario	Asco	64	90	48	49	251	GCA_013347345.1	Washington State University Mount Vernon Northwestern Research and Extension Center
163	*Gamarada debralockiae*	Leotio	Asco	65	79	43	63	250	GCA_002803225.1	Macquarie University
164	*Fusarium oxysporum*	Sordario	Asco	65	89	47	49	250	GCA_009755825.1	University of Massachusetts Amherst
165	*Fusarium oxysporum*	Sordario	Asco	63	93	46	48	250	GCA_002234135.1	University of Amsterdam
166	*Fusarium oxysporum*	Sordario	Asco	65	91	47	47	250	GCA_002234105.1	University of Amsterdam
167	*Byssothecium circinans*	Dothideo	Asco	73	74	44	59	250	GCA_010015675.1	DOE Joint Genome Institute
168	*Juglanconis sp. DMW523*	Sordario	Asco	76	82	31	60	249	GCA_003013055.1	USDA-ARS
169	*Pseudomassariella vexata*	Sordario	Asco	68	87	33	61	249	GCA_002105095.1	DOE Joint Genome Institute
170	*Melanomma pulvis-pyrius*	Dothideo	Asco	72	62	49	66	249	GCA_010093585.1	DOE Joint Genome Institute
171	*Aaosphaeria arxii*	Dothideo	Asco	82	65	44	58	249	GCA_010015735.1	DOE Joint Genome Institute
172	*Clavariopsis aquatica*	Sordario	Asco	79	61	42	67	249	GCA_013620735.1	Leibniz-Institute of Freshwater Ecology and Inland Fisheries
173	*Fusarium fracticaudum*	Sordario	Asco	59	87	56	46	248	GCA_003353625.1	Forestry and Agricultural Biotechnology Institute, University of Pretoria

174	*Fusarium tuaranense*	Sordario	Asco	72	88	40	48	248	GCA_013363205.1	US Department of Agriculture, Agriculture Research Service
175	*Fusarium sp. NRRL 62941*	Sordario	Asco	74	85	40	49	248	GCA_012978555.1	US Department of Agriculture, Agriculture Research Service
176	*Fusarium newnesense*	Sordario	Asco	59	83	51	54	247	GCA_013184375.1	US Department of Agriculture, Agriculture Research Service
177	*Arthrinium phaeospermum*	Sordario	Asco	78	54	52	63	247	GCA_006503535.1	Sichuan Agricultural University
178	*Fusarium sterilihyphosum*	Sordario	Asco	58	88	52	49	247	GCA_013186845.1	US Department of Agriculture, Agriculture Research Service
179	*Clohesyomyces aquaticus*	Dothideo	Asco	75	49	52	71	247	GCA_002105025.1	DOE Joint Genome Institute
180	*Hymenoscyphus fructigenus*	Leotio	Asco	70	69	42	66	247	GCA_001414455.1	University of Exeter
181	*Fusarium oxysporum*	Sordario	Asco	62	92	45	47	246	GCA_000350345.1	BGI
182	*Fusarium sarcochroum*	Sordario	Asco	63	96	41	46	246	GCA_013266185.1	US Department of Agriculture, Agriculture Research Service
183	*Neofusicoccum cordaticola*	Dothideo	Asco	64	84	30	68	246	GCA_009830905.1	University of Pretoria
184	*Pseudogymnoascus sp. 24MN13*	Leotio	Asco	72	89	49	36	246	GCA_001662595.1	US Forest Service
185	*Fusarium pseudocircinatum*	Sordario	Asco	60	87	48	50	245	GCA_013396035.1	US Department of Agriculture, Agriculture Research Service
186	*Fusarium sp. NRRL 62944*	Sordario	Asco	74	88	37	46	245	GCA_013186395.1	US Department of Agriculture, Agriculture Research Service
187	*Pseudogymnoascus verrucosus*	Leotio	Asco	68	89	51	37	245	GCA_001662655.1	US Forest Service
188	*Pseudogymnoascus sp. 04NY16*	Leotio	Asco	72	90	48	35	245	GCA_001630575.1	Ohio State University
189	*Aquanectria penicillioides*	Sordario	Asco	70	98	38	39	245	GCA_003415625.1	Nakdonggang National Institute of Biological Resources
190	*Trematosphaeria pertusa*	Dothideo	Asco	72	60	44	69	245	GCA_010094035.1	DOE Joint Genome Institute
191	*Fusarium oxysporum*	Sordario	Asco	64	91	39	50	244	GCA_014154955.1	University of Amsterdam
192	*Fusarium sp. NRRL 25303*	Sordario	Asco	60	86	49	49	244	GCA_013396255.1	US Department of Agriculture, Agriculture Research Service
193	*Fusarium oxysporum*	Sordario	Asco	63	86	46	49	244	GCA_001703455.1	University of Amsterdam
194	*Neofusicoccum kwambonambiense*	Dothideo	Asco	62	84	31	67	244	GCA_009829855.1	University of Pretoria
195	*Pseudogymnoascus sp. VKM F-3775*	Leotio	Asco	66	86	52	40	244	GCA_000750715.1	Moscow State University
196	*Verticillium nubilum*	Sordario	Asco	72	95	37	40	244	GCA_002851675.1	Wageningen University & Research
197	*Fusarium sp. NRRL 66088*	Sordario	Asco	77	86	36	44	243	GCA_013186415.1	US Department of Agriculture, Agriculture Research Service
198	*Fusarium sp. NRRL 25184*	Sordario	Asco	63	78	51	51	243	GCA_013755755.1	US Department of Agriculture, Agriculture Research Service
199	*Neofusicoccum parvum*	Dothideo	Asco	62	85	30	66	243	GCA_000385595.1	UC Davis
200	*Hymenopellis radicata*	Agarico	Basidio	58	55	34	96	243	GCA_003314005.1	Kunming University of Science and Technology
201	*Hymenotorrendiella dingleyae*	Leotio	Asco	69	68	40	66	243	GCA_003988905.1	Manaaki Whenua Landcare Research
202	*Fusarium ambrosium*	Sordario	Asco	74	82	37	49	242	GCA_003947045.1	University of California, Riverside

203	*Penicillium janthinellum*	Eurotio	Asco	59	104	49	30	242	GCA_002369805.1	CSIR-NIIST
204	*Neofusicoccum ribis*	Dothideo	Asco	63	81	30	68	242	GCA_009829435.1	University of Pretoria
205	*Neofusicoccum umdonicola*	Dothideo	Asco	63	82	30	67	242	GCA_009829365.1	University of Pretoria
206	*Lachnellula suecica*	Leotio	Asco	67	62	51	62	242	GCA_007825345.1	Canadian Food Inspection Agency (CFIA)
207	*Fusarium tupiense*	Sordario	Asco	56	85	51	49	241	GCA_013364945.1	US Department of Agriculture, Agriculture Research Service
208	*Arthrinium malaysianum*	Sordario	Asco	75	54	54	58	241	GCA_006508115.1	Indian Institute Of Chemical biology
209	*Juglanconis oblonga*	Sordario	Asco	74	79	32	56	241	GCA_003012965.1	USDA-ARS
210	*Colletotrichum orchidophilum*	Sordario	Asco	62	86	38	55	241	GCA_001831195.1	University of Western Brittany
211	*Pseudogymnoascus sp. BL308*	Leotio	Asco	71	91	45	34	241	GCA_001630595.1	Ohio State University
212	*Auricularia cornea*	Agarico	Basidio	65	42	53	80	240	GCA_008368385.1	Jilin Agricultural University
213	*Fusarium oxysporum*	Sordario	Asco	56	91	43	50	240	GCA_009746015.1	University of Massachusetts Amherts
214	*Botryosphaeria dothidea*	Dothideo	Asco	68	80	26	66	240	GCA_011503125.2	Shandong Agricultural University
215	*Fusarium odoratissimum*	Sordario	Asco	59	88	45	48	240	GCA_000260195.2	Broad Institute
216	*Colletotrichum tanaceti*	Sordario	Asco	60	92	38	50	240	GCA_005350895.1	The University of Melbourne
217	*Fusarium sp. NRRL 53293*	Sordario	Asco	55	83	54	47	239	GCA_013759125.1	US Department of Agriculture, Agriculture Research Service
218	*Juglanconis juglandina*	Sordario	Asco	72	80	33	54	239	GCA_003012975.1	USDA-ARS
219	*Fusarium begoniae*	Sordario	Asco	57	82	53	47	239	GCA_013186755.1	US Department of Agriculture, Agriculture Research Service
220	*Neonectria sp. DH2*	Sordario	Asco	72	90	38	39	239	GCA_003934905.1	Sun Yat-sen University
221	*Alternaria tenuissima*	Dothideo	Asco	70	68	42	59	239	GCA_004156035.1	NIAB-East Malling Research
222	*Fusarium foetens*	Sordario	Asco	64	82	43	49	238	GCA_013623845.1	US Department of Agriculture, Agriculture Research Service
223	*Fusarium euwallaceae*	Sordario	Asco	72	83	37	46	238	GCA_003957675.1	University of California, Riverside
224	*Colletotrichum lentis*	Sordario	Asco	60	91	37	50	238	GCA_003386485.1	University of Saskatchewan
225	*Alternaria burnsii*	Dothideo	Asco	67	69	43	59	238	GCA_013036055.1	Northwest A&F University
226	*Fusarium mexicanum*	Sordario	Asco	55	84	51	47	237	GCA_013396015.1	US Department of Agriculture, Agriculture Research Service
227	*Fusarium commune*	Sordario	Asco	59	86	45	46	236	GCA_001599515.1	RIKEN Center for Life Science Technologies, Division of Genomic Technologies
228	*Fusarium oxysporum*	Sordario	Asco	62	81	41	52	236	GCA_001757345.1	ICRISAT
229	*Colletotrichum sublineola*	Sordario	Asco	64	66	48	58	236	GCA_001951195.1	University of Kentucky
230	*Fusarium udum*	Sordario	Asco	56	86	44	50	236	GCA_013186905.1	US Department of Agriculture, Agriculture Research Service
231	*Alternaria arborescens*	Dothideo	Asco	69	68	40	59	236	GCA_004634205.1	USFDA
232	*Verticillium nonalfalfae*	Sordario	Asco	69	89	38	40	236	GCA_002776445.1	University of Ljubljana, Biotechnical faculty

233	*Fusarium guttiforme*	Sordario	Asco	57	81	49	48	235	GCA_013186795.1	US Department of Agriculture, Agriculture Research Service
234	*Fusarium nygamai*	Sordario	Asco	59	77	48	51	235	GCA_001262555.1	University of Pretoria
235	*Lentithecium fluviatile*	Dothideo	Asco	71	53	38	73	235	GCA_010405425.1	DOE Joint Genome Institute
236	*Alternaria alternata*	Dothideo	Asco	66	68	41	60	235	GCA_011420255.1	National Institute of Biological Resources
237	*Alternaria sp. MG1*	Dothideo	Asco	68	68	41	58	235	GCA_003574525.1	Northwestern Polytechnical University
238	*Fusarium subglutinans*	Sordario	Asco	58	77	49	50	234	GCA_012071885.1	UNRC
239	*Fusarium anthophilum*	Sordario	Asco	57	80	49	48	234	GCA_013364935.1	US Department of Agriculture, Agriculture Research Service
240	*Fusarium pininemorale*	Sordario	Asco	56	81	51	46	234	GCA_002165215.1	Forestry and Agricultural Biotechnology Institute
241	*Fusarium mangiferae*	Sordario	Asco	57	85	47	45	234	GCA_900044065.1	Institute of Bioinformatics and Systems Biology
242	*Clarireedia monteithiana*	Leotio	Asco	63	75	36	60	234	GCA_002242835.1	Rutgers University
243	*Fusarium sp. AF-4*	Sordario	Asco	73	80	36	45	234	GCA_003946995.1	University of California, Riverside
244	*Verticillium alfalfae*	Sordario	Asco	70	86	38	40	234	GCA_002851655.1	Wageningen University & Research
245	*Rhytidhysteron rufulum*	Dothideo	Asco	63	55	57	59	234	GCA_000467735.1	Assembling the Fungal Tree of Life (AFTOL)
246	*Trametes villosa*	Agarico	Basidio	66	38	35	94	233	GCA_002964805.1	Universidade Federal de Minas Gerais
247	*Phoma sp. XZ068*	Dothideo	Asco	59	88	31	55	233	GCA_004835665.1	Institute Of Microbiology Chinese Academy of Sciences
248	*Talaromyces purpureogenus*	Eurotio	Asco	56	94	50	33	233	GCA_001270325.1	Mathomics
249	*Alternaria atra*	Dothideo	Asco	67	66	44	56	233	GCA_004634305.1	USFDA
250	*Cladosporium sp. SL-16*	Dothideo	Asco	68	71	42	52	233	GCA_002921095.1	Chinese academy of agricultural sciences
251	*Cylindrocarpon cylindroides*	Sordario	Asco	68	93	34	37	232	GCA_013756995.1	US Department of Agriculture, Agriculture Research Service
252	*Fusarium floridanum*	Sordario	Asco	72	76	38	46	232	GCA_003947005.1	University of California, Riverside
253	*Macrophomina phaseolina*	Dothideo	Asco	60	80	26	66	232	GCA_008729105.1	USDA
254	*Karstenula rhodostoma*	Dothideo	Asco	64	72	40	56	232	GCA_010093485.1	DOE Joint Genome Institute
255	*Pseudogymnoascus pannorum*	Leotio	Asco	63	82	50	37	232	GCA_001630605.1	Ohio State University
256	*Lophiostoma macrostomum*	Dothideo	Asco	72	59	37	64	232	GCA_010405375.1	DOE Joint Genome Institute
257	*Phaeosphaeria sp. A1 3.1a*	Dothideo	Asco	73	51	51	57	232	GCA_003501895.1	Centre for Crop & Disease Management, Curtin University
258	*Auricularia subglabra*	Agarico	Basidio	64	38	54	75	231	GCA_000265015.1	JGI
259	*Fusarium acuminatum*	Sordario	Asco	66	76	41	48	231	GCA_013363215.1	US Department of Agriculture, Agriculture Research Service

260	*Fusarium succisae*	Sordario	Asco	56	81	49	45	231	GCA_013186925.1	US Department of Agriculture, Agriculture Research Service
261	*Phoma herbarum*	Dothideo	Asco	65	79	32	55	231	GCA_001599375.1	RIKEN Center for Life Science Technologies, Division of Genomic Technologies
262	*Alternaria consortialis*	Dothideo	Asco	66	68	42	55	231	GCA_001950455.1	RIKEN Center for Life Science Technologies, Division of Genomic Technologies
263	*Xylaria sp. MSU_SB201401*	Sordario	Asco	66	67	44	54	231	GCA_002288965.1	University of Arkansas
264	*Preussia sp. BSL10*	Dothideo	Asco	66	74	40	51	231	GCA_001553865.1	Macrogen, Korea
265	*Fusarium fractiflexum*	Sordario	Asco	61	76	43	50	230	GCA_013758875.1	US Department of Agriculture, Agriculture Research Service
266	*Fusarium azukicola*	Sordario	Asco	66	80	34	50	230	GCA_001680625.1	Iowa State University
267	*Phaeosphaeria sp. H6.2b*	Dothideo	Asco	72	51	51	56	230	GCA_003503105.1	Centre for Crop & Disease Management, Curtin University
268	*Amylostereum areolatum*	Agarico	Basidio	70	40	26	94	230	GCA_012932865.1	Beijing Forestry University
269	*Stachybotrys chlorohalonata*	Sordario	Asco	79	58	42	51	230	GCA_000732775.1	UT Southwestern
270	*Fibularhizoctonia sp. CBS 109695*	Agarico	Basidio	70	40	31	88	229	GCA_001630335.1	DOE Joint Genome Institute
271	*Lindgomyces ingoldianus*	Dothideo	Asco	74	42	44	69	229	GCA_010093535.1	DOE Joint Genome Institute
272	*Rachicladosporium antarcticum*	Dothideo	Asco	68	32	72	56	228	GCA_002077065.1	University of California, Riverside
273	*Helotiales sp. F229*	Leotio	Asco	66	64	42	56	228	GCA_002554605.1	CEPLAS/University of Cologne
274	*Fusarium tjaetaba*	Sordario	Asco	56	79	46	47	228	GCA_013396195.1	US Department of Agriculture, Agriculture Research Service
275	*Fusarium kuroshium*	Sordario	Asco	70	79	35	44	228	GCA_003698175.1	University of California, Riverside
276	*Didymella heteroderae*	Dothideo	Asco	63	79	33	53	228	GCA_011058895.1	University of Campinas
277	*Ophiobolus disseminans*	Dothideo	Asco	67	61	40	60	228	GCA_010093685.1	DOE Joint Genome Institute
278	*Fusarium ramigenum*	Sordario	Asco	56	79	42	50	227	GCA_013186855.1	US Department of Agriculture, Agriculture Research Service
279	*Fusarium sp. Na10*	Sordario	Asco	53	80	48	46	227	GCA_002234255.1	University of Amsterdam
280	*Fusarium globosum*	Sordario	Asco	56	77	45	49	227	GCA_013396165.1	US Department of Agriculture, Agriculture Research Service
281	*Lasiodiplodia sp. COLG20*	Dothideo	Asco	59	83	26	59	227	GCA_009801085.1	University of Agriculture, Faisalabad Pakistan.
282	*Penicillium subrubescens*	Eurotio	Asco	56	86	51	33	226	GCA_001908125.1	CBS-KNAW Fungal Biodiversity Centre Institute of the Royal Netherlands Academy of Arts and Science
283	*Fusarium proliferatum*	Sordario	Asco	58	75	43	50	226	GCA_003290285.1	University of Pisa
284	*Bimuria novae-zelandiae*	Dothideo	Asco	64	69	39	54	226	GCA_010015655.1	DOE Joint Genome Institute
285	*Fusarium fujikuroi*	Sordario	Asco	56	78	43	49	226	GCA_900096635.1	Technische Universitat Munchen - WZW
286	*Colletotrichum graminicola*	Sordario	Asco	62	60	47	57	226	GCA_000149035.1	Broad Institute

287	*Fusarium setosum*	Sordario	Asco	65	83	38	40	226	GCA_013623625.1	US Department of Agriculture, Agriculture Research Service
288	*Didymella segeticola*	Dothideo	Asco	58	91	29	48	226	GCA_004522025.1	Guizhou University
289	*Fusarium phyllophilum*	Sordario	Asco	54	80	44	47	225	GCA_013396025.1	US Department of Agriculture, Agriculture Research Service
290	*Fusarium xylarioides*	Sordario	Asco	54	83	42	46	225	GCA_013183765.1	University of Pretoria
291	*Aspergillus sergii*	Eurotio	Asco	53	100	28	44	225	GCA_009193525.1	DOE Joint Genome Institute
292	*Lasiodiplodia theobromae*	Dothideo	Asco	58	80	28	59	225	GCA_012971845.1	Beltsville Agricultural Research Center
293	*Lasiodiplodia pseudotheobromae*	Dothideo	Asco	58	82	27	58	225	GCA_009829805.1	University of Pretoria
294	*Ganoderma sp. BRIUMSc*	Agarico	Basidio	62	31	34	98	225	GCA_008694245.1	Universiti Malaysia Sabah
295	*Fusarium denticulatum*	Sordario	Asco	56	82	39	47	224	GCA_013396175.1	US Department of Agriculture, Agriculture Research Service
296	*Monosporascus sp. MG133*	Sordario	Asco	69	60	39	56	224	GCA_004155925.1	University of New Mexico
297	*Fusarium sacchari*	Sordario	Asco	53	78	46	47	224	GCA_013759005.1	US Department of Agriculture, Agriculture Research Service
298	*Fusarium temperatum*	Sordario	Asco	55	77	47	45	224	GCA_001513835.1	Forestry and Agricultural Biotechnology Institute (FABI)
299	*Fusarium pseudoanthophilum*	Sordario	Asco	57	76	44	47	224	GCA_013395995.1	US Department of Agriculture, Agriculture Research Service
300	*Pseudocercospora fijiensis*	Dothideo	Asco	58	81	26	59	224	GCA_011057605.1	Instituto de Investigacion Lightbourn
301	*Aspergillus ustus*	Eurotio	Asco	62	78	42	42	224	GCA_000812125.1	Institute of developmentive and regenerative biology
302	*Fusarium sp. AF-8*	Sordario	Asco	70	78	34	42	224	GCA_003946985.1	University of California, Riverside
303	*Monosporascus sp. mg162*	Sordario	Asco	65	66	40	53	224	GCA_004155945.1	University of New Mexico
304	*Fusarium avenaceum*	Sordario	Asco	62	74	40	47	223	GCA_000769295.1	Collaboration between Erik Lysøe (Department of Plant Health and Plant Protection, Bioforsk - Norwegian Institute of Agricultural and Environmental Research) and Linda Harris (Eastern Cereal & Oilseed Research Centre, Agriculture & Agri-Food Canada).
305	*Fusarium mundagurra*	Sordario	Asco	58	70	46	49	223	GCA_013396205.1	US Department of Agriculture, Agriculture Research Service
306	*Pseudogymnoascus sp. VKM F-4517 (FW-2822)*	Leotio	Asco	62	76	48	37	223	GCA_000750875.1	Moscow State University
307	*Aspergillus sojae*	Eurotio	Asco	52	100	28	43	223	GCA_009687765.1	Tokyo Institute of Technology
308	*Pseudogymnoascus sp. VKM F-4246*	Leotio	Asco	66	79	43	35	223	GCA_000750735.1	Moscow State University
309	*Neoscytalidium dimidiatum*	Dothideo	Asco	61	78	24	59	222	GCA_900092665.1	University Malaya

310	*Fusarium pseudonygamai*	Sordario	Asco	57	76	43	45	221	GCA_013186785.1	US Department of Agriculture, Agriculture Research Service
311	*Botryosphaeria kuwatsukai*	Dothideo	Asco	63	72	26	60	221	GCA_004016305.1	Northwest A&F University
312	*Rutstroemia sydowiana*	Leotio	Asco	62	69	24	66	221	GCA_000812895.1	USDA-ARS
313	*Pseudogymnoascus sp. VKM F-4514 (FW-929)*	Leotio	Asco	60	71	51	39	221	GCA_000750795.1	Moscow State University
314	*Fusarium bulbicola*	Sordario	Asco	54	76	45	45	220	GCA_013758895.1	US Department of Agriculture, Agriculture Research Service
315	*Fusarium concolor*	Sordario	Asco	54	71	47	48	220	GCA_013184415.1	US Department of Agriculture, Agriculture Research Service
316	*Polyplosphaeria fusca*	Dothideo	Asco	65	48	44	63	220	GCA_010093805.1	DOE Joint Genome Institute
317	*Rutstroemia sp. NJR-2017a BVV2*	Leotio	Asco	56	73	39	52	220	GCA_002946415.1	Brigham Young University
318	*Didymella pinodes*	Dothideo	Asco	55	85	32	48	220	GCA_004151525.1	Curtin University
319	*Pseudogymnoascus sp. VKM F-3808*	Leotio	Asco	63	72	50	35	220	GCA_000750675.1	Moscow State University
320	*Amniculicola lignicola*	Dothideo	Asco	64	53	42	61	220	GCA_010015725.1	DOE Joint Genome Institute
321	*Monosporascus sp. 5C6A*	Sordario	Asco	68	56	37	58	219	GCA_004155035.1	University of New Mexico
322	*Fusarium coicis*	Sordario	Asco	54	74	45	46	219	GCA_013781345.1	US Department of Agriculture, Agriculture Research Service
323	*Fusarium sp. NRRL 47473*	Sordario	Asco	51	76	50	42	219	GCA_013759115.1	US Department of Agriculture, Agriculture Research Service
324	*Colletotrichum falcatum*	Sordario	Asco	59	57	46	57	219	GCA_001484525.1	ICAR-Sugarcane Breeding Institute
325	*Pyrenochaeta sp. UM 256*	Dothideo	Asco	62	64	40	53	219	GCA_000359685.2	UNIVERSITY OF MALAYA
326	*Pseudogymnoascus sp. 05NY08*	Leotio	Asco	60	85	44	30	219	GCA_001662605.1	US Forest Service
327	*Verticillium dahliae*	Sordario	Asco	62	80	39	38	219	GCA_004798895.1	Wageningen University and Research
328	*Monosporascus sp. CRB-9-2*	Sordario	Asco	63	64	39	52	218	GCA_004155915.1	University of New Mexico
329	*Fusarium hostae*	Sordario	Asco	62	73	37	46	218	GCA_002234235.1	University of Amsterdam
330	*Fusarium brevicatenulatum*	Sordario	Asco	57	71	44	46	218	GCA_013363135.1	US Department of Agriculture, Agriculture Research Service
331	*Didymella lethalis*	Dothideo	Asco	54	88	30	46	218	GCA_004335245.1	Curtin University
332	*Ascochyta lentis*	Dothideo	Asco	56	84	26	52	218	GCA_004011705.1	Curtin University
333	*Pseudogymnoascus sp. VKM F-4516 (FW-969)*	Leotio	Asco	61	70	48	39	218	GCA_000750815.1	Moscow State University
334	*Ascochyta viciae*	Dothideo	Asco	56	83	28	51	218	GCA_004335155.1	Curtin University
335	*Ascochyta fabae*	Dothideo	Asco	57	82	28	51	218	GCA_004335285.1	Curtin University
336	*Aspergillus sp. ATCC 12892*	Eurotio	Asco	51	95	29	42	217	GCA_002894705.1	Pacific Northwest National Lab
337	*Hyaloscypha bicolor*	Leotio	Asco	52	64	40	61	217	GCA_002865645.1	DOE Joint Genome Institute
338	*Lasiodiplodia gonubiensis*	Dothideo	Asco	55	81	27	54	217	GCA_009829795.1	University of Pretoria
339	*Fusarium dlaminii*	Sordario	Asco	54	72	46	45	217	GCA_013186775.1	US Department of Agriculture, Agriculture Research Service
340	*Neonectria galligena*	Sordario	Asco	59	90	34	34	217	GCA_013759035.1	US Department of Agriculture, Agriculture Research Service
341	*Xylaria grammica*	Sordario	Asco	66	56	43	52	217	GCA_004353285.1	National Institute of Biological Resources
342	*Phoma sp. RAV-16-625*	Dothideo	Asco	57	85	30	45	217	GCA_004335185.1	Curtin University

343	*Pseudogymnoascus sp. VKM F-3557*	Leotio	Asco	60	67	50	40	217	GCA_000750665.1	Moscow State University
344	*Aspergillus novoparasiticus*	Eurotio	Asco	55	84	30	47	216	GCA_009176405.1	DOE Joint Genome Institute
345	*Fusarium verticillioides*	Sordario	Asco	54	71	45	46	216	GCA_003317015.2	CSIRO
346	*Setomelanomma holmii*	Dothideo	Asco	58	66	36	56	216	GCA_010015745.1	DOE Joint Genome Institute
347	*Ascochyta viciae-villosae*	Dothideo	Asco	56	83	26	51	216	GCA_004335205.1	Curtin University
348	*Hymenoscyphus repandus*	Leotio	Asco	66	60	34	56	216	GCA_001414415.1	University of Exeter
349	*Fusarium sp. QHM*	Sordario	Asco	54	69	46	46	215	GCA_013416755.1	Liaoning Technical University
350	*Fusarium sp. BWC*	Sordario	Asco	54	69	46	46	215	GCA_013416785.1	Liaoning Technical University
351	*Aspergillus pseudocaelatus*	Eurotio	Asco	57	87	31	40	215	GCA_009193665.1	DOE Joint Genome Institute
352	*Aspergillus amoenus*	Eurotio	Asco	57	79	37	42	215	GCA_009812435.1	Universidad de Antioquia
353	*Aspergillus versicolor*	Eurotio	Asco	54	85	40	36	215	GCA_001890125.1	DOE Joint Genome Institute
354	*Pseudogymnoascus sp. VKM F-4515 (FW-2607)*	Leotio	Asco	60	75	45	35	215	GCA_000750805.1	Moscow State University
355	*Didymella keratinophila*	Dothideo	Asco	58	76	32	49	215	GCA_011058865.1	University of Campinas
356	*fungal sp. EF0021*	0	0	63	58	38	56	215	GCA_000292665.1	Fraunhofer
357	*Hymenoscyphus scutula*	Leotio	Asco	62	61	38	54	215	GCA_001399465.1	University of Exeter
358	*Fusarium secorum*	Sordario	Asco	48	81	40	45	214	GCA_013363185.1	US Department of Agriculture, Agriculture Research Service
359	*Fusarium brasiliense*	Sordario	Asco	59	78	34	43	214	GCA_001680685.1	Iowa State University
360	*Fusarium napiforme*	Sordario	Asco	55	72	42	45	214	GCA_013396005.1	US Department of Agriculture, Agriculture Research Service
361	*Monosporascus sp. CRB-8-3*	Sordario	Asco	65	62	36	51	214	GCA_004155055.1	University of New Mexico
362	*Stemphylium vesicarium*	Dothideo	Asco	61	62	36	55	214	GCA_004380135.1	University of Bologna
363	*Chaetomium cochliodes*	Sordario	Asco	67	42	59	46	214	GCA_001752565.1	BOKU University
364	*Xylaria flabelliformis*	Sordario	Asco	61	60	40	53	214	GCA_007182795.1	Vanderbilt University
365	*Alternaria solani*	Dothideo	Asco	60	62	38	54	214	GCA_002837235.1	Institute Of Microbiology Chinese Academy of Sciences
366	*Madurella mycetomatis*	Sordario	Asco	69	32	61	52	214	GCA_001275765.2	ErasmusMC
367	*Lomentospora prolificans*	Sordario	Asco	74	39	52	49	214	GCA_002276285.1	Johns Hopkins University
368	*Fusarium musae*	Sordario	Asco	53	70	45	45	213	GCA_013623345.1	US Department of Agriculture, Agriculture Research Service
369	*Aspergillus caelatus*	Eurotio	Asco	57	85	31	40	213	GCA_009193585.1	DOE Joint Genome Institute
370	*Pseudogymnoascus sp. WSF 3629*	Leotio	Asco	64	79	36	34	213	GCA_001662585.1	US Forest Service
371	*Massarina eburnea*	Dothideo	Asco	57	65	42	49	213	GCA_010093635.1	DOE Joint Genome Institute
372	*Calonectria henricotiae*	Sordario	Asco	54	76	38	44	212	GCA_004380885.1	USDA-ARS
373	*Fusarium algeriense*	Sordario	Asco	54	67	41	50	212	GCA_002982035.1	US Department of Agriculture, Agriculture Research Service
374	*Fusarium bactridioides*	Sordario	Asco	53	71	44	44	212	GCA_013623355.1	US Department of Agriculture, Agriculture Research Service
375	*Monosporascus ibericus*	Sordario	Asco	62	60	38	52	212	GCA_004154915.1	University of New Mexico
376	*Aspergillus sp. MA 6037*	Eurotio	Asco	54	83	35	40	212	GCA_003138035.1	University of Natural Resources and Life Sciences, Vienna
377	*Sclerotiophoma versabilis*	Dothideo	Asco	54	80	30	48	212	GCA_012274445.1	Fujian Agriculture and Forestry University

378	*Rutstroemia sp. NJR-2017a WRK4*	Leotio	Asco	56	66	36	54	212	GCA_002946385.1	Brigham Young University
379	*Glarea lozoyensis*	Leotio	Asco	67	49	45	51	212	GCA_000409485.1	Institute Of Microbiology, Chinese Academy of Sciences
380	*Fusarium beomiforme*	Sordario	Asco	54	64	41	52	211	GCA_002980475.2	US Department of Agriculture, Agriculture Research Service
381	*Neonothopanus nambi*	Agarico	Basidio	58	18	33	102	211	GCA_003987895.1	Universidade de Sao Paulo
382	*Epicoccum sorghinum*	Dothideo	Asco	59	64	36	52	211	GCA_001879705.1	University of Sao Paulo
383	*Fusarium solani*	Sordario	Asco	58	80	31	42	211	GCA_002215905.1	National Institute of Biological Resources
384	*Hymenoscyphus infarciens*	Leotio	Asco	60	65	36	50	211	GCA_001414345.1	University of Exeter
385	*Aspergillus sydowii*	Eurotio	Asco	52	78	36	44	210	GCA_009193685.1	Hamburg University
386	*Neonectria ditissima*	Sordario	Asco	58	84	34	34	210	GCA_001305495.1	The New Zealand Institute for Plant & Food Research Ltd
387	*Xylaria longipes*	Sordario	Asco	65	52	38	55	210	GCA_003426265.1	IHI Zittau / TU Dresden
388	*Curvularia kusanoi*	Dothideo	Asco	58	64	36	52	210	GCA_011058905.1	University of Campinas
389	*Pseudogymnoascus sp. VKM F-4513 (FW-928)*	Leotio	Asco	58	72	46	34	210	GCA_000750755.1	Moscow State University
390	*Sphaerobolus stellatus*	Agarico	Basidio	59	18	46	87	210	GCA_000827215.1	DOE Joint Genome Institute
391	*Zasmidium citrigriseum*	Dothideo	Asco	52	62	34	61	209	GCA_002786025.1	Northwest A&F University
392	*Aspergillus pseudotamarii*	Eurotio	Asco	55	88	28	38	209	GCA_009193445.1	DOE Joint Genome Institute
393	*Fusarium sp. NRRL 29148*	Sordario	Asco	54	68	41	46	209	GCA_013759095.1	US Department of Agriculture, Agriculture Research Service
394	*Aspergillus transmontanensis*	Eurotio	Asco	52	86	28	43	209	GCA_009193505.1	DOE Joint Genome Institute
395	*Hypomontagnella monticulosa*	Sordario	Asco	68	42	45	54	209	GCA_902825475.1	CEBITEC
396	*Xylaria sp. BCC 1067*	Sordario	Asco	64	54	40	51	209	GCA_005188305.1	King Mongkut's University of Technology Thonburi
397	*Rachicladosporium sp. CCFEE 5018*	Dothideo	Asco	64	32	64	48	208	GCA_002077045.2	University of California, Riverside
398	*Fusarium burgessii*	Sordario	Asco	51	61	40	56	208	GCA_002980515.1	US Department of Agriculture, Agriculture Research Service
399	*Talaromyces adpressus*	Eurotio	Asco	51	72	49	36	208	GCA_002775195.1	University of Naples "Federico II"
400	*Fusarium tricinctum*	Sordario	Asco	54	68	41	45	208	GCA_900382705.2	INRA (Nouvelle-Aquitaine-Bordeaux)
401	*Aspergillus arachidicola*	Eurotio	Asco	52	84	28	44	208	GCA_009193545.1	DOE Joint Genome Institute
402	*Aspergillus parasiticus*	Eurotio	Asco	51	86	28	43	208	GCA_013146005.1	USDA
403	*Fusarium sp. JS626*	Sordario	Asco	54	72	40	42	208	GCA_000966865.1	Seoul National University
404	*Fusarium circinatum*	Sordario	Asco	54	71	38	45	208	GCA_000497325.3	Pitch canker sequencing project
405	*Xylaria polymorpha*	Sordario	Asco	60	56	42	50	208	GCA_003426235.1	IHI Zittau / TU Dresden
406	*Fusarium sambucinum*	Sordario	Asco	54	76	36	42	208	GCA_001567575.1	Central Potato Research Institute
407	*Didymobotryum rigidum*	Sordario	Asco	64	49	38	57	208	GCA_001600575.1	RIKEN Center for Life Science Technologies, Division of Genomic Technologies
408	*Xylaria multiplex*	Sordario	Asco	63	54	42	49	208	GCA_011057905.1	TU Dresden - IHI Zittau

409	*Aspergillus sp. Z5*	Eurotio	Asco	52	76	35	44	207	GCA_001044295.1	Zhejiang University
410	*Fusarium citri*	Sordario	Asco	54	73	42	38	207	GCA_004367485.1	US Department of Agriculture, Agriculture Research Service
411	*Fusarium sp. AF-6*	Sordario	Asco	62	72	31	42	207	GCA_003947015.1	University of California, Riverside
412	*Rosellinia necatrix*	Sordario	Asco	60	60	44	43	207	GCA_001445595.3	NARO Institute of Fruit Tree Science
413	*Chrysoporthe deuterocubensis*	Sordario	Asco	56	70	32	49	207	GCA_001513825.1	Forestry and Agricultural Biotechnology Institute (FABI), University of Pretoria
414	*Scedosporium apiospermum*	Sordario	Asco	73	22	56	56	207	GCA_000732125.1	LUNAM - Angers University
415	*Arachnopeziza araneosa*	Leotio	Asco	66	40	49	51	206	GCA_003988855.1	Manaaki Whenua Landcare Research
416	*Aspergillus steynii*	Eurotio	Asco	58	84	32	32	206	GCA_002849105.1	DOE Joint Genome Institute
417	*Psilocybe cf. subviscida*	Agarico	Basidio	58	33	51	64	206	GCA_013368295.1	Lund University
418	*Panaeolus cyanescens*	Agarico	Basidio	56	34	50	66	206	GCA_002938355.1	Ohio State University
419	*Monosporascus sp. GIB2*	Sordario	Asco	60	56	40	49	205	GCA_004155935.1	University of New Mexico
420	*Aspergillus minisclerotigenes*	Eurotio	Asco	48	90	28	39	205	GCA_009176455.1	DOE Joint Genome Institute
421	*Fusarium agapanthi*	Sordario	Asco	51	72	39	43	205	GCA_001654545.1	US Department of Agriculture, Agriculture Research Service
422	*Hymenoscyphus laetus*	Leotio	Asco	66	42	44	53	205	GCA_001414375.1	University of Exeter
423	*Diplocarpon rosae*	Leotio	Asco	48	82	20	54	204	GCA_002317995.1	Leibniz Universitaet Hannover
424	*Aspergillus oryzae*	Eurotio	Asco	50	88	32	34	204	GCA_002007945.1	Bioprocess Technology Laboratory, BIOTEC, Thailand
425	*Parastagonospora nodorum*	Dothideo	Asco	65	43	45	51	204	GCA_002267045.1	North Dakota State University
426	*Chrysoporthe austroafricana*	Sordario	Asco	55	70	32	47	204	GCA_001051155.1	Forestry and Agricultural Biotechnology Institute (FABI), University of Pretoria
427	*Talaromyces verruculosus*	Eurotio	Asco	48	67	48	40	203	GCA_001305275.1	Penicillium verruculosum
428	*Aspergillus tamarii*	Eurotio	Asco	56	86	26	35	203	GCA_009193485.1	DOE Joint Genome Institute
429	*Fusarium sp. NRRL 52700*	Sordario	Asco	50	65	44	44	203	GCA_013396095.1	US Department of Agriculture, Agriculture Research Service
430	*Fusarium thapsinum*	Sordario	Asco	51	67	40	45	203	GCA_013186935.1	US Department of Agriculture, Agriculture Research Service
431	*Bipolaris maydis*	Dothideo	Asco	65	48	35	55	203	GCA_000338975.1	JGI
432	*Coniothyrium glycines*	Dothideo	Asco	56	64	36	47	203	GCA_004523985.2	Oklahoma State University
433	*Sarocladium strictum*	Sordario	Asco	67	42	54	40	203	GCA_900290465.1	CEBITEC
434	*Helminthosporium solani*	Dothideo	Asco	58	58	39	48	203	GCA_000498615.1	University of Wisconsin-Madison
435	*Proliferodiscus dingleyae*	Leotio	Asco	53	58	46	46	203	GCA_003988795.1	Manaaki Whenua Landcare Research
436	*Pseudogymnoascus sp. BL549*	Leotio	Asco	58	75	41	29	203	GCA_001630585.1	Ohio State University
437	*Scytalidium sp. 3C*	Leotio	Asco	46	62	46	48	202	GCA_000743665.3	Saint-Petersburg State University

438	*Talaromyces cellulolyticus*	Eurotio	Asco	48	67	45	42	202	GCA_009805475.1	JNU
439	*Pseudohalonectria lignicola*	Sordario	Asco	60	47	46	49	202	GCA_003049395.1	Agricultural Research Service, United States Department of Agriculture
440	*Penicillium sp. MT2 MMC-2018*	Eurotio	Asco	51	77	46	28	202	GCA_003852855.1	Florida A&M University
441	*Fusarium scirpi*	Sordario	Asco	51	71	40	40	202	GCA_004367495.1	US Department of Agriculture, Agriculture Research Service
442	*Plenodomus tracheiphilus*	Dothideo	Asco	56	64	31	51	202	GCA_010093695.1	DOE Joint Genome Institute
443	*Rutstroemia sp. NJR-2017a BBW*	Leotio	Asco	53	65	33	51	202	GCA_002946425.1	Brigham Young University
444	*Ascochyta koolunga*	Dothideo	Asco	52	79	27	44	202	GCA_004151165.1	Curtin University
445	*Coniochaeta ligniaria*	Sordario	Asco	61	46	50	44	201	GCA_001879275.1	DOE Joint Genome Institute
446	*Fusarium venenatum*	Sordario	Asco	54	71	36	40	201	GCA_900007375.1	ROTHAMSTED RESEARCH
447	*Aspergillus hancockii*	Eurotio	Asco	54	78	29	40	201	GCA_001696595.1	CSIRO
448	*Ganoderma multipileum*	Agarico	Basidio	54	40	33	74	201	GCA_000338015.1	Ganoderma lucidum Research Consortium
449	*Cladosporium cladosporioides*	Dothideo	Asco	55	57	40	49	201	GCA_002901145.1	Yeungnam University
450	*Hymenoscyphus fraxineus*	Leotio	Asco	59	54	32	56	201	GCA_001414365.1	University of Exeter
451	*Chaetomium globosum*	Sordario	Asco	62	43	54	42	201	GCA_000143365.1	The Genome Sequencing Platform, The Genome Assembly Team
452	*Penicillium sp. 'occitanis'*	Eurotio	Asco	48	67	45	40	200	GCA_002382835.1	Center for Genomic Regulation (CRG)
453	*Talaromyces pinophilus*	Eurotio	Asco	50	66	46	38	200	GCA_011392495.1	Embrapa Agroenergia
454	*Fusarium tanahbumbuense*	Sordario	Asco	52	65	43	40	200	GCA_012977735.1	US Department of Agriculture, Agriculture Research Service
455	*Macroventuria anomochaeta*	Dothideo	Asco	48	82	27	43	200	GCA_010093625.1	DOE Joint Genome Institute
456	*Aspergillus westerdijkiae*	Eurotio	Asco	52	84	30	34	200	GCA_001307345.1	Nanyang Technological University
457	*Fusarium nisikadoi*	Sordario	Asco	50	63	41	45	199	GCA_013623555.1	US Department of Agriculture, Agriculture Research Service
458	*Fusarium austroafricanum*	Sordario	Asco	52	61	45	41	199	GCA_012932025.1	US Department of Agriculture, Agriculture Research Service
459	*Fusarium humuli*	Sordario	Asco	50	66	43	40	199	GCA_004366955.1	US Department of Agriculture, Agriculture Research Service
460	*Fusarium albidum*	Sordario	Asco	53	77	32	37	199	GCA_013618265.1	US Department of Agriculture, Agriculture Research Service
461	*Neonectria punicea*	Sordario	Asco	54	78	32	35	199	GCA_003385315.1	USDA-ARS
462	*fungal sp. No.14919*	0	0	59	50	40	50	199	GCA_002003505.1	Tsukuba Biotechnology Research Center, Astellas Pharma Inc.
463	*Curvularia geniculata*	Dothideo	Asco	62	52	36	49	199	GCA_002982235.1	Universidade Federal de Mato Grosso
464	*Curvularia lunata*	Dothideo	Asco	62	52	36	49	199	GCA_005212705.1	Universidade Federal de Mato Grosso

465	*Hymenoscyphus occultus*	Leotio	Asco	58	56	32	53	199	GCA_900536445.1	UNIVERSITY OF COPENHAGEN, DEPARTMENT OF GEOSCIENCES AND NATURAL RESOURCE MANAGEMENT
466	*Hymenoscyphus koreanus*	Leotio	Asco	58	56	32	53	199	GCA_902652825.1	UNIVERSITY OF COPENHAGEN, DEPARTMENT OF GEOSCIENCES AND NATURAL RESOURCE MANAGEMENT
467	*Fusarium flagelliforme*	Sordario	Asco	52	68	38	40	198	GCA_004367175.1	US Department of Agriculture, Agriculture Research Service
468	*Aspergillus aff. floccosus IMV 01167*	Eurotio	Asco	51	70	39	38	198	GCA_001931935.1	Jet Propulsion Laboratory, California Institute of Technology
469	*Chrysoporthe cubensis*	Sordario	Asco	54	64	30	50	198	GCA_004802525.1	USDA-ARS
470	*Neonectria coccinea*	Sordario	Asco	53	78	32	35	198	GCA_013757005.1	US Department of Agriculture, Agriculture Research Service
471	*Baeospora myosura*	Agarico	Basidio	58	38	23	79	198	GCA_001179705.1	Royal Botanic Gardens, Kew
472	*Limonomyces culmigenus*	Agarico	Basidio	58	42	45	52	197	GCA_002233555.1	USDA-ARS
473	*Ganoderma tsugae*	Agarico	Basidio	52	30	35	80	197	GCA_003057275.1	Biotechnology and Nuclear Technology Research Institute
474	*Fusarium babinda*	Sordario	Asco	52	63	38	44	197	GCA_012977765.1	US Department of Agriculture, Agriculture Research Service
475	*Aspergillus flavus*	Eurotio	Asco	47	86	31	33	197	GCA_004150275.1	The Energy and Resources Institute
476	*Stemphylium lycopersici*	Dothideo	Asco	52	61	36	48	197	GCA_003268315.1	Centro de Investigaciones en Fitopatologias (CIDEFI)
477	*Pleomassaria siparia*	Dothideo	Asco	56	60	34	47	197	GCA_010093715.1	DOE Joint Genome Institute
478	*Ceratobasidium theobromae*	Agarico	Basidio	54	72	18	53	197	GCA_009078325.1	Beltsville Agricultural Research Center
479	*Phialemoniopsis curvata*	Sordario	Asco	64	48	36	48	196	GCA_004353045.1	UES / AFRL
480	*Fusarium sp. NRRL 53497*	Sordario	Asco	51	64	37	44	196	GCA_013184445.1	US Department of Agriculture, Agriculture Research Service
481	*Fusarium equiseti*	Sordario	Asco	50	65	41	40	196	GCA_003313175.1	Gansu Academy of Agricultural Sciences
482	*Ganoderma sinense*	Agarico	Basidio	52	34	34	76	196	GCA_002760635.1	The Institute of Medicinal Plant Development, China
483	*Fusarium cuneirostrum*	Sordario	Asco	54	70	37	35	196	GCA_001680505.1	Iowa State University
484	*Kretzschmaria deusta*	Sordario	Asco	56	56	38	46	196	GCA_002081935.3	IHI Zittau / TU Dresden
485	*Fusarium incarnatum*	Sordario	Asco	53	65	40	38	196	GCA_004367075.1	US Department of Agriculture, Agriculture Research Service
486	*Fusarium caatingaense*	Sordario	Asco	52	66	40	38	196	GCA_013624355.1	US Department of Agriculture, Agriculture Research Service
487	*Curvularia papendorfii*	Dothideo	Asco	61	51	35	49	196	GCA_000817285.1	UNIVERSITY OF MALAYA

488	*Hymenoscyphus linearis*	Leotio	Asco	56	60	31	49	196	GCA_900536425.1	UNIVERSITY OF COPENHAGEN, DEPARTMENT OF GEOSCIENCES AND NATURAL RESOURCE MANAGEMENT
489	*Bipolaris oryzae*	Dothideo	Asco	60	52	34	50	196	GCA_000523455.1	JGI
490	*Cryphonectria radicalis*	Sordario	Asco	50	71	34	41	196	GCA_003054855.1	USDA-ARS
491	*Tinctoporellus epimiltinus*	Agarico	Basidio	55	36	34	71	196	GCA_900155495.1	BIOTECHNOLOGY RESEARCH INSTITUTE
492	*Cucurbitaria berberidis*	Dothideo	Asco	57	61	36	42	196	GCA_010015615.1	DOE Joint Genome Institute
493	*Talaromyces amestolkiae*	Eurotio	Asco	44	73	43	35	195	GCA_001896365.1	Centro de Investigaciones Biologicas
494	*Fusarium gaditjirri*	Sordario	Asco	51	60	40	44	195	GCA_013266175.1	US Department of Agriculture, Agriculture Research Service
495	*Fusarium tucumaniae*	Sordario	Asco	55	68	36	36	195	GCA_001680535.1	Iowa State University
496	*Neonectria hederae*	Sordario	Asco	54	77	30	34	195	GCA_003385265.1	USDA-ARS
497	*Cladosporium phlei*	Dothideo	Asco	60	54	35	46	195	GCA_003614995.1	National Institute of Agricultural Science
498	*Scedosporium boydii*	Sordario	Asco	66	25	49	55	195	GCA_002221725.1	GEIHP, UPRES EA 3142
499	*Epicoccum nigrum*	Dothideo	Asco	53	61	33	47	194	GCA_002116315.1	The University of Auckland
500	*Bipolaris victoriae*	Dothideo	Asco	60	49	36	49	194	GCA_000527765.1	JGI
501	*Shiraia sp. slf14*	Dothideo	Asco	59	49	40	46	194	GCA_000498155.1	Jiangxi Normal University
502	*Amphirosellinia nigrospora*	Sordario	Asco	56	55	36	47	194	GCA_004123355.1	National Institute of Biological Resources
503	*Bipolaris zeicola*	Dothideo	Asco	60	50	36	48	194	GCA_000523435.1	JGI
504	*Glonium stellatum*	Dothideo	Asco	55	43	35	61	194	GCA_001692915.1	1000 Fungal Genome Project
505	*Pseudogymnoascus sp. 03VT05*	Leotio	Asco	53	72	40	28	193	GCA_001662645.1	US Forest Service
506	*Hypoxylon pulicicidum*	Sordario	Asco	61	42	40	50	193	GCA_902806525.1	CEBITEC
507	*Parastagonospora avenae*	Dothideo	Asco	62	41	43	47	193	GCA_003503115.1	Centre for Crop & Disease Management, Curtin University
508	*Tulasnella calospora*	Agarico	Basidio	68	46	44	35	193	GCA_000827465.1	DOE Joint Genome Institute
509	*Auricularia auricula-judae*	Agarico	Basidio	52	35	44	61	192	GCA_002092955.1	Jilin Agricultural University
510	*Fusarium torulosum*	Sordario	Asco	49	62	40	41	192	GCA_013623875.1	US Department of Agriculture, Agriculture Research Service
511	*Magnaporthe sp. MG03*	Sordario	Asco	64	37	40	51	192	GCA_001936055.1	Centre for Cellular and Molecular Platforms
512	*Pseudogymnoascus sp. 23342-1-I1*	Leotio	Asco	60	66	34	32	192	GCA_001662575.1	US Forest Service
513	*Fusarium coffeatum*	Sordario	Asco	49	63	43	37	192	GCA_003316985.1	CSIRO
514	*Cryphonectria macrospora*	Sordario	Asco	48	70	33	41	192	GCA_004802535.1	USDA-ARS
515	*Cryphonectria nitschkei*	Sordario	Asco	48	70	33	41	192	GCA_006503525.1	USDA
516	*Hypomontagnella submonticulosa*	Sordario	Asco	63	41	40	48	192	GCA_902806495.1	CEBITEC
517	*Clarireedia sp. CPB17*	Leotio	Asco	54	57	32	49	192	GCA_002242865.1	Rutgers University
518	*Piromyces finnis*	Neocallimastigo	Chytridio	91	30	71	0	192	GCA_002104945.1	DOE Joint Genome Institute
519	*Auricularia heimuer*	Agarico	Basidio	53	37	41	60	191	GCA_002287115.1	Beijing Forestry University
520	*Zasmidium cellare*	Dothideo	Asco	49	50	31	61	191	GCA_010093935.1	DOE Joint Genome Institute

521	*Falciphora oryzae*	Sordario	Asco	67	36	36	52	191	GCA_000733355.1	Institute of Biotechnology
522	*Ascochyta rabiei*	Dothideo	Asco	47	78	26	40	191	GCA_004011695.1	Curtin University
523	*Diplodia seriata*	Dothideo	Asco	52	66	24	49	191	GCA_001975905.1	INRA
524	*Fusarium irregulare*	Sordario	Asco	50	63	40	38	191	GCA_004367085.1	US Department of Agriculture, Agriculture Research Service
525	*Bipolaris sorokiniana*	Dothideo	Asco	59	51	36	45	191	GCA_004329375.1	ICAR-Indian Agricultural Research Institute
526	*Cryphonectria parasitica*	Sordario	Asco	47	68	34	42	191	GCA_011745365.1	DOE Joint Genome Institute
527	*Xylaria sp. JS573*	Sordario	Asco	57	50	34	50	191	GCA_000966885.1	Seoul National University
528	*Xylaria hypoxylon*	Sordario	Asco	58	47	39	47	191	GCA_902806585.1	CEBITEC
529	*Golovinomyces magnicellulatus*	Leotio	Asco	38	70	38	44	190	GCA_006912115.1	The Ohio State University
530	*Morchella eximia*	Pezizo	Asco	51	80	26	33	190	GCA_003314645.1	Kunming University of Science and Technology
531	*Paraphaeosphaeria minitans*	Dothideo	Asco	57	50	35	48	190	GCA_009707825.1	State Key Laboratory of Agriculture Microbiology
532	*Aspergillus bombycis*	Eurotio	Asco	52	74	26	38	190	GCA_001792695.1	USDA-ARS-SRRC
533	*Aspergillus nomiae*	Eurotio	Asco	52	72	28	38	190	GCA_001204775.2	USDA-ARS-SRRC
534	*Fusarium sporotrichioides*	Sordario	Asco	49	65	37	39	190	GCA_003012315.1	Robert Proctor's shared submissions
535	*Fusarium armeniacum*	Sordario	Asco	50	60	39	41	190	GCA_013618295.1	US Department of Agriculture, Agriculture Research Service
536	*Fusarium camptoceras*	Sordario	Asco	50	65	38	37	190	GCA_004367475.1	US Department of Agriculture, Agriculture Research Service
537	*Fusarium cerealis*	Sordario	Asco	50	57	40	43	190	GCA_012600195.1	University of Minnesota
538	*Ophioceras dolichostomum*	Sordario	Asco	61	36	42	51	190	GCA_003049485.1	Agricultural Research Service, United States Department of Agriculture
539	*Aspergillus ochraceus*	Eurotio	Asco	50	81	27	32	190	GCA_005784425.1	Shanghai Institute of Technology
540	*Hypoxylon sp. E7406B*	Sordario	Asco	60	41	39	50	190	GCA_000931505.1	Yale University
541	*Fusarium clavum*	Sordario	Asco	50	66	36	37	189	GCA_004367155.1	US Department of Agriculture, Agriculture Research Service
542	*Phialocephala sp. D728*	Leotio	Asco	48	43	40	58	189	GCA_003988865.1	Manaaki Whenua Landcare Research
543	*Annulohypoxylon stygium*	Sordario	Asco	58	43	38	50	189	GCA_003314315.1	Kunming University of Science and Technology
544	*Hypoxylon sp. CO27-5*	Sordario	Asco	59	40	41	49	189	GCA_002120305.1	DOE Joint Genome Institute
545	*Dactylellina cionopaga*	Orbilio	Asco	49	73	35	32	189	GCA_012184355.1	Laboratory for Conservation and Utilization of Bio-Resources and Key Laboratory for Microbial Diversity in Southwest China, Ministry of Education
546	*Calonectria pseudonaviculata*	Sordario	Asco	49	69	34	36	188	GCA_001696505.1	USDA-ARS

547	*Magnaporthe sp. MG07*	Sordario	Asco	61	37	42	48	188	GCA_001936455.1	Centre for Cellular and Molecular Platforms
548	*Fusarium sp. NRRL 6227*	Sordario	Asco	49	60	39	40	188	GCA_013623825.1	US Department of Agriculture, Agriculture Research Service
549	*Fusarium luffae*	Sordario	Asco	48	64	38	38	188	GCA_013184325.1	US Department of Agriculture, Agriculture Research Service
550	*Clarireedia homoeocarpa*	Leotio	Asco	48	67	30	43	188	GCA_001465935.1	UMass Amherst
551	*Alternaria brassicae*	Dothideo	Asco	53	56	35	44	188	GCA_004936725.1	University of Delhi South Campus
552	*Fusarium hainanense*	Sordario	Asco	50	60	40	38	188	GCA_013618405.1	US Department of Agriculture, Agriculture Research Service
553	*Clarireedia sp. SE16F4*	Leotio	Asco	50	63	31	44	188	GCA_002242985.1	Rutgers University
554	*Peniophora sp. CBMAI 1063*	Agarico	Basidio	45	45	24	73	187	GCA_900536885.1	LABORATORIO NACIONAL DE CIENCIA E TECNOLOGIA DO BI
555	*Hysterium pulicare*	Dothideo	Asco	55	40	36	56	187	GCA_000467715.1	Assembling the Fungal Tree of Life (AFTOL)
556	*Hypoxylon sp. EC38*	Sordario	Asco	58	40	40	49	187	GCA_002120335.1	DOE Joint Genome Institute
557	*Pleurotus salmoneostramineus*	Agarico	Basidio	56	33	28	70	187	GCA_002933715.1	Applied Bioscience, Kindai University, Faculty of Agriculture
558	*Coniochaeta prunicola*	Sordario	Asco	54	50	46	36	186	GCA_007388105.1	University of Arizona
559	*Aspergillus pseudonomius*	Eurotio	Asco	51	73	26	36	186	GCA_009176395.1	DOE Joint Genome Institute
560	*Aspergillus fumigatiaffinis*	Eurotio	Asco	53	70	33	30	186	GCA_012656285.1	UNICAMP
561	*Fusarium virguliforme*	Sordario	Asco	55	65	30	36	186	GCA_013363175.1	US Department of Agriculture, Agriculture Research Service
562	*Fusarium compactum*	Sordario	Asco	49	62	34	41	186	GCA_013618385.1	US Department of Agriculture, Agriculture Research Service
563	*Daldinia childiae*	Sordario	Asco	64	38	34	50	186	GCA_008694065.1	National Institute of Biological Resources
564	*Spermospora avenae*	0	Asco	57	49	42	38	186	GCA_014282315.1	Agribio-Center for Agribioscience
565	*Hypoxylon sp. CI-4A*	Sordario	Asco	60	40	36	50	186	GCA_002120315.1	DOE Joint Genome Institute
566	*Fusarium nanum*	Sordario	Asco	46	60	43	36	185	GCA_004367095.1	US Department of Agriculture, Agriculture Research Service
567	*Entonaema liquescens*	Sordario	Asco	63	36	36	50	185	GCA_902805475.1	CEBITEC
568	*Magnaporthales sp. P1609*	Sordario	Asco	66	33	37	48	184	GCA_003709005.1	Fujian Agriculture and Forestry University
569	*Monosporascus cannonballus*	Sordario	Asco	52	54	32	46	184	GCA_004155895.1	University of New Mexico
570	*Pyricularia oryzae*	Sordario	Asco	63	34	38	49	184	GCA_004785725.1	Kansas State University
571	*Fusarium graminearum*	Sordario	Asco	48	61	36	39	184	GCA_900044135.1	ROTHAMSTED RESEARCH
572	*Helotiales sp. DMI_Dod_QoI*	Leotio	Asco	47	40	42	55	184	GCA_009613015.1	ARS
573	*Rhynchosporium agropyri*	Leotio	Asco	54	47	41	42	184	GCA_900074905.1	Technische Universitat Munchen - WZW

574	*Rhynchosporium commune*	Leotio	Asco	53	46	43	42	184	GCA_900074885.1	Technische Universitat Munchen - WZW
575	*Elsinoe arachidis*	Dothideo	Asco	46	68	32	38	184	GCA_013372555.1	Shenyang Agricultural University
576	*Pleurotus pulmonarius*	Agarico	Basidio	66	26	20	72	184	GCA_012980535.1	Academia Sinica
577	*Zasmidium angulare*	Dothideo	Asco	46	57	26	54	183	GCA_002786045.1	Northwest A&F University
578	*Pyricularia sp. CBS 133598*	Sordario	Asco	62	36	39	46	183	GCA_004337975.1	Academia Sinica
579	*Aspergillus lentulus*	Eurotio	Asco	53	69	34	27	183	GCA_010724575.1	Medical Mycology Research Center
580	*Coniochaeta sp. YLH0009*	Sordario	Asco	52	48	47	36	183	GCA_007388125.1	University of Arizona
581	*Fusarium anguioides*	Sordario	Asco	50	49	40	44	183	GCA_012977745.1	US Department of Agriculture, Agriculture Research Service
582	*Fusarium cortaderiae*	Sordario	Asco	49	59	34	41	183	GCA_009617495.1	University of Sao Paulo
583	*Fusarium transvaalense*	Sordario	Asco	52	58	35	38	183	GCA_013623685.1	US Department of Agriculture, Agriculture Research Service
584	*Diplodia sapinea*	Dothideo	Asco	47	67	23	46	183	GCA_000671355.1	Forestry and Agricultural Biotechnology Institute, University of Pretoria
585	*Fusarium sublunatum*	Sordario	Asco	44	72	29	38	183	GCA_013623665.1	US Department of Agriculture, Agriculture Research Service
586	*Clarireedia jacksonii*	Leotio	Asco	46	66	30	41	183	GCA_002242905.1	Rutgers University
587	*Podospora comata*	Sordario	Asco	58	23	48	54	183	GCA_900290415.1	IED
588	*Podospora anserina*	Sordario	Asco	58	23	48	54	183	GCA_005222925.1	Lomonosov Moscow State University
589	*Pseudocercospora macadamiae*	Dothideo	Asco	46	60	22	54	182	GCA_012978405.1	University of Queensland
590	*Magnaporthe sp. MG05*	Sordario	Asco	60	35	39	48	182	GCA_001936065.1	Centre for Cellular and Molecular Platforms
591	*Coniochaeta sp. NC1642*	Sordario	Asco	52	48	46	36	182	GCA_007388135.1	University of Arizona
592	*Coniochaeta sp. AEA 9055*	Sordario	Asco	53	48	45	36	182	GCA_007388195.1	University of Arizona
593	*Coniochaeta sp. AEA 9094*	Sordario	Asco	53	48	45	36	182	GCA_007388145.1	University of Arizona
594	*Didymella exigua*	Dothideo	Asco	46	78	19	39	182	GCA_010094145.1	DOE Joint Genome Institute
595	*Fusarium meridionale*	Sordario	Asco	48	59	35	40	182	GCA_009617515.1	University of Sao Paulo
596	*Valsa mali*	Sordario	Asco	52	66	20	44	182	GCA_000818155.1	Northwest A&F University
597	*Tubaria furfuracea*	Agarico	Basidio	38	93	30	20	181	GCA_900069095.1	Royal Botanic Gardens, Kew
598	*Coniochaeta sp. IL0111*	Sordario	Asco	53	48	44	36	181	GCA_007388115.1	University of Arizona
599	*Aspergillus leporis*	Eurotio	Asco	48	72	28	33	181	GCA_009176345.1	DOE Joint Genome Institute
600	*Fusarium austroamericanum*	Sordario	Asco	47	60	35	39	181	GCA_009617525.1	University of Sao Paulo
601	*Fusarium kyushuense*	Sordario	Asco	49	57	34	41	181	GCA_013184315.1	US Department of Agriculture, Agriculture Research Service
602	*Alternaria brassicicola*	Dothideo	Asco	50	56	34	41	181	GCA_002796735.1	IRHS
603	*Dendrothele bispora*	Agarico	Basidio	46	26	19	90	181	GCA_004369135.1	DOE Joint Genome Institute
604	*Gaeumannomyces sp. JS-464*	Sordario	Asco	64	31	35	50	180	GCA_002197995.1	National Institute of Biological Resources
605	*Monosporascus sp. MC13-8B*	Sordario	Asco	52	53	32	43	180	GCA_004154975.1	University of New Mexico

606	*Lizonia empirigonia*	Dothideo	Asco	50	62	26	42	180	GCA_009982855.1	DOE Joint Genome Institute
607	*Fusarium brasilicum*	Sordario	Asco	47	59	35	39	180	GCA_013184295.1	US Department of Agriculture, Agriculture Research Service
608	*Polyporus brumalis*	Agarico	Basidio	59	28	26	67	180	GCA_001792895.1	National Institute of Forest Science
609	*Didymosphaeria enalia*	Dothideo	Asco	59	33	32	56	180	GCA_010094045.1	DOE Joint Genome Institute
610	*Laburnicola sp. R22_1*	Dothideo	Asco	52	54	32	41	179	GCA_014281115.1	Chinese Academy of Forestry
611	*Fusarium asiaticum*	Sordario	Asco	48	58	35	38	179	GCA_001717845.1	Government of Canada
612	*Penicillium sclerotiorum*	Eurotio	Asco	46	70	28	35	179	GCA_001750025.1	Rutgers, The State University of New Jersey
613	*Fusarium culmorum*	Sordario	Asco	46	58	36	39	179	GCA_003033665.1	NIOO-KNAW
614	*Fusarium heterosporum*	Sordario	Asco	49	53	38	39	179	GCA_013396295.1	US Department of Agriculture, Agriculture Research Service
615	*Scedosporium dehoogii*	Sordario	Asco	64	20	44	51	179	GCA_002812735.1	GEIHP, UPRES EA 3142
616	*Magnaporthe sp. MG08*	Sordario	Asco	59	35	38	46	178	GCA_001936555.1	Centre for Cellular and Molecular Platforms
617	*Diplodia corticola*	Dothideo	Asco	46	66	22	44	178	GCA_001883845.1	University of Aveiro
618	*Fusarium dimerum*	Sordario	Asco	45	68	32	33	178	GCA_013623525.1	US Department of Agriculture, Agriculture Research Service
619	*Fusarium praegraminearum*	Sordario	Asco	48	57	36	37	178	GCA_002093855.1	US Department of Agriculture, Agriculture Research Service
620	*Pyrenophora tritici-repentis*	Dothideo	Asco	51	49	31	47	178	GCA_008692205.1	Curtin University
621	*Rhynchosporium secalis*	Leotio	Asco	54	42	41	41	178	GCA_900074895.1	Technische Universitat Munchen - WZW
622	*Grammothele lineata*	Agarico	Basidio	49	30	30	69	178	GCA_002150815.3	Molecular Biology Lab
623	*Fusarium illudens*	Sordario	Asco	50	63	30	35	178	GCA_013623515.1	US Department of Agriculture, Agriculture Research Service
624	*Auriculariopsis ampla*	Agarico	Basidio	48	54	32	43	177	GCA_007026445.1	DOE Joint Genome Institute
625	*Aspergillus felis*	Eurotio	Asco	47	66	37	27	177	GCA_014281915.1	UNICAMP
626	*Aspergillus bertholletiae*	Eurotio	Asco	48	72	23	34	177	GCA_009193595.1	DOE Joint Genome Institute
627	*Curvularia sp. IFB-Z10*	Dothideo	Asco	55	42	33	47	177	GCA_002161795.1	Nanjing University
628	*Annulohypoxylon truncatum*	Sordario	Asco	51	42	36	48	177	GCA_902805465.1	CEBITEC
629	*Exserohilum turcicum*	Dothideo	Asco	53	48	33	43	177	GCA_000359705.1	JGI
630	*Taxomyces andreanae*	0	Asco	61	18	31	67	177	GCA_001969225.1	Fraunhofer
631	*Moniliophthora roreri*	Agarico	Basidio	54	31	22	70	177	GCA_001466705.1	Purdue University
632	*Phanerochaete chrysosporium*	Agarico	Basidio	60	18	25	74	177	GCA_001910725.1	National Institute of Forest Science
633	*Scedosporium sp. IMV 00882*	Sordario	Asco	63	20	43	51	177	GCA_001931805.1	Jet Propulsion Laboratory, California Institute of Technology
634	*Magnaporthe sp. MG12*	Sordario	Asco	59	35	37	45	176	GCA_001936955.1	Centre for Cellular and Molecular Platforms
635	*Gaeumannomyces tritici*	Sordario	Asco	61	32	34	49	176	GCA_000145635.1	Broad Institute
636	*Aspergillus quadrilineatus*	Eurotio	Asco	46	69	30	31	176	GCA_013305525.1	Vanderbilt University

637	*Fusarium pseudograminearum*	Sordario	Asco	46	57	36	37	176	GCA_000303195.2	CSIRO
638	*Aspergillus udagawae*	Eurotio	Asco	49	64	32	30	175	GCA_010724255.1	Medical Mycology Research Center
639	*Hawksworthiomyces lignivorus*	Sordario	Asco	49	37	44	45	175	GCA_002917075.1	University of Pretoria
640	*Bipolaris cookei*	Dothideo	Asco	56	44	30	45	175	GCA_002286855.1	University of Arkansas
641	*Fusarium zanthoxyli*	Sordario	Asco	40	69	31	35	175	GCA_013623745.1	US Department of Agriculture, Agriculture Research Service
642	*Daldinia eschscholtzii*	Sordario	Asco	57	38	34	46	175	GCA_001951055.1	nanjing university
643	*Daldinia concentrica*	Sordario	Asco	57	38	34	46	175	GCA_902805455.1	CEBITEC
644	*Alternaria gansuensis*	Dothideo	Asco	51	51	30	43	175	GCA_009289805.1	Lanzhou University
645	*Clavaria fumosa*	Agarico	Basidio	26	78	45	25	174	GCA_001179745.1	Royal Botanic Gardens, Kew
646	*Microdochium bolleyi*	Sordario	Asco	52	52	27	43	174	GCA_001566295.1	DOE Joint Genome Institute
647	*Aspergillus terreus*	Eurotio	Asco	49	57	39	29	174	GCA_009834425.1	Nanyang Technological University
648	*Fusarium longipes*	Sordario	Asco	46	51	37	40	174	GCA_013618495.1	US Department of Agriculture, Agriculture Research Service
649	*Diplodia scrobiculata*	Dothideo	Asco	44	64	20	46	174	GCA_001455585.1	Forestry & Agricultural Biotechnology Institute, University of Pretoria
650	*Fusarium subtropicale*	Sordario	Asco	47	57	33	37	174	GCA_003670145.1	US Department of Agriculture, Agriculture Research Service
651	*Lanzia echinophila*	Leotio	Asco	53	53	18	50	174	GCA_000812885.1	USDA-ARS
652	*Coriolopsis trogii*	Agarico	Basidio	48	32	24	70	174	GCA_007896425.1	Kunming University of Science and Technology
653	*Hypoxylon rickii*	Sordario	Asco	55	37	36	46	174	GCA_902806535.1	CEBITEC
654	*Armillaria cepistipes*	Agarico	Basidio	47	39	20	68	174	GCA_900157415.1	Technische Universitat Munchen - WZW
655	*Uromyces transversalis*	Puccinio	Basidio	50	64	19	40	173	GCA_002994575.1	University of Georgia
656	*Cercospora brassicicola*	Dothideo	Asco	47	40	28	58	173	GCA_013365245.1	Banaras Hindu University
657	*Fusarium poae*	Sordario	Asco	45	54	33	41	173	GCA_001675295.1	Wageningen UR
658	*Sarocladium brachiariae*	Sordario	Asco	55	41	47	30	173	GCA_008271525.1	Environment and Plant Protection Institute
659	*Dactylellina haptotyla*	Orbilio	Asco	47	72	27	27	173	GCA_000441935.1	Lund University
660	*Armillaria gallica*	Agarico	Basidio	51	32	17	73	173	GCA_012064365.1	Yunnan Minzu University
661	*Cantharellus lutescens*	Agarico	Basidio	30	81	41	20	172	GCA_003314295.1	Kunming University of Science and Technology
662	*Penicillium arizonense*	Eurotio	Asco	50	64	23	35	172	GCA_001773325.1	Chalmers University of Technology
663	*Valsa malicola*	Sordario	Asco	44	68	23	37	172	GCA_003795315.1	Northwest A&F University
664	*Aspergillus mulundensis*	Eurotio	Asco	50	70	25	27	172	GCA_003369625.1	The University of Texas Health Science Center at Houston
665	*Daldinia sp. EC12*	Sordario	Asco	54	36	38	44	172	GCA_002120325.1	DOE Joint Genome Institute
666	*Leptosphaeria biglobosa*	Dothideo	Asco	48	54	31	39	172	GCA_900465125.1	GSC

667	*Penicillium capsulatum*	Eurotio	Asco	52	58	23	38	171	GCA_000943775.1	Shanghai Key Laboratory of Molecular Medical Mycology, Department of Dermatology, Shanghai Changzheng Hospital; Academy of Military Medical Sciences
668	*Magnaporthiopsis rhizophila*	Sordario	Asco	58	35	35	43	171	GCA_003049465.1	Agricultural Research Service, United States Department of Agriculture
669	*Laburnicola sp. JP-R-44*	Dothideo	Asco	46	56	31	38	171	GCA_009805535.1	Chinese Academy of Forestry
670	*Coniochaeta hoffmannii*	Sordario	Asco	51	37	44	39	171	GCA_002798055.1	IHI Zittau / TU Dresden
671	*Aspergillus spinulosporus*	Eurotio	Asco	48	69	25	29	171	GCA_013305485.1	Vanderbilt University
672	*Polyporus arcularius*	Agarico	Basidio	46	28	26	71	171	GCA_004369055.1	DOE Joint Genome Institute
673	*Pseudogymnoascus sp. VKM F-4281 (FW-2241)*	Leotio	Asco	53	52	36	30	171	GCA_000750745.1	Moscow State University
674	*Magnaporthiopsis incrustans*	Sordario	Asco	55	35	35	45	170	GCA_003049425.1	Agricultural Research Service, United States Department of Agriculture
675	*Pyricularia grisea*	Sordario	Asco	59	32	35	44	170	GCA_003933175.1	IARI
676	*Pyricularia pennisetigena*	Sordario	Asco	60	32	35	43	170	GCA_004337985.1	Academia Sinica
677	*Talaromyces stollii*	Eurotio	Asco	36	65	38	31	170	GCA_014065225.1	Beijing University of Chemical Technology
678	*Ganoderma lucidum*	Agarico	Basidio	44	34	28	64	170	GCA_000271565.1	The Institute of Medicinal Plant Development
679	*Aureobasidium pullulans*	Dothideo	Asco	47	60	28	35	170	GCA_000721785.1	DOE Joint Genome Institute
680	*Elsinoe fawcettii*	Dothideo	Asco	42	69	26	33	170	GCA_012977835.1	University of Southern Queensland
681	*Basipetospora chlamydospora*	Eurotio	Asco	52	17	59	42	170	GCA_001599675.1	RIKEN Center for Life Science Technologies, Division of Genomic Technologies
682	*Aspergillus novofumigatus*	Eurotio	Asco	46	70	29	24	169	GCA_002847465.1	DOE Joint Genome Institute
683	*Penicillium brasilianum*	Eurotio	Asco	49	63	28	29	169	GCA_001048715.1	HKI JENA
684	*Aspergillus pseudoterreus*	Eurotio	Asco	48	52	39	30	169	GCA_002927005.1	Pacific Northwest National Lab
685	*Fusarium langsethiae*	Sordario	Asco	49	52	32	36	169	GCA_001292635.1	Bioforsk
686	*Pirottaea palmicola*	Leotio	Asco	54	40	30	45	169	GCA_003988945.1	Manaaki Whenua Landcare Research
687	*Talaromyces rugulosus*	Eurotio	Asco	36	58	30	44	168	GCA_013368755.1	Xi'an Jiaotong University
688	*Fusarium penzigii*	Sordario	Asco	47	59	32	30	168	GCA_013623535.1	US Department of Agriculture, Agriculture Research Service
689	*Sarocladium oryzae*	Sordario	Asco	55	38	43	32	168	GCA_001972265.1	RIKEN Center for Life Science Technologies, Division of Genomic Technologies
690	*Stereum hirsutum*	Agarico	Basidio	46	41	21	60	168	GCA_000264905.1	JGI
691	*Botrytis tulipae*	Leotio	Asco	48	58	18	44	168	GCA_004786125.1	Wageningen University

692	*Nakataea oryzae*	Sordario	Asco	56	30	36	45	167	GCA_003049435.1	Agricultural Research Service, United States Department of Agriculture
693	*Jackrogersella multiformis*	Sordario	Asco	49	41	34	43	167	GCA_902806575.1	CEBITEC
694	*Cladosporium sphaerospermum*	Dothideo	Asco	42	54	32	39	167	GCA_000261425.2	UNIVERSITY OF MALAYA
695	*Talaromyces wortmannii*	Eurotio	Asco	36	58	34	38	166	GCA_001939245.1	Universidad Nacional Agraria La Molina,
696	*Mycena chlorophos*	Agarico	Basidio	40	35	27	64	166	GCA_001612595.1	KEIO UNIVERSITY
697	*Pseudocercospora pini-densiflorae*	Dothideo	Asco	44	51	21	50	166	GCA_000504365.2	Canada's Michael Smith Genome Sciences Centre
698	*Aspergillus thermomutatus*	Eurotio	Asco	47	62	30	27	166	GCA_002237265.2	University of Montreal
699	*Valsa sordida*	Sordario	Asco	42	66	23	35	166	GCA_003795275.1	Northwest A&F University
700	*Penicillium paxilli*	Eurotio	Asco	42	68	21	35	166	GCA_000347475.1	Massey University
701	*Aspergillus brasiliensis*	Eurotio	Asco	44	65	18	39	166	GCA_001889945.1	DOE Joint Genome Institute
702	*Fusarium nurragi*	Sordario	Asco	44	51	36	35	166	GCA_012977755.1	US Department of Agriculture, Agriculture Research Service
703	*Fusarium continuum*	Sordario	Asco	40	64	28	34	166	GCA_013184455.1	US Department of Agriculture, Agriculture Research Service
704	*Cladosporium sp. TM138-S3*	Dothideo	Asco	42	53	32	39	166	GCA_011745625.1	Hellenic Centre of Marine Research (HCMR)
705	*Botrytis fabae*	Leotio	Asco	45	59	20	42	166	GCA_004335055.1	Curtin University
706	*Botrytis elliptica*	Leotio	Asco	45	58	22	41	166	GCA_004786205.1	Wageningen University
707	*Botrytis galanthina*	Leotio	Asco	46	59	20	41	166	GCA_004916875.1	Wageningen University
708	*Pterula gracilis*	Agarico	Basidio	55	28	22	61	166	GCA_004369125.1	DOE Joint Genome Institute
709	*Fusarium zealandicum*	Sordario	Asco	42	68	24	32	166	GCA_013266195.1	US Department of Agriculture, Agriculture Research Service
710	*Dictyopanus pusillus*	Agarico	Basidio	36	26	32	72	166	GCA_013387415.1	INRS-Institut Armand-Frappier
711	*Puccinia arachidis*	Puccinio	Basidio	52	54	34	25	165	GCA_001013415.1	Junagadh Agricultural University
712	*Aspergillus fischeri*	Eurotio	Asco	50	62	27	26	165	GCA_008711165.1	UMass Amherst
713	*Lentinus tigrinus*	Agarico	Basidio	43	28	24	70	165	GCA_003813185.1	DOE Joint Genome Institute
714	*Massariosphaeria phaeospora*	Dothideo	Asco	51	30	35	49	165	GCA_011032825.1	DOE Joint Genome Institute
715	*Aspergillus sp. CLMG-2019a*	Eurotio	Asco	47	60	22	35	164	GCA_013421405.1	The University of Western Australia
716	*Aspergillus nidulans*	Eurotio	Asco	42	64	26	32	164	GCA_011074995.1	Barcelona Supercomputing center (BSC)
717	*Pyrenophora teres*	Dothideo	Asco	48	40	32	44	164	GCA_008086845.1	NDSU
718	*Botrytis cinerea*	Leotio	Asco	44	59	20	41	164	GCA_000292645.1	Wageningen University
719	*Aspergillus welwitschiae*	Eurotio	Asco	42	67	16	39	164	GCA_009761105.1	Universidade Federal de Minas Gerais
720	*Aureobasidium namibiae*	Dothideo	Asco	46	53	29	36	164	GCA_000721765.1	DOE Joint Genome Institute
721	*Pyrenophora graminea*	Dothideo	Asco	48	40	32	43	163	GCA_012365135.1	USDA-ARS
722	*Pyrenophora seminiperda*	Dothideo	Asco	48	41	33	41	163	GCA_000465215.2	Brigham Young University
723	*Aspergillus phoenicis*	Eurotio	Asco	42	68	14	39	163	GCA_003344505.1	DOE Joint Genome Institute

724	*Botrytis hyacinthi*	Leotio	Asco	44	58	20	41	163	GCA_004786245.1	Wageningen University
725	*Coprinellus angulatus*	Agarico	Basidio	44	12	50	57	163	GCA_013368325.1	Lund University
726	*Delitschia confertaspora*	Dothideo	Asco	48	26	36	53	163	GCA_010093945.1	DOE Joint Genome Institute
727	*Pseudocercospora fuligena*	Dothideo	Asco	46	50	18	48	162	GCA_014298035.1	University of California
728	*Leptosphaeria maculans*	Dothideo	Asco	47	50	28	37	162	GCA_900465115.1	GSC
729	*Botrytis paeoniae*	Leotio	Asco	46	55	18	43	162	GCA_001005785.1	Washington State University
730	*Dichomitus squalens*	Agarico	Basidio	46	31	21	64	162	GCA_004307925.1	DOE Joint Genome Institute
731	*Trametes pubescens*	Agarico	Basidio	48	23	23	68	162	GCA_001895945.1	CBS-KNAW Fungal Biodiversity Centre Institute of the Royal Netherlands Academy of Arts and Science
732	*Psathyrella aberdarensis*	Agarico	Basidio	56	8	36	62	162	GCA_004126415.1	IHI Zittau / TU Dresden
733	*Steccherinum ochraceum*	Agarico	Basidio	50	16	30	66	162	GCA_004332605.1	The Federal Research Centre "Fundamentals of Biotechnology" of the Russian Academy of Sciences
734	*Friedmanniomyces simplex*	Dothideo	Asco	43	34	44	40	161	GCA_005059865.1	University of California, Riverside
735	*Trametes versicolor*	Agarico	Basidio	46	24	23	68	161	GCA_000271585.1	JGI
736	*Botrytis fragariae*	Leotio	Asco	45	56	18	42	161	GCA_013461495.1	University of Maryland, College Park
737	*Agrocybe cylindracea*	Agarico	Basidio	55	20	24	62	161	GCA_013376435.1	Beijing Institute of Genomics, CAS
738	*Scedosporium aurantiacum*	Sordario	Asco	58	17	40	46	161	GCA_000812075.1	Australian National University
739	*Macrolepiota dolichaula*	Agarico	Basidio	45	18	26	72	161	GCA_003315915.1	Kunming University of Science and Technology
740	*Thelonectria rubi*	Sordario	Asco	42	58	29	31	160	GCA_013420875.1	USDA-ARS
741	*Botryotinia calthae*	Leotio	Asco	46	54	20	40	160	GCA_004379285.1	Wageningen University
742	*Botryotinia narcissicola*	Leotio	Asco	45	56	18	41	160	GCA_004786225.1	Wageningen University
743	*Neobulgaria alba*	Leotio	Asco	48	58	30	24	160	GCA_003988965.1	Manaaki Whenua Landcare Research
744	*Armillaria ostoyae*	Agarico	Basidio	39	42	22	57	160	GCA_900157425.1	Technische Universitat Munchen - WZW
745	*Pseudocercospora cruenta*	Dothideo	Asco	41	49	21	48	159	GCA_013365205.1	Banaras Hindu University
746	*Aspergillus alliaceus*	Eurotio	Asco	43	61	22	33	159	GCA_009176365.1	DOE Joint Genome Institute
747	*Raffaelea albimanens*	Sordario	Asco	50	34	34	41	159	GCA_002778245.1	University of Montana
748	*Aureobasidium sp. FSWF8-4*	Dothideo	Asco	44	58	24	33	159	GCA_001914275.1	Uppsala University
749	*Clathrospora elynae*	Dothideo	Asco	46	38	35	40	159	GCA_010015635.1	DOE Joint Genome Institute
750	*Graphilbum fragrans*	Sordario	Asco	49	40	32	37	158	GCA_001513895.1	University of Pretoria
751	*Cytospora leucostoma*	Sordario	Asco	40	60	24	34	158	GCA_003795295.1	Northwest A&F University
752	*Penicillium citrinum*	Eurotio	Asco	42	62	20	34	158	GCA_001950535.1	RIKEN Center for Life Science Technologies, Division of Genomic Technologies
753	*Corinectria fuckeliana*	Sordario	Asco	44	61	22	31	158	GCA_003385255.1	USDA-ARS

754	*Botryotinia convoluta*	Leotio	Asco	46	52	19	41	158	GCA_004786275.1	Wageningen University
755	*Venustampulla echinocandica*	Leotio	Asco	48	32	39	39	158	GCA_003357145.1	The University of Texas Health Science Center at Houston
756	*Punctularia strigosozonata*	Agarico	Basidio	36	44	24	54	158	GCA_000264995.1	JGI
757	*Crucibulum laeve*	Agarico	Basidio	54	19	22	63	158	GCA_004379715.1	DOE Joint Genome Institute
758	*Leucoagaricus leucothites*	Agarico	Basidio	50	20	20	68	158	GCA_013368445.1	Lund University
759	*Pleurotus citrinopileatus*	Agarico	Basidio	46	24	20	68	158	GCA_003314595.1	Kunming University of Science and Technology
760	*Aspergillus unguis*	Eurotio	Asco	52	55	20	30	157	GCA_003324175.1	CSIR-NIIST
761	*Raffaelea ambrosiae*	Sordario	Asco	47	36	34	40	157	GCA_002778195.1	University of Montana
762	*Aspergillus incahuasiensis*	Eurotio	Asco	44	60	29	24	157	GCA_003719405.1	National Center For Agricultural Utilization Research
763	*Sporothrix pallida*	Sordario	Asco	48	38	38	33	157	GCA_000710705.2	University of Messina
764	*Aspergillus aculeatinus*	Eurotio	Asco	40	64	21	32	157	GCA_003184765.1	DOE Joint Genome Institute
765	*Lachnellula willkommii*	Leotio	Asco	50	46	12	49	157	GCA_007825375.1	Canadian Food Inspection Agency (CFIA)
766	*Penicillium steckii*	Eurotio	Asco	42	62	18	35	157	GCA_002072375.1	Chalmers University of Technology
767	*Aspergillus tubingensis*	Eurotio	Asco	42	66	16	33	157	GCA_010614855.1	Hankyong National University
768	*Elsinoe ampelina*	Dothideo	Asco	42	54	30	31	157	GCA_005959805.1	Northwest A&F University
769	*Armillaria solidipes*	Agarico	Basidio	38	40	24	55	157	GCA_002307675.1	DOE Joint Genome Institute
770	*Aspergillus avenaceus*	Eurotio	Asco	40	66	20	30	156	GCA_009193465.1	DOE Joint Genome Institute
771	*Xenoacremonium recifei*	Sordario	Asco	53	52	22	29	156	GCA_012184525.1	Belgian Coordinated Collection of Microorganisms, BCCM
772	*Pyrenopolyporus hunteri*	Sordario	Asco	47	35	34	40	156	GCA_902806595.1	CEBITEC
773	*Aspergillus piperis*	Eurotio	Asco	42	64	14	36	156	GCA_003184755.1	DOE Joint Genome Institute
774	*Fusarium verrucosum*	Sordario	Asco	44	41	35	36	156	GCA_013623715.1	US Department of Agriculture, Agriculture Research Service
775	*Rhizoctonia solani*	Agarico	Basidio	39	58	16	43	156	GCA_001899475.2	Universiti Kebangsaan Malaysia
776	*Aspergillus viridinutans*	Eurotio	Asco	44	60	26	24	154	GCA_004368095.1	University of Melbourne
777	*Cylindrobasidium torrendii*	Agarico	Basidio	44	52	25	33	154	GCA_000934385.1	DOE Joint Genome Institute
778	*Aspergillus luchuensis*	Eurotio	Asco	41	64	14	35	154	GCA_001890685.1	DOE Joint Genome Institute
779	*Lachnellula arida*	Leotio	Asco	49	42	14	48	153	GCA_007821475.1	Canadian Food Inspection Agency (CFIA)
780	*Pezoloma ericae*	Leotio	Asco	40	55	22	36	153	GCA_002865625.1	DOE Joint Genome Institute
781	*Aspergillus brunneoviolaceus*	Eurotio	Asco	38	64	18	33	153	GCA_003184695.1	DOE Joint Genome Institute
782	*Fusarium xyrophilum*	Sordario	Asco	34	50	35	34	153	GCA_008711595.1	US Department of Agriculture, Agriculture Research Service
783	*Trametes polyzona*	Agarico	Basidio	42	26	27	58	153	GCA_001939255.1	Universidad Nacional Agraria La Molina,
784	*Aspergillus hiratsukae*	Eurotio	Asco	43	55	26	28	152	GCA_014281905.1	UNICAMP
785	*Venturia oleaginea*	Dothideo	Asco	43	48	14	47	152	GCA_013176395.1	FUJIAN AGRICULTURE AND FORESTRY UNIVERSITY

786	*Fusarium sp. KOD 1611*	Sordario	Asco	34	49	35	34	152	GCA_013624395.1	US Department of Agriculture, Agriculture Research Service
787	*Penicillium rolfsii*	Eurotio	Asco	44	59	27	22	152	GCA_011392555.1	Embrapa Agroenergia
788	*Trametes sp. AH28-2*	Agarico	Basidio	44	24	20	64	152	GCA_001304625.1	Anhui University
789	*Phanerochaete carnosa*	Agarico	Basidio	53	16	21	62	152	GCA_000300595.1	DOE Joint Genome Institute
790	*Aspergillus olivimuriae*	Eurotio	Asco	42	53	32	24	151	GCA_003719415.1	National Center For Agricultural Utilization Research
791	*Aspergillus oerlinghausenensis*	Eurotio	Asco	42	58	27	24	151	GCA_014250555.1	Vanderbilt University
792	*Esteya vermicola*	Sordario	Asco	59	25	32	35	151	GCA_002778215.1	University of Montana
793	*Lachnellula cervina*	Leotio	Asco	46	46	15	44	151	GCA_007825325.1	Canadian Food Inspection Agency (CFIA)
794	*Aspergillus costaricensis*	Eurotio	Asco	40	64	14	33	151	GCA_003184835.1	DOE Joint Genome Institute
795	*Aspergillus niger*	Eurotio	Asco	40	63	14	34	151	GCA_011316255.1	South China Agricultural University
796	*Hericium coralloides*	Agarico	Basidio	54	21	24	52	151	GCA_003675405.1	Tibet Vocational Technical College
797	*Agrocybe pediades*	Agarico	Basidio	44	17	24	66	151	GCA_013053245.1	Lund University
798	*Pleurotus ostreatus*	Agarico	Basidio	54	21	18	58	151	GCA_003313235.2	Chinese Academy of Agricultural Sciences
799	*Pallidocercospora crystallina*	Dothideo	Asco	41	46	15	48	150	GCA_003666085.1	Xijing Hospital, Fourth Military Medical University
800	*Passalora fulva*	Dothideo	Asco	38	52	20	40	150	GCA_000301015.1	Wageningen UR (University & Research centre)
801	*Magnaporthiopsis poae*	Sordario	Asco	48	33	30	39	150	GCA_000193285.1	Broad Institute
802	*Aspergillus turcosus*	Eurotio	Asco	42	58	25	25	150	GCA_002234965.2	University of Montreal
803	*Penicillium fuscoglaucum*	Eurotio	Asco	46	50	18	36	150	GCA_000576735.1	INRA-LIPM
804	*Coniella lustricola*	Sordario	Asco	38	60	26	26	150	GCA_003019895.1	DOE Joint Genome Institute
805	*Penicillium sp. MA 6036*	Eurotio	Asco	46	49	17	38	150	GCA_003138045.1	University of Natural Resources and Life Sciences, Vienna
806	*Aspergillus fijiensis*	Eurotio	Asco	39	62	18	31	150	GCA_003184825.1	DOE Joint Genome Institute
807	*Aspergillus uvarum*	Eurotio	Asco	38	62	18	32	150	GCA_003184745.1	DOE Joint Genome Institute
808	*Crepidotus sp. BD-2015*	Agarico	Basidio	36	28	24	62	150	GCA_001179765.1	Royal Botanic Gardens, Kew
809	*Aspergillus awamori*	Eurotio	Asco	40	64	14	32	150	GCA_001599415.1	RIKEN Center for Life Science Technologies, Division of Genomic Technologies
810	*Trametes hirsuta*	Agarico	Basidio	42	22	24	62	150	GCA_001302255.2	A.N. Bach Institute of Biochemistry
811	*Lepista sordida*	Agarico	Basidio	44	22	22	62	150	GCA_900168675.1	UNIVERSITY OF BRISTOL
812	*Cercospora sesami*	Dothideo	Asco	42	38	27	42	149	GCA_013365235.1	Banaras Hindu University
813	*Penicillium sp. SPG-F15*	Eurotio	Asco	46	46	17	40	149	GCA_003800485.1	Texas A&M University - Corpus Christi
814	*Aspergillus japonicus*	Eurotio	Asco	39	62	16	32	149	GCA_003184785.1	DOE Joint Genome Institute
815	*Aspergillus indologenus*	Eurotio	Asco	38	63	18	30	149	GCA_003184685.1	DOE Joint Genome Institute
816	*Aspergillus violaceofuscus*	Eurotio	Asco	40	60	16	33	149	GCA_003184705.1	DOE Joint Genome Institute

817	*Penicillium expansum*	Eurotio	Asco	51	48	16	34	149	GCA_004302965.1	ARS
818	*Aspergillus vadensis*	Eurotio	Asco	38	64	14	33	149	GCA_003184925.1	DOE Joint Genome Institute
819	*Aureobasidium subglaciale*	Dothideo	Asco	44	53	24	28	149	GCA_000721755.1	JGI
820	*Dothidotthia symphoricarpi*	Dothideo	Asco	42	60	16	31	149	GCA_010015815.1	DOE Joint Genome Institute
821	*Aspergillus floridensis*	Eurotio	Asco	41	60	16	32	149	GCA_012184565.1	Belgian Coordinated Collection of Microorganisms, BCCM
822	*Aureobasidium melanogenum*	Dothideo	Asco	42	45	24	38	149	GCA_002156615.1	Ocean University of China
823	*Thermothielavioides terrestris*	Sordario	Asco	45	32	36	36	149	GCA_900343105.1	CHALMERS UNIVERSITY OF TECHNOLOGY
824	*Armillaria borealis*	Agarico	Basidio	40	31	22	56	149	GCA_013427175.2	Genome Research and Education Center, Siberian Federal University
825	*Coniella vitis*	Sordario	Asco	35	60	24	29	148	GCA_011317545.1	qingdao agricultural university
826	*Elsinoe australis*	Dothideo	Asco	38	58	24	28	148	GCA_003013795.1	Nanjing Forestry University
827	*Aspergillus neoniger*	Eurotio	Asco	38	60	14	35	147	GCA_003184625.1	DOE Joint Genome Institute
828	*Monilinia laxa*	Leotio	Asco	41	50	20	36	147	GCA_009299455.1	UniBa - Univpm
829	*Galerina marginata*	Agarico	Basidio	42	26	27	52	147	GCA_000697645.1	DOE Joint Genome Institute
830	*Rhizodiscina lignyota*	Dothideo	Asco	34	46	24	42	146	GCA_010015805.1	DOE Joint Genome Institute
831	*Fusarium aywerte*	Sordario	Asco	36	46	34	30	146	GCA_013186375.1	US Department of Agriculture, Agriculture Research Service
832	*Botrytis porri*	Leotio	Asco	42	52	16	36	146	GCA_004786265.1	Wageningen University
833	*Viridothelium virens*	Dothideo	Asco	36	39	32	39	146	GCA_010094025.1	DOE Joint Genome Institute
834	*Morchella crassipes*	Pezizo	Asco	44	56	22	24	146	GCA_009192285.1	Huazhong Agricultural University
835	*Aspergillus eucalypticola*	Eurotio	Asco	38	60	14	33	145	GCA_003184535.1	DOE Joint Genome Institute
836	*Fusarium domesticum*	Sordario	Asco	43	66	16	20	145	GCA_013618395.1	US Department of Agriculture, Agriculture Research Service
837	*Marssonina brunnea*	Leotio	Asco	41	46	22	36	145	GCA_011750725.1	Nanjing Forestry University
838	*Morchella septimelata*	Pezizo	Asco	42	54	22	27	145	GCA_003062385.1	Biotechnology and Nuclear Technology Research Institute
839	*Friedmanniomyces endolithicus*	Dothideo	Asco	40	24	46	34	144	GCA_005059855.1	University of California, Riverside
840	*Penicillium sp. str. #12*	Eurotio	Asco	46	48	18	32	144	GCA_013138035.1	None
841	*Microthyrium microscopicum*	Dothideo	Asco	38	33	24	49	144	GCA_010405405.1	DOE Joint Genome Institute
842	*Penicillium sp. BW_12*	Eurotio	Asco	46	48	18	32	144	GCA_008931925.1	Tufts University
843	*Penicillium antarcticum*	Eurotio	Asco	44	54	16	30	144	GCA_002072345.1	Chalmers University of Technology
844	*Aspergillus wentii*	Eurotio	Asco	40	50	16	38	144	GCA_001890725.1	DOE Joint Genome Institute
845	*Agrocybe aegerita*	Agarico	Basidio	52	18	22	52	144	GCA_902728275.1	Integrative Pilzforschung
846	*Monilinia fructicola*	Leotio	Asco	42	45	18	38	143	GCA_008692225.1	UniBa - Univpm
847	*Chondrostereum purpureum*	Agarico	Basidio	35	23	16	69	143	GCA_004354395.1	Internationales Hochschulinstitut Zittau
848	*Raffaelea sp. RL272*	Sordario	Asco	51	43	16	32	142	GCA_002777955.1	University of Montana

849	*Schizophyllum commune*	Agarico	Basidio	40	40	26	36	142	GCA_001599475.1	RIKEN Center for Life Science Technologies, Division of Genomic Technologies
850	*Scytalidium lignicola*	Leotio	Asco	40	30	30	42	142	GCA_002812745.2	IHI Zittau / TU Dresden
851	*Aspergillus neoellipticus*	Eurotio	Asco	39	58	25	20	142	GCA_003116565.1	personal
852	*Mycosphaerelloides madeirae*	Dothideo	Asco	42	38	16	46	142	GCA_002785995.1	Northwest A&F University
853	*Aspergillus homomorphus*	Eurotio	Asco	39	59	16	28	142	GCA_003184865.1	DOE Joint Genome Institute
854	*Aspergillus sclerotiicarbonarius*	Eurotio	Asco	38	57	12	35	142	GCA_003184635.1	DOE Joint Genome Institute
855	*Ciborinia camelliae*	Leotio	Asco	42	48	14	38	142	GCA_001247705.1	Massey University
856	*Monilinia polystroma*	Leotio	Asco	40	51	17	34	142	GCA_002909645.1	United States Department of Agriculture
857	*Gymnopus androsaceus*	Agarico	Basidio	34	27	26	55	142	GCA_009733575.1	DOE Joint Genome Institute
858	*Aspergillus persii*	Eurotio	Asco	42	44	20	35	141	GCA_002215965.1	National Institute of Biological Resources
859	*Aspergillus fumigatus*	Eurotio	Asco	40	57	25	19	141	GCA_005768625.2	Nanyang Technological University
860	*Aspergillus carbonarius*	Eurotio	Asco	36	58	12	35	141	GCA_001990825.1	DOE Joint Genome Institute
861	*Aureobasidium sp. P6*	Dothideo	Asco	37	48	26	30	141	GCA_003992365.1	Ocean University of China
862	*Serendipita vermifera*	Agarico	Basidio	50	23	34	34	141	GCA_003073295.1	DOE Joint Genome Institute
863	*Penicillium sp. SPG-F1*	Eurotio	Asco	46	46	18	30	140	GCA_003800495.1	Texas A&M University Corpus Christi
864	*Aspergillus sclerotioniger*	Eurotio	Asco	38	56	12	34	140	GCA_003184525.1	DOE Joint Genome Institute
865	*Coprinopsis cinerea*	Agarico	Basidio	46	9	33	52	140	GCA_000182895.1	Broad Institute
866	*Lignosus rhinocerotis*	Agarico	Basidio	40	20	20	60	140	GCA_000743315.1	University of Malaya
867	*Paradendryphiella salina*	Dothideo	Asco	40	40	25	35	140	GCA_900634815.1	DTU Bioengineering
868	*Patellaria atrata*	Dothideo	Asco	50	23	29	38	140	GCA_010093705.1	DOE Joint Genome Institute
869	*Pleurotus tuoliensis*	Agarico	Basidio	48	26	14	52	140	GCA_003243755.1	Northeast Normal University
870	*Armillaria fuscipes*	Agarico	Basidio	40	28	18	54	140	GCA_001679825.1	Forestry and Agricultural Biotechnology Institute, University of Pretoria
871	*Penicillium sp. BW_162_3FA*	Eurotio	Asco	44	47	16	32	139	GCA_008931945.1	Tufts University
872	*Venturia pyrina*	Dothideo	Asco	33	48	14	44	139	GCA_000738655.1	La Trobe University Department of Botany
873	*Neurospora pannonica*	Sordario	Asco	43	25	37	34	139	GCA_009805235.1	University of California, Berkeley
874	*Decorospora gaudefroyi*	Dothideo	Asco	44	27	32	36	139	GCA_010015605.1	DOE Joint Genome Institute
875	*Pleurotus ostreatoroseus*	Agarico	Basidio	43	18	22	56	139	GCA_005298045.1	Amazonian macromicets: knowing their diversity and evaluating their biotechnological potential in a sustainable way
876	*Irpex lacteus*	Agarico	Basidio	46	23	22	48	139	GCA_001986395.2	Anhui university
877	*Penicillium biforme*	Eurotio	Asco	43	47	16	32	138	GCA_000577785.1	INRA-LIPM
878	*Venturia aucupariae*	Dothideo	Asco	33	49	14	42	138	GCA_003693225.1	INRA

879	*Neurospora sp. FGSC 6877*	Sordario	Asco	41	24	36	37	138	GCA_009805295.1	University of California, Berkeley
880	*Morchella sextelata*	Pezizo	Asco	41	54	19	24	138	GCA_009741755.1	Inner Mongolia International Mongolian Hospital
881	*Morchella importuna*	Pezizo	Asco	42	52	20	24	138	GCA_003444635.1	Huazhong Agricultural University
882	*Penicillium solitum*	Eurotio	Asco	43	48	18	28	137	GCA_000952775.2	JCVI
883	*Monilinia aucupariae*	Leotio	Asco	40	47	18	32	137	GCA_002162555.1	University of Toronto Mississauga
884	*Lyophyllum decastes*	Agarico	Basidio	40	22	16	59	137	GCA_001950495.1	RIKEN Center for Life Science Technologies, Division of Genomic Technologies
885	*Penicillium flavigenum*	Eurotio	Asco	40	46	18	32	136	GCA_002072365.1	Chalmers University of Technology
886	*Cercospora cf. sigesbeckiae*	Dothideo	Asco	39	35	22	40	136	GCA_005356805.1	University of Arkansas
887	*Cercospora beticola*	Dothideo	Asco	38	36	22	40	136	GCA_003370525.1	University of Southern Queensland
888	*Hypoxylon fragiforme*	Sordario	Asco	40	31	31	34	136	GCA_902806515.1	CEBITEC
889	*Flammulina velutipes*	Agarico	Basidio	43	24	25	44	136	GCA_011800155.1	Fujian Agriculture and Forestry University
890	*Aspergillus ellipticus*	Eurotio	Asco	36	58	14	28	136	GCA_003184645.1	DOE Joint Genome Institute
891	*Thermothelomyces thermophilus*	Sordario	Asco	40	32	36	28	136	GCA_000226095.1	DOE Joint Genome Institute
892	*Lentinus polychrous*	Agarico	Basidio	40	20	22	54	136	GCA_000787475.1	none
893	*Morchella conica*	Pezizo	Asco	40	52	20	24	136	GCA_003790465.1	DOE Joint Genome Institute
894	*Ascobolus immersus*	Pezizo	Asco	50	12	38	36	136	GCA_003788565.2	DOE Joint Genome Institute
895	*Gymnopilus dilepis*	Agarico	Basidio	42	12	20	62	136	GCA_002938385.1	Ohio State University
896	*Pleurotus eryngii*	Agarico	Basidio	48	22	14	52	136	GCA_001717165.1	Shanghai Academy of Agricultural Sciences
897	*Venturia inaequalis*	Dothideo	Asco	33	46	12	44	135	GCA_003689225.1	INRA
898	*Trametes sanguinea*	Agarico	Basidio	42	18	22	53	135	GCA_008973685.1	School of Bioscience and Technology
899	*Lachnellula subtilissima*	Leotio	Asco	41	42	16	36	135	GCA_007821545.1	Canadian Food Inspection Agency (CFIA)
900	*Sclerotium cepivorum*	0	Asco	39	44	18	34	135	GCA_002162485.1	University of Toronto Mississauga
901	*Myriosclerotinia duriaeana*	Leotio	Asco	40	41	18	36	135	GCA_002162615.1	University of Toronto Mississauga
902	*Stropharia rugosoannulata*	Agarico	Basidio	44	12	17	62	135	GCA_003314255.1	Kunming University of Science and Technology
903	*Leucoagaricus sp. SymC.cos*	Agarico	Basidio	37	15	21	62	135	GCA_001563735.1	BGI
904	*Phlebia tremellosa*	Agarico	Basidio	44	11	23	56	134	GCA_011032875.1	Ningxia University
905	*Pleurotus tuber-regium*	Agarico	Basidio	45	14	14	61	134	GCA_014058305.1	Huazhong Agricultural University
906	*Talaromyces islandicus*	Eurotio	Asco	32	46	24	31	133	GCA_000985935.1	CEBITEC

907	*Cercospora kikuchii*	Dothideo	Asco	38	34	22	39	133	GCA_009193115.1	School of Agriculture of the University of Buenos Aires
908	*Cercospora cf. flagellaris*	Dothideo	Asco	39	34	22	38	133	GCA_005356885.1	University of Arkansas
909	*Venturia nashicola*	Dothideo	Asco	30	48	15	40	133	GCA_004522665.1	POSTECH
910	*Penicillium polonicum*	Eurotio	Asco	42	44	17	30	133	GCA_013466175.1	National Institute of Agricultural Sciences, RDA
911	*Aspergillus sclerotiorum*	Eurotio	Asco	41	42	20	30	133	GCA_000530345.1	SVSB
912	*Ciboria shiraiana*	Leotio	Asco	37	46	20	30	133	GCA_008122225.1	Southwest University
913	*Aspergillus ibericus*	Eurotio	Asco	34	56	11	32	133	GCA_003184845.1	DOE Joint Genome Institute
914	*Myriosclerotinia scirpicola*	Leotio	Asco	39	41	16	37	133	GCA_002162505.1	University of Toronto Mississauga
915	*Pholiota adiposa*	Agarico	Basidio	46	11	20	56	133	GCA_009935795.1	Dezhou University
916	*Trichoderma virens*	Sordario	Asco	42	28	36	26	132	GCA_001835465.1	Bhabha Atomic Research Centre
917	*Cercospora canescens*	Dothideo	Asco	37	36	19	40	132	GCA_000347735.1	Banaras Hindu University
918	*Cercospora nicotianae*	Dothideo	Asco	38	34	22	38	132	GCA_002994015.1	VIB
919	*Penicillium camemberti*	Eurotio	Asco	40	46	16	30	132	GCA_000513335.1	INRA-LIPM
920	*Lachnellula hyalina*	Leotio	Asco	41	43	12	36	132	GCA_007821495.1	Canadian Food Inspection Agency (CFIA)
921	*Neurospora sp. FGSC 26632*	Sordario	Asco	38	24	36	34	132	GCA_009804965.1	University of California, Berkeley
922	*Neurospora cerealis*	Sordario	Asco	41	22	33	36	132	GCA_009806135.1	University of California, Berkeley
923	*Hypholoma sublateritium*	Agarico	Basidio	38	15	19	60	132	GCA_000827495.1	JGI
924	*Cercospora citrullina*	Dothideo	Asco	38	34	22	37	131	GCA_013365195.1	Banaras Hindu University
925	*Penicillium vulpinum*	Eurotio	Asco	42	37	15	37	131	GCA_002072255.1	Chalmers University of Technology
926	*Neurospora sp. FGSC 26635*	Sordario	Asco	36	24	36	35	131	GCA_009804975.1	University of California, Berkeley
927	*Sclerotinia sclerotiorum*	Leotio	Asco	36	45	20	30	131	GCA_001857865.1	Curtin University
928	*Trametes cinnabarina*	Agarico	Basidio	40	16	24	51	131	GCA_000787515.1	Aalborg University
929	*Sordaria macrospora*	Sordario	Asco	40	20	34	36	130	GCA_008692325.1	Ruhr-University Bochum
930	*Ascocoryne sarcoides*	Leotio	Asco	44	45	20	21	130	GCA_000328965.1	Yale University
931	*Tricholomella constricta*	Agarico	Basidio	36	26	16	52	130	GCA_013368375.1	Lund University
932	*Ploettnerulaceae sp. D365*	Leotio	Asco	41	38	18	33	130	GCA_003988805.1	Manaaki Whenua Landcare Research
933	*Dactylella cylindrospora*	Orbilio	Asco	40	44	20	26	130	GCA_012184295.1	Laboratory for Conservation and Utilization of Bio-Resources and Key Laboratory for Microbial Diversity in Southwest China, Ministry of Education
934	*Coprinopsis marcescibilis*	Agarico	Basidio	38	7	27	58	130	GCA_004369085.1	DOE Joint Genome Institute
935	*Rhizophlyctis rosea*	Chytridio	Chytridio	39	25	42	24	130	GCA_002214945.1	DTU

936	*Penicillium sp. BW_MB*	Eurotio	Asco	38	42	21	28	129	GCA_008931935.1	Tufts University
937	*Penicillium freii*	Eurotio	Asco	37	44	20	28	129	GCA_001513925.1	Agriculture and Agri-Food Canada
938	*Neurospora terricola*	Sordario	Asco	39	22	34	34	129	GCA_009805285.1	University of California, Berkeley
939	*Neurospora sp. CHS-2018c*	Sordario	Asco	38	21	34	36	129	GCA_009806235.1	University of California, Berkeley
940	*Trametes coccinea*	Agarico	Basidio	38	18	23	50	129	GCA_002092935.1	DOE Joint Genome Institute
941	*Cercospora berteroae*	Dothideo	Asco	38	33	20	37	128	GCA_002933655.1	VIB
942	*Venturia asperata*	Dothideo	Asco	30	44	14	40	128	GCA_003689065.1	INRA
943	*Neurospora sp. FGSC 26631*	Sordario	Asco	37	23	33	35	128	GCA_009805105.1	University of California, Berkeley
944	*Venturia carpophila*	Dothideo	Asco	35	44	11	38	128	GCA_001990985.1	USDA-ARS
945	*Neurospora sp. FGSC 26628*	Sordario	Asco	39	22	30	37	128	GCA_009805345.1	University of California, Berkeley
946	*Neurospora sp. FGSC 26638*	Sordario	Asco	36	23	34	35	128	GCA_009804825.1	University of California, Berkeley
947	*Ampelomyces quisqualis*	Dothideo	Asco	39	34	25	30	128	GCA_010094095.1	DOE Joint Genome Institute
948	*Sphaceloma murrayae*	Dothideo	Asco	34	46	21	27	128	GCA_002895985.1	Nanjing Forestry University
949	*Volvariella volvacea*	Agarico	Basidio	45	17	26	40	128	GCA_001691835.3	Harvard University
950	*Neurospora sp. FGSC 26637*	Sordario	Asco	37	21	34	35	127	GCA_009804845.1	University of California, Berkeley
951	*Neurospora sp. FGSC 26636*	Sordario	Asco	37	23	32	35	127	GCA_009804955.1	University of California, Berkeley
952	*Sclerotinia borealis*	Leotio	Asco	38	45	12	32	127	GCA_000503235.1	Centre Bioengineering RAS
953	*Venturia effusa*	Dothideo	Asco	32	42	13	40	127	GCA_001901625.1	USDA-ARS
954	*Sclerotinia glacialis*	Leotio	Asco	37	43	17	30	127	GCA_002162575.1	University of Toronto Mississauga
955	*Neurospora sp. CHS-2018a*	Sordario	Asco	38	20	34	35	127	GCA_009802375.1	University of California, Berkeley
956	*Westerdykella ornata*	Dothideo	Asco	40	21	33	33	127	GCA_010094085.1	DOE Joint Genome Institute
957	*Arthrobotrys oligospora*	Orbilio	Asco	40	42	22	23	127	GCA_004768765.1	Academia Sinica
958	*Hypsizygus marmoreus*	Agarico	Basidio	40	17	14	56	127	GCA_013433165.1	Fujian Agriculture and Forestry University
959	*Tothia fuscella*	Dothideo	Asco	34	36	14	42	126	GCA_010583015.1	DOE Joint Genome Institute
960	*Penicillium sp. MA 6040*	Eurotio	Asco	38	42	16	30	126	GCA_003138025.1	University of Natural Resources and Life Sciences, Vienna
961	*Penicillium chrysogenum*	Eurotio	Asco	38	42	16	30	126	GCA_000710275.1	Ruhr-Univeristaet Bochum
962	*Penicillium rubens*	Eurotio	Asco	38	42	16	30	126	GCA_902636305.1	IMPERIAL COLLEGE LONDON
963	*Neurospora sp. LNF1-2*	Sordario	Asco	35	23	33	35	126	GCA_009805125.1	University of California, Berkeley
964	*Neurospora sp. FGSC 26633*	Sordario	Asco	36	23	33	34	126	GCA_009804945.1	University of California, Berkeley

965	*Neurospora sp. FGSC 26623*	Sordario	Asco	37	24	31	34	126	GCA_009805935.1	University of California, Berkeley
966	*Heterobasidion parviporum*	Agarico	Basidio	36	22	12	56	126	GCA_002994785.1	University of Helsinki
967	*Neurospora sp. FGSC 26629*	Sordario	Asco	35	23	33	34	125	GCA_009805155.1	University of California, Berkeley
968	*Neurospora sp. LNF1-1*	Sordario	Asco	35	23	33	34	125	GCA_009805225.1	University of California, Berkeley
969	*Neurospora sp. FGSC 26630*	Sordario	Asco	35	23	33	34	125	GCA_009805085.1	University of California, Berkeley
970	*Neurospora sp. FGSC 26627*	Sordario	Asco	35	23	33	34	125	GCA_009805365.1	University of California, Berkeley
971	*Neurospora sp. FGSC 26634*	Sordario	Asco	37	22	30	36	125	GCA_009804985.1	University of California, Berkeley
972	*Neurospora sp. FGSC 26625*	Sordario	Asco	38	22	30	35	125	GCA_009805435.1	University of California, Berkeley
973	*Neurospora africana*	Sordario	Asco	36	21	34	34	125	GCA_000604205.2	EBC
974	*Lyophyllum shimeji*	Agarico	Basidio	34	16	14	61	125	GCA_001950515.1	RIKEN Center for Life Science Technologies, Division of Genomic Technologies
975	*Sporormia fimetaria*	Dothideo	Asco	40	14	37	34	125	GCA_010093795.1	DOE Joint Genome Institute
976	*Fragosphaeria purpurea*	Sordario	Asco	38	22	32	32	124	GCA_002778095.1	University of Montana
977	*Penicillium sp. HKF2*	Eurotio	Asco	38	40	16	30	124	GCA_002000375.1	CSIR-NEERI
978	*Penicillium verrucosum*	Eurotio	Asco	40	42	16	26	124	GCA_000970515.2	Max Rubner-Institut
979	*Ramularia coccinea*	Dothideo	Asco	41	22	35	26	124	GCA_013461505.1	Yancheng Institute of Technology
980	*Sporothrix globosa*	Sordario	Asco	35	31	28	30	124	GCA_001630435.1	University of Messina
981	*Neurospora sp. CHS-2018b*	Sordario	Asco	36	19	34	35	124	GCA_009806015.1	University of California, Berkeley
982	*Monilinia fructigena*	Leotio	Asco	34	46	14	30	124	GCA_003260565.1	Rita Milvia De Miccolis Angelini's shared submissions
983	*Macrocybe gigantea*	Agarico	Basidio	36	20	14	54	124	GCA_011319805.1	Yunnan Agricultural University
984	*Amorphotheca resinae*	Leotio	Asco	25	39	14	45	123	GCA_001270345.1	East China University of Science and Technology
985	*Sporothrix insectorum*	Sordario	Asco	37	21	32	33	123	GCA_001636815.1	Shanghai Institutes for Biological Sciences, CAS
986	*Pseudocercospora musae*	Dothideo	Asco	34	39	16	34	123	GCA_001578225.1	University of California Davis
987	*Trichothecium roseum*	Sordario	Asco	37	36	26	24	123	GCA_003012185.1	USDA, ARS, NCAUR
988	*Penicillium nordicum*	Eurotio	Asco	40	42	16	25	123	GCA_001278595.1	Agriculture and Agri-Food Canada
989	*Penicillium coprophilum*	Eurotio	Asco	41	32	18	32	123	GCA_002072405.1	Chalmers University of Technology
990	*Sporothrix schenckii*	Sordario	Asco	38	27	28	30	123	GCA_000961545.1	LNCC
991	*Agaricales sp. HM26-F1*	Agarico	Basidio	31	26	16	50	123	GCA_002718315.1	Shanghai Academy of Agricultural Sciences

992	*Polytolypa hystricis*	Eurotio	Asco	41	24	32	26	123	GCA_002573605.1	Broad Institute
993	*Lachnellula occidentalis*	Leotio	Asco	38	36	14	34	122	GCA_007821535.1	Canadian Food Inspection Agency (CFIA)
994	*Neurospora sp. FGSC 26626*	Sordario	Asco	37	22	28	35	122	GCA_009805385.1	University of California, Berkeley
995	*Pseudovirgaria hyperparasitica*	Dothideo	Asco	28	36	24	34	122	GCA_010093815.1	DOE Joint Genome Institute
996	*Arthrobotrys entomopaga*	Orbilio	Asco	42	30	25	25	122	GCA_012184315.1	Laboratory for Conservation and Utilization of Bio-Resources and Key Laboratory for Microbial Diversity in Southwest China, Ministry of Education
997	*Myriosclerotinia curreyana*	Leotio	Asco	38	36	18	30	122	GCA_002162495.1	University of Toronto Mississauga
998	*Arthrobotrys flagrans*	Orbilio	Asco	36	42	20	24	122	GCA_004000055.1	Institute for Applied Biosciences
999	*Neurospora sp. FGSC 26624*	Sordario	Asco	35	22	30	34	121	GCA_009805485.1	University of California, Berkeley
1000	*Lophium mytilinum*	Dothideo	Asco	44	20	15	42	121	GCA_010093605.1	DOE Joint Genome Institute
1001	*Hericium erinaceus*	Agarico	Basidio	42	15	16	48	121	GCA_006506795.2	Institute of Bast Fiber Crops, Chinese Academy of Agricultural Sciences
1002	*Nothophaeocryptopus gaeumannii*	Dothideo	Asco	37	34	8	41	120	GCA_002116385.1	Canada's Michael Smith Genome Sciences Centre
1003	*Penicillium sp. CF05*	Eurotio	Asco	34	44	15	27	120	GCA_002916455.1	INAIL-Research Area
1004	*Talaromyces funiculosus*	Eurotio	Asco	29	36	28	27	120	GCA_004299765.1	Shandong Agricultural University
1005	*Penicillium griseofulvum*	Eurotio	Asco	40	33	19	28	120	GCA_001561935.1	University of Turin
1006	*Raffaelea lauricola*	Sordario	Asco	40	28	22	30	120	GCA_014183025.1	University of Florida
1007	*Sporothrix brasiliensis*	Sordario	Asco	37	26	28	29	120	GCA_000820605.1	LNCC
1008	*Dentipellis sp. KUC8613*	Agarico	Basidio	41	17	16	46	120	GCA_002286715.1	Korea University
1009	*Neurospora sublineolata*	Sordario	Asco	33	22	33	32	120	GCA_000604185.2	EBC
1010	*Penicillium oxalicum*	Eurotio	Asco	32	47	26	15	120	GCA_005546515.1	Nanyang Technological University
1011	*Phlebiopsis gigantea*	Agarico	Basidio	37	21	17	45	120	GCA_000832265.1	DOE Joint Genome Institute
1012	*Heterobasidion irregulare*	Agarico	Basidio	34	23	11	52	120	GCA_000320585.2	JGI
1013	*Ramularia endophylla*	Dothideo	Asco	38	38	13	30	119	GCA_002116395.1	Canada's Michael Smith Genome Sciences Centre
1014	*Pseudocercospora eumusae*	Dothideo	Asco	32	42	16	29	119	GCA_001578235.1	University of California Davis
1015	*Neurospora tetraspora*	Sordario	Asco	34	23	30	32	119	GCA_009806155.1	University of California, Berkeley
1016	*Phyllosticta capitalensis*	Dothideo	Asco	32	38	10	39	119	GCA_001604925.1	Citrus Research and Education Center, University of Florida
1017	*Heterobasidion annosum*	Agarico	Basidio	32	24	10	53	119	GCA_000633895.1	Seoul National University

1018	*Claviceps aff. purpurea*	Sordario	Asco	18	58	28	14	118	GCA_004016085.1	Agriculture and Agri-Food Canada
1019	*Penicillium nalgiovense*	Eurotio	Asco	34	43	15	26	118	GCA_000577395.2	INRA-LIPM
1020	*Hyphodiscus sp. D1413*	Leotio	Asco	34	34	20	30	118	GCA_003988895.1	Manaaki Whenua Landcare Research
1021	*Trichocladium griseum*	Sordario	Asco	39	17	35	27	118	GCA_011316235.1	Embrapa Agroenergia
1022	*Cercospora sojina*	Dothideo	Asco	33	32	20	32	117	GCA_002534735.1	Chinese Academy of Sciences
1023	*Gelatoporia subvermispora*	Agarico	Basidio	31	14	26	46	117	GCA_000320605.2	DOE Joint Genome Institute
1024	*Macrocystidia cucumis*	Agarico	Basidio	36	12	13	56	117	GCA_001179725.1	Royal Botanic Gardens, Kew
1025	*Aspergillus saccharolyticus*	Eurotio	Asco	32	45	13	26	116	GCA_003184585.1	DOE Joint Genome Institute
1026	*Cyanodermella asteris*	Lecanoro	Asco	34	34	18	30	116	GCA_900618795.1	CEBITEC
1027	*Gloeostereum incarnatum*	Agarico	Basidio	37	18	16	45	116	GCA_010588315.1	Jilin Agricultural University
1028	*Hericium alpestre*	Agarico	Basidio	40	16	18	42	116	GCA_004681135.1	IHI Zittau / TU Dresden
1029	*Leucocalocybe mongolica*	Agarico	Basidio	34	22	16	44	116	GCA_013420905.1	Jilin Agricultural University
1030	*Trichoderma sp. IMV 00454*	Sordario	Asco	38	22	33	22	115	GCA_001931985.1	Jet Propulsion Laboratory, California Institute of Technology
1031	*Talaromyces stipitatus*	Eurotio	Asco	28	31	27	29	115	GCA_000003125.1	J. Craig Venter Institute
1032	*Dentipellis fragilis*	Agarico	Basidio	40	16	16	43	115	GCA_004679275.1	IHI Zittau / TU Dresden
1033	*Morchella sp. a71*	Pezizo	Asco	39	41	15	20	115	GCA_013407065.1	Chongqing Normal University
1034	*Raffaelea arxii*	Sordario	Asco	37	26	14	36	113	GCA_002778165.1	University of Montana
1035	*Teratosphaeria zuluensis*	Dothideo	Asco	30	35	13	35	113	GCA_007113905.1	University of Pretoria
1036	*Sistotremastrum suecicum*	Agarico	Basidio	38	3	18	54	113	GCA_001632355.1	DOE Joint Genome Institute
1037	*Trichoderma hamatum*	Sordario	Asco	34	30	28	20	112	GCA_000331835.2	University of Exeter
1038	*Trichoderma guizhouense*	Sordario	Asco	37	22	32	21	112	GCA_002022785.1	Nanjing Agricultural University
1039	*Coniophora puteana*	Agarico	Basidio	40	24	14	34	112	GCA_000271625.1	DOE Joint Genome Institute
1040	*Acidomyces sp. 'richmondensis'*	Dothideo	Asco	25	46	20	21	112	GCA_003545705.1	University of Massachusetts
1041	*Aspergillus heteromorphus*	Eurotio	Asco	31	44	12	25	112	GCA_003184545.1	DOE Joint Genome Institute
1042	*Microcyclosporella mali*	Dothideo	Asco	30	30	20	32	112	GCA_002785985.1	Northwest A&F University
1043	*Bondarzewia mesenterica*	Agarico	Basidio	40	10	10	52	112	GCA_004802705.1	IHI Zittau / TU Dresden
1044	*Phyllosticta citricarpa*	Dothideo	Asco	29	36	10	36	111	GCA_000382785.1	Zhejiang university
1045	*Serendipita indica*	Agarico	Basidio	28	19	36	28	111	GCA_000313545.1	Institute of Bioinformatics and Systems Biology, Helmholtz Zentrum München, Neuherberg, Germany
1046	*Trichoderma atrobrunneum*	Sordario	Asco	37	22	30	21	110	GCA_003439915.1	Institute of Sciences of Food Production (ISPA)
1047	*Trichoderma lentiforme*	Sordario	Asco	37	20	32	21	110	GCA_011066345.1	Embrapa Agroenergia
1048	*Mycosphaerella arachidis*	Dothideo	Asco	30	34	14	32	110	GCA_001297265.1	USDA
1049	*Cladophialophora immunda*	Eurotio	Asco	28	15	15	52	110	GCA_000835495.1	Broad Institute
1050	*Hydnomerulius pinastri*	Agarico	Basidio	44	18	14	34	110	GCA_000827185.1	DOE Joint Genome Institute
1051	*Dothistroma pini*	Dothideo	Asco	31	30	16	32	109	GCA_002116355.1	Canada's Michael Smith Genome Sciences Centre

1052	*Saitozyma sp. JCM 24511*	Tremello	Basidio	27	48	18	16	109	GCA_001600855.1	RIKEN Center for Life Science Technologies, Division of Genomic Technologies
1053	*Saitozyma podzolica*	Tremello	Basidio	28	46	17	18	109	GCA_003942215.1	Karlsruhe Institute of Technology
1054	*Porodaedalea pini*	Agarico	Basidio	26	22	14	47	109	GCA_002794775.1	Academia Sinica
1055	*Obba rivulosa*	Agarico	Basidio	30	12	23	44	109	GCA_001687445.1	DOE Joint Genome Institute
1056	*Grifola frondosa*	Agarico	Basidio	30	13	14	52	109	GCA_001683735.1	Korea University
1057	*Psilocybe cyanescens*	Agarico	Basidio	32	8	22	47	109	GCA_002938375.1	Ohio State University
1058	*Rickenella mellea*	Agarico	Basidio	29	23	2	55	109	GCA_004355085.1	DOE Joint Genome Institute
1059	*Penicillium sp. OUCMDZ-019*	Eurotio	Asco	32	33	14	29	108	GCA_011750695.1	ocean university of China
1060	*Mycosphaerella populi*	Dothideo	Asco	28	37	13	30	108	GCA_002153405.1	Canada's Michael Smith Genome Sciences Centre
1061	*Acremonium chrysogenum*	Sordario	Asco	38	24	25	21	108	GCA_000769265.1	Ruhr-Universitaet Bochum
1062	*Pseudonectria buxi*	Sordario	Asco	34	44	12	18	108	GCA_003693545.1	USDA-ARS
1063	*Marssonina coronariae*	Leotio	Asco	25	43	11	29	108	GCA_002204255.1	Nanjing Forestry University
1064	*Phyllosticta citriasiana*	Dothideo	Asco	29	34	10	35	108	GCA_009193405.1	Zhejiang University
1065	*Collybia sp. MG36*	Agarico	Basidio	35	14	8	51	108	GCA_003313185.1	Kunming University of Science and Technology
1066	*Cercospora zeae-maydis*	Dothideo	Asco	32	26	18	31	107	GCA_010093985.1	JGI
1067	*Teratosphaeria gauchensis*	Dothideo	Asco	29	36	12	30	107	GCA_007113925.1	University of Pretoria
1068	*Marssonina rosae*	Leotio	Asco	25	42	10	30	107	GCA_011750715.1	Nanjing Forestry University
1069	*Botryobasidium botryosum*	Agarico	Basidio	42	9	25	30	106	GCA_000697705.1	JGI
1070	*Pseudonectria foliicola*	Sordario	Asco	32	46	12	16	106	GCA_002911195.1	USDA
1071	*Coprinopsis strossmayeri*	Agarico	Basidio	34	6	26	40	106	GCA_900156845.1	UNIVERSITY OF BRISTOL
1072	*Trichoderma harzianum*	Sordario	Asco	36	19	30	20	105	GCA_010015525.1	The University of Adelaide
1073	*Talaromyces borbonicus*	Eurotio	Asco	22	40	29	14	105	GCA_002916415.1	University of Naples "Federico II"
1074	*Aspergillus sclerotialis*	Eurotio	Asco	35	30	16	24	105	GCA_003589665.1	University of Natural Resources and Life Sciences, Vienna
1075	*Byssochlamys sp. AF001*	Eurotio	Asco	24	28	11	42	105	GCA_002914405.1	University of Oklahoma
1076	*Sodiomyces alkalinus*	Sordario	Asco	39	16	24	26	105	GCA_003711515.1	DOE Joint Genome Institute
1077	*Pluteus cervinus*	Agarico	Basidio	32	8	13	52	105	GCA_004369065.1	DOE Joint Genome Institute
1078	*Sistotremastrum niveocremeum*	Agarico	Basidio	34	3	18	50	105	GCA_001630475.1	DOE Joint Genome Institute
1079	*Trichoderma koningiopsis*	Sordario	Asco	30	25	29	20	104	GCA_002246955.1	INBIOMIS
1080	*Passalora sequoiae*	Dothideo	Asco	29	28	12	35	104	GCA_013248845.1	USDA
1081	*Cutaneotrichosporon mucoides*	Tremello	Basidio	27	26	20	31	104	GCA_003116955.1	RIKEN Center for Life Science Technologies, Division of Genomic Technologies
1082	*Schizopora paradoxa*	Agarico	Basidio	31	8	14	51	104	GCA_001020605.1	DOE Joint Genome Institute
1083	*Jaapia argillacea*	Agarico	Basidio	48	10	16	30	104	GCA_000697665.1	DOE Joint Genome Institute
1084	*Trichoderma gamsii*	Sordario	Asco	29	25	30	19	103	GCA_002894205.1	CSIRO
1085	*Cercospora zeina*	Dothideo	Asco	30	28	17	28	103	GCA_002844615.1	University of Pretoria

1086	*Chlorencoelia torta*	Leotio	Asco	38	20	18	27	103	GCA_003988815.1	Manaaki Whenua Landcare Research
1087	*Delphinella strobiligena*	Dothideo	Asco	25	34	8	36	103	GCA_009982845.1	DOE Joint Genome Institute
1088	*Antrodiella citrinella*	Agarico	Basidio	33	10	20	40	103	GCA_004802725.1	IHI Zittau / TU Dresden
1089	*Trichothecium sympodiale*	Sordario	Asco	32	20	29	21	102	GCA_003012115.1	USDA, ARS, NCAUR
1090	*Aspergillus clavatus*	Eurotio	Asco	34	28	21	19	102	GCA_000002715.1	J. Craig Venter Institute
1091	*Rasamsonia emersonii*	Eurotio	Asco	28	32	16	26	102	GCA_000968595.1	DSM Bio-based Products & Services B.V.
1092	*Neurospora tetrasperma*	Sordario	Asco	29	22	25	26	102	GCA_000213195.1	US DOE Joint Genome Institute (JGI-PGF)
1093	*Aspergillus ochraceoroseus*	Eurotio	Asco	30	34	18	20	102	GCA_002846915.2	DOE Joint Genome Institute
1094	*Coccinonectria pachysandricola*	Sordario	Asco	30	42	10	20	102	GCA_013283235.1	USDA-ARS
1095	*Aspergillus chevalieri*	Eurotio	Asco	32	28	12	30	102	GCA_001599875.1	RIKEN Center for Life Science Technologies, Division of Genomic Technologies
1096	*Neurospora crassa*	Sordario	Asco	27	23	25	26	101	GCA_007478085.1	Ronin Genetics
1097	*Ochroconis constricta*	Dothideo	Asco	32	18	9	42	101	GCA_000611715.1	UNIVERSITY OF MALAYA
1098	*Thermoascaceae sp. COH1141*	Eurotio	Asco	26	25	12	38	101	GCA_003123655.1	Jet Propulsion Laboratory, California Institute of Technology
1099	*Aspergillus rambellii*	Eurotio	Asco	29	34	17	21	101	GCA_000986645.1	USDA-ARS-SRRC
1100	*Lineolata rhizophorae*	Dothideo	Asco	35	10	28	28	101	GCA_010093515.1	DOE Joint Genome Institute
1101	*Calcarisporium arbuscula*	Sordario	Asco	32	22	19	27	100	GCA_009828645.1	Zhejiang University
1102	*Trichoderma erinaceum*	Sordario	Asco	29	25	29	17	100	GCA_013365115.1	None
1103	*Ramularia collo-cygni*	Dothideo	Asco	36	20	16	28	100	GCA_900074925.1	Technische Universitat Munchen - WZW
1104	*Talaromyces marneffei*	Eurotio	Asco	24	28	18	30	100	GCA_009650675.1	Broad Institute
1105	*Drechslerella brochopaga*	Orbilio	Asco	31	32	16	21	100	GCA_012184305.1	Laboratory for Conservation and Utilization of Bio-Resources and Key Laboratory for Microbial Diversity in Southwest China, Ministry of Education
1106	*Gymnopus luxurians*	Agarico	Basidio	30	22	14	34	100	GCA_000827265.1	DOE JGI
1107	*Trichoderma atroviride*	Sordario	Asco	28	26	29	16	99	GCA_001599035.1	RIKEN Center for Life Science Technologies, Division of Genomic Technologies
1108	*[Talaromyces] leycettanus*	Eurotio	Asco	28	32	23	16	99	GCA_000787455.1	none
1109	*Trichothecium ovalisporum*	Sordario	Asco	33	17	29	20	99	GCA_003012195.1	USDA, ARS, NCAUR
1110	*Saccharata proteae*	Dothideo	Asco	30	28	10	31	99	GCA_010015785.1	DOE Joint Genome Institute
1111	*Fomitiporia mediterranea*	Agarico	Basidio	30	13	10	46	99	GCA_000271605.1	JGI
1112	*Trichoderma viride*	Sordario	Asco	31	18	30	19	98	GCA_007896495.1	Institute of Biology, Shnaodong Academy of Science, China
1113	*Trichoderma asperellum*	Sordario	Asco	30	22	28	18	98	GCA_013423425.1	Chubu university

1114	*Cantharellus appalachiensis*	Agarico	Basidio	27	24	29	18	98	GCA_003314335.1	Kunming University of Science and Technology
1115	*Chlorociboria aeruginascens*	Leotio	Asco	32	19	20	27	98	GCA_002276475.2	IHI Zittau / TU Dresden
1116	*Heliocybe sulcata*	Agarico	Basidio	30	20	18	30	98	GCA_004369045.1	DOE Joint Genome Institute
1117	*Aspergillus tritici*	Eurotio	Asco	34	24	16	24	98	GCA_009812425.1	Universidad de Antioquia
1118	*Dothistroma septosporum*	Dothideo	Asco	28	21	14	34	97	GCA_002236755.2	Massey University
1119	*Neurospora discreta*	Sordario	Asco	27	21	23	26	97	GCA_009805215.1	University of California, Berkeley
1120	*Blumeriella jaapii*	Leotio	Asco	28	28	12	29	97	GCA_009599575.1	Michigan State University
1121	*Plicaturopsis crispa*	Agarico	Basidio	28	20	10	38	96	GCA_000827205.1	DOE Joint Genome Institute
1122	*Ophiostoma novo-ulmi*	Sordario	Asco	32	26	14	24	96	GCA_000317715.1	Association of Biomolecular Resource Facilities
1123	*Coniferiporia sulphurascens*	Agarico	Basidio	26	14	12	44	96	GCA_002794785.1	Biodiversity Research Center, Academia Sinica
1124	*Pochonia chlamydosporia*	Sordario	Asco	30	22	16	27	95	GCA_001653235.2	The Institute of Vegetables and Flowers CAAS
1125	*Cryomyces minteri*	Dothideo	Asco	22	9	12	52	95	GCA_005059845.1	University of California, Riverside
1126	*Fomitopsis palustris*	Agarico	Basidio	23	30	18	24	95	GCA_001937815.1	National Institute of Forest Science
1127	*Byssochlamys nivea*	Eurotio	Asco	25	26	11	33	95	GCA_003116535.1	Cornell University
1128	*Aspergillus campestris*	Eurotio	Asco	30	25	18	22	95	GCA_002847485.1	DOE Joint Genome Institute
1129	*Aulographum hederae*	Dothideo	Asco	22	26	14	32	94	GCA_010015705.1	DOE Joint Genome Institute
1130	*Penicillium italicum*	Eurotio	Asco	34	27	9	24	94	GCA_000769765.1	Center for Genomic Regulation (CRG)
1131	*Pseudophaeomoniella oleicola*	Eurotio	Asco	25	38	11	20	94	GCA_003868215.1	University of Bari "A. Moro"
1132	*Hortaea thailandica*	Dothideo	Asco	26	32	12	24	94	GCA_005059885.1	University of California, Riverside
1133	*Aspergillus taichungensis*	Eurotio	Asco	30	25	17	22	94	GCA_002850765.1	DOE Joint Genome Institute
1134	*Aspergillus coremiiformis*	Eurotio	Asco	27	38	10	19	94	GCA_009193565.1	DOE Joint Genome Institute
1135	*Chaetomium thermophilum*	Sordario	Asco	34	14	26	20	94	GCA_000221225.1	EMBL
1136	*Sarcomyxa edulis*	Agarico	Basidio	24	16	14	40	94	GCA_009761415.1	Engineering Research Center of Chinese Ministry of Education for Edible and Medicinal Fungi
1137	*Clitocybe nebularis*	Agarico	Basidio	28	16	12	38	94	GCA_900068955.1	Royal Botanic Gardens, Kew
1138	*Fomitopsis pinicola*	Agarico	Basidio	24	27	16	26	93	GCA_000344655.2	DOE Joint Genome Institute
1139	*Aspergillus cristatus*	Eurotio	Asco	28	24	11	30	93	GCA_001693355.1	Shandong Freda Pharmaceutical Group Corporation
1140	*Gymnopus confluens*	Agarico	Basidio	25	12	10	46	93	GCA_013368555.1	Lund University
1141	*Talaromyces atroroseus*	Eurotio	Asco	26	30	13	23	92	GCA_001907595.1	Technical University of Denmark
1142	*Talaromyces piceae*	Eurotio	Asco	22	32	18	20	92	GCA_001657655.1	Tianjin Institute of Biotechnology, CAS

1143	*Amesia nigricolor*	Sordario	Asco	30	20	20	22	92	GCA_004802645.1	Qingdao University
1144	*Teratosphaeria nubilosa*	Dothideo	Asco	23	33	11	25	92	GCA_010093825.1	DOE Joint Genome Institute
1145	*Penicillium digitatum*	Eurotio	Asco	30	32	10	20	92	GCA_012295545.1	Huazhong Agricultural University
1146	*Gloeophyllum trabeum*	Agarico	Basidio	26	24	16	26	92	GCA_000344685.1	JGI
1147	*Pholiota microspora*	Agarico	Basidio	29	10	11	42	92	GCA_003314615.1	Kunming University of Science and Technology
1148	*Lecanicillium fungicola*	Sordario	Asco	34	12	5	40	91	GCA_900169235.1	UNIVERSITY OF BRISTOL
1149	*Mytilinidion resinicola*	Dothideo	Asco	27	14	10	40	91	GCA_010093595.1	DOE Joint Genome Institute
1150	*Penicillium carneum*	Eurotio	Asco	32	25	8	26	91	GCA_000577495.1	INRA-LIPM
1151	*Cyphellophora europaea*	Eurotio	Asco	28	10	18	35	91	GCA_000365145.2	Broad Institute
1152	*Ophiostoma piceae*	Sordario	Asco	30	26	13	22	91	GCA_000410735.1	University of British Columbia
1153	*Choiromyces venosus*	Pezizo	Asco	29	30	10	22	91	GCA_003788595.2	DOE Joint Genome Institute
1154	*Aspergillus glaucus*	Eurotio	Asco	28	28	8	27	91	GCA_001890805.1	DOE Joint Genome Institute
1155	*Teratosphaeria pseudoeucalypti*	Dothideo	Asco	22	32	10	26	90	GCA_013403795.1	University of Pretoria
1156	*Penicillium roqueforti*	Eurotio	Asco	30	25	10	25	90	GCA_001599855.1	RIKEN Center for Life Science Technologies, Division of Genomic Technologies
1157	*Zymoseptoria pseudotritici*	Dothideo	Asco	30	24	16	20	90	GCA_000223685.2	University of Aarhus, Bioinformatics Research Center
1158	*Athelia rolfsii*	Agarico	Basidio	20	32	18	20	90	GCA_002940785.1	Southwest University
1159	*Termitomyces sp. MG145*	Agarico	Basidio	26	9	20	35	90	GCA_003313055.1	Kunming University of Science and Technology
1160	*Podosphaera xanthii*	Leotio	Asco	24	21	8	36	89	GCA_010015925.1	National Academy of Agricultural Science, Rural Development Adminstration
1161	*Dicyma pulvinata*	Sordario	Asco	26	30	13	20	89	GCA_006538405.1	National Agriculture and Food Research Organization
1162	*Lecanosticta acicola*	Dothideo	Asco	24	21	12	32	89	GCA_002441625.1	Mississippi State University
1163	*Zymoseptoria tritici*	Dothideo	Asco	30	23	14	22	89	GCA_900184105.1	ETH
1164	*Teratosphaeria destructans*	Dothideo	Asco	23	31	11	24	89	GCA_010367415.1	Forestry and Agricultural Biotechnology Institute (FABI), University of Pretoria
1165	*Zymoseptoria brevis*	Dothideo	Asco	30	24	15	20	89	GCA_000966595.1	Christian-Albrechts University of Kiel
1166	*Penicilliopsis zonata*	Eurotio	Asco	24	34	10	21	89	GCA_001890105.1	DOE Joint Genome Institute
1167	*Penicillium paneum*	Eurotio	Asco	30	24	10	24	88	GCA_000577715.1	INRA-LIPM
1168	*Exophiala xenobiotica*	Eurotio	Asco	20	19	13	36	88	GCA_000835505.1	Broad Institute
1169	*Baudoinia panamericana*	Dothideo	Asco	25	20	19	24	88	GCA_000338955.1	JGI
1170	*Termitomyces sp. J132*	Agarico	Basidio	26	8	22	32	88	GCA_001263195.1	BGI
1171	*Termitomyces sp. MG148*	Agarico	Basidio	27	17	15	29	88	GCA_003313785.1	Kunming University of Science and Technology
1172	*Aspergillus tanneri*	Eurotio	Asco	30	22	4	31	87	GCA_003426965.1	JCVI

1173	*Cladosporiaceae sp. IMV 00236*	Dothideo	Asco	20	26	10	31	87	GCA_001931875.2	Jet Propulsion Laboratory, California Institute of Technology
1174	*Epichloe uncinata*	Sordario	Asco	26	15	16	29	86	GCA_001043855.1	University of Kentucky
1175	*Trichoderma arundinaceum*	Sordario	Asco	32	14	22	18	86	GCA_003012105.1	USDA, ARS, NCAUR
1176	*Epichloe glyceriae*	Sordario	Asco	18	30	24	14	86	GCA_000225285.2	University of Kentucky, Dept of Plant Pathology
1177	*Trichoderma pleuroti*	Sordario	Asco	33	14	20	19	86	GCA_001721665.1	University of Pecs
1178	*Zymoseptoria ardabiliae*	Dothideo	Asco	30	20	16	20	86	GCA_000223765.2	University of Aarhus, Bioinformatics Research Center
1179	*Exutisphaerella laricina*	Dothideo	Asco	26	20	14	26	86	GCA_000504385.2	Canada's Michael Smith Genome Sciences Centre
1180	*Phialophora attinorum*	Eurotio	Asco	27	12	16	31	86	GCA_001299255.1	CBS-KNAW Fungal Biodiversity Centre
1181	*Trichodelitschia bisporula*	Dothideo	Asco	21	28	10	27	86	GCA_010356995.1	DOE Joint Genome Institute
1182	*Phialophora verrucosa*	Eurotio	Asco	22	14	14	36	86	GCA_002099365.1	Peking University First Hospital
1183	*Periglandula ipomoeae*	Sordario	Asco	26	23	18	18	85	GCA_000222875.2	University of Kentucky, Dept of Plant Pathology
1184	*Exophiala oligosperma*	Eurotio	Asco	20	10	13	42	85	GCA_000835515.1	Broad Institute
1185	*Exophiala lecanii-corni*	Eurotio	Asco	28	20	9	28	85	GCA_003955835.1	Naval Research Laboratory
1186	*Phlebia centrifuga*	Agarico	Basidio	26	7	14	38	85	GCA_001913855.2	CBS-KNAW Fungal Biodiversity Centre Institute of the Royal Netherlands Academy of Arts and Science
1187	*Gymnopilus junonius*	Agarico	Basidio	26	3	14	42	85	GCA_900068935.1	Royal Botanic Gardens, Kew
1188	*Myriangium duriaei*	Dothideo	Asco	22	22	14	26	84	GCA_010093895.1	DOE Joint Genome Institute
1189	*Apiotrichum porosum*	Tremello	Basidio	22	32	12	18	84	GCA_003942205.1	Karlsruhe Institute of Technology
1190	*Termitomyces sp. JCM 13351*	Agarico	Basidio	24	10	20	30	84	GCA_001972325.1	RIKEN Center for Life Science Technologies, Division of Genomic Technologies
1191	*Trichoderma brevicompactum*	Sordario	Asco	32	11	22	18	83	GCA_003012085.1	USDA, ARS, NCAUR
1192	*Raffaelea quercivora*	Sordario	Asco	27	24	8	24	83	GCA_002778125.1	University of Montana
1193	*Raffaelea quercus-mongolicae*	Sordario	Asco	28	21	8	26	83	GCA_002215975.1	Seoul National University
1194	*Cenococcum geophilum*	Dothideo	Asco	27	14	10	32	83	GCA_001692895.1	DOE Joint Genome Institute
1195	*Fonsecaea multimorphosa*	Eurotio	Asco	23	10	12	38	83	GCA_000836435.1	Broad Institute
1196	*Termitomyces eurrhizus*	Agarico	Basidio	25	8	14	36	83	GCA_003316525.1	Kunming University of Science and Technology
1197	*Knoxdaviesia capensis*	Sordario	Asco	32	6	21	23	82	GCA_001510575.1	University of Stellenbosch
1198	*Simplicillium aogashimaense*	Sordario	Asco	26	16	13	26	81	GCA_012273805.1	AgResearch
1199	*Fomitopsis rosea*	Agarico	Basidio	24	18	14	25	81	GCA_004679265.1	IHI Zittau / TU Dresden
1200	*Mycosphaerella sp. Ston1*	Dothideo	Asco	22	26	7	26	81	GCA_000504405.2	Canada's Michael Smith Genome Sciences Centre

1201	*Fusarium ventricosum*	Sordario	Asco	24	24	10	23	81	GCA_013623725.1	US Department of Agriculture, Agriculture Research Service
1202	*Cladophialophora psammophila*	Eurotio	Asco	20	10	11	40	81	GCA_000585535.1	Broad Institute
1203	*Laetiporus sulphureus*	Agarico	Basidio	26	15	14	26	81	GCA_001632365.1	DOE Joint Genome Institute
1204	*Geosmithia putterillii*	Sordario	Asco	28	18	10	24	80	GCA_900188575.1	UNIVERSITY OF NEW HAMPSHIRE
1205	*Arthonia radiata*	Arthonio	Asco	22	23	18	17	80	GCA_002989075.1	Stanford University
1206	*Thielaviopsis ethacetica*	Sordario	Asco	27	18	18	17	80	GCA_001599055.1	RIKEN Center for Life Science Technologies, Division of Genomic Technologies
1207	*Sanghuangporus sanghuang*	Agarico	Basidio	26	5	8	41	80	GCA_009806525.1	Anhui Provincial Key Laboratory of Microbial Pest Control
1208	*Lepiota venenata*	Agarico	Basidio	25	8	9	38	80	GCA_004296355.1	Kunming Institute of Botany
1209	*Lecanicillium psalliotae*	Sordario	Asco	26	16	9	28	79	GCA_002796755.1	Western Sydney University
1210	*Aeminium ludgeri*	Dothideo	Asco	22	30	16	11	79	GCA_004216415.1	Centre for Functional Diversity
1211	*Grosmannia penicillata*	Sordario	Asco	23	26	8	22	79	GCA_002778075.1	University of Montana
1212	*Leptographium lundbergii*	Sordario	Asco	24	28	6	21	79	GCA_001455505.1	University of Pretoria
1213	*Megacollybia marginata*	Agarico	Basidio	26	8	9	36	79	GCA_003313965.1	Kunming University of Science and Technology
1214	*Purpureocillium lilacinum*	Sordario	Asco	23	16	12	27	78	GCA_001468795.1	The Energy and Resources Institute
1215	*Moniliella sp. 'wahieum'*	Moniliello	Basidio	18	12	0	48	78	GCA_003971905.1	Kyungpook National University
1216	*Trichoderma sp. TW21990_1*	Sordario	Asco	26	12	24	16	78	GCA_010015515.1	The University of Adelaide
1217	*Exophiala aquamarina*	Eurotio	Asco	24	12	8	34	78	GCA_000709125.1	Broad Institute
1218	*Apiotrichum akiyoshidainum*	Tremello	Basidio	22	15	18	23	78	GCA_002973495.1	PROIMI - CONICET
1219	*Phyllachora maydis*	Sordario	Asco	19	24	14	21	78	GCA_011801745.1	Purdue University, Botany and Plant Pathology
1220	*Knoxdaviesia proteae*	Sordario	Asco	30	8	19	21	78	GCA_001510565.1	University of Stellenbosch
1221	*Cantharellus cibarius*	Agarico	Basidio	18	26	22	11	77	GCA_003521295.1	Kunming University of Science and Technology
1222	*Trichoderma parareesei*	Sordario	Asco	24	12	24	17	77	GCA_001050175.1	Vienna University of Technology
1223	*Geosmithia flava*	Sordario	Asco	28	20	6	23	77	GCA_900188565.1	UNIVERSITY OF NEW HAMPSHIRE
1224	*Sphaerulina musiva*	Dothideo	Asco	21	27	6	23	77	GCA_000320565.2	JGI
1225	*Leptographium procerum*	Sordario	Asco	28	24	2	23	77	GCA_000806385.1	University of Pretoria
1226	*Mycosphaerella sp. PB-2012b*	Dothideo	Asco	21	25	7	23	76	GCA_002116345.1	Canada's Michael Smith Genome Sciences Centre
1227	*Zymoseptoria passerinii*	Dothideo	Asco	26	18	16	16	76	GCA_000223825.2	University of Aarhus, Bioinformatics Research Center
1228	*Apiotrichum laibachii*	Tremello	Basidio	20	18	18	20	76	GCA_001600735.1	RIKEN Center for Life Science Technologies, Division of Genomic Technologies

1229	*Grosmannia clavigera*	Sordario	Asco	26	24	4	22	76	GCA_000143105.2	Michael Smith Laboratories at UBC
1230	*Thielaviopsis euricoi*	Sordario	Asco	25	19	15	17	76	GCA_001599615.1	RIKEN Center for Life Science Technologies, Division of Genomic Technologies
1231	*Omphalotus olearius*	Agarico	Basidio	25	6	7	38	76	GCA_000296255.1	University of Minnesota
1232	*Trichoderma reesei*	Sordario	Asco	24	10	24	17	75	GCA_000167675.2	DOE Joint Genome Institute
1233	*Exophiala sideris*	Eurotio	Asco	20	16	11	28	75	GCA_000835395.1	Broad Institute
1234	*Sanghuangporus baumii*	Agarico	Basidio	22	8	10	35	75	GCA_001481415.2	China University of Petroleum
1235	*Aspergillus sp. HF37*	Eurotio	Asco	26	20	12	16	74	GCA_003698115.1	University of Natural Resources and Life Sciences, Vienna
1236	*Cladophialophora bantiana*	Eurotio	Asco	20	8	10	36	74	GCA_000835475.1	Broad Institute
1237	*Byssochlamys spectabilis*	Eurotio	Asco	16	20	10	28	74	GCA_003990805.1	Kweichow moutai Co.,Ltd.
1238	*Sphaerosporella brunnea*	Pezizo	Asco	23	19	12	20	74	GCA_008704415.1	DOE Joint Genome Institute
1239	*Infundibulicybe gibba*	Agarico	Basidio	25	8	5	36	74	GCA_900068925.1	Royal Botanic Gardens, Kew
1240	*Tolypocladium sp. Sup5 PDA-1*	Sordario	Asco	26	12	8	27	73	GCA_000750105.3	OMRF
1241	*Trichoderma longibrachiatum*	Sordario	Asco	23	10	24	16	73	GCA_000332775.1	Shandong University
1242	*Trichoderma koningii*	Sordario	Asco	23	10	24	16	73	GCA_001950475.1	RIKEN Center for Life Science Technologies, Division of Genomic Technologies
1243	*Verruconis gallopava*	Dothideo	Asco	17	20	6	30	73	GCA_000836295.1	Broad Institute
1244	*Lecanicillium sp. MT-2017a*	Sordario	Asco	22	17	11	22	72	GCA_003056605.1	Northern Arizona University
1245	*Lepidopterella palustris*	Dothideo	Asco	18	15	6	33	72	GCA_001692735.1	DOE Joint Genome Institute
1246	*Daedalea quercina*	Agarico	Basidio	21	13	16	22	72	GCA_001632345.1	DOE Joint Genome Institute
1247	*Pascua guehoae*	Tremello	Basidio	20	14	16	22	72	GCA_001600415.1	RIKEN Center for Life Science Technologies, Division of Genomic Technologies
1248	*Fonsecaea erecta*	Eurotio	Asco	21	9	12	30	72	GCA_001651985.1	Federal University of Parana
1249	*Postia placenta*	Agarico	Basidio	26	14	10	22	72	GCA_002117355.1	DOE Joint Genome Institute
1250	*Neolentinus lepideus*	Agarico	Basidio	24	13	11	23	71	GCA_001632425.1	DOE Joint Genome Institute
1251	*Phialophora americana*	Eurotio	Asco	18	8	15	30	71	GCA_000835435.1	Broad Institute
1252	*Exophiala alcalophila*	Eurotio	Asco	21	6	12	32	71	GCA_001599775.1	RIKEN Center for Life Science Technologies, Division of Genomic Technologies
1253	*Malbranchea cinnamomea*	Eurotio	Asco	25	16	14	16	71	GCA_900128795.2	CHALMERS UNIVERSITY OF TECHNOLOGY
1254	*Ophiostoma ips*	Sordario	Asco	18	24	8	19	69	GCA_002917055.1	University of Pretoria
1255	*Apiotrichum gamsii*	Tremello	Basidio	19	23	9	18	69	GCA_001600315.1	RIKEN Center for Life Science Technologies, Division of Genomic Technologies
1256	*Fistulina hepatica*	Agarico	Basidio	17	18	13	21	69	GCA_000934395.1	DOE Joint Genome Institute
1257	*Calocera cornea*	Dacry	Basidio	20	14	17	18	69	GCA_001632435.1	DOE Joint Genome Institute
1258	*Penicillium decumbens*	Eurotio	Asco	16	20	7	26	69	GCA_002072245.1	Chalmers University of Technology

1259	*Phellinidium pouzarii*	Agarico	Basidio	21	6	8	34	69	GCA_004802695.1	IHI Zittau / TU Dresden
1260	*Thermoascus crustaceus*	Eurotio	Asco	20	14	11	23	68	GCA_001599835.1	RIKEN Center for Life Science Technologies, Division of Genomic Technologies
1261	*Cladophialophora carrionii*	Eurotio	Asco	16	12	14	26	68	GCA_000365165.2	Broad Institute
1262	*Pseudogymnoascus destructans*	Leotio	Asco	18	26	8	16	68	GCA_001641265.1	US Forest Service
1263	*Chiua virens*	Agarico	Basidio	16	8	6	38	68	GCA_003316485.1	Kunming University of Science and Technology
1264	*Metarhizium majus*	Sordario	Asco	24	7	6	30	67	GCA_000814945.1	Shanghai Insititutes for Biological Sciences, CAS
1265	*Microcyclospora tardicrescens*	Dothideo	Asco	16	13	14	24	67	GCA_003012245.1	USDA, ARS, NCAUR
1266	*Thielaviopsis punctulata*	Sordario	Asco	22	16	12	17	67	GCA_002925815.1	Forestry and Agricultural Biotechnology Institute
1267	*Trichosporon coremiiforme*	Tremello	Basidio	17	8	6	35	66	GCA_001752605.1	RIKEN Center for Life Science Technologies, Division of Genomic Technologies
1268	*Grosmannia galeiformis*	Sordario	Asco	21	16	5	24	66	GCA_004028395.1	University of Pretoria
1269	*Fonsecaea nubica*	Eurotio	Asco	18	8	10	30	66	GCA_001646965.1	Federal University of Parana
1270	*Calocera viscosa*	Dacry	Basidio	20	12	15	19	66	GCA_001630345.1	DOE Joint Genome Institute
1271	*Apiotrichum veenhuisii*	Tremello	Basidio	16	15	15	20	66	GCA_001600595.1	RIKEN Center for Life Science Technologies, Division of Genomic Technologies
1272	*Cladophialophora yegresii*	Eurotio	Asco	15	11	12	28	66	GCA_000585515.1	Broad Institute
1273	*Metarhizium robertsii*	Sordario	Asco	22	7	6	30	65	GCA_000187425.2	Metarhizium genome sequencing Consortium
1274	*Mycosphaerella populicola*	Dothideo	Asco	18	22	5	20	65	GCA_000291705.1	University of British Columbia
1275	*Eremomyces bilateralis*	Dothideo	Asco	15	16	12	22	65	GCA_010015585.1	DOE Joint Genome Institute
1276	*Boletus edulis*	Agarico	Basidio	17	4	10	34	65	GCA_003316165.1	Kunming University of Science and Technology
1277	*Moniliophthora perniciosa*	Agarico	Basidio	15	12	10	28	65	GCA_000183025.1	Universidade Estadual de Campinas (UNICAMP), Brazil
1278	*Metarhizium guizhouense*	Sordario	Asco	23	7	6	28	64	GCA_000814955.1	Shanghai Insititutes for Biological Sciences, CAS
1279	*Dirinaria sp. GBRC AP01*	Lecanoro	Asco	25	4	5	30	64	GCA_013315955.1	Gujarat Biotechnology Research Centre
1280	*Lactarius sp. MG50*	Agarico	Basidio	16	0	1	47	64	GCA_003314065.1	Kunming University of Science and Technology
1281	*Basidioascus undulatus*	Geminibasidio	Basidio	18	12	5	29	64	GCA_000826855.1	University of Ottawa
1282	*Fonsecaea pugnacius*	Eurotio	Asco	16	8	10	30	64	GCA_011800825.1	FEDERAL UNIVERSITY OF PARANA
1283	*Rhinocladiella mackenziei*	Eurotio	Asco	17	13	8	26	64	GCA_000835555.1	Broad Institute
1284	*Thielaviopsis musarum*	Sordario	Asco	22	14	12	16	64	GCA_001513885.1	University of Pretoria
1285	*Ustilago trichophora*	Ustilagino	Basidio	16	16	5	26	63	GCA_900323505.1	Technische Universitat Munchen - WZW

1286	*Raffaelea sulphurea*	Sordario	Asco	24	17	3	19	63	GCA_002778055.1	University of Montana
1287	*Fonsecaea pedrosoi*	Eurotio	Asco	16	7	10	30	63	GCA_000835455.1	Broad Institute
1288	*Exophiala spinifera*	Eurotio	Asco	18	6	11	28	63	GCA_010882955.1	Research Center for Medical Mycology - Peking University First Hospital
1289	*Knufia petricola*	Eurotio	Asco	18	18	7	20	63	GCA_002319055.1	University of Natural Resources and Life Sciences
1290	*Davidsoniella eucalypti*	Sordario	Asco	17	21	6	19	63	GCA_004009845.1	Forestry and Agricultural Biotechnology Institute
1291	*Akanthomyces lecanii*	Sordario	Asco	19	13	8	22	62	GCA_001636795.1	Shanghai Institutes for Biological Sciences, CAS
1292	*Ramichloridium luteum*	Dothideo	Asco	15	9	12	26	62	GCA_002368545.1	Northwest A&F University
1293	*Sporothrix phasma*	Sordario	Asco	24	10	10	18	62	GCA_011037845.1	University of Pretoria
1294	*Apiotrichum mycotoxinovorans*	Tremello	Basidio	18	10	14	20	62	GCA_013177335.1	Nanjing TECH University
1295	*Exophiala sp.*	Eurotio	Asco	20	14	8	20	62	GCA_004026505.1	Jill Banfield's Lab at Berkeley
1296	*Calocybe gambosa*	Agarico	Basidio	14	7	11	30	62	GCA_900068965.1	Royal Botanic Gardens, Kew
1297	*Tolypocladium inflatum*	Sordario	Asco	20	8	9	24	61	GCA_003945705.1	University of Minnesota
1298	*Acaromyces ingoldii*	Exobasidio	Basidio	14	18	5	24	61	GCA_003144295.1	DOE Joint Genome Institute
1299	*Eurotiomycetes sp.*	Eurotio	Asco	18	18	7	18	61	GCA_002917005.1	Jill Banfield's Lab at Berkeley
1300	*Boletus sp. MG55*	Agarico	Basidio	15	6	4	36	61	GCA_003313885.1	Kunming University of Science and Technology
1301	*Bretziella fagacearum*	Sordario	Asco	18	17	11	15	61	GCA_002018255.1	Forestry and Agricultural Biotechnology Institute
1302	*Termitomyces heimii*	Agarico	Basidio	22	6	10	23	61	GCA_003313675.1	Kunming University of Science and Technology
1303	*Metarhizium anisopliae*	Sordario	Asco	21	6	6	27	60	GCA_013305495.1	Chonbuk National University
1304	*Hirsutella minnesotensis*	Sordario	Asco	20	4	4	32	60	GCA_000956045.1	Institute of Microbiology, Chinese Academy of Sciences
1305	*Usnea hakonensis*	Lecanoro	Asco	23	4	1	32	60	GCA_013423325.1	Swedish Museum of Natural History
1306	*Fonsecaea monophora*	Eurotio	Asco	16	6	8	30	60	GCA_001642475.1	Federal University of Parana
1307	*Suillus luteus*	Agarico	Basidio	18	4	6	32	60	GCA_000827255.1	DOE Joint Genome Institute
1308	*Torrubiella hemipterigena*	Sordario	Asco	18	14	7	20	59	GCA_000825705.1	HKI JENA
1309	*Paecilomyces hepiali*	Sordario	Asco	17	12	8	22	59	GCA_001455915.2	Beijing Normal University
1310	*Hypomyces rosellus*	Sordario	Asco	24	4	11	20	59	GCA_011799845.1	Jilin Agricultural University
1311	*Metarhizium brunneum*	Sordario	Asco	19	6	6	28	59	GCA_000814965.1	Shanghai Insititutes for Biological Sciences, CAS
1312	*Lentinula edodes*	Agarico	Basidio	16	12	3	28	59	GCA_001562095.1	Chungbuk National University
1313	*Megacollybia platyphylla*	Agarico	Basidio	20	4	9	26	59	GCA_900068915.1	Royal Botanic Gardens, Kew
1314	*Cordyceps javanica*	Sordario	Asco	18	12	8	20	58	GCA_006981985.1	The Institute of Vegetables and Flowers CAAS
1315	*Trichosporon ovoides*	Tremello	Basidio	14	6	6	32	58	GCA_009833065.1	Heilongjiang bayi agricultural university
1316	*Tolypocladium sp. Salcha MEA-2*	Sordario	Asco	19	8	9	22	58	GCA_000750145.2	INPART

1317	*Exophiala mesophila*	Eurotio	Asco	24	3	6	25	58	GCA_000836275.1	Broad Institute
1318	*Wolfiporia cocos*	Agarico	Basidio	16	12	14	16	58	GCA_000344635.1	DOE Joint Genome Institute
1319	*Davidsoniella neocaledoniae*	Sordario	Asco	18	17	6	17	58	GCA_009806295.1	Forestry and Agricultural Biotechnology Institute
1320	*Davidsoniella australis*	Sordario	Asco	17	17	7	17	58	GCA_009806335.1	Forestry and Agricultural Biotechnology Institute
1321	*Davidsoniella virescens*	Sordario	Asco	18	19	6	15	58	GCA_001513805.1	University of Pretoria, Forestry and Agricultural Biotechnology Institute
1322	*Suillus pictus*	Agarico	Basidio	20	2	6	30	58	GCA_003313085.1	Kunming University of Science and Technology
1323	*Suillus alpinus*	Agarico	Basidio	15	4	5	34	58	GCA_003316505.1	Kunming University of Science and Technology
1324	*Auricularia polytricha*	Agarico	Basidio	16	2	6	34	58	GCA_003316125.1	Kunming University of Science and Technology
1325	*Cordyceps farinosa*	Sordario	Asco	17	12	8	20	57	GCA_000733625.1	CSIR- Institute of Microbial technology (IMTECH)
1326	*Geosmithia morbida*	Sordario	Asco	19	16	5	17	57	GCA_900108815.1	UNIVERSITY OF NEW HAMPSHIRE
1327	*Cutaneotrichosporon dermatis*	Tremello	Basidio	14	16	11	16	57	GCA_003116895.1	RIKEN Center for Life Science Technologies, Division of Genomic Technologies
1328	*Prillingera fragicola*	Tremello	Basidio	14	13	12	18	57	GCA_002335605.1	RIKEN Center for Life Science Technologies, Division of Genomic Technologies
1329	*Rhizopus oryzae*	Mucoro	Mucoro	16	24	0	16	56	GCA_000697195.1	IGS
1330	*Evernia prunastri*	Lecanoro	Asco	20	3	2	31	56	GCA_003184365.1	Senckenberg
1331	*Lactarius indigo*	Agarico	Basidio	16	2	2	36	56	GCA_003313985.1	Kunming University of Science and Technology
1332	*Dacryopinax primogenitus*	Dacry	Basidio	17	8	14	17	56	GCA_000292625.1	DOE Joint Genome Institute
1333	*Albatrellus ellisii*	Agarico	Basidio	12	4	6	34	56	GCA_003314395.1	Kunming University of Science and Technology
1334	*Suillus brevipes*	Agarico	Basidio	18	2	4	32	56	GCA_011800875.1	DOE Joint Genome Institute
1335	*Agaricus bisporus*	Agarico	Basidio	16	2	8	30	56	GCA_008271545.1	Jilin Agricultural Unversity
1336	*Pterula multifida*	Agarico	Basidio	12	8	6	30	56	GCA_900068985.1	Royal Botanic Gardens, Kew
1337	*Moesziomyces sp. F16C1*	Ustilagino	Basidio	14	13	10	18	55	GCA_003004725.1	Universidade de Sao Paulo
1338	*Lactarius hatsudake*	Agarico	Basidio	16	2	1	36	55	GCA_003315955.1	Kunming University of Science and Technology
1339	*Berkeleyomyces basicola*	Sordario	Asco	15	19	6	15	55	GCA_003671435.1	Forestry and Agricultural Biotechnology Institute
1340	*Huntiella moniliformis*	Sordario	Asco	16	14	5	20	55	GCA_000712465.1	Forestry and Agricultural Biotechnology Institute
1341	*Gomphus sp. MG54*	Agarico	Basidio	10	0	0	45	55	GCA_003314385.1	Kunming University of Science and Technology

1342	*Coprinus comatus*	Agarico	Basidio	19	4	9	23	55	GCA_003316025.1	Kunming University of Science and Technology
1343	*Beauveria pseudobassiana*	Sordario	Asco	20	8	2	24	54	GCA_003267905.1	National Institute of Horticultural and Herbal Science, Rural Development Administration
1344	*Hirsutella thompsonii*	Sordario	Asco	17	4	4	29	54	GCA_000472145.1	Institute of Microbial Technology
1345	*Rhizopus delemar*	Mucoro	Mucoro	13	25	0	16	54	GCA_000149305.1	Broad Institute
1346	*Coniosporium apollinis*	Dothideo	Asco	17	12	4	21	54	GCA_000281105.1	Broad Institute
1347	*Rhizopus stolonifer*	Mucoro	Mucoro	12	26	1	15	54	GCA_000697035.1	IGS
1348	*Exophiala dermatitidis*	Eurotio	Asco	14	6	10	24	54	GCA_010883545.1	Research Center for Medical Mycology - Peking University First Hospital
1349	*Fibroporia radiculosa*	Agarico	Basidio	18	12	10	14	54	GCA_000313525.1	Forest Products, Mississippi State University
1350	*Rhizopogon vinicolor*	Agarico	Basidio	16	2	6	30	54	GCA_001658105.1	DOE Joint Genome Institute
1351	*Rhizoclosmatium globosum*	Chytridio	Chytridio	8	16	0	29	53	GCA_002104985.1	DOE Joint Genome Institute
1352	*Cladobotryum protrusum*	Sordario	Asco	22	2	10	19	53	GCA_004303015.1	Jilin Agricultural University
1353	*Cordyceps pruinosa*	Sordario	Asco	16	11	6	20	53	GCA_003025255.1	National Institute of Horticultural and Herbal Science, Rural Development Administration
1354	*Umbelopsis isabellina*	Umbelopsido	Mucoro	10	22	6	15	53	GCA_000697415.1	IGS
1355	*Hirsutella rhossiliensis*	Sordario	Asco	16	5	6	26	53	GCA_004142005.1	Chinese Academy of Sciences
1356	*Gyalolechia flavorubescens*	Lecanoro	Asco	16	6	7	24	53	GCA_000442125.1	Seoul National University
1357	*Takashimella koratensis*	Tremello	Basidio	12	17	12	12	53	GCA_003116875.1	RIKEN Center for Life Science Technologies, Division of Genomic Technologies
1358	*Gonapodya prolifera*	*Monoblepharido*	Chytridio	9	26	0	18	53	GCA_001574975.1	DOE Joint Genome Institute
1359	*Boletus sp. MG95*	Agarico	Basidio	12	4	5	32	53	GCA_003313155.1	Kunming University of Science and Technology
1360	*Neoboletus brunneissimus*	Agarico	Basidio	14	2	5	32	53	GCA_003316195.1	Kunming University of Science and Technology
1361	*Cordyceps cicadae*	Sordario	Asco	15	8	6	23	52	GCA_010211705.1	Anhui Agricultural University
1362	*Exophiala phaeomuriformis*	Eurotio	Asco	14	6	8	24	52	GCA_010883475.1	Research Center for Medical Mycology - Peking University First Hospital
1363	*Moesziomyces sp. F5C1*	Ustilagino	Basidio	12	12	10	18	52	GCA_003004715.1	Universidade de Sao Paulo
1364	*Pseudevernia furfuracea*	Lecanoro	Asco	18	2	2	30	52	GCA_003184345.1	Senckenberg
1365	*Cladonia rangiferina*	Lecanoro	Asco	14	4	2	32	52	GCA_006146055.1	Lake Superior State University
1366	*Cutaneotrichosporon daszewskae*	Tremello	Basidio	12	14	10	16	52	GCA_002335585.1	RIKEN Center for Life Science Technologies, Division of Genomic Technologies

1367	*Endoconidiophora laricicola*	Sordario	Asco	18	15	4	15	52	GCA_001640655.1	Forestry and Agricultural Biotechnology Institute
1368	*Huntiella savannae*	Sordario	Asco	16	14	5	17	52	GCA_001483325.1	Forestry and Agricultural Biotechnology Institute
1369	*Huntiella decipiens*	Sordario	Asco	16	14	5	17	52	GCA_003032515.1	Forestry and Agricultural Biotechnology Institute
1370	*Albatrellus sp. MG142*	Agarico	Basidio	14	2	6	30	52	GCA_003314695.1	Kunming University of Science and Technology
1371	*Beauveria sp. IMV 00265*	Sordario	Asco	18	9	2	22	51	GCA_001931865.2	Jet Propulsion Laboratory, California Institute of Technology
1372	*Beauveria rudraprayagi*	Sordario	Asco	16	8	4	23	51	GCA_000733645.1	CSIR- Institute of Microbial technology (IMTECH)
1373	*Paecilomyces penicillatus*	Sordario	Asco	22	10	5	14	51	GCA_005765155.1	Biotechnology and Nuclear Technology Research Institute
1374	*Cordyceps sp. RAO-2017*	Sordario	Asco	16	5	4	26	51	GCA_002591385.1	Utrecht University
1375	*Moesziomyces aphidis*	Ustilagino	Basidio	12	12	10	17	51	GCA_000517465.1	Fraunhofer IGB
1376	*Symbiotaphrina buchneri*	Xylono	Asco	10	14	8	19	51	GCA_001599915.1	RIKEN Center for Life Science Technologies, Division of Genomic Technologies
1377	*Sparassis latifolia*	Agarico	Basidio	18	4	9	20	51	GCA_009812315.1	Chosun University
1378	*Gigaspora margarita*	Glomero	Mucoro	12	3	0	36	51	GCA_009809945.1	CNR
1379	*Paxillus involutus*	Agarico	Basidio	12	4	5	30	51	GCA_000827475.1	JGI
1380	*Endoconidiophora polonica*	Sordario	Asco	17	15	4	15	51	GCA_001856765.1	Forestry and Agricultural Biotechnology Institute
1381	*Huntiella bhutanensis*	Sordario	Asco	16	13	5	17	51	GCA_002018275.1	Forestry and Agricultural Biotechnology Institute
1382	*Huntiella omanensis*	Sordario	Asco	16	13	5	17	51	GCA_000833645.1	FABI
1383	*Chroogomphus rutilus*	Agarico	Basidio	12	0	2	37	51	GCA_003314275.1	Kunming University of Science and Technology
1384	*Cordyceps fumosorosea*	Sordario	Asco	16	8	4	22	50	GCA_001636725.1	Shanghai Institutes for Biological Sciences, CAS
1385	*Ceratocystiopsis minuta*	Sordario	Asco	14	16	4	16	50	GCA_001676865.1	University of Pretoria
1386	*Claviceps fusiformis*	Sordario	Asco	14	12	10	14	50	GCA_000223055.1	University of Kentucky, Dept of Plant Pathology
1387	*Ceratocystis manginecans*	Sordario	Asco	15	15	6	14	50	GCA_006408425.1	University of Pretoria
1388	*Leucosporidium scottii*	Microbotryo	Basidio	11	12	6	21	50	GCA_003054985.1	UCIBIO-REQUIMTE, Faculdade de Ciencias e Tecnologia, Universidade Nova da Lisboa
1389	*Ceratocystis eucalypticola*	Sordario	Asco	15	15	6	14	50	GCA_001513815.1	University of pretoria
1390	*Ceratocystis cacaofunesta*	Sordario	Asco	15	15	6	14	50	GCA_002776505.1	University of Campinas
1391	*Ceratocystis fimbriata*	Sordario	Asco	15	15	6	14	50	GCA_009914735.1	Universidade Federal de Vicosa
1392	*Eurotiomycetes sp. MA 6038*	Eurotio	Asco	15	12	6	17	50	GCA_003004525.2	University of Natural Resources and Life Sciences, Vienna

1393	*Ceratocystis platani*	Sordario	Asco	15	15	6	14	50	GCA_000978885.1	University of Neuchatel
1394	*Russula griseocarnosa*	Agarico	Basidio	10	0	2	38	50	GCA_004801975.1	Research Institute of Tropical Forestry, Chinese Academy of Forestry
1395	*Ceratocystis harringtonii*	Sordario	Asco	15	15	6	14	50	GCA_002018265.1	Forestry and Agricultural Biotechnology Institute
1396	*Rhizopogon vesiculosus*	Agarico	Basidio	13	2	7	28	50	GCA_001882365.1	University of Florida
1397	*Ceratocystis albifundus*	Sordario	Asco	15	15	6	14	50	GCA_002742255.2	University of Pretoria
1398	*Metarhizium acridum*	Sordario	Asco	16	6	3	24	49	GCA_000187405.1	Metarhizium genome sequencing Consortium
1399	*Beauveria brongniartii*	Sordario	Asco	16	9	2	22	49	GCA_001636735.1	Shanghai Institutes for Biological Sciences, CAS
1400	*Bifiguratus adelaidae*	Endogono	Mucoro	16	12	5	16	49	GCA_002261195.1	University of California, Riverside
1401	*Albophoma yamanashiensis*	Sordario	Asco	15	8	5	21	49	GCA_001600555.1	RIKEN Center for Life Science Technologies, Division of Genomic Technologies
1402	*fungal sp. No.11243*	0	0	14	12	5	18	49	GCA_000836255.1	Tsukuba Biotechnology Research Center, Astellas Pharma Inc.,
1403	*Moesziomyces antarcticus*	Ustilagino	Basidio	12	11	10	16	49	GCA_900322835.1	Technische Universitat Munchen - WZW
1404	*Sparassis crispa*	Agarico	Basidio	18	6	9	16	49	GCA_003851025.1	Department of Pediatric Cardiology, Tokyo Women's Medical University
1405	*Cladonia uncialis*	Lecanoro	Asco	14	4	3	28	49	GCA_002927785.1	University of Manitoba
1406	*Eurotiomycetes sp. MA 6039*	Eurotio	Asco	16	13	4	16	49	GCA_003004485.1	University of Natural Resources and Life Sciences, Vienna
1407	*Ceratocystis adiposa*	Sordario	Asco	14	13	7	15	49	GCA_001640685.1	Forestry and Agricultural Biotechnology Institute
1408	*Chalaropsis thielavioides*	Sordario	Asco	15	14	5	15	49	GCA_001599435.1	RIKEN Center for Life Science Technologies, Division of Genomic Technologies
1409	*Rhizopogon sp. AM-2018a*	Agarico	Basidio	12	3	6	28	49	GCA_002995095.1	Oregon State University
1410	*Mucor racemosus*	Mucoro	Mucoro	16	7	1	24	48	GCA_000697255.1	IGS
1411	*Beauveria bassiana*	Sordario	Asco	16	8	2	22	48	GCA_003337105.1	Biotechnology Institute- National University of Colombia- Bogota
1412	*Polychaeton citri*	Dothideo	Asco	12	8	5	23	48	GCA_010093785.1	DOE Joint Genome Institute
1413	*Herpotrichiellaceae sp. UM238*	Eurotio	Asco	18	8	0	22	48	GCA_000315175.1	UNIVERSITY OF MALAYA
1414	*Pseudozyma tsukubaensis*	Ustilagino	Basidio	12	14	6	16	48	GCA_001736125.1	RWTH Aachen University
1415	*Tylopilus plumbeoviolaceoides*	Agarico	Basidio	10	2	5	31	48	GCA_003316375.1	Kunming University of Science and Technology

1416	*Ceratocystis smalleyi*	Sordario	Asco	15	14	6	13	48	GCA_003449175.1	Forestry and Agricultural Biotechnology Institute
1417	*Verruconis sp.*	Dothideo	Asco	13	12	5	18	48	GCA_004026245.1	Jill Banfield's Lab at Berkeley
1418	*Floccularia luteovirens*	Agarico	Basidio	12	2	8	26	48	GCA_009739215.1	Zhejiang University
1419	*Basidiobolus meristosporus*	Basidiobolo	Zoopago	10	3	0	34	47	GCA_002104905.1	DOE Joint Genome Institute
1420	*Linderina pennispora*	Kickxello	Zoopago	3	4	1	39	47	GCA_002104995.1	DOE Joint Genome Institute
1421	*Tolypocladium paradoxum*	Sordario	Asco	17	6	7	17	47	GCA_002916505.1	Oregon State University
1422	*Tuber microsphaerosporum*	Pezizo	Asco	15	12	2	18	47	GCA_003521225.1	Kunming University of Science and Technology
1423	*Cryomyces antarcticus*	Dothideo	Asco	10	7	4	26	47	GCA_000504465.1	VIBT Extremophile Center, University of Natural Resources and Life Sciences Vienna, Austria
1424	*Claviceps paspali*	Sordario	Asco	11	12	10	14	47	GCA_013435705.1	INIA
1425	*Atkinsonella hypoxylon*	Sordario	Asco	11	14	9	13	47	GCA_000729835.1	University of Kentucky
1426	*Arthrocladium fulminans*	Eurotio	Asco	20	10	4	13	47	GCA_003614865.1	Westerdijk Fungal Biodiversity Institute
1427	*Peltaster fructicola*	Dothideo	Asco	10	9	8	20	47	GCA_001592805.2	Northwest A&F University
1428	*Capronia coronata*	Eurotio	Asco	15	6	6	20	47	GCA_000585585.1	Broad Institute
1429	*Suillus sp. MG131*	Agarico	Basidio	14	5	4	24	47	GCA_003313855.1	Kunming University of Science and Technology
1430	*Cordyceps tenuipes*	Sordario	Asco	14	8	4	20	46	GCA_003025305.1	National Institute of Horticultural and Herbal Science, Rural Development Administration
1431	*Rhizopus azygosporus*	Mucoro	Mucoro	18	10	0	18	46	GCA_003325435.1	University of California, Riverside
1432	*Moelleriella libera*	Sordario	Asco	14	4	6	22	46	GCA_001636675.1	Shanghai Institutes for Biological Sciences, CAS
1433	*Epichloe brachyelytri*	Sordario	Asco	14	8	8	16	46	GCA_000222915.1	University of Kentucky, Dept of Plant Pathology
1434	*Pseudozyma hubeiensis*	Ustilagino	Basidio	12	10	8	16	46	GCA_000403515.1	Kitami Institute of Technology
1435	*Bullera alba*	Tremello	Basidio	10	12	14	10	46	GCA_001600095.1	RIKEN Center for Life Science Technologies, Division of Genomic Technologies
1436	*Rusavskia elegans*	Lecanoro	Asco	16	4	6	20	46	GCA_011316305.1	Yunnan Academy of Forestry
1437	*Apiotrichum gracile*	Tremello	Basidio	14	4	9	19	46	GCA_001600335.1	RIKEN Center for Life Science Technologies, Division of Genomic Technologies
1438	*Cladonia metacorallifera*	Lecanoro	Asco	13	4	3	26	46	GCA_000482085.2	Seoul National University
1439	*Drechslerella stenobrocha*	Orbilio	Asco	17	8	8	13	46	GCA_000525045.1	Institute of Microbiology, Chinese Academy of Sciences
1440	*Boletus speciosus*	Agarico	Basidio	11	4	5	26	46	GCA_003316055.1	Kunming University of Science and Technology

1441	*Ramaria sp. MG151*	Agarico	Basidio	14	2	2	28	46	GCA_003314545.1	Kunming University of Science and Technology
1442	*Epichloe gansuensis*	Sordario	Asco	13	8	9	15	45	GCA_000222895.2	University of Kentucky, Dept of Plant Pathology
1443	*Epichloe inebrians*	Sordario	Asco	13	7	9	16	45	GCA_000309355.1	University of Kentucky
1444	*Atkinsonella texensis*	Sordario	Asco	10	14	7	14	45	GCA_001008035.1	University of Kentucky
1445	*Epichloe bromicola*	Sordario	Asco	12	10	8	15	45	GCA_002319005.1	University of Kentucky
1446	*Balansia obtecta*	Sordario	Asco	12	12	7	14	45	GCA_000709145.1	University of Kentucky
1447	*Sporisorium reilianum*	Ustilagino	Basidio	12	9	8	16	45	GCA_900162835.1	Technische Universitat Munchen - WZW
1448	*Pseudozyma sp. F8B2*	Ustilagino	Basidio	11	10	8	16	45	GCA_003004685.1	Universidade de Sao Paulo
1449	*Sporisorium iseilematis-ciliati*	Ustilagino	Basidio	11	10	8	16	45	GCA_001748505.1	RWTH Aachen University
1450	*Capronia epimyces*	Eurotio	Asco	12	4	4	25	45	GCA_000585565.1	Broad Institute
1451	*Gigaspora rosea*	Glomero	Mucoro	10	3	0	32	45	GCA_003550325.1	INRA
1452	*Solicoccozyma phenolica*	Tremello	Basidio	10	16	5	14	45	GCA_001600015.1	RIKEN Center for Life Science Technologies, Division of Genomic Technologies
1453	*Paxillus rubicundulus*	Agarico	Basidio	10	2	5	28	45	GCA_000827395.1	DOE Joint Genome Institute
1454	*Rhizopogon rudus*	Agarico	Basidio	12	3	2	28	45	GCA_002995055.1	Oregon State University
1455	*Retiboletus ornatipes*	Agarico	Basidio	12	3	6	24	45	GCA_003316065.1	Kunming University of Science and Technology
1456	*Boletus bicolor*	Agarico	Basidio	13	2	6	24	45	GCA_003316205.1	Kunming University of Science and Technology
1457	*Hypomyces perniciosus*	Sordario	Asco	18	6	6	14	44	GCA_008477525.1	Jilin Agricultural Unversity
1458	*Cordyceps militaris*	Sordario	Asco	16	6	2	20	44	GCA_003332165.1	pangugene
1459	*Epichloe sylvatica*	Sordario	Asco	12	8	8	16	44	GCA_001008265.1	University of Kentucky
1460	*Epichloe baconii*	Sordario	Asco	14	8	8	14	44	GCA_000729845.1	University of Kentucky
1461	*Sporisorium graminicola*	Ustilagino	Basidio	11	11	6	16	44	GCA_005498985.1	University of Liverpool, Institute of Integrative Biology
1462	*Epichloe aotearoae*	Sordario	Asco	12	8	10	14	44	GCA_000729855.1	University of Kentucky
1463	*Aciculosporium take*	Sordario	Asco	12	10	6	16	44	GCA_000222935.2	University of Kentucky, Dept of Plant Pathology
1464	*Tuber borchii*	Pezizo	Asco	16	12	2	14	44	GCA_003070745.1	DOE Joint Genome Institute
1465	*Tuber brumale*	Pezizo	Asco	14	12	3	15	44	GCA_014065205.1	INRAE
1466	*Synchytrium microbalum*	Chytridio	Chytridio	14	6	0	24	44	GCA_006535985.1	Agriculture and Agri-Food Canada
1467	*Lactarius sp. MG121*	Agarico	Basidio	12	1	1	30	44	GCA_003315925.1	Kunming University of Science and Technology
1468	*Cutaneotrichosporon cutaneum*	Tremello	Basidio	10	6	10	18	44	GCA_001600715.1	RIKEN Center for Life Science Technologies, Division of Genomic Technologies
1469	*Russula foetens*	Agarico	Basidio	12	0	4	28	44	GCA_003316565.1	Kunming University of Science and Technology
1470	*Lactarius deliciosus*	Agarico	Basidio	12	0	0	32	44	GCA_006680135.1	UCSI University

1471	*Serpula lacrymans*	Agarico	Basidio	17	6	7	14	44	GCA_002891385.1	University of Oslo
1472	*Xerocomus impolitus*	Agarico	Basidio	10	6	6	22	44	GCA_003316335.1	Kunming University of Science and Technology
1473	*Suillellus subvelutipes*	Agarico	Basidio	11	2	4	27	44	GCA_003316035.1	Kunming University of Science and Technology
1474	*Solicoccozyma terricola*	Tremello	Basidio	16	16	2	10	44	GCA_001600875.1	RIKEN Center for Life Science Technologies, Division of Genomic Technologies
1475	*Butyriboletus roseoflavus*	Agarico	Basidio	12	4	4	24	44	GCA_003315995.1	Kunming University of Science and Technology
1476	*Mucor indicus*	Mucoro	Mucoro	12	3	0	28	43	GCA_000697295.1	IGS
1477	*Microcyclospora pomicola*	Dothideo	Asco	10	6	12	15	43	GCA_002786065.1	Northwest A&F University
1478	*Syncephalastrum monosporum*	Mucoro	Mucoro	9	16	2	16	43	GCA_000697355.1	IGS
1479	*Ophiocordyceps camponoti-leonardi* *(nom. inval.)*	Sordario	Asco	12	7	3	21	43	GCA_003339455.1	National Center for Genetic Engineering and Biotechnology
1480	*Ophiocordyceps polyrhachis-furcata*	Sordario	Asco	12	6	5	20	43	GCA_001633055.2	National Center for Genetic Engineering and Biotechnology (BIOTEC)
1481	*Claviceps cf. purpurea*	Sordario	Asco	12	12	7	12	43	GCA_004016475.1	Agriculture and Agri-Food Canada
1482	*Ophiocordyceps sinensis*	Sordario	Asco	14	2	3	24	43	GCA_002077885.1	Institute of Microbiology, Chinese Academy of Sciences
1483	*Papiliotrema flavescens*	Tremello	Basidio	8	12	11	12	43	GCA_000442785.1	OSU/OARDC
1484	*Letharia lupina*	Lecanoro	Asco	16	2	2	23	43	GCA_014066315.1	Eastern Washington University
1485	*Vanrija humicola*	Tremello	Basidio	16	6	5	16	43	GCA_008065275.1	Ruhr-University Bochum
1486	*Ustilago tritici*	Ustilagino	Basidio	12	8	7	16	43	GCA_002993085.1	University of Sao Paulo
1487	*Violaceomyces palustris*	Ustilagino	Basidio	10	10	4	19	43	GCA_003144235.1	DOE Joint Genome Institute
1488	*Ambrosiella xylebori*	Sordario	Asco	12	11	6	14	43	GCA_002778035.1	University of Montana
1489	*Gomphus bonarii*	Agarico	Basidio	10	0	1	32	43	GCA_003316585.1	Kunming University of Science and Technology
1490	*Ophio. camponoti-saundersi* *(nom. inval.)*	Sordario	Asco	12	5	3	22	42	GCA_003339415.1	National Center for Genetic Engineering and Biotechnology
1491	*Epichloe festucae*	Sordario	Asco	12	8	8	14	42	GCA_002318955.1	University of Kentucky
1492	*Claviceps purpurea*	Sordario	Asco	12	12	6	12	42	GCA_004016095.1	Agriculture and Agri-Food Canada
1493	*Claviceps spartinae*	Sordario	Asco	12	11	7	12	42	GCA_004016175.1	Agriculture and Agri-Food Canada
1494	*Claviceps humidiphila*	Sordario	Asco	12	11	7	12	42	GCA_004016155.1	Agriculture and Agri-Food Canada
1495	*Epichloe typhina*	Sordario	Asco	12	8	8	14	42	GCA_000308955.1	University of Kentucky
1496	*Epichloe elymi*	Sordario	Asco	12	8	8	14	42	GCA_002591845.1	Massey University
1497	*Epichloe amarillans*	Sordario	Asco	12	8	8	14	42	GCA_000223075.2	University of Kentucky, Dept of Plant Pathology
1498	*Letharia columbiana*	Lecanoro	Asco	15	3	2	22	42	GCA_014066305.1	Eastern Washington University

1499	*Ustilago cynodontis*	Ustilagino	Basidio	12	8	6	16	42	GCA_001736195.1	RWTH Aachen University
1500	*Tuber umbilicatum*	Pezizo	Asco	12	14	2	14	42	GCA_003313605.1	Kunming University of Science and Technology
1501	*Ustilago xerochloae*	Ustilagino	Basidio	10	8	8	16	42	GCA_001736075.1	RWTH Aachen University
1502	*Tuber magnatum*	Pezizo	Asco	14	10	4	14	42	GCA_003182015.1	INRA
1503	*Lactarius trivialis*	Agarico	Basidio	11	0	1	30	42	GCA_003315845.1	Kunming University of Science and Technology
1504	*Papiliotrema laurentii*	Tremello	Basidio	8	12	10	12	42	GCA_012922615.1	Jet Propulsion Laboratory, California Institute of Technology
1505	*Piloderma croceum*	Agarico	Basidio	14	2	0	26	42	GCA_000827315.1	DOE Joint Genome Institute
1506	*Lactifluus pinguis*	Agarico	Basidio	7	0	0	35	42	GCA_003313945.1	Kunming University of Science and Technology
1507	*Ramaria cf. rubripermanens*	Agarico	Basidio	12	0	2	28	42	GCA_003316465.1	Kunming University of Science and Technology
1508	*Tolypocladium ophioglossoides*	Sordario	Asco	13	6	7	15	41	GCA_001189435.1	Oregon State University
1509	*Anthracocystis flocculosa*	Ustilagino	Basidio	10	15	4	12	41	GCA_900322975.1	Technische Universitat Munchen - WZW
1510	*Hypocrella siamensis*	Sordario	Asco	12	4	5	20	41	GCA_000731825.1	CSIR- Institute of Microbial technology (IMTECH)
1511	*Syncephalastrum racemosum*	Mucoro	Mucoro	9	16	2	14	41	GCA_002105135.1	DOE Joint Genome Institute
1512	*Leptoxyphium fumago*	Dothideo	Asco	9	4	4	24	41	GCA_001660795.1	International Institute Zittau - TU Dresden
1513	*Claviceps arundinis*	Sordario	Asco	10	12	7	12	41	GCA_004016465.1	Agriculture and Agri-Food Canada
1514	*Monascus purpureus*	Eurotio	Asco	11	10	4	16	41	GCA_011319195.1	Beijing Technology and Business University
1515	*Dioszegia crocea*	Tremello	Basidio	12	12	10	7	41	GCA_001600615.1	RIKEN Center for Life Science Technologies, Division of Genomic Technologies
1516	*Kalmanozyma brasiliensis*	Ustilagino	Basidio	10	10	6	15	41	GCA_000497045.1	Laboratorio Nacional de Ciencia e Tecnologia do Bioetanol
1517	*Tuber indicum*	Pezizo	Asco	14	10	3	14	41	GCA_006112555.1	College of Pharmacy and Biological Engineering
1518	*Dioszegia aurantiaca*	Tremello	Basidio	10	14	9	8	41	GCA_001600655.1	RIKEN Center for Life Science Technologies, Division of Genomic Technologies
1519	*Cladonia macilenta*	Lecanoro	Asco	10	4	3	24	41	GCA_000444155.1	Seoul National University
1520	*Russula aff. compacta*	Agarico	Basidio	9	0	2	30	41	GCA_003313875.1	Kunming University of Science and Technology
1521	*Amanita thiersii*	Agarico	Basidio	10	4	5	22	41	GCA_002554575.1	JGI
1522	*Caloboletus calopus*	Agarico	Basidio	10	2	3	26	41	GCA_003316085.1	Kunming University of Science and Technology
1523	*Rhizopogon villosulus*	Agarico	Basidio	11	2	4	24	41	GCA_002995315.1	Oregon State University

1524	*Metarhizium rileyi*	Sordario	Asco	14	6	2	18	40	GCA_007866325.1	Embrapa
1525	*Gongronella sp. w5*	Mucoro	Mucoro	8	12	4	16	40	GCA_001650995.1	Anhui University
1526	*Ustilaginoidea virens*	Sordario	Asco	12	10	4	14	40	GCA_000965225.2	Computational Biology Research Center (CBRC), National Institute of Advanced Industrial Science and Technology (AIST)
1527	*Syncephalastrum sp. IA-2019*	Mucoro	Mucoro	7	16	2	15	40	GCA_013461545.1	University of Melbourne
1528	*Dissoconium aciculare*	Dothideo	Asco	12	2	8	18	40	GCA_010015565.1	DOE Joint Genome Institute
1529	*Epichloe sp. AL9924*	Sordario	Asco	12	8	8	12	40	GCA_000729825.1	University of Kentucky
1530	*Apiotrichum brassicae*	Tremello	Basidio	8	2	14	16	40	GCA_001600295.1	RIKEN Center for Life Science Technologies, Division of Genomic Technologies
1531	*Tuber melanosporum*	Pezizo	Asco	13	10	3	14	40	GCA_000151645.1	The French-Italian Tuber Genome Consortium
1532	*Cutaneotrichosporon arboriformis*	Tremello	Basidio	8	5	9	18	40	GCA_002335565.1	RIKEN Center for Life Science Technologies, Division of Genomic Technologies
1533	*Ustilago hordei*	Ustilagino	Basidio	11	8	8	13	40	GCA_003012045.1	Jawaharlal Nehru Centre for Advanced Scientific Research
1534	*Suillus placidus*	Agarico	Basidio	12	2	4	22	40	GCA_003313645.1	Kunming University of Science and Technology
1535	*Rhizopogon vulgaris*	Agarico	Basidio	10	1	3	26	40	GCA_002995295.1	Oregon State University
1536	*Rhizopogon hawkerae*	Agarico	Basidio	10	4	4	22	40	GCA_002995035.1	Oregon State University
1537	*Neoboletus magnificus*	Agarico	Basidio	10	0	2	28	40	GCA_003316145.1	Kunming University of Science and Technology
1538	*Escovopsis sp. TC*	Sordario	Asco	16	6	9	8	39	GCA_003055185.1	University of East Anglia
1539	*Ophiocordyceps camponoti-rufipedis*	Sordario	Asco	10	6	3	20	39	GCA_002591395.1	Utrecht University
1540	*Elaphomyces granulatus*	Eurotio	Asco	10	4	2	23	39	GCA_002240705.1	Oregon State University
1541	*Leucosporidium creatinivorum*	Microbotryo	Basidio	10	6	2	21	39	GCA_001600635.1	RIKEN Center for Life Science Technologies, Division of Genomic Technologies
1542	*Neohortaea acidophila*	Dothideo	Asco	10	10	3	16	39	GCA_010093505.1	DOE Joint Genome Institute
1543	*Taiwanofungus camphoratus*	Agarico	Basidio	14	2	7	16	39	GCA_000766995.1	Academia Sinica
1544	*Rhizopogon parksii*	Agarico	Basidio	10	4	3	22	39	GCA_002994865.1	Oregon State University
1545	*Lactifluus hygrophoroides*	Agarico	Basidio	10	0	1	28	39	GCA_003314055.1	Kunming University of Science and Technology
1546	*Lactarius piperatus*	Agarico	Basidio	8	0	0	31	39	GCA_003315875.1	Kunming University of Science and Technology
1547	*Ophiocordyceps australis*	Sordario	Asco	15	4	3	16	38	GCA_002591415.1	Utrecht University
1548	*Ustilago maydis*	Ustilagino	Basidio	10	5	6	17	38	GCA_001599495.1	RIKEN Center for Life Science Technologies, Division of Genomic Technologies

1549	*Sporisorium scitamineum*	Ustilagino	Basidio	10	10	4	14	38	GCA_000772675.1	Key Lab of Sugarcane Biology and Genetic Breeding, Ministry of Agriculture, P.R.China
1550	*Kockovaella imperatae*	Tremello	Basidio	8	10	10	10	38	GCA_002102565.1	DOE Joint Genome Institute
1551	*Kwoniella heveanensis*	Tremello	Basidio	8	12	6	12	38	GCA_000507405.3	Broad Institute
1552	*Tricholoma saponaceum*	Agarico	Basidio	13	2	3	20	38	GCA_003313625.1	Kunming University of Science and Technology
1553	*Ustilago bromivora*	Ustilagino	Basidio	10	8	6	14	38	GCA_900101485.1	Institute of Bioinformatics and Systems Biology
1554	*Russula abietina*	Agarico	Basidio	7	0	2	29	38	GCA_003313715.1	Kunming University of Science and Technology
1555	*Powellomyces hirtus*	Chytridio	Chytridio	9	12	1	16	38	GCA_006536005.1	Agriculture and Agri-Food Canada
1556	*Pulveroboletus ravenelii*	Agarico	Basidio	6	2	4	26	38	GCA_003316545.1	Kunming University of Science and Technology
1557	*Conidiobolus incongruus*	Entomophthoro	Zoopago	7	2	1	27	37	GCA_000697335.1	IGS
1558	*Metarhizium album*	Sordario	Asco	11	4	6	16	37	GCA_000804445.1	Shanghai Institutes for Biological Sciences, CAS
1559	*Escovopsis weberi*	Sordario	Asco	15	4	7	11	37	GCA_003055145.1	University of East Anglia
1560	*Ophiocordyceps camponoti-floridani*	Sordario	Asco	9	6	3	19	37	GCA_012980515.1	University of Central Florida
1561	*Ophiocordyceps unilateralis*	Sordario	Asco	10	4	3	20	37	GCA_001272575.2	Utrecht University
1562	*Monascus ruber*	Eurotio	Asco	9	10	4	14	37	GCA_002976275.1	Fujian Institute of Microbiology
1563	*Apiotrichum domesticum*	Tremello	Basidio	9	2	12	14	37	GCA_001599015.1	RIKEN Center for Life Science Technologies, Division of Genomic Technologies
1564	*Trichosporon asahii*	Tremello	Basidio	11	4	4	18	37	GCA_001972365.1	RIKEN Center for Life Science Technologies, Division of Genomic Technologies
1565	*Trichosporon faecale*	Tremello	Basidio	10	5	4	18	37	GCA_001752585.1	RIKEN Center for Life Science Technologies, Division of Genomic Technologies
1566	*Cerataphis brasiliensis yeast-like symbiont*	0	Asco	8	4	4	21	37	GCA_000372705.1	University of Arizona
1567	*Ustilago vetiveriae*	Ustilagino	Basidio	12	7	6	12	37	GCA_001735935.1	RWTH Aachen University
1568	*Russula virescens*	Agarico	Basidio	10	0	1	26	37	GCA_003316435.1	Kunming University of Science and Technology
1569	*Ascodesmis nigricans*	Pezizo	Asco	14	6	5	12	37	GCA_004786065.1	DOE Joint Genome Institute
1570	*Leucoagaricus gongylophorus*	Agarico	Basidio	12	3	7	15	37	GCA_000382605.1	UW-Madison
1571	*Ceratocystiopsis brevicomis*	Sordario	Asco	8	12	2	14	36	GCA_002778105.1	University of Montana
1572	*Blastomyces emzantsi*	Eurotio	Asco	10	5	7	14	36	GCA_003206725.1	National Institute for Communicable Diseases
1573	*Apiotrichum montevideense*	Tremello	Basidio	9	2	12	13	36	GCA_001598995.1	RIKEN Center for Life Science Technologies, Division of Genomic Technologies
1574	*Ramalina peruviana*	Lecanoro	Asco	10	4	2	20	36	GCA_001956345.1	Yunnan Academy of Forestry

1575	*Umbilicaria muehlenbergii*	Lecanoro	Asco	12	2	2	20	36	GCA_000611775.1	Seoul National University
1576	*Lasallia hispanica*	Lecanoro	Asco	10	2	2	22	36	GCA_003254425.1	Senckenberg
1577	*Cutaneotrichosporon oleaginosum*	Tremello	Basidio	7	4	8	17	36	GCA_008065305.1	Ruhr-University Bochum
1578	*Hebeloma cylindrosporum*	Agarico	Basidio	12	2	2	20	36	GCA_000827355.1	DOE Joint Genome Institute
1579	*Rhizopogon salebrosus*	Agarico	Basidio	12	2	2	20	36	GCA_002995475.1	Oregon State University
1580	*Lactarius echinatus*	Agarico	Basidio	10	1	1	24	36	GCA_003315975.1	Kunming University of Science and Technology
1581	*Tremella fuciformis*	Tremello	Basidio	15	4	7	9	35	GCA_000987905.1	Fujian Agriculture and Forestry University
1582	*Ramalina intermedia*	Lecanoro	Asco	10	3	2	20	35	GCA_003073195.1	Yunnan Academy of Forestry
1583	*Tuber calosporum*	Pezizo	Asco	12	8	1	14	35	GCA_003316355.1	Kunming University of Science and Technology
1584	*Chaetothyriales sp. CBS 134920*	Eurotio	Asco	12	3	2	18	35	GCA_003693665.1	Westerdijk Fungal Biodiversity Institute
1585	*Phycomyces blakesleeanus*	Mucoro	Mucoro	8	14	0	13	35	GCA_001638985.2	JGI-PSF
1586	*Cutaneotrichosporon cyanovorans*	Tremello	Basidio	9	4	5	17	35	GCA_002335625.1	RIKEN Center for Life Science Technologies, Division of Genomic Technologies
1587	*Mrakia frigida*	Tremello	Basidio	8	10	3	14	35	GCA_001600395.1	RIKEN Center for Life Science Technologies, Division of Genomic Technologies
1588	*Naganishia sp. IF7SW-B1*	Tremello	Basidio	10	12	5	8	35	GCA_012922685.1	Jet Propulsion Laboratory, California Institute of Technology
1589	*Naganishia adeliensis*	Tremello	Basidio	10	12	5	8	35	GCA_012922715.1	Jet Propulsion Laboratory, California Institute of Technology
1590	*Glomus cerebriforme*	Glomero	Mucoro	4	1	0	30	35	GCA_003833025.1	University of Ottawa
1591	*Lactarius volemus*	Agarico	Basidio	5	0	1	29	35	GCA_003315835.1	Kunming University of Science and Technology
1592	*Diversispora epigaea*	Glomero	Mucoro	4	1	0	30	35	GCA_003547095.1	Cornell University
1593	*Erythrobasidium hasegawianum*	Cystobasidio	Basidio	10	2	6	16	34	GCA_001972285.1	RIKEN Center for Life Science Technologies, Division of Genomic Technologies
1594	*Escovopsis sp. AC*	Sordario	Asco	15	4	5	10	34	GCA_003055955.1	University of East Anglia
1595	*Lichtheimia ramosa*	Mucoro	Mucoro	6	12	2	14	34	GCA_000945115.1	HKI JENA
1596	*Apophysomyces elegans*	Mucoro	Mucoro	16	0	0	18	34	GCA_000696995.1	IGS
1597	*Apophysomyces variabilis*	Mucoro	Mucoro	16	0	0	18	34	GCA_002749535.1	Postgraduate Institute of medical education and research
1598	*Choanephora cucurbitarum*	Mucoro	Mucoro	9	10	0	15	34	GCA_001683725.1	Korea University
1599	*Cryptococcus amylolentus*	Tremello	Basidio	9	8	4	13	34	GCA_001720205.1	Broad Institute
1600	*Cryptococcus floricola*	Tremello	Basidio	8	8	4	14	34	GCA_006352305.1	Duke University

1601	*Naganishia liquefaciens*	Tremello	Basidio	10	12	4	8	34	GCA_013423385.1	School of Life Science and Technology, Tokyo Institute of Technology
1602	*Hygrocybe conica*	Agarico	Basidio	4	6	2	22	34	GCA_900068975.1	Royal Botanic Gardens, Kew
1603	*Ascosphaera apis*	Eurotio	Asco	4	18	2	10	34	GCA_001636715.1	Shanghai Institutes for Biological Sciences, CAS
1604	*Escovopsis sp. Ae720*	Sordario	Asco	15	2	5	11	33	GCA_003055925.1	University of East Anglia
1605	*Escovopsis sp. Ae733*	Sordario	Asco	15	2	5	11	33	GCA_003055945.1	University of East Anglia
1606	*Lichtheimia corymbifera*	Mucoro	Mucoro	6	11	2	14	33	GCA_000697175.1	IGS
1607	*Tilletia indica*	Exobasidio	Basidio	11	2	2	18	33	GCA_001689995.1	ICAR-Indian Institute of Wheat and Barley Research, Karnal
1608	*Jimgerdemannia lactiflua*	Endogono	Mucoro	7	6	3	17	33	GCA_003951145.1	Oregon State University
1609	*Endocarpon pusillum*	Eurotio	Asco	12	2	0	19	33	GCA_000611755.1	Seoul National University
1610	*Terfezia boudieri*	Pezizo	Asco	11	8	6	8	33	GCA_003788615.2	DOE Joint Genome Institute
1611	*Alectoria sarmentosa*	Lecanoro	Asco	11	2	2	18	33	GCA_009733775.1	Universidad Complutense de Madrid
1612	*Nilaparvata lugens yeast-like symbiont*	0	Asco	8	4	4	17	33	GCA_000758425.1	Nilaparvata lugens Genome Consortium
1613	*Entoloma clypeatum*	Agarico	Basidio	12	4	1	16	33	GCA_900068945.1	Royal Botanic Gardens, Kew
1614	*Phlebopus portentosus*	Agarico	Basidio	6	2	1	24	33	GCA_000766925.2	Yunnan University
1615	*Tricholoma matsutake*	Agarico	Basidio	14	2	2	15	33	GCA_002939025.2	Korea University
1616	*Tilletia controversa*	Exobasidio	Basidio	8	7	1	16	32	GCA_001645045.2	Agriculture and Agri-Food Canada
1617	*Cystobasidiaceae sp. HBUAS51001*	Cystobasidio	Basidio	8	2	5	17	32	GCA_003351005.1	Hubei University of Arts and Science
1618	*Escovopsis sp. Ae724*	Sordario	Asco	14	2	5	11	32	GCA_003055165.1	University of East Anglia
1619	*Tolypocladium capitatum*	Sordario	Asco	10	4	6	12	32	GCA_002901185.1	Oregon State University
1620	*Chytriomyces confervae*	Chytridio	Chytridio	2	12	2	16	32	GCA_006535975.1	Agriculture and Agri-Food Canada
1621	*Tilletia caries*	Exobasidio	Basidio	8	7	1	16	32	GCA_001645005.2	Agriculture and Agri-Food Canada
1622	*Trichosporon inkin*	Tremello	Basidio	8	4	2	18	32	GCA_001752625.1	RIKEN Center for Life Science Technologies, Division of Genomic Technologies
1623	*Thecaphora thlaspeos*	Ustilagino	Basidio	9	10	1	12	32	GCA_900260175.1	RWTH Aachen University
1624	*Lasallia pustulata*	Lecanoro	Asco	10	2	2	18	32	GCA_008636195.1	University of Frankfurt
1625	*Emmonsia crescens*	Eurotio	Asco	10	6	4	12	32	GCA_002572855.1	Broad Institute
1626	*Rhodotorula paludigena*	Microbotryo	Basidio	8	4	4	16	32	GCA_005281665.1	Suranaree University of Technology
1627	*Kwoniella shandongensis*	Tremello	Basidio	8	8	4	12	32	GCA_008629635.1	Broad Institute
1628	*Microbotryum silenes-dioicae*	Microbotryo	Basidio	8	5	1	18	32	GCA_900120095.1	ESE
1629	*Absidia repens*	Mucoro	Mucoro	8	1	0	22	31	GCA_002105175.1	DOE Joint Genome Institute
1630	*Rhizomucor pusillus*	Mucoro	Mucoro	7	8	0	16	31	GCA_900175165.2	CHALMERS UNIVERSITY OF TECHNOLOGY

1631	*Ustilago esculenta*	Ustilagino	Basidio	8	5	2	16	31	GCA_000819925.1	Zhejiang Provincial Key Laboratory of Biometrology and Inspection & Quarantine
1632	*Emergomyces orientalis*	Eurotio	Asco	11	5	3	12	31	GCA_002110485.1	Beijing Institute of Radiation Medicine
1633	*Laccaria bicolor*	Agarico	Basidio	6	1	0	24	31	GCA_000143565.1	Laccaria Genome Consortium
1634	*Smittium culicis*	Harpello	Zoopago	1	0	0	30	31	GCA_001970855.1	University of Toronto
1635	*Hygrophorus pudorinus*	Agarico	Basidio	6	4	1	20	31	GCA_003314045.1	Kunming University of Science and Technology
1636	*Melampsora pinitorqua*	Puccinio	Basidio	6	12	5	8	31	GCA_000464645.1	Tree Aggressors Identification using Genomic Approaches
1637	*Lactifluus rugatus*	Agarico	Basidio	7	1	2	21	31	GCA_003315895.1	Kunming University of Science and Technology
1638	*Russula lepida*	Agarico	Basidio	8	0	2	21	31	GCA_003316425.1	Kunming University of Science and Technology
1639	*Mucor velutinosus*	Mucoro	Mucoro	6	2	2	20	30	GCA_000696895.1	IGS
1640	*Mucor circinelloides*	Mucoro	Mucoro	8	2	0	20	30	GCA_001599575.1	RIKEN Center for Life Science Technologies, Division of Genomic Technologies
1641	*Mucor irregularis*	Mucoro	Mucoro	8	1	1	20	30	GCA_000587855.1	Nanjing Biozeron
1642	*Jimgerdemannia flammicorona*	Endogono	Mucoro	3	6	4	17	30	GCA_003990745.1	DOE Joint Genome Institute
1643	*Chaetothyriales sp. CBS 134916*	Eurotio	Asco	10	0	2	18	30	GCA_003709845.1	Westerdijk Fungal Biodiversity Institute
1644	*Apophysomyces trapeziformis*	Mucoro	Mucoro	12	0	0	18	30	GCA_000696975.1	IGS
1645	*Chaetothyriales sp. CBS 135597*	Eurotio	Asco	10	3	1	16	30	GCA_003709825.1	Westerdijk Fungal Biodiversity Institute
1646	*Thermomyces lanuginosus*	Eurotio	Asco	11	4	3	12	30	GCA_000315935.1	Durban University of Technology
1647	*Naganishia albida*	Tremello	Basidio	8	8	4	10	30	GCA_001599735.1	RIKEN Center for Life Science Technologies, Division of Genomic Technologies
1648	*Blastomyces silverae*	Eurotio	Asco	10	5	3	12	30	GCA_001014755.1	Broad Institute
1649	*Emergomyces pasteurianus*	Eurotio	Asco	11	4	2	13	30	GCA_001883825.1	Broad Institute
1650	*Rhizopogon fuscorubens*	Agarico	Basidio	8	0	2	20	30	GCA_002995455.1	Oregon State University
1651	*Rhizophagus irregularis*	Glomero	Mucoro	3	1	0	26	30	GCA_002897155.1	NIBB core research facilities, National Institues for Basic Biology
1652	*Tricholoma terreum*	Agarico	Basidio	6	2	2	20	30	GCA_003316345.1	Kunming University of Science and Technology
1653	*Rhizophagus clarus*	Glomero	Mucoro	6	1	0	23	30	GCA_003203555.1	National Institute for Basic Biology
1654	*Pyrrhoderma noxium*	Agarico	Basidio	9	4	0	17	30	GCA_002287475.2	Academia Sinica
1655	*Chrysosporium queenslandicum*	Eurotio	Asco	8	1	2	18	29	GCA_001430955.1	UC Berkeley
1656	*Cryptococcus wingfieldii*	Tremello	Basidio	4	8	4	13	29	GCA_001720155.1	Broad Institute

1657	*Erythrobasidium yunnanense*	Cystobasidio	Basidio	8	4	5	12	29	GCA_001600175.1	RIKEN Center for Life Science Technologies, Division of Genomic Technologies
1658	*Naganishia randhawae*	Tremello	Basidio	8	10	2	9	29	GCA_013461525.1	Wits University
1659	*Microbotryum saponariae*	Microbotryo	Basidio	8	3	1	17	29	GCA_900102585.1	ESE
1660	*Tricholoma sp. MG99*	Agarico	Basidio	11	0	1	17	29	GCA_003521275.1	Kunming University of Science and Technology
1661	*Laccaria amethystina*	Agarico	Basidio	8	1	2	18	29	GCA_000827195.1	DOE Joint Genome Institute
1662	*Allomyces macrogynus*	Blastocladio	Blastocladio	2	7	9	10	28	GCA_000151295.1	Broad Institute
1663	*Amauroascus niger*	Eurotio	Asco	8	2	1	17	28	GCA_001430945.1	UC Berkeley
1664	*Rhizomucor miehei*	Mucoro	Mucoro	6	8	2	12	28	GCA_000611695.1	Department of Biotechnology, College of Food Science and Nutritional Engineering, China Agricultural University
1665	*Rhizopus microsporus*	Mucoro	Mucoro	12	6	0	10	28	GCA_006680115.1	TGen-North
1666	*Thermomucor indicae-seudaticae*	Mucoro	Mucoro	4	8	2	14	28	GCA_000787465.1	Aalborg University
1667	*Rhodotorula graminis*	Microbotryo	Basidio	6	5	5	12	28	GCA_001329695.1	DOE Joint Genome Institute
1668	*Ceraceosorus guamensis*	Exobasidio	Basidio	8	4	1	15	28	GCA_003144195.1	DOE Joint Genome Institute
1669	*Blastomyces parvus*	Eurotio	Asco	10	5	3	10	28	GCA_002572885.1	Broad Institute
1670	*Cutaneotrichosporon curvatum*	Tremello	Basidio	8	4	4	12	28	GCA_001600275.1	RIKEN Center for Life Science Technologies, Division of Genomic Technologies
1671	*Coemansia reversa*	Kickxello	Zoopago	4	2	0	22	28	GCA_002705745.1	JGI
1672	*Cetradonia linearis*	Lecanoro	Asco	7	2	1	18	28	GCA_003521265.1	The New York Botanical Garden
1673	*Blyttiomyces helicus*	Chytridio	Chytridio	6	4	0	18	28	GCA_003614705.1	DOE Joint Genome Institute
1674	*Microbotryum silenes-acaulis*	Microbotryo	Basidio	6	5	1	16	28	GCA_003665825.1	Universite Paris Sud
1675	*Naematelia encephala*	Tremello	Basidio	4	12	4	8	28	GCA_002105065.1	DOE Joint Genome Institute
1676	*Filobasidium wieringae*	Tremello	Basidio	10	6	6	6	28	GCA_001600055.1	RIKEN Center for Life Science Technologies, Division of Genomic Technologies
1677	*Chaetothyriales sp. CBS 132003*	Eurotio	Asco	8	0	1	18	27	GCA_003709865.1	Westerdijk Fungal Biodiversity Institute
1678	*Ceraceosorus bombacis*	Exobasidio	Basidio	8	3	1	15	27	GCA_900000165.1	IPF
1679	*Blastomyces percursus*	Eurotio	Asco	8	7	2	10	27	GCA_001883805.1	Broad Institute
1680	*Scleroderma citrinum*	Agarico	Basidio	4	1	5	17	27	GCA_000827425.1	DOE Joint Genome Institute
1681	*Microbotryum intermedium*	Microbotryo	Basidio	6	4	1	16	27	GCA_900096595.1	ESE
1682	*Mucor lusitanicus*	Mucoro	Mucoro	6	2	0	18	26	GCA_010203745.1	DOE Joint Genome Institute
1683	*Drechmeria coniospora*	Sordario	Asco	8	5	1	12	26	GCA_001625195.1	Biotechnology Research Institute, Chinese Academy of Agricultural Sciences
1684	*Parasitella parasitica*	Mucoro	Mucoro	6	1	1	18	26	GCA_000938895.1	FRIEDRICH SCHILLER UNIVERSITY JENA
1685	*Aspergillus cejpii*	Eurotio	Asco	10	4	0	12	26	GCA_004769165.1	Guangdong Institute of Microbiology

1686	*Antarctomyces pellizariae*	Leotio	Asco	13	5	0	8	26	GCA_010623925.1	UFMG - Universidade Federal de Minas Gerais
1687	*Histoplasma capsulatum*	Eurotio	Asco	9	3	2	12	26	GCA_000313325.1	Taipei Medical University
1688	*Blastomyces gilchristii*	Eurotio	Asco	10	4	2	10	26	GCA_000003855.2	Broad Institute
1689	*Tricholoma sp. MG77*	Agarico	Basidio	6	2	0	18	26	GCA_003314665.1	Kunming University of Science and Technology
1690	*Sarcodon aspratus*	Agarico	Basidio	6	2	5	13	26	GCA_003313825.1	Kunming University of Science and Technology
1691	*Kwoniella bestiolae*	Tremello	Basidio	4	8	4	10	26	GCA_000512585.2	Broad Institute
1692	*Basidiobolus heterosporus*	Basidiobolo	Zoopago	6	0	1	18	25	GCA_000697455.1	IGS
1693	*Mucor ambiguus*	Mucoro	Mucoro	7	2	0	16	25	GCA_000950595.1	National Institute of Advanced Industrial Science and Technology (AIST)
1694	*Actinomucor elegans*	Mucoro	Mucoro	6	2	0	17	25	GCA_001599635.1	RIKEN Center for Life Science Technologies, Division of Genomic Technologies
1695	*Quambalaria eucalypti*	Exobasidio	Basidio	4	8	1	12	25	GCA_004016185.1	University of Pretoria
1696	*Jaminaea rosea*	Exobasidio	Basidio	6	8	1	10	25	GCA_003144245.1	DOE Joint Genome Institute
1697	*Holtermannia corniformis*	Tremello	Basidio	9	4	1	11	25	GCA_001599935.1	RIKEN Center for Life Science Technologies, Division of Genomic Technologies
1698	*Cryptococcus neoformans AD hybrid*	Tremello	Basidio	5	2	0	18	25	GCA_006992865.1	Weill Cornell Medicine
1699	*Kwoniella dejecticola*	Tremello	Basidio	5	8	2	10	25	GCA_000512565.2	Broad Institute
1700	*Salmacisia buchloeana*	Exobasidio	Basidio	9	2	0	13	24	GCA_001990185.1	Pennsylvania State University
1701	*Entomophthora muscae*	Entomophthoro	Zoopago	6	0	0	18	24	GCA_900018355.1	UNIVERSITY OF COPENHAGEN
1702	*Saksenaea vasiformis*	Mucoro	Mucoro	8	0	0	16	24	GCA_000697055.1	IGS
1703	*Saksenaea oblongispora*	Mucoro	Mucoro	8	0	0	16	24	GCA_000697495.1	IGS
1704	*Cystobasidiopsis lactophilus*	Agaricostilbo	Basidio	6	1	1	16	24	GCA_001599975.1	RIKEN Center for Life Science Technologies, Division of Genomic Technologies
1705	*Endogone sp. FLAS-F59071*	Endogono	Mucoro	4	3	1	16	24	GCA_003990785.1	DOE Joint Genome Institute
1706	*Blastomyces dermatitidis*	Eurotio	Asco	8	4	2	10	24	GCA_000003525.2	Broad Institute
1707	*Rhodotorula toruloides*	Microbotryo	Basidio	6	1	3	14	24	GCA_001255795.1	UCB
1708	*Pseudomicrostroma glucosiphilum*	Exobasidio	Basidio	8	2	4	10	24	GCA_003144135.1	DOE Joint Genome Institute
1709	*Microbotryum violaceum*	Microbotryo	Basidio	5	3	1	15	24	GCA_900015485.1	INRA-LIPM
1710	*Tricholoma flavovirens*	Agarico	Basidio	5	0	2	17	24	GCA_003313805.1	Kunming University of Science and Technology
1711	*Oehlia diaphana*	Glomero	Mucoro	3	1	0	20	24	GCA_003833135.1	University of Ottawa
1712	*Mortierella elongata*	Mortierello	Mucoro	4	4	1	14	23	GCA_001651415.1	DOE Joint Genome Institute
1713	*Tilletiopsis washingtonensis*	Exobasidio	Basidio	4	5	2	12	23	GCA_003144115.1	DOE Joint Genome Institute
1714	*Microsporum canis*	Eurotio	Asco	6	2	1	14	23	GCA_000151145.1	Broad Institute
1715	*Xeromyces bisporus*	Eurotio	Asco	7	5	3	8	23	GCA_900006255.1	UPPSALA UNIVERSITET
1716	*Rhodotorula sp. ZM1*	Microbotryo	Basidio	6	0	3	14	23	GCA_009806315.1	Zhejiang Normal University

1717	*Tricholoma bakamatsutake*	Agarico	Basidio	9	0	1	13	23	GCA_003313665.1	Kunming University of Science and Technology
1718	*Holtermanniella nyarrowii*	Tremello	Basidio	7	7	2	7	23	GCA_001600035.1	RIKEN Center for Life Science Technologies, Division of Genomic Technologies
1719	*Hygrophorus russula*	Agarico	Basidio	2	1	2	18	23	GCA_003314125.1	Kunming University of Science and Technology
1720	*Rhizophagus sp. MUCL 43196*	Glomero	Mucoro	3	1	0	19	23	GCA_003549995.1	INRA
1721	*Phellinus lamaoensis*	Agarico	Basidio	6	2	1	14	23	GCA_002794735.1	Academia Sinica
1722	*Mortierella verticillata*	Mortierello	Mucoro	4	2	0	16	22	GCA_000739165.1	Broad Institute
1723	*Mortierella alpina*	Mortierello	Mucoro	4	2	2	14	22	GCA_000507065.1	IGS
1724	*Tilletia walkeri*	Exobasidio	Basidio	6	3	1	12	22	GCA_009428295.1	Agriculture and Agri-Food Canada
1725	*Cunninghamella bertholletiae*	Mucoro	Mucoro	6	2	0	14	22	GCA_000697215.1	IGS
1726	*Nannizzia gypsea*	Eurotio	Asco	7	0	0	15	22	GCA_000150975.2	Broad Institute
1727	*Cokeromyces recurvatus*	Mucoro	Mucoro	4	0	0	18	22	GCA_000697235.1	IGS
1728	*Emmonsia sp. CAC-2015a*	Eurotio	Asco	8	2	2	10	22	GCA_001660665.1	Broad Institute
1729	*Symmetrospora coprosmae*	Cystobasidio	Basidio	7	4	2	9	22	GCA_008802785.1	University College Dublin
1730	*Glaciozyma antarctica*	Microbotryo	Basidio	6	0	2	14	22	GCA_002917775.1	Malaysia Genome Institute
1731	*Cryptococcus neoformans*	Tremello	Basidio	5	4	2	11	22	GCA_002216725.1	Washington University in St. Louis
1732	*Phaeotremella skinneri*	Tremello	Basidio	4	5	3	10	22	GCA_001599695.1	RIKEN Center for Life Science Technologies, Division of Genomic Technologies
1733	*Puccinia novopanici*	Puccinio	Basidio	10	0	0	12	22	GCA_004348175.1	Noble Research Institute
1734	*Takashimella tepidaria*	Tremello	Basidio	3	7	4	8	22	GCA_003116915.1	RIKEN Center for Life Science Technologies, Division of Genomic Technologies
1735	*fungal sp. Mo6-1*	0	0	6	2	5	8	21	GCA_002939055.1	University of Montana
1736	*Absidia glauca*	Mucoro	Mucoro	4	1	1	15	21	GCA_900079185.1	FRIEDRICH SCHILLER UNIVERSITY JENA
1737	*Amauroascus mutatus*	Eurotio	Asco	6	2	1	12	21	GCA_001430935.1	UC Berkeley
1738	*Hesseltinella vesiculosa*	Mucoro	Mucoro	4	0	0	17	21	GCA_002104935.1	DOE Joint Genome Institute
1739	*Conidiobolus coronatus*	Entomophthoro	Zoopago	5	1	0	15	21	GCA_001566745.1	JGI-PSF
1740	*Rhodotorula diobovata*	Microbotryo	Basidio	5	2	2	12	21	GCA_006352295.1	University of Manitoba
1741	*Tilletiaria anomala*	Exobasidio	Basidio	6	3	0	12	21	GCA_000711695.1	DOE Joint Genome Institute
1742	*Puccinia coronata*	Puccinio	Basidio	6	0	0	15	21	GCA_002873275.1	University of Minnesota
1743	*Cryptococcus gattii VGII*	Tremello	Basidio	4	3	1	13	21	GCA_003011995.1	Jawaharlal Nehru Centre for Advanced Scientific Research
1744	*Puccinia graminis*	Puccinio	Basidio	9	0	0	12	21	GCA_008522505.1	University of Minnesota
1745	*Synchytrium endobioticum*	Chytridio	Chytridio	6	2	0	13	21	GCA_006536045.1	Agriculture and Agri-Food Canada
1746	*Amanita muscaria*	Agarico	Basidio	4	1	0	16	21	GCA_000827485.1	DOE Joint Genome Institute
1747	*Cunninghamella elegans*	Mucoro	Mucoro	6	2	0	12	20	GCA_000697015.1	IGS

1748	*Arthroderma uncinatum*	Eurotio	Asco	6	0	1	13	20	GCA_011692745.1	Chinese Academy of Medical Science and Peking Union Medical College
1749	*Mrakia blollopis*	Tremello	Basidio	4	2	4	10	20	GCA_000950635.1	National Institute of Advanced Industrial Science and Technology (AIST)
1750	*Piedraia hortae*	Dothideo	Asco	2	2	4	12	20	GCA_010093745.1	DOE Joint Genome Institute
1751	*Mrakia psychrophila*	Tremello	Basidio	4	4	4	8	20	GCA_001889225.1	Institute Of Microbiology Chinese Academy of Sciences
1752	*Protomyces lactucaedebilis*	Taphrino	Asco	4	6	2	8	20	GCA_002105105.1	DOE Joint Genome Institute
1753	*Paracoccidioides lutzii*	Eurotio	Asco	7	1	2	10	20	GCA_000150705.2	Broad Institute
1754	*Protomyces macrosporus*	Taphrino	Asco	4	6	2	8	20	GCA_003717175.1	University of Helsinki
1755	*Zoophagus insidians*	Zoopago	Zoopago	0	10	0	10	20	GCA_004114325.1	University of Michigan
1756	*Trichophyton mentagrophytes*	Eurotio	Asco	5	2	0	12	19	GCA_003664465.1	North-Western State Medical University named after I.I. Mechnicov
1757	*Trichophyton benhamiae*	Eurotio	Asco	5	2	0	12	19	GCA_001858085.1	Swiss Institute of Bioinformatics
1758	*Trichophyton equinum*	Eurotio	Asco	5	0	0	14	19	GCA_000151175.1	Broad Institute
1759	*Trichophyton tonsurans*	Eurotio	Asco	5	0	0	14	19	GCA_000151455.1	Broad Institute
1760	*Protomyces inouyei*	Taphrino	Asco	4	4	3	8	19	GCA_003717155.1	University of Helsinki
1761	*Saitoella complicata*	0	Asco	6	2	1	10	19	GCA_000227095.3	Saitoella complicata genome sequencing consortium
1762	*Malassezia furfur*	Malassezio	Basidio	6	1	0	12	19	GCA_002551515.1	University of Utrecht
1763	*Onygena corvina*	Eurotio	Asco	6	0	0	12	18	GCA_000812245.1	Aalborg University
1764	*Coccidioides immitis*	Eurotio	Asco	6	1	1	10	18	GCA_004115165.2	TGen North
1765	*Paracoccidioides brasiliensis*	Eurotio	Asco	6	1	2	9	18	GCA_000150475.2	Broad Institute
1766	*Cryptococcus gattii VGIV*	Tremello	Basidio	4	1	1	12	18	GCA_000835755.1	Broad Institute
1767	*Pisolithus tinctorius*	Agarico	Basidio	3	0	1	14	18	GCA_000827335.1	DOE Joint Genome Institute
1768	*Amanita brunnescens*	Agarico	Basidio	4	4	1	9	18	GCA_001691785.2	Harvard University
1769	*Trichophyton verrucosum*	Eurotio	Asco	5	0	0	12	17	GCA_000151505.1	Arthroderma Genome Sequencing Consortium
1770	*Trichophyton rubrum*	Eurotio	Asco	5	0	0	12	17	GCA_000616845.1	Broad Institute
1771	*Trichophyton soudanense*	Eurotio	Asco	5	0	0	12	17	GCA_000616865.1	Broad Institute
1772	*Trichophyton interdigitale*	Eurotio	Asco	5	0	0	12	17	GCA_000622975.1	Broad Institute
1773	*Trichophyton kuryangei*	Eurotio	Asco	5	0	0	12	17	GCA_012184535.1	Belgian Coordinated Collection of Microorganisms, BCCM
1774	*Trichophyton violaceum*	Eurotio	Asco	5	0	0	12	17	GCA_001651435.1	Chinese Academy of Medical Science & Peking Union Medical College
1775	*Trichophyton yaoundei*	Eurotio	Asco	5	0	0	12	17	GCA_012184575.1	Belgian Coordinated Collection of Microorganisms, BCCM
1776	*Byssoonygena ceratinophila*	Eurotio	Asco	6	1	0	10	17	GCA_001430925.1	UC Berkeley
1777	*Rhodotorula mucilaginosa*	Microbotryo	Basidio	4	3	0	10	17	GCA_003055205.1	Indian Institute Of Chemical biology

1778	*Rhodotorula sp. JG-1b*	Microbotryo	Basidio	3	3	1	10	17	GCA_001541205.1	DOE Joint Genome Institute
1779	*Thelephora aurantiotincta*	Agarico	Basidio	4	0	3	10	17	GCA_003316405.1	Kunming University of Science and Technology
1780	*Cryptococcus cf. gattii*	Tremello	Basidio	4	2	1	10	17	GCA_009650685.1	Broad Institute
1781	*Cryptococcus gattii VGIII*	Tremello	Basidio	4	2	1	10	17	GCA_000836335.1	Broad Institute
1782	*Pisolithus microcarpus*	Agarico	Basidio	2	2	1	12	17	GCA_000827275.1	JGI
1783	*Sarcodon sp. MG97*	Agarico	Basidio	5	2	1	9	17	GCA_003313065.1	Kunming University of Science and Technology
1784	*Xylona heveae*	Xylono	Asco	6	0	0	10	16	GCA_001619985.1	DOE Joint Genome Institute
1785	*Uncinocarpus reesii*	Eurotio	Asco	6	1	1	8	16	GCA_000003515.2	Broad Institute
1786	*Taphrina betulina*	Taphrino	Asco	4	2	2	8	16	GCA_008802775.1	University College Dublin
1787	*Protomyces sp. C29*	Taphrino	Asco	4	4	0	8	16	GCA_003568695.1	University of Helsinki
1788	*Protomyces gravidus*	Taphrino	Asco	4	4	0	8	16	GCA_003717255.1	University of Helsinki
1789	*Cryptococcus gattii VGI*	Tremello	Basidio	3	1	1	11	16	GCA_000836355.1	Broad Institute
1790	*Wallemia hederae*	Wallemio	Basidio	7	2	0	7	16	GCA_004918325.1	Biotechnical Faculty, University of Ljubljana
1791	*Malassezia globosa*	Malassezio	Basidio	4	0	0	12	16	GCA_001264805.1	Genome Institute of Singapore
1792	*Caulochytrium protostelioides*	Chytridio	Chytridio	2	2	3	8	15	GCA_003615045.1	DOE Joint Genome Institute
1793	*Podosphaera leucotricha*	Leotio	Asco	2	2	2	9	15	GCA_013170925.1	Washington State University
1794	*Rhodotorula taiwanensis*	Microbotryo	Basidio	3	1	1	10	15	GCA_002922495.1	Lawrence Livermore National Laboratory
1795	*Taphrina sp. SM11*	Taphrino	Asco	4	2	2	7	15	GCA_004000165.1	University of Helsinki
1796	*Erysiphe pulchra*	Leotio	Asco	2	6	2	5	15	GCA_002918395.1	USDA-ARS
1797	*Kwoniella mangrovensis*	Tremello	Basidio	2	3	2	8	15	GCA_000507485.3	Broad Institute
1798	*Lobosporangium transversale*	Mortierello	Mucoro	2	2	0	10	14	GCA_002105155.1	DOE Joint Genome Institute
1799	*Spizellomyces sp. 'palustris'*	Chytridio	Chytridio	5	0	0	9	14	GCA_006535965.1	Agriculture and Agri-Food Canada
1800	*Protomyces pachydermus*	Taphrino	Asco	4	4	0	6	14	GCA_003717275.1	University of Helsinki
1801	*Taphrina pruni*	Taphrino	Asco	2	5	1	6	14	GCA_005281585.1	Northwest A&F University
1802	*Taphrina flavorubra*	Taphrino	Asco	2	5	1	6	14	GCA_000836175.1	University of Miyazaki
1803	*Golovinomyces cichoracearum*	Leotio	Asco	2	3	2	7	14	GCA_003611215.1	University of Maryland
1804	*Golubevia sp. BC0812*	Exobasidio	Basidio	3	2	0	9	14	GCA_012976215.1	Wageningen UR
1805	*Amanita phalloides*	Agarico	Basidio	4	0	0	10	14	GCA_001983385.1	Michigan State University
1806	*Malassezia caprae*	Malassezio	Basidio	4	0	0	10	14	GCA_001264625.1	Genome Institute of Singapore
1807	*Rhodotorula sp. CCFEE 5036*	Microbotryo	Basidio	4	1	0	8	13	GCA_005059875.1	University of California, Riverside
1808	*Taphrina confusa*	Taphrino	Asco	2	4	1	6	13	GCA_005281535.1	Northwest A&F University
1809	*Blumeria graminis*	Leotio	Asco	1	3	1	8	13	GCA_900237765.1	MAX PLANCK INSTITUTE FOR PLANT BREEDING RESEARCH
1810	*Ophidiomyces ophiodiicola*	Eurotio	Asco	5	0	0	8	13	GCA_002167195.1	University of Arizona
1811	*Taphrina communis*	Taphrino	Asco	2	4	1	6	13	GCA_005281525.1	Northwest A&F University
1812	*Taphrina deformans*	Taphrino	Asco	4	2	1	6	13	GCA_005281805.1	Northwest A&F University
1813	*Golubevia sp. BC0902*	Exobasidio	Basidio	3	2	0	8	13	GCA_012976205.1	Wageningen UR
1814	*Sporidiobolus pararoseus*	Microbotryo	Basidio	4	0	1	8	13	GCA_010758995.1	Shenyang Agricultral University

1815	*Malassezia obtusa*	Malassezio	Basidio	3	1	0	9	13	GCA_001264985.1	Genome Institute of Singapore
1816	*Mortierella sp. BCC40632*	Mortierello	Mucoro	2	0	0	10	12	GCA_011634665.1	National Center for Genetic Engineering and Biotechnology
1817	*Taphrina wiesneri*	Taphrino	Asco	3	3	0	6	12	GCA_005281515.1	Northwest A&F University
1818	*Spizellomyces punctatus*	Chytridio	Chytridio	4	0	0	8	12	GCA_000182565.2	Broad Institute
1819	*Mixia osmundae*	Mixio	Basidio	2	0	0	10	12	GCA_000708205.1	DOE Joint Genome Institute
1820	*Zoopage sp. CT-All*	Zoopago	Zoopago	0	0	4	8	12	GCA_004114245.1	University of Michigan
1821	*Malassezia japonica*	Malassezio	Basidio	3	0	0	9	12	GCA_001600795.1	RIKEN Center for Life Science Technologies, Division of Genomic Technologies
1822	*Golubevia sp. BC0850*	Exobasidio	Basidio	2	2	0	8	12	GCA_012976225.1	Wageningen UR
1823	*Amanita bisporigera*	Agarico	Basidio	4	0	0	8	12	GCA_001983365.1	Michigan State University
1824	*Malassezia equina*	Malassezio	Basidio	2	0	0	10	12	GCA_001264685.1	Genome Institute of Singapore
1825	*Neolecta irregularis*	Neolecto	Asco	3	2	2	5	12	GCA_001929475.1	University of California, Riverside
1826	*Malassezia nana*	Malassezio	Basidio	3	0	0	9	12	GCA_001600835.1	RIKEN Center for Life Science Technologies, Division of Genomic Technologies
1827	*Malassezia dermatis*	Malassezio	Basidio	2	0	0	10	12	GCA_001600775.1	RIKEN Center for Life Science Technologies, Division of Genomic Technologies
1828	*Puccinia striiformis*	Puccinio	Basidio	8	1	1	2	12	GCA_011750755.1	Australian National University
1829	*Golubevia pallescens*	Exobasidio	Basidio	3	3	0	6	12	GCA_001599655.1	RIKEN Center for Life Science Technologies, Division of Genomic Technologies
1830	*Smittium angustum*	Harpello	Zoopago	0	0	0	11	11	GCA_003097675.1	University of Toronto
1831	*Furculomyces boomerangus*	Harpello	Zoopago	0	0	0	11	11	GCA_003086725.1	University of Toronto
1832	*Taphrina populina*	Taphrino	Asco	3	3	0	5	11	GCA_000836195.1	University of Miyazaki
1833	*fungal sp. ARF18*	0	0	4	0	0	7	11	GCA_002224055.1	University of Arkansas
1834	*Puccinia triticina*	Puccinio	Basidio	6	0	0	5	11	GCA_013090125.1	University of Sydney
1835	*Malassezia cuniculi*	Malassezio	Basidio	3	0	0	8	11	GCA_001264635.1	Genome Institute of Singapore
1836	*Malassezia slooffiae*	Malassezio	Basidio	3	0	0	8	11	GCA_010577765.1	Jawaharlal Nehru Centre for Advanced Scientific Research
1837	*Amanita pseudoporphyria*	Agarico	Basidio	4	0	0	7	11	GCA_003316615.1	Kunming University of Science and Technology
1838	*Smittium mucronatum*	Harpello	Zoopago	0	2	1	7	10	GCA_001953115.1	University of Toronto
1839	*Puccinia sorghi*	Puccinio	Basidio	4	0	0	6	10	GCA_001263375.1	BIA - FCEN - UBA
1840	*Piptocephalis cylindrospora*	Zoopago	Zoopago	1	0	0	9	10	GCA_003614145.1	DOE Joint Genome Institute
1841	*Malassezia sympodialis*	Malassezio	Basidio	2	0	0	8	10	GCA_001264715.1	Genome Institute of Singapore
1842	*Malassezia pachydermatis*	Malassezio	Basidio	3	0	0	7	10	GCA_001264975.1	Genome Institute of Singapore
1843	*Amanita polypyramis*	Agarico	Basidio	2	0	0	8	10	GCA_001691755.2	Harvard University
1844	*Kwoniella pini*	Tremello	Basidio	2	2	2	4	10	GCA_000512605.2	Broad Institute
1845	*Massospora cicadina*	Entomophthoro	Zoopago	2	0	0	7	9	GCA_006912075.1	University of Michigan

1846	*Massospora platypediae*	Entomophthoro	Zoopago	4	0	0	5	9	GCA_006912095.1	University of California, Riverside
1847	*Thamnocephalis sphaerospora*	Zoopago	Zoopago	0	0	2	7	9	GCA_003614735.1	DOE Joint Genome Institute
1848	*Oidium heveae*	Leotio	Asco	1	2	0	6	9	GCA_003957845.1	Hainan University
1849	*Amanita inopinata*	Agarico	Basidio	2	0	0	7	9	GCA_001691775.3	Harvard University
1850	*Acaulopage tetraceros*	Zoopago	Zoopago	1	0	0	8	9	GCA_004114255.1	University of Michigan
1851	*Wallemia ichthyophaga*	Wallemio	Basidio	4	1	0	4	9	GCA_004918895.1	Biotechnical Faculty, University of Ljubljana
1852	*Amanita jacksonii*	Agarico	Basidio	1	0	0	8	9	GCA_000497225.1	University of Toronto
1853	*Malassezia vespertilionis*	Malassezio	Basidio	2	0	0	7	9	GCA_002818225.1	US Forest Service
1854	*Homolaphlyctis polyrhiza*	Chytridio	Chytridio	0	0	0	8	8	GCA_000235945.1	University of Idaho, Initiative for Bioinformatics and Evolutionary Studies
1855	*Capniomyces stellatus*	Harpello	Zoopago	0	0	0	8	8	GCA_001661515.1	University of Toronto
1856	*Smittium simulii*	Harpello	Zoopago	0	0	0	8	8	GCA_003086735.1	University of Toronto
1857	*Oidium neolycopersici*	Leotio	Asco	2	1	0	5	8	GCA_003610855.1	University of Maryland
1858	*Cystobasidium pallidum*	Cystobasidio	Basidio	2	0	2	4	8	GCA_001599955.1	RIKEN Center for Life Science Technologies, Division of Genomic Technologies
1859	*uncultured Malassezia*	Malassezio	Basidio	2	0	0	6	8	GCA_903798065.1	EBI
1860	*Malassezia restricta*	Malassezio	Basidio	2	0	0	6	8	GCA_001264725.1	Genome Institute of Singapore
1861	*Puccinia hordei*	Puccinio	Basidio	4	0	0	4	8	GCA_007896445.1	University of Sydney
1862	*Malassezia yamatoensis*	Malassezio	Basidio	2	0	0	6	8	GCA_001264885.1	Genome Institute of Singapore
1863	*Catenaria anguillulae*	Blastocladio	Blastocladio	0	1	2	4	7	GCA_002102555.1	DOE Joint Genome Institute
1864	*Zancudomyces culisetae*	Harpello	Zoopago	2	1	0	4	7	GCA_001969505.1	University of Toronto
1865	*Batrachochytrium dendrobatidis*	Chytridio	Chytridio	1	0	0	6	7	GCA_003595275.1	University of Otago
1866	*Smittium megazygosporum*	Harpello	Zoopago	0	0	0	7	7	GCA_003086715.1	University of Toronto
1867	*Syncephalis pseudoplumigaleata*	Zoopago	Zoopago	2	1	0	4	7	GCA_003614755.1	DOE Joint Genome Institute
1868	*Cryptococcus depauperatus*	Tremello	Basidio	2	0	1	4	7	GCA_001720245.1	Broad Institute
1869	*Puccinia horiana*	Puccinio	Basidio	3	0	0	4	7	GCA_001624995.1	National Academy of Agricultural Science
1870	*Phaeotremella fagi*	Tremello	Basidio	0	0	3	4	7	GCA_001599715.1	RIKEN Center for Life Science Technologies, Division of Genomic Technologies
1871	*Dimargaris cristalligena*	Dimargarito	Zoopago	1	1	0	4	6	GCA_003614675.1	DOE Joint Genome Institute
1872	*Erysiphe pisi*	Leotio	Asco	1	1	0	4	6	GCA_000208805.1	Max-Planck-Institute for Plant Breeding Research
1873	*Malassezia sp.*	Malassezio	Basidio	2	0	0	4	6	GCA_004026415.1	Jill Banfield's Lab at Berkeley
1874	*Batrachochytrium salamandrivorans*	Chytridio	Chytridio	0	0	0	5	5	GCA_002006685.1	Broad Institute
1875	*Erysiphe necator*	Leotio	Asco	1	0	0	4	5	GCA_000798715.1	University of California, Davis
1876	*Phaffia rhodozyma*	Tremello	Basidio	0	1	0	4	5	GCA_001600435.1	RIKEN Center for Life Science Technologies, Division of Genomic Technologies

1877	*Cronartium ribicola*	Puccinio	Basidio	3	0	0	2	5	GCA_000500245.1	Tree Aggressors Identification using Genomic Approaches
1878	*Cochlonema odontosperma*	Zoopago	Zoopago	0	0	0	4	4	GCA_004114315.1	University of Michigan
1879	*Stylopage hadra*	Zoopago	Zoopago	0	1	0	3	4	GCA_005111325.1	University of Michigan
1880	*Rozella allomycis*	0	Crypto	0	0	0	4	4	GCA_000442015.1	University of Michigan
1881	*Wallemia mellicola*	Wallemio	Basidio	2	0	0	2	4	GCA_004919005.1	Biotechnical Faculty, University of Ljubljana
1882	*Tremella mesenterica*	Tremello	Basidio	0	0	0	4	4	GCA_004117975.1	Broad Institute
1883	*Melampsora medusae*	Puccinio	Basidio	2	1	0	0	3	GCA_002157035.1	BC Cancer Agency, Canada's Michael Smith Genome Sciences Centre
1884	*Melampsora abietis-canadensis*	Puccinio	Basidio	3	0	0	0	3	GCA_002157025.1	BC Cancer Agency, Canada's Michael Smith Genome Sciences Centre
1885	*Schizosaccharomyces japonicus*	Schizosaccharo	Asco	0	0	0	2	2	GCA_000149845.2	Broad Institute
1886	*Schizosaccharomyces cryophilus*	Schizosaccharo	Asco	0	0	0	2	2	GCA_000004155.2	Broad Institute
1887	*Schizosaccharomyces octosporus*	Schizosaccharo	Asco	0	0	0	2	2	GCA_000150505.2	Broad Institute
1888	*Schizosaccharomyces pombe*	Schizosaccharo	Asco	0	0	0	2	2	GCA_003086255.1	Uppsala University
1889	*Herpomyces periplanetae*	Laboulbenio	Asco	2	0	0	0	2	GCA_009733715.1	University of Warsaw
1890	*Nosema bombycis*	0	Microsporidia	1	0	1	0	2	GCA_000383075.1	The Institute of Sericulture and Systems Biology (ISSB) of Southwest University (SWU)
1891	*Melampsora occidentalis*	Puccinio	Basidio	2	0	0	0	2	GCA_002157085.1	BC Cancer Agency, Canada's Michael Smith Genome Sciences Centre
1892	*Endocronartium harknessii*	Puccinio	Basidio	0	2	0	0	2	GCA_000500795.1	Tree Aggressors Identification using Genomic Approaches
1893	*Uromyces viciae-fabae*	Puccinio	Basidio	2	0	0	0	2	GCA_000785685.1	Universitaet Hohenheim
1894	*Melampsora larici-populina*	Puccinio	Basidio	1	0	0	0	1	GCA_000204055.1	US DOE Joint Genome Institute (JGI-PGF)
1895	*Cronartium comandrae*	Puccinio	Basidio	0	0	0	1	1	GCA_000464975.1	Tree Aggressors Identification using Genomic Approaches
1896	*Meira miltonrushii*	Exobasidio	Basidio	0	0	0	1	1	GCA_003144205.1	DOE Joint Genome Institute
1897	*Mitosporidium daphniae*	0	Microsporidia	0	0	0	1	1	GCA_000760515.2	Illinois Institute of Technology
1898	*Melampsora allii-populina*	Puccinio	Basidio	0	0	0	1	1	GCA_002157005.1	BC Cancer Agency, Canada's Michael Smith Genome Sciences Centre
1899	*Microbotryum lychnidis-dioicae*	Microbotryo	Basidio	0	1	0	0	1	GCA_001244265.1	INRA-LIPM
1900	*Pseudoloma neurophilia*	0	Microsporidia	1	0	0	0	1	GCA_001432165.1	University of Ottawa
1901	*Melampsora aecidioides*	Puccinio	Basidio	0	0	0	1	1	GCA_002157015.1	BC Cancer Agency, Canada's Michael Smith Genome Sciences Centre
1902	*Paramicrosporidium saccamoebae*	0	Crypto	0	0	0	0	0	GCA_002794465.1	University of Michigan
1903	*Nosema ceranae*	0	Microsporidia	0	0	0	0	0	GCA_004919615.1	Jiangxi Agricultural University

1904	*Amphiamblys sp. WSBS2006*	0	Microsporidia	0	0	0	0	0		GCA_001875675.1	A.N. Belozersky Institute of Physico-Chemical Biology, Lomonosov Moscow State University
1905	*Vavraia culicis*	0	Microsporidia	0	0	0	0	0		GCA_000192795.1	Broad Institute
1906	*Tubulinosema ratisbonensis*	0	Microsporidia	0	0	0	0	0		GCA_004000155.1	Universite Clermont Auvergne, CNRS UMR 6023
1907	*Edhazardia aedis*	0	Microsporidia	0	0	0	0	0		GCA_000230595.3	Broad Institute
1908	*Nematocida displodere*	0	Microsporidia	0	0	0	0	0		GCA_001642395.1	University of California San Diego
1909	*Hamiltosporidium tvaerminnensis*	0	Microsporidia	0	0	0	0	0		GCA_004325075.1	Illinois Institute of Technology
1910	*Trachipleistophora hominis*	0	Microsporidia	0	0	0	0	0		GCA_000316135.1	Institute for Cell and Molecular Biosciences, Newcastle University
1911	*Hamiltosporidium magnivora*	0	Microsporidia	0	0	0	0	0		GCA_004325065.1	Illinois Institute of Technology
1912	*Austropuccinia psidii*	Puccinio	Basidio	0	0	0	0	0		GCA_902702905.1	UNIVERSITY OF SYDNEY
1913	*Encephalitozoon intestinalis*	0	Microsporidia	0	0	0	0	0		GCA_000146465.1	Keeling lab, University of British Columbia
1914	*Nematocida parisii*	0	Microsporidia	0	0	0	0	0		GCA_000190615.1	Broad Institute
1915	*Enterocytozoon bieneusi*	0	Microsporidia	0	0	0	0	0		GCA_000209485.1	Tufts Cummings School of Veterinary Medicine
1916	*Vittaforma corneae*	0	Microsporidia	0	0	0	0	0		GCA_000231115.1	Broad Institute
1917	*Encephalitozoon hellem*	0	Microsporidia	0	0	0	0	0		GCA_000277815.3	University of British Columbia
1918	*Encephalitozoon romaleae*	0	Microsporidia	0	0	0	0	0		GCA_000280035.2	University of Ottawa
1919	*Nematocida sp. 1*	0	Microsporidia	0	0	0	0	0		GCA_000738915.1	Broad Institute
1920	*Ordospora colligata*	0	Microsporidia	0	0	0	0	0		GCA_000803265.1	University of British Columbia
1921	*Encephalitozoon cuniculi*	0	Microsporidia	0	0	0	0	0		GCA_001078035.1	University of Ottawa
1922	*Nematocida sp. ERTm5*	0	Microsporidia	0	0	0	0	0		GCA_001642415.1	University of California San Diego
1923	*Hemileia vastatrix*	Puccinio	Basidio	0	0	0	0	0		GCA_004125335.1	Universidade Federal de Lavras (UFLA)
1924	*Antonospora locustae*	0	Microsporidia	0	0	0	0	0		GCA_007674295.1	Zhengzhou Normal University
1925	*Cronartium quercuum*	Puccinio	Basidio	0	0	0	0	0		GCA_000500775.1	Tree Aggressors Identification using Genomic Approaches
1926	*Pneumocystis jirovecii*	Pneumocystido	Asco	0	0	0	0	0		GCA_001477535.1	Broad Institute
1927	*Hepatospora eriocheir*	0	Microsporidia	0	0	0	0	0		GCA_002087885.1	University of Exeter
1928	*Metchnikovella incurvata*	0	Microsporidia	0	0	0	0	0		GCA_003600395.1	SINGEK
1929	*Enterocytozoon hepatopenaei*	0	Microsporidia	0	0	0	0	0		GCA_003709115.1	Korea Research Institute of Bioscience & Biotechnology
1930	*Pneumocystis carinii*	Pneumocystido	Asco	0	0	0	0	0		GCA_001477545.1	Broad Institute
1931	*Meira nashicola*	Exobasidio	Basidio	0	0	0	0	0		GCA_001600355.1	RIKEN Center for Life Science Technologies, Division of Genomic Technologies
1932	*Enterospora canceri*	0	Microsporidia	0	0	0	0	0		GCA_002087915.1	University of Exeter

Table S2. List of all 1.932 fungal species/strains analyzed based on genome sequence. The species are ranked with regard to the total number of unique "Function;Family" observations, reflecting the enzyme function specificity diversity of the fungal species analyzed, specifying the number of "Function;Family" observations for each type of biomass substrate as well. The data shown is the full list of data supporting Table 2 in the main manuscript.

No	Species	Class	Phylum	Cellulose	Pectin	Xylan	Lignin	Total	ACC	Submitter
1	*Colletotrichum tropicale*	Sordario	Asco	16	53	25	18	112	GCA_013201785.1	RIKEN
2	*Colletotrichum aenigma*	Sordario	Asco	16	53	25	18	112	GCA_013390185.1	RIKEN
3	*Colletotrichum sp. COLG25*	Sordario	Asco	17	53	24	18	112	GCA_009801095.1	University of Agriculture, Faisalabad Pakistan.
4	*Colletotrichum asianum*	Sordario	Asco	16	53	24	18	111	GCA_009806415.1	Northwest A&F University
5	*Colletotrichum sp. COLG31*	Sordario	Asco	16	53	24	18	111	GCA_009800995.1	University of Agriculture, Faisalabad Pakistan.
6	*Colletotrichum siamense*	Sordario	Asco	16	53	23	18	110	GCA_013390195.1	RIKEN
7	*Colletotrichum fructicola*	Sordario	Asco	15	52	24	18	109	GCA_000319635.2	RIKEN Plant Science Center
8	*Colletotrichum viniferum*	Sordario	Asco	16	51	23	19	109	GCA_013201765.1	RIKEN
9	*Paramyrothecium roridum*	Sordario	Asco	18	49	25	17	109	GCA_003012165.1	USDA, ARS, NCAUR
10	*Colletotrichum gloeosporioides*	Sordario	Asco	15	52	24	18	109	GCA_003243855.1	Nanjing Forestry University
11	*Colletotrichum camelliae*	Sordario	Asco	16	51	23	18	108	GCA_011947485.1	Central South University of Forestry and Technology
12	*Colletotrichum karsti*	Sordario	Asco	16	49	22	18	105	GCA_011947395.1	Central South University of Forestry and Technology
13	*Stagonosporopsis tanaceti*	Dothideo	Asco	17	46	23	18	104	GCA_000812845.1	The University of Melbourne
14	*Colletotrichum musae*	Sordario	Asco	16	47	22	19	104	GCA_002814275.1	CAPES
15	*Cadophora sp. DSE1049*	Leotio	Asco	16	45	26	16	103	GCA_003073865.1	DOE Joint Genome Institute
16	*Xylaria striata*	Sordario	Asco	17	43	25	17	102	GCA_002749545.1	Zhejiang University
17	*Pestalotiopsis fici*	Sordario	Asco	15	46	23	18	102	GCA_000516985.1	Institute of microbiology, chinese academy of sciences
18	*Fusarium sp. JS1030*	Sordario	Asco	15	46	24	17	102	GCA_000966855.1	Seoul National University

19	*Fusarium oxysporum*	Sordario	Asco	15	46	23	18	102	GCA_001652425.1	CSIRO
20	*Hymenoscyphus herbarum*	Leotio	Asco	18	43	22	19	102	GCA_001414485.1	University of Exeter
21	*Clonostachys rosea*	Sordario	Asco	16	45	25	15	101	GCA_000963775.2	Chinese Academy of Agricultural Sciences
22	*Fusarium oxysporum*	Sordario	Asco	16	44	22	19	101	GCA_001703215.1	University of Amsterdam
23	*Fusarium oxysporum*	Sordario	Asco	15	45	23	18	101	GCA_012610815.1	Contact; Kurt. A. Zeller, USDA-APHIS-PPQ-S&T
24	*Fusarium oxysporum*	Sordario	Asco	16	45	23	17	101	GCA_013347345.1	Washington State University Mount Vernon Northwestern Research and Extension Center
25	*Diaporthe sp. HANT25*	Sordario	Asco	15	47	21	17	100	GCA_013435955.1	Faculty of Science, Mahidol University
26	*Phialocephala subalpina*	Leotio	Asco	16	45	24	15	100	GCA_900073065.1	Institute of Bioinformatics and Systems Biology
27	*Colletotrichum nymphaeae*	Sordario	Asco	15	47	21	17	100	GCA_001563115.1	Universidad de Salamanca
28	*Fusarium oxysporum*	Sordario	Asco	16	44	22	18	100	GCA_000260075.2	Broad Institute
29	*Colletotrichum scovillei*	Sordario	Asco	15	46	22	17	100	GCA_011075155.1	Tianjin Institute of Plant Protection
30	*Colletotrichum acutatum*	Sordario	Asco	15	46	22	17	100	GCA_001593745.1	Seoul National University
31	*Fusarium oxysporum*	Sordario	Asco	15	45	23	17	100	GCA_002234115.1	University of Amsterdam
32	*Fusarium oxysporum*	Sordario	Asco	16	45	22	17	100	GCA_009602545.1	Auburn University
33	*Fusarium oxysporum*	Sordario	Asco	15	45	23	17	100	GCA_002234045.1	University of Amsterdam
34	*Fusarium oxysporum*	Sordario	Asco	16	44	22	18	100	GCA_009746015.1	University of Massachusetts Amherts
35	*Morchella eximia*	Pezizo	Asco	17	48	18	16	99	GCA_003314645.1	Kunming University of Science and Technology
36	*Diaporthe ampelina*	Sordario	Asco	16	44	21	18	99	GCA_001630405.1	Bangalore University
37	*Diaporthe longicolla*	Sordario	Asco	16	45	20	18	99	GCA_000800745.1	Purdue University
38	*Paraphaeosphaeria sporulosa*	Dothideo	Asco	17	41	24	17	99	GCA_001642045.1	DOE Joint Genome Institute
39	*Neopestalotiopsis sp. 37M*	Sordario	Asco	15	45	21	18	99	GCA_011058875.1	University of Campinas
40	*Pestalotiopsis sp. JCM 9685*	Sordario	Asco	16	44	22	17	99	GCA_001599175.1	RIKEN Center for Life Science Technologies, Division of Genomic Technologies
41	*Fusarium oxysporum*	Sordario	Asco	15	44	24	16	99	GCA_002233775.1	University of Amsterdam
42	*Fusarium oxysporum*	Sordario	Asco	15	45	22	17	99	GCA_001702785.1	University of Amsterdam
43	*Fusarium decemcellulare*	Sordario	Asco	14	46	22	17	99	GCA_013266205.1	US Department of Agriculture, Agriculture Research Service

44	*Fusarium sp. NRRL 25184*	Sordario	Asco	16	44	22	17	99	GCA_013755755.1	US Department of Agriculture, Agriculture Research Service
45	*Fusarium oxysporum*	Sordario	Asco	15	45	22	17	99	GCA_002234135.1	University of Amsterdam
46	*Fusarium oxysporum*	Sordario	Asco	15	44	22	18	99	GCA_002233865.1	University of Amsterdam
47	*Colletotrichum sidae*	Sordario	Asco	16	45	21	17	99	GCA_004367935.1	RIKEN
48	*Fusarium oxysporum*	Sordario	Asco	15	45	23	16	99	GCA_003615085.1	NIAB-EMR
49	*Fusarium oxysporum*	Sordario	Asco	15	44	23	17	99	GCA_001702645.1	University of Amsterdam
50	*Fusarium oxysporum*	Sordario	Asco	15	45	22	17	99	GCA_002234105.1	University of Amsterdam
51	*Diaporthe capsici*	Sordario	Asco	15	45	21	17	98	GCA_013364905.1	Sichuan agricultural university
52	*Diaporthe sp. NJD1*	Sordario	Asco	15	45	21	17	98	GCA_013842865.1	Anhui Agricultural University
53	*Colletotrichum fioriniae*	Sordario	Asco	14	46	22	16	98	GCA_002930455.1	VIB
54	*Colletotrichum salicis*	Sordario	Asco	15	45	21	17	98	GCA_001563125.1	Universidad de Salamanca
55	*Colletotrichum simmondsii*	Sordario	Asco	14	46	22	16	98	GCA_001563135.1	Universidad de Salamanca
56	*Colletotrichum truncatum*	Sordario	Asco	15	45	21	17	98	GCA_014235925.1	University of Salamanca
57	*Colletotrichum musicola*	Sordario	Asco	16	45	20	17	98	GCA_014235935.1	University of Salamanca
58	*Fusarium oxysporum*	Sordario	Asco	15	44	23	16	98	GCA_002233805.1	University of Amsterdam
59	*Fusarium oxysporum*	Sordario	Asco	15	45	22	16	98	GCA_002233895.1	University of Amsterdam
60	*Fusarium udum*	Sordario	Asco	15	45	23	15	98	GCA_013186905.1	US Department of Agriculture, Agriculture Research Service
61	*Fusarium oxysporum*	Sordario	Asco	15	43	23	17	98	GCA_001702695.2	University of Amsterdam
62	*Aspergillus versicolor*	Eurotio	Asco	17	46	21	14	98	GCA_001890125.1	DOE Joint Genome Institute
63	*Fusarium oxysporum*	Sordario	Asco	15	44	22	17	98	GCA_005930515.1	RIKEN
64	*Fusarium oxysporum*	Sordario	Asco	15	44	22	17	98	GCA_000350345.1	BGI
65	*Gliomastix tumulicola*	Sordario	Asco	18	40	24	15	97	GCA_001599755.1	RIKEN Center for Life Science Technologies, Division of Genomic Technologies
66	*Lachnum nothofagi*	Leotio	Asco	16	42	21	18	97	GCA_003988875.1	Manaaki Whenua Landcare Research
67	*Diaporthe aspalathi*	Sordario	Asco	15	43	21	18	97	GCA_001447215.1	Beltsville Agricultural Research Center
68	*Pleosporales sp. UM 1110 2012*	Dothideo	Asco	16	39	25	17	97	GCA_000263175.2	UNIVERSITY OF MALAYA
69	*Fusarium commune*	Sordario	Asco	15	44	22	16	97	GCA_001599515.1	RIKEN Center for Life Science Technologies, Division of Genomic Technologies
70	*Fusarium oxysporum*	Sordario	Asco	16	42	22	17	97	GCA_013423245.1	St. Petersburg University

71	*Fusarium oxysporum*	Sordario	Asco	15	44	22	16	97	GCA_003977725.1	University of California at Davis
72	*Fusarium oxysporum*	Sordario	Asco	15	43	23	16	97	GCA_009755825.1	University of Massachusetts Amherst
73	*Colletotrichum sansevieriae*	Sordario	Asco	15	46	20	16	97	GCA_002749775.1	Kagoshima University
74	*Fusarium oxysporum*	Sordario	Asco	15	43	23	16	97	GCA_000260155.3	Broad Institute
75	*Fusarium sp. Na10*	Sordario	Asco	15	43	23	16	97	GCA_002234255.1	University of Amsterdam
76	*Aaosphaeria arxii*	Dothideo	Asco	18	41	22	16	97	GCA_010015735.1	DOE Joint Genome Institute
77	*Fusarium sterilihyphosum*	Sordario	Asco	15	43	22	17	97	GCA_013186845.1	US Department of Agriculture, Agriculture Research Service
78	*Colletotrichum orbiculare*	Sordario	Asco	15	44	21	17	97	GCA_000350065.2	RIKEN Plant Science Center
79	*Fusarium oxysporum*	Sordario	Asco	15	42	22	18	97	GCA_000260235.2	Broad Institute
80	*Colletotrichum trifolii*	Sordario	Asco	16	44	20	17	97	GCA_004367215.1	RIKEN
81	*Hymenoscyphus salicellus*	Leotio	Asco	16	42	20	19	97	GCA_001414355.1	University of Exeter
82	*Aspergillus sp. Z5*	Eurotio	Asco	16	45	22	14	97	GCA_001044295.1	Zhejiang University
83	*Memnoniella echinata*	Sordario	Asco	18	41	21	17	97	GCA_001599555.1	RIKEN Center for Life Science Technologies, Division of Genomic Technologies
84	*Fusarium oxysporum*	Sordario	Asco	15	42	21	19	97	GCA_001757345.1	ICRISAT
85	*Fusarium subglutinans*	Sordario	Asco	15	41	24	16	96	GCA_012071885.1	UNRC
86	*Colletotrichum incanum*	Sordario	Asco	16	43	21	16	96	GCA_001625285.1	Max Planck Genome Center Cologne
87	*Colletotrichum tofieldiae*	Sordario	Asco	15	44	21	16	96	GCA_001618725.1	Max Planck Genome Center Cologne
88	*Aspergillus sp. MA 6041*	Eurotio	Asco	15	44	22	15	96	GCA_003138005.1	University of Natural Resources and Life Sciences, Vienna
89	*Fusarium sp. NRRL 62957*	Sordario	Asco	16	41	21	18	96	GCA_012978535.1	US Department of Agriculture, Agriculture Research Service
90	*Fusarium phyllophilum*	Sordario	Asco	14	43	24	15	96	GCA_013396025.1	US Department of Agriculture, Agriculture Research Service
91	*Pezicula radicicola*	Leotio	Asco	14	45	20	17	96	GCA_003008705.1	The University of Texas Health Science Center at Houston
92	*Fusarium sp. NRRL 25303*	Sordario	Asco	15	42	23	16	96	GCA_013396255.1	US Department of Agriculture, Agriculture Research Service
93	*Aspergillus amoenus*	Eurotio	Asco	15	45	22	14	96	GCA_009812435.1	Universidad de Antioquia

94	*Eutypa lata*	Sordario	Asco	16	39	21	19	95	GCA_000349385.1	UC Davis
95	*Colletotrichum sp. JS-367*	Sordario	Asco	15	45	19	16	95	GCA_003122705.1	National Institute of Biological Resources
96	*Pseudogymnoascus sp. BL308*	Leotio	Asco	13	47	24	11	95	GCA_001630595.1	Ohio State University
97	*Colletotrichum plurivorum*	Sordario	Asco	15	45	18	17	95	GCA_014235945.1	University of Salamanca
98	*Aspergillus sp. MA 6037*	Eurotio	Asco	15	44	22	14	95	GCA_003138035.1	University of Natural Resources and Life Sciences, Vienna
99	*Xylaria sp. MSU_SB201401*	Sordario	Asco	17	40	22	16	95	GCA_002288965.1	University of Arkansas
100	*Fusarium avenaceum*	Sordario	Asco	16	41	21	17	95	GCA_000769295.1	Collaboration between Erik Lysøe (Department of Plant Health and Plant Protection, Bioforsk - Norwegian Institute of Agricultural and Environmental Research) and Linda Harris (Eastern Cereal & Oilseed Research Centre, Agriculture & Agri-Food Canada).
101	*Fusarium nygamai*	Sordario	Asco	15	42	22	16	95	GCA_001262555.1	University of Pretoria
102	*Bimuria novae-zelandiae*	Dothideo	Asco	16	39	24	16	95	GCA_010015655.1	DOE Joint Genome Institute
103	*Fusarium oxysporum*	Sordario	Asco	14	42	23	16	95	GCA_001703455.1	University of Amsterdam
104	*Articulospora tetracladia*	Leotio	Asco	15	43	21	16	95	GCA_003415645.1	Nakdonggang National Institute of Biological Resources
105	*Colletotrichum spinosum*	Sordario	Asco	16	42	20	17	95	GCA_004366825.1	RIKEN
106	*Fusarium mangiferae*	Sordario	Asco	14	42	24	15	95	GCA_900044065.1	Institute of Bioinformatics and Systems Biology
107	*Aspergillus sydowii*	Eurotio	Asco	16	44	21	14	95	GCA_009193685.1	Hamburg University
108	*Colletotrichum graminicola*	Sordario	Asco	17	37	23	18	95	GCA_000149035.1	Broad Institute
109	*Hymenoscyphus infarciens*	Leotio	Asco	16	40	20	19	95	GCA_001414345.1	University of Exeter
110	*Stenocarpella maydis*	Sordario	Asco	17	39	21	17	94	GCA_002270565.1	University of Arkansas
111	*Chalara longipes*	Leotio	Asco	16	39	23	16	94	GCA_009732865.1	DOE Joint Genome Institute
112	*Fusarium tupiense*	Sordario	Asco	14	42	23	15	94	GCA_013364945.1	US Department of Agriculture, Agriculture Research Service
113	*Verticillium zaregamsianum*	Sordario	Asco	17	41	20	16	94	GCA_002851755.1	Wageningen University & Research

114	*Fusarium oxysporum*	Sordario	Asco	15	42	21	16	94	GCA_001888865.1	ZhongKai University of Agriculture and Engineering
115	*Fusarium fracticaudum*	Sordario	Asco	14	42	23	15	94	GCA_003353625.1	Forestry and Agricultural Biotechnology Institute, University of Pretoria
116	*Fusarium sp. NRRL 53293*	Sordario	Asco	14	42	23	15	94	GCA_013759125.1	US Department of Agriculture, Agriculture Research Service
117	*Fusarium pseudocircinatum*	Sordario	Asco	14	41	23	16	94	GCA_013396035.1	US Department of Agriculture, Agriculture Research Service
118	*Paraphoma sp. B47-9*	Dothideo	Asco	15	43	21	15	94	GCA_001748405.1	National Institute for Agro-Environmental Sciences (NIAES)
119	*Fusarium sacchari*	Sordario	Asco	14	43	22	15	94	GCA_013759005.1	US Department of Agriculture, Agriculture Research Service
120	*Neofusicoccum kwambonambiense*	Dothideo	Asco	15	41	19	19	94	GCA_009829855.1	University of Pretoria
121	*Fusarium begoniae*	Sordario	Asco	15	40	23	16	94	GCA_013186755.1	US Department of Agriculture, Agriculture Research Service
122	*Fusarium odoratissimum*	Sordario	Asco	14	42	23	15	94	GCA_000260195.2	Broad Institute
123	*Fusarium pininemorale*	Sordario	Asco	14	41	24	15	94	GCA_002165215.1	Forestry and Agricultural Biotechnology Institute
124	*Fusarium mexicanum*	Sordario	Asco	14	42	23	15	94	GCA_013396015.1	US Department of Agriculture, Agriculture Research Service
125	*Fusarium acuminatum*	Sordario	Asco	16	40	21	17	94	GCA_013363215.1	US Department of Agriculture, Agriculture Research Service
126	*Fusarium foetens*	Sordario	Asco	14	43	21	16	94	GCA_013623845.1	US Department of Agriculture, Agriculture Research Service
127	*Fusarium secorum*	Sordario	Asco	15	42	21	16	94	GCA_013363185.1	US Department of Agriculture, Agriculture Research Service
128	*Pseudopyrenochaeta lycopersici*	Dothideo	Asco	16	41	21	16	94	GCA_003313425.1	University of Verona
129	*Hymenoscyphus fructigenus*	Leotio	Asco	16	39	21	18	94	GCA_001414455.1	University of Exeter
130	*Byssothecium circinans*	Dothideo	Asco	15	40	23	15	93	GCA_010015675.1	DOE Joint Genome Institute
131	*Pseudogymnoascus sp. VKM F-3775*	Leotio	Asco	15	42	22	14	93	GCA_000750715.1	Moscow State University
132	*Colletotrichum sojae*	Sordario	Asco	14	44	19	16	93	GCA_014235955.1	University of Salamanca

133	*Fusarium pseudoanthophilum*	Sordario	Asco	15	40	22	16	93	GCA_013395995.1	US Department of Agriculture, Agriculture Research Service
134	*Cairneyella variabilis*	Leotio	Asco	15	43	18	17	93	GCA_001625345.1	CSIRO
135	*Corynespora cassiicola*	Dothideo	Asco	15	41	20	17	93	GCA_900169545.1	UNIVERSITY OF BRISTOL
136	*Fusarium temperatum*	Sordario	Asco	14	41	23	15	93	GCA_001513835.1	Forestry and Agricultural Biotechnology Institute (FABI)
137	*Fusarium anthophilum*	Sordario	Asco	13	43	23	14	93	GCA_013364935.1	US Department of Agriculture, Agriculture Research Service
138	*Fusarium succisae*	Sordario	Asco	14	42	22	15	93	GCA_013186925.1	US Department of Agriculture, Agriculture Research Service
139	*Phoma sp. XZ068*	Dothideo	Asco	16	39	21	17	93	GCA_004835665.1	Institute Of Microbiology Chinese Academy of Sciences
140	*Fusarium agapanthi*	Sordario	Asco	14	42	21	16	93	GCA_001654545.1	US Department of Agriculture, Agriculture Research Service
141	*Fusarium ramigenum*	Sordario	Asco	14	43	21	15	93	GCA_013186855.1	US Department of Agriculture, Agriculture Research Service
142	*Neofusicoccum cordaticola*	Dothideo	Asco	15	42	18	18	93	GCA_009830905.1	University of Pretoria
143	*Neofusicoccum parvum*	Dothideo	Asco	15	42	17	19	93	GCA_000385595.1	UC Davis
144	*Fusarium mundagurra*	Sordario	Asco	14	41	23	15	93	GCA_013396205.1	US Department of Agriculture, Agriculture Research Service
145	*Neofusicoccum ribis*	Dothideo	Asco	15	41	18	19	93	GCA_009829435.1	University of Pretoria
146	*Fusarium bactridioides*	Sordario	Asco	15	38	24	16	93	GCA_013623355.1	US Department of Agriculture, Agriculture Research Service
147	*Fusarium dlaminii*	Sordario	Asco	14	41	23	15	93	GCA_013186775.1	US Department of Agriculture, Agriculture Research Service
148	*Ilyonectria destructans*	Sordario	Asco	14	42	20	17	93	GCA_001913115.1	Northeast Normal University
149	*Margaritispora aquatica*	Leotio	Asco	14	42	21	16	93	GCA_007644065.1	Nakdonggang National Institute of Biological Resources
150	*Pseudogymnoascus sp. VKM F-4519 (FW-2642)*	Leotio	Asco	13	45	23	11	92	GCA_000750935.1	Moscow State University
151	*Phialocephala scopiformis*	Leotio	Asco	15	39	22	16	92	GCA_001500285.1	DOE Joint Genome Institute

152	*Monosporascus sp. MG133*	Sordario	Asco	17	36	23	16	92	GCA_004155925.1	University of New Mexico
153	*Karstenula rhodostoma*	Dothideo	Asco	16	38	21	17	92	GCA_010093485.1	DOE Joint Genome Institute
154	*Verticillium klebahnii*	Sordario	Asco	17	38	20	17	92	GCA_002851715.1	Wageningen University & Research
155	*Dactylonectria macrodidyma*	Sordario	Asco	14	43	20	15	92	GCA_000935225.1	USDA-ARS
156	*Melanomma pulvis-pyrius*	Dothideo	Asco	15	39	21	17	92	GCA_010093585.1	DOE Joint Genome Institute
157	*Colletotrichum higginsianum*	Sordario	Asco	15	40	20	17	92	GCA_001672515.1	GATC Biotech AG
158	*Fusarium guttiforme*	Sordario	Asco	14	41	22	15	92	GCA_013186795.1	US Department of Agriculture, Agriculture Research Service
159	*Dactylonectria torresensis*	Sordario	Asco	14	41	21	16	92	GCA_011426275.1	AIT-Austrian Institute of Technology
160	*Lophiotrema nucula*	Dothideo	Asco	15	41	19	17	92	GCA_010015825.1	DOE Joint Genome Institute
161	*Trematosphaeria pertusa*	Dothideo	Asco	16	38	21	17	92	GCA_010094035.1	DOE Joint Genome Institute
162	*Fusarium citri*	Sordario	Asco	16	40	20	16	92	GCA_004367485.1	US Department of Agriculture, Agriculture Research Service
163	*Fusarium albosuccineum*	Sordario	Asco	14	41	19	18	92	GCA_012931995.1	US Department of Agriculture, Agriculture Research Service
164	*Fusarium verticillioides*	Sordario	Asco	14	39	23	16	92	GCA_003317015.2	CSIRO
165	*Fusarium proliferatum*	Sordario	Asco	14	41	22	15	92	GCA_003290285.1	University of Pisa
166	*Colletotrichum destructivum*	Sordario	Asco	15	40	21	16	92	GCA_009900065.1	Chinese Academy of Agricultural Sciences
167	*Colletotrichum orchidophilum*	Sordario	Asco	15	40	19	18	92	GCA_001831195.1	University of Western Brittany
168	*Fusarium fractiflexum*	Sordario	Asco	14	41	22	15	92	GCA_013758875.1	US Department of Agriculture, Agriculture Research Service
169	*Fusarium fujikuroi*	Sordario	Asco	14	41	22	15	92	GCA_900096635.1	Technische Universitat Munchen - WZW
170	*Fusarium tricinctum*	Sordario	Asco	14	40	22	16	92	GCA_900382705.2	INRA (Nouvelle-Aquitaine-Bordeaux)
171	*Hymenoscyphus scutula*	Leotio	Asco	15	39	20	18	92	GCA_001399465.1	University of Exeter
172	*Fusarium concolor*	Sordario	Asco	15	39	21	17	92	GCA_013184415.1	US Department of Agriculture, Agriculture Research Service

173	*Fusarium sp. NRRL 29148*	Sordario	Asco	15	39	21	17	92	GCA_013759095.1	US Department of Agriculture, Agriculture Research Service
174	*Xylaria flabelliformis*	Sordario	Asco	17	38	20	17	92	GCA_007182795.1	Vanderbilt University
175	*Fusarium phaseoli*	Sordario	Asco	15	40	20	17	92	GCA_013364925.1	US Department of Agriculture, Agriculture Research Service
176	*Calonectria leucothoes*	Sordario	Asco	15	39	22	16	92	GCA_002179835.1	USDA-ARS
177	*Fusarium sp. JS626*	Sordario	Asco	14	42	20	16	92	GCA_000966865.1	Seoul National University
178	*Pyrenochaeta sp. DS3sAY3a*	Dothideo	Asco	15	40	21	16	92	GCA_001644535.1	DOE Joint Genome Institute
179	*Ophiobolus disseminans*	Dothideo	Asco	16	37	22	17	92	GCA_010093685.1	DOE Joint Genome Institute
180	*Aspergillus steynii*	Eurotio	Asco	15	43	19	15	92	GCA_002849105.1	DOE Joint Genome Institute
181	*Hyaloscypha variabilis*	Leotio	Asco	14	40	22	15	91	GCA_002865655.1	DOE Joint Genome Institute
182	*Zopfia rhizophila*	Dothideo	Asco	15	38	22	16	91	GCA_010093925.1	DOE Joint Genome Institute
183	*Phaeoacremonium minimum*	Sordario	Asco	15	42	19	15	91	GCA_000392275.1	UC Davis
184	*Colletotrichum coccodes*	Sordario	Asco	15	41	20	15	91	GCA_002249775.1	USDA-ARS
185	*Verticillium isaacii*	Sordario	Asco	17	38	19	17	91	GCA_002851775.1	Wageningen University & Research
186	*Fusarium sp. QHM*	Sordario	Asco	14	38	24	15	91	GCA_013416755.1	Liaoning Technical University
187	*Fusarium sp. BWC*	Sordario	Asco	14	38	24	15	91	GCA_013416785.1	Liaoning Technical University
188	*Fusarium musae*	Sordario	Asco	15	38	22	16	91	GCA_013623345.1	US Department of Agriculture, Agriculture Research Service
189	*Fusarium bulbicola*	Sordario	Asco	14	40	22	15	91	GCA_013758895.1	US Department of Agriculture, Agriculture Research Service
190	*Fusarium oxysporum*	Sordario	Asco	13	41	21	16	91	GCA_014154955.1	University of Amsterdam
191	*Lachnellula suecica*	Leotio	Asco	16	38	19	18	91	GCA_007825345.1	Canadian Food Inspection Agency (CFIA)
192	*fungal sp. EF0021*	0	0	16	37	22	16	91	GCA_000292665.1	Fraunhofer
193	*Fusarium beomiforme*	Sordario	Asco	16	38	20	17	91	GCA_002980475.2	US Department of Agriculture, Agriculture Research Service
194	*Massarina eburnea*	Dothideo	Asco	16	36	23	16	91	GCA_010093635.1	DOE Joint Genome Institute

195	*Fusarium pseudonygamai*	Sordario	Asco	14	41	22	14	91	GCA_013186785.1	US Department of Agriculture, Agriculture Research Service
196	*Fusarium tjaetaba*	Sordario	Asco	14	40	22	15	91	GCA_013396195.1	US Department of Agriculture, Agriculture Research Service
197	*Fusarium globosum*	Sordario	Asco	14	39	22	16	91	GCA_013396165.1	US Department of Agriculture, Agriculture Research Service
198	*Stagonospora sp. SRC1lsM3a*	Dothideo	Asco	17	34	22	18	91	GCA_001644525.1	DOE Joint Genome Institute
199	*Aspergillus flavus*	Eurotio	Asco	16	40	18	17	91	GCA_004150275.1	The Energy and Resources Institute
200	*Monosporascus sp. mg162*	Sordario	Asco	16	39	20	16	91	GCA_004155945.1	University of New Mexico
201	*Colletotrichum lindemuthianum*	Sordario	Asco	15	43	16	17	91	GCA_001693025.2	Universidade Federal de Vicosa
202	*Fusarium coicis*	Sordario	Asco	15	39	21	16	91	GCA_013781345.1	US Department of Agriculture, Agriculture Research Service
203	*Ilyonectria mors-panacis*	Sordario	Asco	14	40	21	16	91	GCA_002991585.1	Zhejiang Sci-Tech University
204	*Alternaria alternata*	Dothideo	Asco	15	36	23	17	91	GCA_011420255.1	National Institute of Biological Resources
205	*Phoma herbarum*	Dothideo	Asco	16	39	20	16	91	GCA_001599375.1	RIKEN Center for Life Science Technologies, Division of Genomic Technologies
206	*Neofusicoccum umdonicola*	Dothideo	Asco	15	40	18	18	91	GCA_009829365.1	University of Pretoria
207	*Didymella heteroderae*	Dothideo	Asco	16	39	20	16	91	GCA_011058895.1	University of Campinas
208	*Cudoniella acicularis*	Leotio	Asco	15	40	20	16	91	GCA_013054445.1	TU Dresden - IHI Zittau
209	*Nigrograna mackinnonii*	Dothideo	Asco	16	37	23	15	91	GCA_001007845.1	Yale University
210	*Plenodomus tracheiphilus*	Dothideo	Asco	17	37	21	16	91	GCA_010093695.1	DOE Joint Genome Institute
211	*Lasiodiplodia theobromae*	Dothideo	Asco	16	39	18	18	91	GCA_012971845.1	Beltsville Agricultural Research Center
212	*Fusarium sp. NRRL 22101*	Sordario	Asco	14	39	19	18	90	GCA_013010345.1	US Department of Agriculture, Agriculture Research Service
213	*Verticillium longisporum*	Sordario	Asco	17	37	18	18	90	GCA_001268165.1	SLU
214	*Ophiognomonia clavigignenti-juglandacearum*	Sordario	Asco	14	40	19	17	90	GCA_003671545.1	Colorado State U
215	*Pseudogymnoascus sp. VKM F-4517 (FW-2822)*	Leotio	Asco	13	41	24	12	90	GCA_000750875.1	Moscow State University
216	*Pseudogymnoascus sp. 24MN13*	Leotio	Asco	12	43	24	11	90	GCA_001662595.1	US Forest Service

217	*Fusarium sp. NRRL 66182*	Sordario	Asco	12	42	20	16	90	GCA_013266265.1	US Department of Agriculture, Agriculture Research Service
218	*Monosporascus sp. CRB-9-2*	Sordario	Asco	15	38	20	17	90	GCA_004155915.1	University of New Mexico
219	*Pseudogymnoascus sp. VKM F-4518 (FW-2643)*	Leotio	Asco	15	42	20	13	90	GCA_000750925.1	Moscow State University
220	*Fusarium sp. NRRL 62610*	Sordario	Asco	15	40	18	17	90	GCA_013186425.2	US Department of Agriculture, Agriculture Research Service
221	*Rosellinia necatrix*	Sordario	Asco	17	38	20	15	90	GCA_001445595.3	NARO Institute of Fruit Tree Science
222	*Beverwykella pulmonaria*	Dothideo	Asco	16	38	19	17	90	GCA_001599595.1	RIKEN Center for Life Science Technologies, Division of Genomic Technologies
223	*Fusarium newnesense*	Sordario	Asco	14	39	22	15	90	GCA_013184375.1	US Department of Agriculture, Agriculture Research Service
224	*Setomelanomma holmii*	Dothideo	Asco	15	37	21	17	90	GCA_010015745.1	DOE Joint Genome Institute
225	*Pezicula neosporulosa*	Leotio	Asco	14	41	19	16	90	GCA_009805495.1	Chinese Academy of Forestry
226	*Colletotrichum capsici*	Sordario	Asco	15	42	18	15	90	GCA_011947275.1	KLEF Deemed to be University
227	*Colletotrichum chlorophyti*	Sordario	Asco	15	40	18	17	90	GCA_001937105.1	RIKEN
228	*Fusarium metavorans*	Sordario	Asco	15	39	19	17	90	GCA_001633045.1	USDA-ARS
229	*Helminthosporium solani*	Dothideo	Asco	16	36	21	17	90	GCA_000498615.1	University of Wisconsin-Madison
230	*Cladosporium sp. SL-16*	Dothideo	Asco	15	40	20	15	90	GCA_002921095.1	Chinese academy of agricultural sciences
231	*Clavariopsis aquatica*	Sordario	Asco	18	35	19	18	90	GCA_013620735.1	Leibniz-Institute of Freshwater Ecology and Inland Fisheries
232	*Pseudomassariella vexata*	Sordario	Asco	15	41	18	16	90	GCA_002105095.1	DOE Joint Genome Institute
233	*Coleophoma cylindrospora*	Leotio	Asco	16	39	19	16	90	GCA_003369665.1	The University of Texas Health Science Center at Houston
234	*Preussia sp. BSL10*	Dothideo	Asco	17	38	20	15	90	GCA_001553865.1	Macrogen, Korea
235	*Didymella pinodes*	Dothideo	Asco	15	40	18	17	90	GCA_004151525.1	Curtin University
236	*Alternaria burnsii*	Dothideo	Asco	15	37	22	16	90	GCA_013036055.1	Northwest A&F University
237	*Alternaria consortialis*	Dothideo	Asco	16	36	22	16	90	GCA_001950455.1	RIKEN Center for Life Science Technologies,

238	*Hymenotorrendiella dingleyae*	Leotio	Asco	16	37	19	18	90	GCA_003988905.1	Division of Genomic Technologies Manaaki Whenua Landcare Research
239	*Calonectria naviculata*	Sordario	Asco	14	39	21	15	89	GCA_003031705.1	USDA-ARS
240	*Clohesyomyces aquaticus*	Dothideo	Asco	17	34	21	17	89	GCA_002105025.1	DOE Joint Genome Institute
241	*Verticillium albo-atrum*	Sordario	Asco	17	39	18	15	89	GCA_002851705.1	Wageningen University & Research
242	*Pseudogymnoascus sp. VKM F-103*	Leotio	Asco	12	43	23	11	89	GCA_000750895.1	Moscow State University
243	*Pseudogymnoascus verrucosus*	Leotio	Asco	12	43	24	10	89	GCA_001662655.1	US Forest Service
244	*Verticillium tricorpus*	Sordario	Asco	17	37	19	16	89	GCA_002851695.1	Wageningen University & Research
245	*Periconia macrospinosa*	Dothideo	Asco	17	34	21	17	89	GCA_003073855.1	DOE Joint Genome Institute
246	*Lophiostoma macrostomum*	Dothideo	Asco	16	35	21	17	89	GCA_010405375.1	DOE Joint Genome Institute
247	*Helotiales sp. F229*	Leotio	Asco	15	37	21	16	89	GCA_002554605.1	CEPLAS/University of Cologne
248	*Fusarium denticulatum*	Sordario	Asco	14	39	20	16	89	GCA_013396175.1	US Department of Agriculture, Agriculture Research Service
249	*Fusarium tuaranense*	Sordario	Asco	14	40	18	17	89	GCA_013363205.1	US Department of Agriculture, Agriculture Research Service
250	*Monosporascus sp. CRB-8-3*	Sordario	Asco	15	38	21	15	89	GCA_004155055.1	University of New Mexico
251	*Colletotrichum sublineola*	Sordario	Asco	15	37	21	16	89	GCA_001951195.1	University of Kentucky
252	*Fusarium thapsinum*	Sordario	Asco	14	37	21	17	89	GCA_013186935.1	US Department of Agriculture, Agriculture Research Service
253	*Aspergillus fumigatiaffinis*	Eurotio	Asco	15	39	20	15	89	GCA_012656285.1	UNICAMP
254	*Verticillium nubilum*	Sordario	Asco	16	38	18	17	89	GCA_002851675.1	Wageningen University & Research
255	*Fusarium vanettenii*	Sordario	Asco	15	39	18	17	89	GCA_000151355.1	DOE Joint Genome Institute
256	*Phaeosphaeria sp. A1 3.1a*	Dothideo	Asco	16	34	23	16	89	GCA_003501895.1	Centre for Crop & Disease Management, Curtin University
257	*Phaeosphaeria sp. H6.2b*	Dothideo	Asco	16	34	23	16	89	GCA_003503105.1	Centre for Crop & Disease Management, Curtin University

258	*Fusarium anguioides*	Sordario	Asco	16	35	20	18	89	GCA_012977745.1	US Department of Agriculture, Agriculture Research Service
259	*Aspergillus sergii*	Eurotio	Asco	16	41	15	17	89	GCA_009193525.1	DOE Joint Genome Institute
260	*Fusarium circinatum*	Sordario	Asco	14	40	19	16	89	GCA_000497325.3	Pitch canker sequencing project
261	*Didymella keratinophila*	Dothideo	Asco	15	39	20	15	89	GCA_011058865.1	University of Campinas
262	*Alternaria tenuissima*	Dothideo	Asco	15	36	22	16	89	GCA_004156035.1	NIAB-East Malling Research
263	*Alternaria arborescens*	Dothideo	Asco	16	35	22	16	89	GCA_004634205.1	USFDA
264	*Xylaria multiplex*	Sordario	Asco	17	37	20	15	89	GCA_011057905.1	TU Dresden - IHI Zittau
265	*Aspergillus ustus*	Eurotio	Asco	15	39	22	13	89	GCA_000812125.1	Institute of developmentive and regenerative biology
266	*Lasiodiplodia pseudotheobromae*	Dothideo	Asco	16	38	16	19	89	GCA_009829805.1	University of Pretoria
267	*Didymella segeticola*	Dothideo	Asco	16	39	17	17	89	GCA_004522025.1	Guizhou University
268	*Pseudogymnoascus sp. 04NY16*	Leotio	Asco	12	43	22	11	88	GCA_001630575.1	Ohio State University
269	*Monosporascus sp. 5C6A*	Sordario	Asco	17	36	19	16	88	GCA_004155035.1	University of New Mexico
270	*Pseudogymnoascus sp. VKM F-4513 (FW-928)*	Leotio	Asco	12	43	22	11	88	GCA_000750755.1	Moscow State University
271	*Pseudogymnoascus sp. VKM F-4514 (FW-929)*	Leotio	Asco	13	38	24	13	88	GCA_000750795.1	Moscow State University
272	*Colletotrichum tanaceti*	Sordario	Asco	16	38	18	16	88	GCA_005350895.1	The University of Melbourne
273	*Diaporthe helianthi*	Sordario	Asco	14	39	18	17	88	GCA_001702395.2	University of Western Brittany
274	*Pseudogymnoascus pannorum*	Leotio	Asco	13	41	22	12	88	GCA_001630605.1	Ohio State University
275	*Fusarium sarcochroum*	Sordario	Asco	13	41	18	16	88	GCA_013266185.1	US Department of Agriculture, Agriculture Research Service
276	*Aspergillus calidoustus*	Eurotio	Asco	14	41	21	12	88	GCA_001511075.1	HKI JENA
277	*Xylaria longipes*	Sordario	Asco	17	33	20	18	88	GCA_003426265.1	IHI Zittau / TU Dresden
278	*[Nectria] haematococca*	Sordario	Asco	14	39	19	16	88	GCA_010015875.1	Lishui University
279	*Fusarium xylarioides*	Sordario	Asco	14	40	19	15	88	GCA_013183765.1	University of Pretoria
280	*Fusarium sp. NRRL 66088*	Sordario	Asco	14	39	18	17	88	GCA_013186415.1	US Department of Agriculture, Agriculture Research Service
281	*Cladosporium cladosporioides*	Dothideo	Asco	16	35	22	15	88	GCA_002901145.1	Yeungnam University
282	*Fusarium algeriense*	Sordario	Asco	15	36	21	16	88	GCA_002982035.1	US Department of Agriculture, Agriculture Research Service

283	*Fusarium brevicatenulatum*	Sordario	Asco	14	38	21	15	88	GCA_013363135.1	US Department of Agriculture, Agriculture Research Service
284	*Fusarium napiforme*	Sordario	Asco	14	38	21	15	88	GCA_013396005.1	US Department of Agriculture, Agriculture Research Service
285	*Xylaria sp. BCC 1067*	Sordario	Asco	17	35	20	16	88	GCA_005188305.1	King Mongkut's University of Technology Thonburi
286	*Fusarium burgessii*	Sordario	Asco	16	35	20	17	88	GCA_002980515.1	US Department of Agriculture, Agriculture Research Service
287	*Fusarium sp. NRRL 52700*	Sordario	Asco	13	39	22	14	88	GCA_013396095.1	US Department of Agriculture, Agriculture Research Service
288	*Polyplosphaeria fusca*	Dothideo	Asco	16	34	22	16	88	GCA_010093805.1	DOE Joint Genome Institute
289	*Calonectria aciculata*	Sordario	Asco	14	37	22	15	88	GCA_013406995.1	FABI
290	*Calonectria honghensis*	Sordario	Asco	14	38	21	15	88	GCA_013403855.1	FABI
291	*Calonectria fujianensis*	Sordario	Asco	14	37	21	16	88	GCA_013406965.1	FABI
292	*Fusarium sp. NRRL 47473*	Sordario	Asco	13	38	23	14	88	GCA_013759115.1	US Department of Agriculture, Agriculture Research Service
293	*Alternaria atra*	Dothideo	Asco	16	34	22	16	88	GCA_004634305.1	USFDA
294	*Fusarium gaditjirri*	Sordario	Asco	15	36	21	16	88	GCA_013266175.1	US Department of Agriculture, Agriculture Research Service
295	*Stemphylium vesicarium*	Dothideo	Asco	15	35	21	17	88	GCA_004380135.1	University of Bologna
296	*Fusarium venenatum*	Sordario	Asco	14	38	20	16	88	GCA_900007375.1	ROTHAMSTED RESEARCH
297	*Fusarium nisikadoi*	Sordario	Asco	14	37	22	15	88	GCA_013623555.1	US Department of Agriculture, Agriculture Research Service
298	*Epicoccum sorghinum*	Dothideo	Asco	17	36	20	15	88	GCA_001879705.1	University of Sao Paulo
299	*Fusarium ambrosium*	Sordario	Asco	15	37	19	17	88	GCA_003947045.1	University of California, Riverside
300	*Alternaria sp. MG1*	Dothideo	Asco	15	35	22	16	88	GCA_003574525.1	Northwestern Polytechnical University
301	*Parastagonospora nodorum*	Dothideo	Asco	16	32	23	17	88	GCA_002267045.1	North Dakota State University
302	*Rutstroemia sp. NJR-2017a BVV2*	Leotio	Asco	15	36	22	15	88	GCA_002946415.1	Brigham Young University
303	*Alternaria solani*	Dothideo	Asco	15	35	22	16	88	GCA_002837235.1	Institute Of Microbiology Chinese Academy of Sciences

304	*Calonectria pseudoreteaudii*	Sordario	Asco	14	39	20	15	88	GCA_001879505.1	Fujian Agriculture and Forestry University
305	*Fusarium incarnatum*	Sordario	Asco	15	36	21	16	88	GCA_004367075.1	US Department of Agriculture, Agriculture Research Service
306	*Byssochlamys sp. IMV 00045*	Eurotio	Asco	16	38	19	15	88	GCA_001931905.2	Jet Propulsion Laboratory, California Institute of Technology
307	*Aureobasidium pullulans*	Dothideo	Asco	16	35	20	17	88	GCA_000721785.1	DOE Joint Genome Institute
308	*Pseudogymnoascus sp. VKM F-4515 (FW-2607)*	Leotio	Asco	12	41	23	11	87	GCA_000750805.1	Moscow State University
309	*Pseudogymnoascus sp. VKM F-4520 (FW-2644)*	Leotio	Asco	12	42	22	11	87	GCA_000750995.1	Moscow State University
310	*Pseudogymnoascus sp. VKM F-4246*	Leotio	Asco	12	44	21	10	87	GCA_000750735.1	Moscow State University
311	*Colletotrichum shisoi*	Sordario	Asco	14	40	19	14	87	GCA_006783085.1	RIKEN
312	*Pseudogymnoascus sp. VKM F-3557*	Leotio	Asco	13	39	23	12	87	GCA_000750665.1	Moscow State University
313	*Pseudogymnoascus sp. BL549*	Leotio	Asco	13	41	22	11	87	GCA_001630585.1	Ohio State University
314	*Macrophomina phaseolina*	Dothideo	Asco	14	40	17	16	87	GCA_008729105.1	USDA
315	*Arthrinium malaysianum*	Sordario	Asco	16	34	21	16	87	GCA_006508115.1	Indian Institute Of Chemical biology
316	*Aspergillus udagawae*	Eurotio	Asco	15	36	21	15	87	GCA_010724255.1	Medical Mycology Research Center
317	*Fusarium flagelliforme*	Sordario	Asco	15	36	20	16	87	GCA_004367175.1	US Department of Agriculture, Agriculture Research Service
318	*Kretzschmaria deusta*	Sordario	Asco	15	38	19	15	87	GCA_002081935.3	IHI Zittau / TU Dresden
319	*Fusarium babinda*	Sordario	Asco	14	38	19	16	87	GCA_012977765.1	US Department of Agriculture, Agriculture Research Service
320	*Cylindrocarpon cylindroides*	Sordario	Asco	17	39	17	14	87	GCA_013756995.1	US Department of Agriculture, Agriculture Research Service
321	*Fusarium austroafricanum*	Sordario	Asco	15	34	22	16	87	GCA_012932025.1	US Department of Agriculture, Agriculture Research Service
322	*Laburnicola sp. R22_1*	Dothideo	Asco	15	37	21	14	87	GCA_014281115.1	Chinese Academy of Forestry
323	*Bipolaris oryzae*	Dothideo	Asco	16	34	20	17	87	GCA_000523455.1	JGI
324	*Bipolaris zeicola*	Dothideo	Asco	16	34	20	17	87	GCA_000523435.1	JGI
325	*Pleomassaria siparia*	Dothideo	Asco	15	36	20	16	87	GCA_010093715.1	DOE Joint Genome Institute
326	*Didymella lethalis*	Dothideo	Asco	15	38	18	16	87	GCA_004335245.1	Curtin University
327	*Fusarium euwallaceae*	Sordario	Asco	15	37	18	17	87	GCA_003957675.1	University of California, Riverside

328	*Nectria sp. B-13*	Sordario	Asco	16	40	17	14	87	GCA_002682825.1	Anhui University
329	*Aspergillus minisclerotigenes*	Eurotio	Asco	16	38	16	17	87	GCA_009176455.1	DOE Joint Genome Institute
330	*Fusarium sambucinum*	Sordario	Asco	14	38	19	16	87	GCA_001567575.1	Central Potato Research Institute
331	*Fusarium sp. NRRL 62944*	Sordario	Asco	13	39	19	16	87	GCA_013186395.1	US Department of Agriculture, Agriculture Research Service
332	*Fusarium sp. AF-4*	Sordario	Asco	15	38	18	16	87	GCA_003946995.1	University of California, Riverside
333	*Colletotrichum falcatum*	Sordario	Asco	16	34	20	17	87	GCA_001484525.1	ICAR-Sugarcane Breeding Institute
334	*Diplodia seriata*	Dothideo	Asco	14	39	17	17	87	GCA_001975905.1	INRA
335	*Pseudocercospora fijiensis*	Dothideo	Asco	16	37	16	18	87	GCA_011057605.1	Instituto de Investigacion Lightbourn
336	*Hymenoscyphus laetus*	Leotio	Asco	16	31	23	17	87	GCA_001414375.1	University of Exeter
337	*Curvularia kusanoi*	Dothideo	Asco	17	35	20	15	87	GCA_011058905.1	University of Campinas
338	*Coniothyrium glycines*	Dothideo	Asco	17	35	20	15	87	GCA_004523985.2	Oklahoma State University
339	*Sclerotiophoma versabilis*	Dothideo	Asco	15	37	19	16	87	GCA_012274445.1	Fujian Agriculture and Forestry University
340	*Hymenoscyphus occultus*	Leotio	Asco	15	38	16	18	87	GCA_900536445.1	UNIVERSITY OF COPENHAGEN, DEPARTMENT OF GEOSCIENCES AND NATURAL RESOURCE MANAGEMENT
341	*Hymenoscyphus koreanus*	Leotio	Asco	15	38	16	18	87	GCA_902652825.1	UNIVERSITY OF COPENHAGEN, DEPARTMENT OF GEOSCIENCES AND NATURAL RESOURCE MANAGEMENT
342	*Alternaria brassicae*	Dothideo	Asco	15	34	22	16	87	GCA_004936725.1	University of Delhi South Campus
343	*Pyrenochaeta sp. UM 256*	Dothideo	Asco	15	37	21	14	87	GCA_000359685.2	UNIVERSITY OF MALAYA
344	*Aspergillus ochraceus*	Eurotio	Asco	14	40	19	14	87	GCA_005784425.1	Shanghai Institute of Technology
345	*Cladosporium phlei*	Dothideo	Asco	16	35	19	17	87	GCA_003614995.1	National Institute of Agricultural Science
346	*Fusarium nanum*	Sordario	Asco	15	35	22	15	87	GCA_004367095.1	US Department of Agriculture, Agriculture Research Service

347	*Tubaria furfuracea*	Agarico	Basidio	15	41	21	9	86	GCA_900069095.1	Royal Botanic Gardens, Kew
348	*Oidiodendron maius*	Leotio	Asco	11	41	22	12	86	GCA_000827325.1	JGI
349	*Coniochaeta sp. 2T2.1*	Sordario	Asco	15	36	22	13	86	GCA_009194965.1	DOE Joint Genome Institute
350	*Penicillium janthinellum*	Eurotio	Asco	14	37	22	13	86	GCA_002369805.1	CSIR-NIIST
351	*Fusarium nematophilum*	Sordario	Asco	13	40	19	14	86	GCA_013623595.1	US Department of Agriculture, Agriculture Research Service
352	*Xylaria polymorpha*	Sordario	Asco	16	35	20	15	86	GCA_003426235.1	IHI Zittau / TU Dresden
353	*Pseudogymnoascus sp. VKM F-3808*	Leotio	Asco	13	38	23	12	86	GCA_000750675.1	Moscow State University
354	*Paraphaeosphaeria minitans*	Dothideo	Asco	14	35	22	15	86	GCA_009707825.1	State Key Laboratory of Agriculture Microbiology
355	*Pseudogymnoascus sp. WSF 3629*	Leotio	Asco	14	41	20	11	86	GCA_001662585.1	US Forest Service
356	*Monosporascus sp. GIB2*	Sordario	Asco	16	35	20	15	86	GCA_004155935.1	University of New Mexico
357	*Lentithecium fluviatile*	Dothideo	Asco	16	33	20	17	86	GCA_010405425.1	DOE Joint Genome Institute
358	*Monosporascus cannonballus*	Sordario	Asco	15	36	20	15	86	GCA_004155895.1	University of New Mexico
359	*Aspergillus lentulus*	Eurotio	Asco	14	38	20	14	86	GCA_010724575.1	Medical Mycology Research Center
360	*Aspergillus arachidicola*	Eurotio	Asco	16	37	16	17	86	GCA_009193545.1	DOE Joint Genome Institute
361	*Endocalyx cinctus*	0	Asco	15	33	22	16	86	GCA_001600455.1	RIKEN Center for Life Science Technologies, Division of Genomic Technologies
362	*Aspergillus sp. ATCC 12892*	Eurotio	Asco	16	38	16	16	86	GCA_002894705.1	Pacific Northwest National Lab
363	*Fusarium torulosum*	Sordario	Asco	14	37	20	15	86	GCA_013623875.1	US Department of Agriculture, Agriculture Research Service
364	*Fusarium hostae*	Sordario	Asco	13	39	20	14	86	GCA_002234235.1	University of Amsterdam
365	*Verticillium nonalfalfae*	Sordario	Asco	17	36	17	16	86	GCA_002776445.1	University of Ljubljana, Biotechnical faculty
366	*Neonectria sp. DH2*	Sordario	Asco	16	39	16	15	86	GCA_003934905.1	Sun Yat-sen University
367	*Aspergillus novoparasiticus*	Eurotio	Asco	16	38	16	16	86	GCA_009176405.1	DOE Joint Genome Institute
368	*Fusarium armeniacum*	Sordario	Asco	14	35	21	16	86	GCA_013618295.1	US Department of Agriculture, Agriculture Research Service

369	*Fusarium sp. NRRL 53497*	Sordario	Asco	14	38	18	16	86	GCA_013184445.1	US Department of Agriculture, Agriculture Research Service
370	*Clarireedia monteithiana*	Leotio	Asco	14	35	21	16	86	GCA_002242835.1	Rutgers University
371	*Aspergillus tamarii*	Eurotio	Asco	16	39	17	14	86	GCA_009193485.1	DOE Joint Genome Institute
372	*Aspergillus sojae*	Eurotio	Asco	16	39	15	16	86	GCA_009687765.1	Tokyo Institute of Technology
373	*Bipolaris victoriae*	Dothideo	Asco	16	33	20	17	86	GCA_000527765.1	JGI
374	*Fusarium sporotrichioides*	Sordario	Asco	14	37	20	15	86	GCA_003012315.1	Robert Proctor's shared submissions
375	*Fusarium transvaalense*	Sordario	Asco	16	34	19	17	86	GCA_013623685.1	US Department of Agriculture, Agriculture Research Service
376	*Fusarium sp. NRRL 62941*	Sordario	Asco	13	38	19	16	86	GCA_012978555.1	US Department of Agriculture, Agriculture Research Service
377	*Hymenoscyphus fraxineus*	Leotio	Asco	15	36	17	18	86	GCA_001414365.1	University of Exeter
378	*Rhytidhysteron rufulum*	Dothideo	Asco	15	36	19	16	86	GCA_000467735.1	Assembling the Fungal Tree of Life (AFTOL)
379	*Calonectria crousiana*	Sordario	Asco	13	38	22	13	86	GCA_013406985.1	FABI
380	*Fusarium compactum*	Sordario	Asco	15	34	20	17	86	GCA_013618385.1	US Department of Agriculture, Agriculture Research Service
381	*Fusarium sp. AF-8*	Sordario	Asco	15	36	18	17	86	GCA_003946985.1	University of California, Riverside
382	*Lasiodiplodia sp. COLG20*	Dothideo	Asco	16	38	14	18	86	GCA_009801085.1	University of Agriculture, Faisalabad Pakistan.
383	*Verticillium dahliae*	Sordario	Asco	17	36	18	15	86	GCA_004798895.1	Wageningen University and Research
384	*Fusarium camptoceras*	Sordario	Asco	14	36	21	15	86	GCA_004367475.1	US Department of Agriculture, Agriculture Research Service
385	*Fusarium culmorum*	Sordario	Asco	14	36	21	15	86	GCA_003033665.1	NIOO-KNAW
386	*Fusarium caatingaense*	Sordario	Asco	15	35	20	16	86	GCA_013624355.1	US Department of Agriculture, Agriculture Research Service
387	*Aspergillus leporis*	Eurotio	Asco	15	39	17	15	86	GCA_009176345.1	DOE Joint Genome Institute
388	*Lasiodiplodia gonubiensis*	Dothideo	Asco	15	39	15	17	86	GCA_009829795.1	University of Pretoria
389	*Fusarium coffeatum*	Sordario	Asco	15	35	21	15	86	GCA_003316985.1	CSIRO
390	*Diplodia sapinea*	Dothideo	Asco	14	41	15	16	86	GCA_000671355.1	Forestry and Agricultural Biotechnology Institute, University of Pretoria

391	*Fusarium sublunatum*	Sordario	Asco	14	36	19	17	86	GCA_013623665.1	US Department of Agriculture, Agriculture Research Service
392	*Cucurbitaria berberidis*	Dothideo	Asco	14	37	21	14	86	GCA_010015615.1	DOE Joint Genome Institute
393	*Penicillium subrubescens*	Eurotio	Asco	14	35	23	13	85	GCA_001908125.1	CBS-KNAW Fungal Biodiversity Centre Institute of the Royal Netherlands Academy of Arts and Science
394	*Pseudogymnoascus sp. 05NY08*	Leotio	Asco	12	42	21	10	85	GCA_001662605.1	US Forest Service
395	*Pseudogymnoascus sp. VKM F-4516 (FW-969)*	Leotio	Asco	13	37	23	12	85	GCA_000750815.1	Moscow State University
396	*Gamarada debralockiae*	Leotio	Asco	13	37	22	13	85	GCA_002803225.1	Macquarie University
397	*Aspergillus terreus*	Eurotio	Asco	15	34	21	15	85	GCA_009834425.1	Nanyang Technological University
398	*Hyaloscypha bicolor*	Leotio	Asco	16	35	19	15	85	GCA_002865645.1	DOE Joint Genome Institute
399	*Xylaria grammica*	Sordario	Asco	16	35	19	15	85	GCA_004353285.1	National Institute of Biological Resources
400	*Aspergillus aff. floccosus IMV 01167*	Eurotio	Asco	15	34	21	15	85	GCA_001931935.1	Jet Propulsion Laboratory, California Institute of Technology
401	*Fusarium azukicola*	Sordario	Asco	15	34	17	19	85	GCA_001680625.1	Iowa State University
402	*Aspergillus pseudotamarii*	Eurotio	Asco	16	38	16	15	85	GCA_009193445.1	DOE Joint Genome Institute
403	*Fusarium neocosmosporiellum*	Sordario	Asco	14	39	15	17	85	GCA_006518225.1	US Department of Agriculture, Agriculture Research Service
404	*Fusarium cerealis*	Sordario	Asco	14	35	21	15	85	GCA_012600195.1	University of Minnesota
405	*Verticillium alfalfae*	Sordario	Asco	16	36	17	16	85	GCA_002851655.1	Wageningen University & Research
406	*Fusarium protoensiforme*	Sordario	Asco	14	37	17	17	85	GCA_011320165.1	US Department of Agriculture, Agriculture Research Service
407	*Aspergillus latus*	Eurotio	Asco	15	39	17	14	85	GCA_013306195.1	Vanderbilt University
408	*Fusarium graminearum*	Sordario	Asco	13	37	21	14	85	GCA_900044135.1	ROTHAMSTED RESEARCH
409	*Stemphylium lycopersici*	Dothideo	Asco	15	34	20	16	85	GCA_003268315.1	Centro de Investigaciones en Fitopatologias (CIDEFI)
410	*Calonectria pseudoturangicola*	Sordario	Asco	13	38	20	14	85	GCA_013403825.1	FABI
411	*Monosporascus sp. MC13-8B*	Sordario	Asco	15	36	19	15	85	GCA_004154975.1	University of New Mexico
412	*Fusarium floridanum*	Sordario	Asco	15	35	18	17	85	GCA_003947005.1	University of California, Riverside

413	*Aspergillus parasiticus*	Eurotio	Asco	16	37	15	17	85	GCA_013146005.1	USDA
414	*Rutstroemia sp. NJR-2017a BBW*	Leotio	Asco	15	36	19	15	85	GCA_002946425.1	Brigham Young University
415	*Coleophoma crateriformis*	Leotio	Asco	16	36	16	17	85	GCA_003369635.1	The University of Texas Health Science Center at Houston
416	*Proliferodiscus dingleyae*	Leotio	Asco	14	35	22	14	85	GCA_003988795.1	Manaaki Whenua Landcare Research
417	*Aspergillus pseudoterreus*	Eurotio	Asco	15	33	21	16	85	GCA_002927005.1	Pacific Northwest National Lab
418	*Aspergillus quadrilineatus*	Eurotio	Asco	15	38	18	14	85	GCA_013305525.1	Vanderbilt University
419	*Fusarium solani*	Sordario	Asco	15	37	16	17	85	GCA_002215905.1	National Institute of Biological Resources
420	*Hymenoscyphus linearis*	Leotio	Asco	15	37	16	17	85	GCA_900536425.1	UNIVERSITY OF COPENHAGEN, DEPARTMENT OF GEOSCIENCES AND NATURAL RESOURCE MANAGEMENT
421	*Fusarium kuroshium*	Sordario	Asco	14	36	18	17	85	GCA_003698175.1	University of California, Riverside
422	*Fusarium praegraminearum*	Sordario	Asco	15	35	21	14	85	GCA_002093855.1	US Department of Agriculture, Agriculture Research Service
423	*Macroventuria anomochaeta*	Dothideo	Asco	14	38	17	16	85	GCA_010093625.1	DOE Joint Genome Institute
424	*Hymenoscyphus repandus*	Leotio	Asco	15	37	17	16	85	GCA_001414415.1	University of Exeter
425	*Fusarium poae*	Sordario	Asco	15	33	20	17	85	GCA_001675295.1	Wageningen UR
426	*Aureobasidium sp. FSWF8-4*	Dothideo	Asco	15	35	19	16	85	GCA_001914275.1	Uppsala University
427	*Talaromyces purpureogenus*	Eurotio	Asco	13	38	22	11	84	GCA_001270325.1	Mathomics
428	*Laburnicola sp. JP-R-44*	Dothideo	Asco	15	36	18	15	84	GCA_009805535.1	Chinese Academy of Forestry
429	*Exidia glandulosa*	Agarico	Basidio	16	32	20	16	84	GCA_001632375.1	DOE Joint Genome Institute
430	*Bipolaris sorokiniana*	Dothideo	Asco	15	33	20	16	84	GCA_004329375.1	ICAR-Indian Agricultural Research Institute
431	*Aspergillus oryzae*	Eurotio	Asco	16	35	17	16	84	GCA_002007945.1	Bioprocess Technology Laboratory, BIOTEC, Thailand
432	*Neoscytalidium dimidiatum*	Dothideo	Asco	14	38	15	17	84	GCA_900092665.1	University Malaya
433	*Aspergillus hancockii*	Eurotio	Asco	16	35	15	18	84	GCA_001696595.1	CSIRO
434	*Bipolaris maydis*	Dothideo	Asco	16	31	20	17	84	GCA_000338975.1	JGI
435	*Botryosphaeria kuwatsukai*	Dothideo	Asco	14	37	17	16	84	GCA_004016305.1	Northwest A&F University

436	*Fusarium sp. NRRL 6227*	Sordario	Asco	13	35	21	15	84	GCA_013623825.1	US Department of Agriculture, Agriculture Research Service
437	*Aspergillus transmontanensis*	Eurotio	Asco	16	37	15	16	84	GCA_009193505.1	DOE Joint Genome Institute
438	*Fusarium equiseti*	Sordario	Asco	14	34	21	15	84	GCA_003313175.1	Gansu Academy of Agricultural Sciences
439	*Fusarium brasilicum*	Sordario	Asco	13	36	21	14	84	GCA_013184295.1	US Department of Agriculture, Agriculture Research Service
440	*Aspergillus bombycis*	Eurotio	Asco	15	37	16	16	84	GCA_001792695.1	USDA-ARS-SRRC
441	*Fusarium pseudograminearum*	Sordario	Asco	14	35	21	14	84	GCA_000303195.2	CSIRO
442	*Didymella exigua*	Dothideo	Asco	15	38	15	16	84	GCA_010094145.1	DOE Joint Genome Institute
443	*Fusarium scirpi*	Sordario	Asco	14	35	20	15	84	GCA_004367495.1	US Department of Agriculture, Agriculture Research Service
444	*Clarireedia jacksonii*	Leotio	Asco	14	36	19	15	84	GCA_002242905.1	Rutgers University
445	*Fusarium longipes*	Sordario	Asco	15	32	20	17	84	GCA_013618495.1	US Department of Agriculture, Agriculture Research Service
446	*Parastagonospora avenae*	Dothideo	Asco	15	31	22	16	84	GCA_003503115.1	Centre for Crop & Disease Management, Curtin University
447	*Fusarium humuli*	Sordario	Asco	14	35	20	15	84	GCA_004366955.1	US Department of Agriculture, Agriculture Research Service
448	*Clarireedia sp. CPB17*	Leotio	Asco	14	34	20	16	84	GCA_002242865.1	Rutgers University
449	*Fusarium clavum*	Sordario	Asco	15	35	19	15	84	GCA_004367155.1	US Department of Agriculture, Agriculture Research Service
450	*Neonectria ditissima*	Sordario	Asco	15	39	16	14	84	GCA_001305495.1	The New Zealand Institute for Plant & Food Research Ltd
451	*Clarireedia sp. SE16F4*	Leotio	Asco	14	36	19	15	84	GCA_002242985.1	Rutgers University
452	*Aquanectria penicillioides*	Sordario	Asco	14	38	18	14	84	GCA_003415625.1	Nakdonggang National Institute of Biological Resources
453	*Neonectria galligena*	Sordario	Asco	15	40	16	13	84	GCA_013759035.1	US Department of Agriculture, Agriculture Research Service
454	*Diplodia corticola*	Dothideo	Asco	15	37	15	17	84	GCA_001883845.1	University of Aveiro

455	*Fusarium luffae*	Sordario	Asco	14	34	20	16	84	GCA_013184325.1	US Department of Agriculture, Agriculture Research Service
456	*Fusarium langsethiae*	Sordario	Asco	14	34	20	16	84	GCA_001292635.1	Bioforsk
457	*Aspergillus westerdijkiae*	Eurotio	Asco	13	39	18	14	84	GCA_001307345.1	Nanyang Technological University
458	*Alternaria brassicicola*	Dothideo	Asco	15	32	21	16	84	GCA_002796735.1	IRHS
459	*Elsinoe arachidis*	Dothideo	Asco	14	34	20	16	84	GCA_013372555.1	Shenyang Agricultural University
460	*Aureobasidium namibiae*	Dothideo	Asco	16	32	20	16	84	GCA_000721765.1	DOE Joint Genome Institute
461	*fungal sp. No.14919*	0	0	15	34	20	14	83	GCA_002003505.1	Tsukuba Biotechnology Research Center, Astellas Pharma Inc.
462	*Pseudogymnoascus sp. 23342-1-I1*	Leotio	Asco	14	38	20	11	83	GCA_001662575.1	US Forest Service
463	*Juglanconis sp. DMW523*	Sordario	Asco	14	37	16	16	83	GCA_003013055.1	USDA-ARS
464	*Arthrinium phaeospermum*	Sordario	Asco	16	32	20	15	83	GCA_006503535.1	Sichuan Agricultural University
465	*Aspergillus pseudocaelatus*	Eurotio	Asco	15	38	16	14	83	GCA_009193665.1	DOE Joint Genome Institute
466	*Colletotrichum lentis*	Sordario	Asco	14	38	16	15	83	GCA_003386485.1	University of Saskatchewan
467	*Curvularia geniculata*	Dothideo	Asco	16	32	19	16	83	GCA_002982235.1	Universidade Federal de Mato Grosso
468	*Curvularia lunata*	Dothideo	Asco	16	32	19	16	83	GCA_005212705.1	Universidade Federal de Mato Grosso
469	*Aspergillus thermomutatus*	Eurotio	Asco	15	36	18	14	83	GCA_002237265.2	University of Montreal
470	*Fusarium meridionale*	Sordario	Asco	13	36	20	14	83	GCA_009617515.1	University of Sao Paulo
471	*Aspergillus felis*	Eurotio	Asco	14	36	19	14	83	GCA_014281915.1	UNICAMP
472	*Aspergillus nomiae*	Eurotio	Asco	15	36	17	15	83	GCA_001204775.2	USDA-ARS-SRRC
473	*Fusarium austroamericanum*	Sordario	Asco	13	36	20	14	83	GCA_009617525.1	University of Sao Paulo
474	*Glarea lozoyensis*	Leotio	Asco	17	32	18	16	83	GCA_000409485.1	Institute Of Microbiology, Chinese Academy of Sciences
475	*Xylaria sp. JS573*	Sordario	Asco	15	33	20	15	83	GCA_000966885.1	Seoul National University
476	*Fusarium irregulare*	Sordario	Asco	14	33	21	15	83	GCA_004367085.1	US Department of Agriculture, Agriculture Research Service
477	*Fusarium hainanense*	Sordario	Asco	15	33	20	15	83	GCA_013618405.1	US Department of Agriculture, Agriculture Research Service
478	*Amniculicola lignicola*	Dothideo	Asco	16	31	19	17	83	GCA_010015725.1	DOE Joint Genome Institute
479	*Clarireedia homoeocarpa*	Leotio	Asco	14	35	19	15	83	GCA_001465935.1	UMass Amherst

480	*Aspergillus nidulans*	Eurotio	Asco	15	36	18	14	83	GCA_011074995.1	Barcelona Supercomputing center (BSC)
481	*Phoma sp. RAV-16-625*	Dothideo	Asco	15	35	18	15	83	GCA_004335185.1	Curtin University
482	*Fusarium brasiliense*	Sordario	Asco	14	35	16	18	83	GCA_001680685.1	Iowa State University
483	*Fusarium tanahbumbuense*	Sordario	Asco	13	35	21	14	83	GCA_012977735.1	US Department of Agriculture, Agriculture Research Service
484	*Aspergillus mulundensis*	Eurotio	Asco	14	40	17	12	83	GCA_003369625.1	The University of Texas Health Science Center at Houston
485	*Fusarium subtropicale*	Sordario	Asco	14	35	20	14	83	GCA_003670145.1	US Department of Agriculture, Agriculture Research Service
486	*Rhynchosporium secalis*	Leotio	Asco	15	30	21	17	83	GCA_900074895.1	Technische Universitat Munchen - WZW
487	*Elsinoe fawcettii*	Dothideo	Asco	14	35	18	16	83	GCA_012977835.1	University of Southern Queensland
488	*Rhynchosporium commune*	Leotio	Asco	15	30	21	17	83	GCA_900074885.1	Technische Universitat Munchen - WZW
489	*Amphirosellinia nigrospora*	Sordario	Asco	15	33	19	15	82	GCA_004123355.1	National Institute of Biological Resources
490	*Pseudohalonectria lignicola*	Sordario	Asco	14	33	22	13	82	GCA_003049395.1	Agricultural Research Service, United States Department of Agriculture
491	*Monosporascus ibericus*	Sordario	Asco	14	34	20	14	82	GCA_004154915.1	University of New Mexico
492	*Hortaea werneckii*	Dothideo	Asco	14	36	21	11	82	GCA_002127715.1	University of California, Riverside
493	*Aspergillus novofumigatus*	Eurotio	Asco	13	39	18	12	82	GCA_002847465.1	DOE Joint Genome Institute
494	*Gaeumannomyces tritici*	Sordario	Asco	17	29	19	17	82	GCA_000145635.1	Broad Institute
495	*Nakataea oryzae*	Sordario	Asco	16	27	22	17	82	GCA_003049435.1	Agricultural Research Service, United States Department of Agriculture
496	*Fusarium asiaticum*	Sordario	Asco	13	34	21	14	82	GCA_001717845.1	Government of Canada
497	*Fusarium cortaderiae*	Sordario	Asco	13	36	19	14	82	GCA_009617495.1	University of Sao Paulo
498	*Botryosphaeria dothidea*	Dothideo	Asco	14	37	15	16	82	GCA_011503125.2	Shandong Agricultural University
499	*Shiraia sp. slf14*	Dothideo	Asco	15	32	21	14	82	GCA_000498155.1	Jiangxi Normal University
500	*Ascochyta lentis*	Dothideo	Asco	15	35	16	16	82	GCA_004011705.1	Curtin University
501	*Ascochyta rabiei*	Dothideo	Asco	14	37	16	15	82	GCA_004011695.1	Curtin University

502	*Rutstroemia sp. NJR-2017a WRK4*	Leotio	Asco	14	35	19	14	82	GCA_002946385.1	Brigham Young University
503	*Ascochyta viciae-villosae*	Dothideo	Asco	15	35	16	16	82	GCA_004335205.1	Curtin University
504	*Fusarium virguliforme*	Sordario	Asco	15	33	16	18	82	GCA_013363175.1	US Department of Agriculture, Agriculture Research Service
505	*Fusarium zanthoxyli*	Sordario	Asco	13	36	17	16	82	GCA_013623745.1	US Department of Agriculture, Agriculture Research Service
506	*Dactylellina cionopaga*	Orbilio	Asco	13	36	19	14	82	GCA_012184355.1	Laboratory for Conservation and Utilization of Bio-Resources and Key Laboratory for Microbial Diversity in Southwest China, Ministry of Education
507	*Sarocladium oryzae*	Sordario	Asco	14	31	22	15	82	GCA_001972265.1	RIKEN Center for Life Science Technologies, Division of Genomic Technologies
508	*Elsinoe ampelina*	Dothideo	Asco	14	32	20	16	82	GCA_005959805.1	Northwest A&F University
509	*Didymobotryum rigidum*	Sordario	Asco	15	31	20	15	81	GCA_001600575.1	RIKEN Center for Life Science Technologies, Division of Genomic Technologies
510	*Penicillium sp. MT2 MMC-2018*	Eurotio	Asco	13	35	22	11	81	GCA_003852855.1	Florida A&M University
511	*Microdochium bolleyi*	Sordario	Asco	15	33	18	15	81	GCA_001566295.1	DOE Joint Genome Institute
512	*Lindgomyces ingoldianus*	Dothideo	Asco	15	30	21	15	81	GCA_010093535.1	DOE Joint Genome Institute
513	*Aspergillus caelatus*	Eurotio	Asco	14	37	16	14	81	GCA_009193585.1	DOE Joint Genome Institute
514	*Aspergillus alliaceus*	Eurotio	Asco	15	35	15	16	81	GCA_009176365.1	DOE Joint Genome Institute
515	*Chrysoporthe deuterocubensis*	Sordario	Asco	13	37	17	14	81	GCA_001513825.1	Forestry and Agricultural Biotechnology Institute (FABI), University of Pretoria
516	*Auricularia cornea*	Agarico	Basidio	17	25	20	19	81	GCA_008368385.1	Jilin Agricultural University
517	*Curvularia papendorfii*	Dothideo	Asco	15	31	19	16	81	GCA_000817285.1	UNIVERSITY OF MALAYA
518	*Aspergillus viridinutans*	Eurotio	Asco	13	36	18	14	81	GCA_004368095.1	University of Melbourne

519	*Chrysoporthe austroafricana*	Sordario	Asco	13	37	17	14	81	GCA_001051155.1	Forestry and Agricultural Biotechnology Institute (FABI), University of Pretoria
520	*Xylaria hypoxylon*	Sordario	Asco	17	32	18	14	81	GCA_902806585.1	CEBITEC
521	*Ophioceras dolichostomum*	Sordario	Asco	16	29	21	15	81	GCA_003049485.1	Agricultural Research Service, United States Department of Agriculture
522	*Ascochyta viciae*	Dothideo	Asco	14	35	16	16	81	GCA_004335155.1	Curtin University
523	*Ascochyta fabae*	Dothideo	Asco	14	35	16	16	81	GCA_004335285.1	Curtin University
524	*Fusarium sp. AF-6*	Sordario	Asco	15	33	15	18	81	GCA_003947015.1	University of California, Riverside
525	*Fusarium heterosporum*	Sordario	Asco	16	31	18	16	81	GCA_013396295.1	US Department of Agriculture, Agriculture Research Service
526	*Fusarium kyushuense*	Sordario	Asco	14	33	18	16	81	GCA_013184315.1	US Department of Agriculture, Agriculture Research Service
527	*Leptosphaeria maculans*	Dothideo	Asco	16	31	20	14	81	GCA_900465115.1	GSC
528	*Pseudocercospora macadamiae*	Dothideo	Asco	13	35	15	17	80	GCA_012978405.1	University of Queensland
529	*Magnaporthales sp. P1609*	Sordario	Asco	17	28	21	14	80	GCA_003709005.1	Fujian Agriculture and Forestry University
530	*Magnaporthe sp. MG07*	Sordario	Asco	16	27	21	16	80	GCA_001936455.1	Centre for Cellular and Molecular Platforms
531	*Penicillium capsulatum*	Eurotio	Asco	14	34	17	15	80	GCA_000943775.1	Shanghai Key Laboratory of Molecular Medical Mycology, Department of Dermatology, Shanghai Changzheng Hospital; Academy of Military Medical Sciences
532	*Juglanconis oblonga*	Sordario	Asco	14	35	16	15	80	GCA_003012965.1	USDA-ARS
533	*Magnaporthiopsis rhizophila*	Sordario	Asco	15	30	20	15	80	GCA_003049465.1	Agricultural Research Service, United States Department of Agriculture
534	*Magnaporthiopsis incrustans*	Sordario	Asco	14	30	20	16	80	GCA_003049425.1	Agricultural Research Service, United States Department of Agriculture
535	*Aspergillus fischeri*	Eurotio	Asco	12	37	17	14	80	GCA_008711165.1	UMass Amherst
536	*Aspergillus sp. CLMG-2019a*	Eurotio	Asco	15	34	15	16	80	GCA_013421405.1	The University of Western Australia

537	*Cryphonectria macrospora*	Sordario	Asco	13	36	18	13	80	GCA_004802535.1	USDA-ARS
538	*Cryphonectria nitschkei*	Sordario	Asco	13	36	18	13	80	GCA_006503525.1	USDA
539	*Juglanconis juglandina*	Sordario	Asco	14	35	16	15	80	GCA_003012975.1	USDA-ARS
540	*Chrysoporthe cubensis*	Sordario	Asco	13	36	17	14	80	GCA_004802525.1	USDA-ARS
541	*Annulohypoxylon stygium*	Sordario	Asco	15	29	20	16	80	GCA_003314315.1	Kunming University of Science and Technology
542	*Epicoccum nigrum*	Dothideo	Asco	16	31	18	15	80	GCA_002116315.1	The University of Auckland
543	*Hypomontagnella monticulosa*	Sordario	Asco	15	29	21	15	80	GCA_902825475.1	CEBITEC
544	*Aspergillus unguis*	Eurotio	Asco	16	34	16	14	80	GCA_003324175.1	CSIR-NIIST
545	*Sarocladium strictum*	Sordario	Asco	15	29	21	15	80	GCA_900290465.1	CEBITEC
546	*Fusarium tucumaniae*	Sordario	Asco	14	33	16	17	80	GCA_001680535.1	Iowa State University
547	*Diplodia scrobiculata*	Dothideo	Asco	14	36	13	17	80	GCA_001455585.1	Forestry & Agricultural Biotechnology Institute, University of Pretoria
548	*Spermospora avenae*	0	Asco	15	30	20	15	80	GCA_014282315.1	Agribio-Center for Agribioscience
549	*Sarocladium brachiariae*	Sordario	Asco	15	30	20	15	80	GCA_008271525.1	Environment and Plant Protection Institute
550	*Helotiales sp. DMI_Dod_QoI*	Leotio	Asco	14	31	20	15	80	GCA_009613015.1	ARS
551	*Fusarium nurragi*	Sordario	Asco	14	31	19	16	80	GCA_012977755.1	US Department of Agriculture, Agriculture Research Service
552	*Aspergillus spinulosporus*	Eurotio	Asco	14	37	17	12	80	GCA_013305485.1	Vanderbilt University
553	*Fusarium sp. KOD 1611*	Sordario	Asco	12	35	21	12	80	GCA_013624395.1	US Department of Agriculture, Agriculture Research Service
554	*Fusarium xyrophilum*	Sordario	Asco	12	35	21	12	80	GCA_008711595.1	US Department of Agriculture, Agriculture Research Service
555	*Rhynchosporium agropyri*	Leotio	Asco	14	30	20	16	80	GCA_900074905.1	Technische Universitat Munchen - WZW
556	*Cladosporium sp. TM138-S3*	Dothideo	Asco	14	35	18	13	80	GCA_011745625.1	Hellenic Centre of Marine Research (HCMR)
557	*Neobulgaria alba*	Leotio	Asco	13	38	18	11	80	GCA_003988965.1	Manaaki Whenua Landcare Research
558	*Cytospora leucostoma*	Sordario	Asco	13	36	17	14	80	GCA_003795295.1	Northwest A&F University
559	*Aureobasidium sp. P6*	Dothideo	Asco	15	32	19	14	80	GCA_003992365.1	Ocean University of China
560	*Elsinoe australis*	Dothideo	Asco	14	33	18	15	80	GCA_003013795.1	Nanjing Forestry University
561	*Gaeumannomyces sp. JS-464*	Sordario	Asco	16	27	20	16	79	GCA_002197995.1	National Institute of Biological Resources

562	*Pseudogymnoascus sp. 03VT05*	Leotio	Asco	11	38	21	9	79	GCA_001662645.1	US Forest Service
563	*Aspergillus pseudonomius*	Eurotio	Asco	14	35	16	14	79	GCA_009176395.1	DOE Joint Genome Institute
564	*Cryphonectria radicalis*	Sordario	Asco	12	37	17	13	79	GCA_003054855.1	USDA-ARS
565	*Aspergillus bertholletiae*	Eurotio	Asco	13	37	15	14	79	GCA_009193595.1	DOE Joint Genome Institute
566	*Aspergillus hiratsukae*	Eurotio	Asco	14	34	17	14	79	GCA_014281905.1	UNICAMP
567	*Curvularia sp. IFB-Z10*	Dothideo	Asco	15	27	20	17	79	GCA_002161795.1	Nanjing University
568	*Cryphonectria parasitica*	Sordario	Asco	12	37	17	13	79	GCA_011745365.1	DOE Joint Genome Institute
569	*Auricularia subglabra*	Agarico	Basidio	16	23	21	19	79	GCA_000265015.1	JGI
570	*Lizonia empirigonia*	Dothideo	Asco	15	32	16	16	79	GCA_009982855.1	DOE Joint Genome Institute
571	*Pyrenophora seminiperda*	Dothideo	Asco	15	27	21	16	79	GCA_000465215.2	Brigham Young University
572	*Ascochyta koolunga*	Dothideo	Asco	14	34	16	15	79	GCA_004151165.1	Curtin University
573	*Bipolaris cookei*	Dothideo	Asco	16	28	19	16	79	GCA_002286855.1	University of Arkansas
574	*Pyrenophora tritici-repentis*	Dothideo	Asco	15	28	20	16	79	GCA_008692205.1	Curtin University
575	*Fusarium dimerum*	Sordario	Asco	11	36	19	13	79	GCA_013623525.1	US Department of Agriculture, Agriculture Research Service
576	*Alternaria gansuensis*	Dothideo	Asco	15	30	17	17	79	GCA_009289805.1	Lanzhou University
577	*Cladosporium sphaerospermum*	Dothideo	Asco	14	34	18	13	79	GCA_000261425.2	UNIVERSITY OF MALAYA
578	*Botrytis elliptica*	Leotio	Asco	14	35	15	15	79	GCA_004786205.1	Wageningen University
579	*Aspergillus avenaceus*	Eurotio	Asco	15	33	14	17	79	GCA_009193465.1	DOE Joint Genome Institute
580	*Talaromyces cellulolyticus*	Eurotio	Asco	12	31	22	13	78	GCA_009805475.1	JNU
581	*Talaromyces amestolkiae*	Eurotio	Asco	11	33	22	12	78	GCA_001896365.1	Centro de Investigaciones Biologicas
582	*Penicillium sp. 'occitanis'*	Eurotio	Asco	12	31	22	13	78	GCA_002382835.1	Center for Genomic Regulation (CRG)
583	*Zasmidium citrigriseum*	Dothideo	Asco	11	35	17	15	78	GCA_002786025.1	Northwest A&F University
584	*Pyricularia pennisetigena*	Sordario	Asco	16	26	21	15	78	GCA_004337985.1	Academia Sinica
585	*Pyricularia sp. CBS 133598*	Sordario	Asco	15	28	21	14	78	GCA_004337975.1	Academia Sinica
586	*Falciphora oryzae*	Sordario	Asco	15	28	19	16	78	GCA_000733355.1	Institute of Biotechnology
587	*Coniochaeta prunicola*	Sordario	Asco	14	32	19	13	78	GCA_007388105.1	University of Arizona
588	*Penicillium arizonense*	Eurotio	Asco	12	34	19	13	78	GCA_001773325.1	Chalmers University of Technology
589	*Magnaporthe sp. MG05*	Sordario	Asco	15	26	21	16	78	GCA_001936065.1	Centre for Cellular and Molecular Platforms
590	*Aspergillus turcosus*	Eurotio	Asco	14	34	17	13	78	GCA_002234965.2	University of Montreal

591	*Fusarium setosum*	Sordario	Asco	13	35	16	14	78	GCA_013623625.1	US Department of Agriculture, Agriculture Research Service
592	*Exserohilum turcicum*	Dothideo	Asco	14	30	20	14	78	GCA_000359705.1	JGI
593	*Stachybotrys chartarum*	Sordario	Asco	15	30	19	14	78	GCA_000732565.1	UT Southwestern
594	*Valsa sordida*	Sordario	Asco	13	36	16	13	78	GCA_003795275.1	Northwest A&F University
595	*Leptosphaeria biglobosa*	Dothideo	Asco	14	30	20	14	78	GCA_900465125.1	GSC
596	*Auricularia heimuer*	Agarico	Basidio	17	26	19	16	78	GCA_002287115.1	Beijing Forestry University
597	*Rutstroemia sydowiana*	Leotio	Asco	13	34	16	15	78	GCA_000812895.1	USDA-ARS
598	*Fusarium cuneirostrum*	Sordario	Asco	13	33	16	16	78	GCA_001680505.1	Iowa State University
599	*Stachybotrys chlorohalonata*	Sordario	Asco	16	29	19	14	78	GCA_000732775.1	UT Southwestern
600	*Glonium stellatum*	Dothideo	Asco	14	31	17	16	78	GCA_001692915.1	1000 Fungal Genome Project
601	*Botrytis hyacinthi*	Leotio	Asco	14	34	14	16	78	GCA_004786245.1	Wageningen University
602	*Arachnopeziza araneosa*	Leotio	Asco	14	27	21	16	78	GCA_003988855.1	Manaaki Whenua Landcare Research
603	*Botrytis galanthina*	Leotio	Asco	14	34	16	14	78	GCA_004916875.1	Wageningen University
604	*Dactylellina haptotyla*	Orbilio	Asco	13	32	19	14	78	GCA_000441935.1	Lund University
605	*Aureobasidium melanogenum*	Dothideo	Asco	15	30	18	15	78	GCA_002156615.1	Ocean University of China
606	*Sphaceloma murrayae*	Dothideo	Asco	14	32	17	15	78	GCA_002895985.1	Nanjing Forestry University
607	*Talaromyces verruculosus*	Eurotio	Asco	12	32	20	13	77	GCA_001305275.1	Penicillium verruculosum
608	*Coniochaeta ligniaria*	Sordario	Asco	15	31	17	14	77	GCA_001879275.1	DOE Joint Genome Institute
609	*Talaromyces stollii*	Eurotio	Asco	11	33	21	12	77	GCA_014065225.1	Beijing University of Chemical Technology
610	*Pyricularia oryzae*	Sordario	Asco	16	26	20	15	77	GCA_004785725.1	Kansas State University
611	*Penicillium brasilianum*	Eurotio	Asco	14	32	18	13	77	GCA_001048715.1	HKI JENA
612	*Hypoxylon sp. CI-4A*	Sordario	Asco	15	28	20	14	77	GCA_002120315.1	DOE Joint Genome Institute
613	*Magnaporthe sp. MG03*	Sordario	Asco	15	27	20	15	77	GCA_001936055.1	Centre for Cellular and Molecular Platforms
614	*Annulohypoxylon truncatum*	Sordario	Asco	14	29	20	14	77	GCA_902805465.1	CEBITEC
615	*Aspergillus oerlinghausenensis*	Eurotio	Asco	12	35	18	12	77	GCA_014250555.1	Vanderbilt University
616	*Auricularia auricula-judae*	Agarico	Basidio	17	24	19	17	77	GCA_002092955.1	Jilin Agricultural University
617	*Jackrogersella multiformis*	Sordario	Asco	14	28	20	15	77	GCA_902806575.1	CEBITEC
618	*Fusarium aywerte*	Sordario	Asco	14	29	19	15	77	GCA_013186375.1	US Department of Agriculture, Agriculture Research Service
619	*Calonectria pseudonaviculata*	Sordario	Asco	13	32	18	14	77	GCA_001696505.1	USDA-ARS

620	*Botrytis tulipae*	Leotio	Asco	14	34	14	15	77	GCA_004786125.1	Wageningen University
621	*Fusarium continuum*	Sordario	Asco	13	32	16	16	77	GCA_013184455.1	US Department of Agriculture, Agriculture Research Service
622	*Botryotinia calthae*	Leotio	Asco	15	33	14	15	77	GCA_004379285.1	Wageningen University
623	*Botrytis fragariae*	Leotio	Asco	14	33	14	16	77	GCA_013461495.1	University of Maryland, College Park
624	*Lomentospora prolificans*	Sordario	Asco	16	28	20	13	77	GCA_002276285.1	Johns Hopkins University
625	*Marssonina brunnea*	Leotio	Asco	15	30	16	16	77	GCA_011750725.1	Nanjing Forestry University
626	*Hymenopellis chiangmaiae*	Agarico	Basidio	16	29	15	17	77	GCA_003314085.1	Kunming University of Science and Technology
627	*Claviceps aff. purpurea*	Sordario	Asco	10	37	20	9	76	GCA_004016085.1	Agriculture and Agri-Food Canada
628	*Scytalidium sp. 3C*	Leotio	Asco	12	32	19	13	76	GCA_000743665.3	Saint-Petersburg State University
629	*Talaromyces wortmannii*	Eurotio	Asco	11	28	21	16	76	GCA_001939245.1	Universidad Nacional Agraria La Molina,
630	*Pyricularia grisea*	Sordario	Asco	16	25	21	14	76	GCA_003933175.1	IARI
631	*Coniochaeta sp. NC1642*	Sordario	Asco	14	31	18	13	76	GCA_007388135.1	University of Arizona
632	*Coniochaeta sp. YLH0009*	Sordario	Asco	14	31	18	13	76	GCA_007388125.1	University of Arizona
633	*Coniochaeta sp. AEA 9055*	Sordario	Asco	14	31	18	13	76	GCA_007388195.1	University of Arizona
634	*Coniochaeta sp. AEA 9094*	Sordario	Asco	14	31	18	13	76	GCA_007388145.1	University of Arizona
635	*Aspergillus neoellipticus*	Eurotio	Asco	11	36	18	11	76	GCA_003116565.1	personal
636	*Valsa malicola*	Sordario	Asco	12	35	16	13	76	GCA_003795315.1	Northwest A&F University
637	*Aspergillus persii*	Eurotio	Asco	15	31	16	14	76	GCA_002215965.1	National Institute of Biological Resources
638	*Viridothelium virens*	Dothideo	Asco	14	29	18	15	76	GCA_010094025.1	DOE Joint Genome Institute
639	*Morchella septimelata*	Pezizo	Asco	15	32	15	14	76	GCA_003062385.1	Biotechnology and Nuclear Technology Research Institute
640	*Botryotinia narcissicola*	Leotio	Asco	14	32	15	15	76	GCA_004786225.1	Wageningen University
641	*Valsa mali*	Sordario	Asco	14	35	13	14	76	GCA_000818155.1	Northwest A&F University
642	*Botrytis fabae*	Leotio	Asco	14	34	13	15	76	GCA_004335055.1	Curtin University
643	*Aureobasidium subglaciale*	Dothideo	Asco	13	32	18	13	76	GCA_000721755.1	JGI
644	*Fusarium penzigii*	Sordario	Asco	12	31	19	14	76	GCA_013623535.1	US Department of Agriculture, Agriculture Research Service
645	*Myriosclerotinia duriaeana*	Leotio	Asco	16	30	14	16	76	GCA_002162615.1	University of Toronto Mississauga

646	*Botrytis paeoniae*	Leotio	Asco	14	33	14	15	76	GCA_001005785.1	Washington State University
647	*Fusarium illudens*	Sordario	Asco	13	32	14	17	76	GCA_013623515.1	US Department of Agriculture, Agriculture Research Service
648	*Clavaria fumosa*	Agarico	Basidio	7	37	19	12	75	GCA_001179745.1	Royal Botanic Gardens, Kew
649	*Penicillium sp. CF01*	Eurotio	Asco	13	35	15	12	75	GCA_005250745.1	INAIL-Research Area
650	*Talaromyces adpressus*	Eurotio	Asco	11	32	20	12	75	GCA_002775195.1	University of Naples "Federico II"
651	*Passalora fulva*	Dothideo	Asco	12	32	14	17	75	GCA_000301015.1	Wageningen UR (University & Research centre)
652	*Magnaporthe sp. MG12*	Sordario	Asco	14	27	20	14	75	GCA_001936955.1	Centre for Cellular and Molecular Platforms
653	*Talaromyces islandicus*	Eurotio	Asco	13	28	18	16	75	GCA_000985935.1	CEBITEC
654	*Coniochaeta sp. IL0111*	Sordario	Asco	14	31	17	13	75	GCA_007388115.1	University of Arizona
655	*Magnaporthiopsis poae*	Sordario	Asco	14	29	17	15	75	GCA_000193285.1	Broad Institute
656	*Hypoxylon pulicicidum*	Sordario	Asco	14	27	19	15	75	GCA_902806525.1	CEBITEC
657	*Penicillium antarcticum*	Eurotio	Asco	14	33	15	13	75	GCA_002072345.1	Chalmers University of Technology
658	*Pyrenophora teres*	Dothideo	Asco	15	25	19	16	75	GCA_008086845.1	NDSU
659	*Penicillium paxilli*	Eurotio	Asco	12	36	16	11	75	GCA_000347475.1	Massey University
660	*Zasmidium angulare*	Dothideo	Asco	12	33	14	16	75	GCA_002786045.1	Northwest A&F University
661	*Hypoxylon sp. EC38*	Sordario	Asco	14	27	19	15	75	GCA_002120335.1	DOE Joint Genome Institute
662	*Phialocephala sp. D728*	Leotio	Asco	13	30	18	14	75	GCA_003988865.1	Manaaki Whenua Landcare Research
663	*Lachnellula willkommii*	Leotio	Asco	15	34	10	16	75	GCA_007825375.1	Canadian Food Inspection Agency (CFIA)
664	*Hypoxylon sp. CO27-5*	Sordario	Asco	14	27	19	15	75	GCA_002120305.1	DOE Joint Genome Institute
665	*Calonectria henricotiae*	Sordario	Asco	14	31	16	14	75	GCA_004380885.1	USDA-ARS
666	*Pyrenophora graminea*	Dothideo	Asco	15	25	19	16	75	GCA_012365135.1	USDA-ARS
667	*Neonectria hederae*	Sordario	Asco	14	33	14	14	75	GCA_003385265.1	USDA-ARS
668	*Botrytis cinerea*	Leotio	Asco	14	33	13	15	75	GCA_000292645.1	Wageningen University
669	*Aspergillus wentii*	Eurotio	Asco	14	32	13	16	75	GCA_001890725.1	DOE Joint Genome Institute
670	*Pirottaea palmicola*	Leotio	Asco	15	28	14	18	75	GCA_003988945.1	Manaaki Whenua Landcare Research
671	*Venturia nashicola*	Dothideo	Asco	14	33	12	16	75	GCA_004522665.1	POSTECH
672	*Myriosclerotinia scirpicola*	Leotio	Asco	15	31	13	16	75	GCA_002162505.1	University of Toronto Mississauga

673	*Sclerotinia glacialis*	Leotio	Asco	15	29	15	16	75	GCA_002162575.1	University of Toronto Mississauga
674	*Lachnellula subtilissima*	Leotio	Asco	15	31	14	15	75	GCA_007821545.1	Canadian Food Inspection Agency (CFIA)
675	*Puccinia arachidis*	Puccinio	Basidio	14	30	15	15	74	GCA_001013415.1	Junagadh Agricultural University
676	*Talaromyces pinophilus*	Eurotio	Asco	11	31	20	12	74	GCA_011392495.1	Embrapa Agroenergia
677	*Cercospora nicotianae*	Dothideo	Asco	13	29	16	16	74	GCA_002994015.1	VIB
678	*Magnaporthe sp. MG08*	Sordario	Asco	14	26	20	14	74	GCA_001936555.1	Centre for Cellular and Molecular Platforms
679	*Phialemoniopsis curvata*	Sordario	Asco	14	28	18	14	74	GCA_004353045.1	UES / AFRL
680	*Aspergillus fumigatus*	Eurotio	Asco	11	35	17	11	74	GCA_005768625.2	Nanyang Technological University
681	*Hypomontagnella submonticulosa*	Sordario	Asco	14	28	18	14	74	GCA_902806495.1	CEBITEC
682	*Hypoxylon sp. E7406B*	Sordario	Asco	14	26	19	15	74	GCA_000931505.1	Yale University
683	*Graphilbum fragrans*	Sordario	Asco	15	28	17	14	74	GCA_001513895.1	University of Pretoria
684	*Aspergillus phoenicis*	Eurotio	Asco	14	34	12	14	74	GCA_003344505.1	DOE Joint Genome Institute
685	*Aspergillus tubingensis*	Eurotio	Asco	14	34	14	12	74	GCA_010614855.1	Hankyong National University
686	*Fusarium zealandicum*	Sordario	Asco	14	34	10	16	74	GCA_013266195.1	US Department of Agriculture, Agriculture Research Service
687	*Thermothelomyces thermophilus*	Sordario	Asco	16	27	18	13	74	GCA_000226095.1	DOE Joint Genome Institute
688	*Sclerotium cepivorum*	0	Asco	15	28	15	16	74	GCA_002162485.1	University of Toronto Mississauga
689	*Paradendryphiella salina*	Dothideo	Asco	15	27	17	15	74	GCA_900634815.1	DTU Bioengineering
690	*Cantharellus lutescens*	Agarico	Basidio	10	37	18	8	73	GCA_003314295.1	Kunming University of Science and Technology
691	*Golovinomyces magnicellulatus*	Leotio	Asco	9	32	20	12	73	GCA_006912115.1	The Ohio State University
692	*Uromyces transversalis*	Puccinio	Basidio	14	32	12	15	73	GCA_002994575.1	University of Georgia
693	*Chaetomium cochliodes*	Sordario	Asco	15	25	21	12	73	GCA_001752565.1	BOKU University
694	*Mycena chlorophos*	Agarico	Basidio	13	26	17	17	73	GCA_001612595.1	KEIO UNIVERSITY
695	*Cercospora berteroae*	Dothideo	Asco	12	28	16	17	73	GCA_002933655.1	VIB
696	*Penicillium sp. MA 6036*	Eurotio	Asco	12	32	16	13	73	GCA_003138045.1	University of Natural Resources and Life Sciences, Vienna
697	*Aspergillus brasiliensis*	Eurotio	Asco	14	32	14	13	73	GCA_001889945.1	DOE Joint Genome Institute
698	*Fusarium albidum*	Sordario	Asco	13	31	14	15	73	GCA_013618265.1	US Department of Agriculture, Agriculture Research Service

699	*Hysterium pulicare*	Dothideo	Asco	14	28	16	15	73	GCA_000467715.1	Assembling the Fungal Tree of Life (AFTOL)
700	*Lachnellula cervina*	Leotio	Asco	15	30	12	16	73	GCA_007825325.1	Canadian Food Inspection Agency (CFIA)
701	*Penicillium steckii*	Eurotio	Asco	12	33	16	12	73	GCA_002072375.1	Chalmers University of Technology
702	*Ciboria shiraiana*	Leotio	Asco	15	27	15	16	73	GCA_008122225.1	Southwest University
703	*Pezoloma ericae*	Leotio	Asco	13	33	15	12	73	GCA_002865625.1	DOE Joint Genome Institute
704	*Lanzia echinophila*	Leotio	Asco	14	31	13	15	73	GCA_000812885.1	USDA-ARS
705	*Sclerotinia sclerotiorum*	Leotio	Asco	15	27	15	16	73	GCA_001857865.1	Curtin University
706	*Aspergillus vadensis*	Eurotio	Asco	14	34	13	12	73	GCA_003184925.1	DOE Joint Genome Institute
707	*Botryotinia convoluta*	Leotio	Asco	14	30	14	15	73	GCA_004786275.1	Wageningen University
708	*Aspergillus uvarum*	Eurotio	Asco	12	35	13	13	73	GCA_003184745.1	DOE Joint Genome Institute
709	*Neurospora pannonica*	Sordario	Asco	16	23	20	14	73	GCA_009805235.1	University of California, Berkeley
710	*Decorospora gaudefroyi*	Dothideo	Asco	15	24	20	14	73	GCA_010015605.1	DOE Joint Genome Institute
711	*Didymosphaeria enalia*	Dothideo	Asco	14	25	18	16	73	GCA_010094045.1	DOE Joint Genome Institute
712	*Monilinia aucupariae*	Leotio	Asco	14	31	13	15	73	GCA_002162555.1	University of Toronto Mississauga
713	*Venturia pyrina*	Dothideo	Asco	14	32	12	15	73	GCA_000738655.1	La Trobe University Department of Botany
714	*Fusarium verrucosum*	Sordario	Asco	14	26	18	15	73	GCA_013623715.1	US Department of Agriculture, Agriculture Research Service
715	*Hymenopellis radicata*	Agarico	Basidio	14	27	13	19	73	GCA_003314005.1	Kunming University of Science and Technology
716	*Pseudocercospora pini-densiflorae*	Dothideo	Asco	11	32	13	16	72	GCA_000504365.2	Canada's Michael Smith Genome Sciences Centre
717	*Talaromyces rugulosus*	Eurotio	Asco	13	28	16	15	72	GCA_013368755.1	Xi'an Jiaotong University
718	*Cercospora sesami*	Dothideo	Asco	12	29	16	15	72	GCA_013365235.1	Banaras Hindu University
719	*Cercospora cf. sigesbeckiae*	Dothideo	Asco	11	30	17	14	72	GCA_005356805.1	University of Arkansas
720	*Aspergillus olivimuriae*	Eurotio	Asco	13	29	19	11	72	GCA_003719415.1	National Center For Agricultural Utilization Research
721	*Cercospora citrullina*	Dothideo	Asco	12	30	15	15	72	GCA_013365195.1	Banaras Hindu University
722	*Coniochaeta hoffmannii*	Sordario	Asco	14	28	17	13	72	GCA_002798055.1	IHI Zittau / TU Dresden
723	*Pseudogymnoascus sp. VKM F-4281 (FW-2241)*	Leotio	Asco	12	30	20	10	72	GCA_000750745.1	Moscow State University

724	*Chaetomium globosum*	Sordario	Asco	14	27	21	10	72	GCA_000143365.1	The Genome Sequencing Platform, The Genome Assembly Team
725	*Neonectria punicea*	Sordario	Asco	15	31	12	14	72	GCA_003385315.1	USDA-ARS
726	*Penicillium fuscoglaucum*	Eurotio	Asco	12	31	16	13	72	GCA_000576735.1	INRA-LIPM
727	*Penicillium sp. SPG-F1*	Eurotio	Asco	14	28	16	14	72	GCA_003800495.1	Texas A&M University Corpus Christi
728	*Massariosphaeria phaeospora*	Dothideo	Asco	15	24	18	15	72	GCA_011032825.1	DOE Joint Genome Institute
729	*Penicillium citrinum*	Eurotio	Asco	13	31	15	13	72	GCA_001950535.1	RIKEN Center for Life Science Technologies, Division of Genomic Technologies
730	*Pseudovirgaria hyperparasitica*	Dothideo	Asco	13	25	18	16	72	GCA_010093815.1	DOE Joint Genome Institute
731	*Aspergillus homomorphus*	Eurotio	Asco	14	33	11	14	72	GCA_003184865.1	DOE Joint Genome Institute
732	*Aspergillus costaricensis*	Eurotio	Asco	14	33	13	12	72	GCA_003184835.1	DOE Joint Genome Institute
733	*Aspergillus indologenus*	Eurotio	Asco	13	34	12	13	72	GCA_003184685.1	DOE Joint Genome Institute
734	*Hawksworthiomyces lignivorus*	Sordario	Asco	14	26	19	13	72	GCA_002917075.1	University of Pretoria
735	*Clathrospora elynae*	Dothideo	Asco	15	25	17	15	72	GCA_010015635.1	DOE Joint Genome Institute
736	*Aspergillus incahuasiensis*	Eurotio	Asco	12	32	17	11	72	GCA_003719405.1	National Center For Agricultural Utilization Research
737	*Aspergillus luchuensis*	Eurotio	Asco	14	33	12	13	72	GCA_001890685.1	DOE Joint Genome Institute
738	*Aspergillus awamori*	Eurotio	Asco	14	33	12	13	72	GCA_001599415.1	RIKEN Center for Life Science Technologies, Division of Genomic Technologies
739	*Aspergillus aculeatinus*	Eurotio	Asco	13	34	12	13	72	GCA_003184765.1	DOE Joint Genome Institute
740	*Aspergillus brunneoviolaceus*	Eurotio	Asco	13	34	12	13	72	GCA_003184695.1	DOE Joint Genome Institute
741	*Botrytis porri*	Leotio	Asco	14	31	12	15	72	GCA_004786265.1	Wageningen University
742	*Aspergillus fijiensis*	Eurotio	Asco	13	34	12	13	72	GCA_003184825.1	DOE Joint Genome Institute
743	*Neurospora sp. FGSC 26635*	Sordario	Asco	14	23	19	16	72	GCA_009804975.1	University of California, Berkeley
744	*Neurospora sp. LNF1-2*	Sordario	Asco	14	23	19	16	72	GCA_009805125.1	University of California, Berkeley

745	*Neurospora sp. FGSC 26631*	Sordario	Asco	14	23	19	16	72	GCA_009805105.1	University of California, Berkeley
746	*Neurospora sp. FGSC 26629*	Sordario	Asco	14	23	19	16	72	GCA_009805155.1	University of California, Berkeley
747	*Neurospora sp. FGSC 26633*	Sordario	Asco	14	23	19	16	72	GCA_009804945.1	University of California, Berkeley
748	*Neurospora sp. LNF1-1*	Sordario	Asco	14	23	19	16	72	GCA_009805225.1	University of California, Berkeley
749	*Neurospora sp. FGSC 26630*	Sordario	Asco	14	23	19	16	72	GCA_009805085.1	University of California, Berkeley
750	*Neurospora sp. FGSC 26627*	Sordario	Asco	14	23	19	16	72	GCA_009805365.1	University of California, Berkeley
751	*Venturia aucupariae*	Dothideo	Asco	14	32	12	14	72	GCA_003693225.1	INRA
752	*Morchella importuna*	Pezizo	Asco	15	30	14	13	72	GCA_003444635.1	Huazhong Agricultural University
753	*Morchella sextelata*	Pezizo	Asco	16	30	13	13	72	GCA_009741755.1	Inner Mongolia International Mongolian Hospital
754	*Phyllosticta capitalensis*	Dothideo	Asco	13	32	10	17	72	GCA_001604925.1	Citrus Research and Education Center, University of Florida
755	*Cercospora brassicicola*	Dothideo	Asco	11	29	16	15	71	GCA_013365245.1	Banaras Hindu University
756	*Raffaelea albimanens*	Sordario	Asco	16	24	18	13	71	GCA_002778245.1	University of Montana
757	*Daldinia childiae*	Sordario	Asco	15	25	17	14	71	GCA_008694065.1	National Institute of Biological Resources
758	*Aspergillus sclerotiorum*	Eurotio	Asco	15	27	15	14	71	GCA_000530345.1	SVSB
759	*Penicillium sp. str. #12*	Eurotio	Asco	14	27	16	14	71	GCA_013138035.1	None
760	*Penicillium sp. BW_12*	Eurotio	Asco	14	27	16	14	71	GCA_008931925.1	Tufts University
761	*Penicillium sp. SPG-F15*	Eurotio	Asco	12	30	15	14	71	GCA_003800485.1	Texas A&M University - Corpus Christi
762	*Entonaema liquescens*	Sordario	Asco	15	25	17	14	71	GCA_902805475.1	CEBITEC
763	*Pyrenopolyporus hunteri*	Sordario	Asco	15	24	17	15	71	GCA_902806595.1	CEBITEC
764	*Lachnellula hyalina*	Leotio	Asco	15	30	10	16	71	GCA_007821495.1	Canadian Food Inspection Agency (CFIA)
765	*Aspergillus welwitschiae*	Eurotio	Asco	14	33	12	12	71	GCA_009761105.1	Universidade Federal de Minas Gerais
766	*Hypoxylon rickii*	Sordario	Asco	15	23	19	14	71	GCA_902806535.1	CEBITEC
767	*Aspergillus niger*	Eurotio	Asco	14	32	12	13	71	GCA_011316255.1	South China Agricultural University
768	*Aspergillus piperis*	Eurotio	Asco	14	33	11	13	71	GCA_003184755.1	DOE Joint Genome Institute
769	*Aspergillus japonicus*	Eurotio	Asco	13	34	11	13	71	GCA_003184785.1	DOE Joint Genome Institute

770	*Aspergillus sclerotiicarbonarius*	Eurotio	Asco	14	31	11	15	71	GCA_003184635.1	DOE Joint Genome Institute
771	*Thelonectria rubi*	Sordario	Asco	12	29	16	14	71	GCA_013420875.1	USDA-ARS
772	*Schizophyllum commune*	Agarico	Basidio	16	25	14	16	71	GCA_001599475.1	RIKEN Center for Life Science Technologies, Division of Genomic Technologies
773	*Neurospora sp. FGSC 26632*	Sordario	Asco	14	23	19	15	71	GCA_009804965.1	University of California, Berkeley
774	*Monilinia laxa*	Leotio	Asco	13	30	14	14	71	GCA_009299455.1	UniBa - Univpm
775	*Venturia oleaginea*	Dothideo	Asco	14	31	11	15	71	GCA_013176395.1	FUJIAN AGRICULTURE AND FORESTRY UNIVERSITY
776	*Morchella conica*	Pezizo	Asco	15	30	13	13	71	GCA_003790465.1	DOE Joint Genome Institute
777	*Zasmidium cellare*	Dothideo	Asco	12	29	13	16	70	GCA_010093935.1	DOE Joint Genome Institute
778	*Cercospora kikuchii*	Dothideo	Asco	11	29	16	14	70	GCA_009193115.1	School of Agriculture of the University of Buenos Aires
779	*Pseudocercospora cruenta*	Dothideo	Asco	12	29	13	16	70	GCA_013365205.1	Banaras Hindu University
780	*Penicillium flavigenum*	Eurotio	Asco	13	29	15	13	70	GCA_002072365.1	Chalmers University of Technology
781	*Cercospora beticola*	Dothideo	Asco	11	28	16	15	70	GCA_003370525.1	University of Southern Queensland
782	*Cercospora canescens*	Dothideo	Asco	12	28	15	15	70	GCA_000347735.1	Banaras Hindu University
783	*Talaromyces funiculosus*	Eurotio	Asco	12	26	19	13	70	GCA_004299765.1	Shandong Agricultural University
784	*Penicillium sclerotiorum*	Eurotio	Asco	12	32	15	11	70	GCA_001750025.1	Rutgers, The State University of New Jersey
785	*Pseudocercospora eumusae*	Dothideo	Asco	11	32	14	13	70	GCA_001578235.1	University of California Davis
786	*Daldinia sp. EC12*	Sordario	Asco	15	24	18	13	70	GCA_002120325.1	DOE Joint Genome Institute
787	*Talaromyces stipitatus*	Eurotio	Asco	12	25	18	15	70	GCA_000003125.1	J. Craig Venter Institute
788	*Trichothecium sympodiale*	Sordario	Asco	16	20	21	13	70	GCA_003012115.1	USDA, ARS, NCAUR
789	*Daldinia concentrica*	Sordario	Asco	15	24	17	14	70	GCA_902805455.1	CEBITEC
790	*Neonectria coccinea*	Sordario	Asco	14	32	11	13	70	GCA_013757005.1	US Department of Agriculture, Agriculture Research Service
791	*Delitschia confertaspora*	Dothideo	Asco	15	25	18	12	70	GCA_010093945.1	DOE Joint Genome Institute
792	*Ampelomyces quisqualis*	Dothideo	Asco	14	27	16	13	70	GCA_010094095.1	DOE Joint Genome Institute

793	*Penicillium expansum*	Eurotio	Asco	12	29	15	14	70	GCA_004302965.1	ARS
794	*Corinectria fuckeliana*	Sordario	Asco	13	32	13	12	70	GCA_003385255.1	USDA-ARS
795	*Aspergillus floridensis*	Eurotio	Asco	13	33	12	12	70	GCA_012184565.1	Belgian Coordinated Collection of Microorganisms, BCCM
796	*Aspergillus violaceofuscus*	Eurotio	Asco	13	33	11	13	70	GCA_003184705.1	DOE Joint Genome Institute
797	*Aspergillus carbonarius*	Eurotio	Asco	13	32	10	15	70	GCA_001990825.1	DOE Joint Genome Institute
798	*Limonomyces culmigenus*	Agarico	Basidio	12	21	22	15	70	GCA_002233555.1	USDA-ARS
799	*Neurospora africana*	Sordario	Asco	14	21	20	15	70	GCA_000604205.2	EBC
800	*Ascocoryne sarcoides*	Leotio	Asco	13	32	14	11	70	GCA_000328965.1	Yale University
801	*Neurospora sp. FGSC 26636*	Sordario	Asco	14	22	19	15	70	GCA_009804955.1	University of California, Berkeley
802	*Neurospora sp. FGSC 26626*	Sordario	Asco	15	22	18	15	70	GCA_009805385.1	University of California, Berkeley
803	*Monilinia fructicola*	Leotio	Asco	14	28	13	15	70	GCA_008692225.1	UniBa - Univpm
804	*Myriosclerotinia curreyana*	Leotio	Asco	14	27	14	15	70	GCA_002162495.1	University of Toronto Mississauga
805	*Podospora anserina*	Sordario	Asco	16	19	19	16	70	GCA_005222925.1	Lomonosov Moscow State University
806	*Thermothielavioides terrestris*	Sordario	Asco	15	24	17	14	70	GCA_900343105.1	CHALMERS UNIVERSITY OF TECHNOLOGY
807	*Sporothrix globosa*	Sordario	Asco	14	25	18	13	70	GCA_001630435.1	University of Messina
808	*Pseudocercospora fuligena*	Dothideo	Asco	11	29	13	16	69	GCA_014298035.1	University of California
809	*Cercospora cf. flagellaris*	Dothideo	Asco	11	28	16	14	69	GCA_005356885.1	University of Arkansas
810	*Penicillium verrucosum*	Eurotio	Asco	14	28	14	13	69	GCA_000970515.2	Max Rubner-Institut
811	*Cercospora sojina*	Dothideo	Asco	12	27	15	15	69	GCA_002534735.1	Chinese Academy of Sciences
812	*Coniella lustricola*	Sordario	Asco	12	33	14	10	69	GCA_003019895.1	DOE Joint Genome Institute
813	*Penicillium nordicum*	Eurotio	Asco	14	28	14	13	69	GCA_001278595.1	Agriculture and Agri-Food Canada
814	*Penicillium rolfsii*	Eurotio	Asco	12	31	16	10	69	GCA_011392555.1	Embrapa Agroenergia
815	*Penicillium sp. BW_162_3FA*	Eurotio	Asco	12	30	14	13	69	GCA_008931945.1	Tufts University
816	*Penicillium biforme*	Eurotio	Asco	12	30	14	13	69	GCA_000577785.1	INRA-LIPM
817	*Trichothecium roseum*	Sordario	Asco	14	28	17	10	69	GCA_003012185.1	USDA, ARS, NCAUR
818	*Lachnellula arida*	Leotio	Asco	14	29	11	15	69	GCA_007821475.1	Canadian Food Inspection Agency (CFIA)
819	*Lachnellula occidentalis*	Leotio	Asco	14	27	13	15	69	GCA_007821535.1	Canadian Food Inspection Agency (CFIA)
820	*Daldinia eschscholtzii*	Sordario	Asco	15	24	17	13	69	GCA_001951055.1	nanjing university

821	*Venustampulla echinocandica*	Leotio	Asco	13	27	17	12	69	GCA_003357145.1	The University of Texas Health Science Center at Houston
822	*Microthyrium microscopicum*	Dothideo	Asco	11	27	16	15	69	GCA_010405405.1	DOE Joint Genome Institute
823	*Cercospora zeae-maydis*	Dothideo	Asco	13	25	15	16	69	GCA_010093985.1	JGI
824	*Aspergillus neoniger*	Eurotio	Asco	14	31	12	12	69	GCA_003184625.1	DOE Joint Genome Institute
825	*Sporothrix pallida*	Sordario	Asco	14	25	18	12	69	GCA_000710705.2	University of Messina
826	*Aspergillus eucalypticola*	Eurotio	Asco	14	32	11	12	69	GCA_003184535.1	DOE Joint Genome Institute
827	*Neurospora sp. FGSC 26634*	Sordario	Asco	14	22	18	15	69	GCA_009804985.1	University of California, Berkeley
828	*Neurospora sp. FGSC 26628*	Sordario	Asco	14	22	18	15	69	GCA_009805345.1	University of California, Berkeley
829	*Aspergillus sclerotioniger*	Eurotio	Asco	14	30	10	15	69	GCA_003184525.1	DOE Joint Genome Institute
830	*Ciborinia camelliae*	Leotio	Asco	13	29	13	14	69	GCA_001247705.1	Massey University
831	*Neurospora sp. FGSC 26637*	Sordario	Asco	14	21	19	15	69	GCA_009804845.1	University of California, Berkeley
832	*Monilinia polystroma*	Leotio	Asco	13	30	12	14	69	GCA_002909645.1	United States Department of Agriculture
833	*Podospora comata*	Sordario	Asco	16	19	18	16	69	GCA_900290415.1	IED
834	*Westerdykella ornata*	Dothideo	Asco	15	21	18	15	69	GCA_010094085.1	DOE Joint Genome Institute
835	*Neurospora sp. FGSC 26624*	Sordario	Asco	14	22	18	15	69	GCA_009805485.1	University of California, Berkeley
836	*Monilinia fructigena*	Leotio	Asco	13	30	11	15	69	GCA_003260565.1	Rita Milvia De Miccolis Angelini's shared submissions
837	*Penicillium solitum*	Eurotio	Asco	13	28	14	13	68	GCA_000952775.2	JCVI
838	*Penicillium polonicum*	Eurotio	Asco	13	28	14	13	68	GCA_013466175.1	National Institute of Agricultural Sciences, RDA
839	*Penicillium sp. BW_MB*	Eurotio	Asco	13	27	14	14	68	GCA_008931935.1	Tufts University
840	*Penicillium freii*	Eurotio	Asco	13	28	15	12	68	GCA_001513925.1	Agriculture and Agri-Food Canada
841	*Coniella vitis*	Sordario	Asco	11	31	14	12	68	GCA_011317545.1	qingdao agricultural university
842	*Penicillium camemberti*	Eurotio	Asco	12	29	14	13	68	GCA_000513335.1	INRA-LIPM
843	*Raffaelea ambrosiae*	Sordario	Asco	16	22	16	14	68	GCA_002778195.1	University of Montana
844	*Neurospora sp. FGSC 26638*	Sordario	Asco	13	22	19	14	68	GCA_009804825.1	University of California, Berkeley

845	*Neurospora sp. FGSC 6877*	Sordario	Asco	13	24	18	13	68	GCA_009805295.1	University of California, Berkeley
846	*Peniophora sp. CBMAI 1063*	Agarico	Basidio	14	22	17	15	68	GCA_900536885.1	LABORATORIO NACIONAL DE CIENCIA E TECNOLOGIA DO BI
847	*Penicillium oxalicum*	Eurotio	Asco	12	29	17	10	68	GCA_005546515.1	Nanyang Technological University
848	*Sclerotinia borealis*	Leotio	Asco	13	30	11	14	68	GCA_000503235.1	Centre Bioengineering RAS
849	*Neurospora cerealis*	Sordario	Asco	15	21	16	16	68	GCA_009806135.1	University of California, Berkeley
850	*Venturia asperata*	Dothideo	Asco	14	29	11	14	68	GCA_003689065.1	INRA
851	*Neurospora sp. FGSC 26623*	Sordario	Asco	14	22	18	14	68	GCA_009805935.1	University of California, Berkeley
852	*Aspergillus ibericus*	Eurotio	Asco	13	30	10	15	68	GCA_003184845.1	DOE Joint Genome Institute
853	*Punctularia strigosozonata*	Agarico	Basidio	13	25	14	16	68	GCA_000264995.1	JGI
854	*Madurella mycetomatis*	Sordario	Asco	15	21	20	12	68	GCA_001275765.2	ErasmusMC
855	*Neurospora sublineolata*	Sordario	Asco	14	22	18	14	68	GCA_000604185.2	EBC
856	*Fusarium domesticum*	Sordario	Asco	12	33	11	12	68	GCA_013618395.1	US Department of Agriculture, Agriculture Research Service
857	*Morchella crassipes*	Pezizo	Asco	16	28	12	12	68	GCA_009192285.1	Huazhong Agricultural University
858	*Phyllosticta citriasiana*	Dothideo	Asco	13	28	10	17	68	GCA_009193405.1	Zhejiang University
859	*Pallidocercospora crystallina*	Dothideo	Asco	11	30	12	14	67	GCA_003666085.1	Xijing Hospital, Fourth Military Medical University
860	*Pseudocercospora musae*	Dothideo	Asco	11	30	13	13	67	GCA_001578225.1	University of California Davis
861	*Ramularia coccinea*	Dothideo	Asco	16	19	21	11	67	GCA_013461505.1	Yancheng Institute of Technology
862	*Acidomyces sp. 'richmondensis'*	Dothideo	Asco	13	26	16	12	67	GCA_003545705.1	University of Massachusetts
863	*Aspergillus ellipticus*	Eurotio	Asco	13	33	9	12	67	GCA_003184645.1	DOE Joint Genome Institute
864	*Venturia inaequalis*	Dothideo	Asco	13	29	10	15	67	GCA_003689225.1	INRA
865	*Mycosphaerelloides madeirae*	Dothideo	Asco	13	27	12	15	67	GCA_002785995.1	Northwest A&F University
866	*Neurospora sp. FGSC 26625*	Sordario	Asco	13	22	18	14	67	GCA_009805435.1	University of California, Berkeley
867	*Neurospora sp. CHS-2018a*	Sordario	Asco	14	20	18	15	67	GCA_009802375.1	University of California, Berkeley
868	*Psilocybe cf. subviscida*	Agarico	Basidio	12	22	20	13	67	GCA_013368295.1	Lund University

869	*Cylindrobasidium torrendii*	Agarico	Basidio	14	27	15	11	67	GCA_000934385.1	DOE Joint Genome Institute
870	*Diplocarpon rosae*	Leotio	Asco	12	29	12	14	67	GCA_002317995.1	Leibniz Universitaet Hannover
871	*Phyllosticta citricarpa*	Dothideo	Asco	12	29	10	16	67	GCA_000382785.1	Zhejiang university
872	*Basipetospora chlamydospora*	Eurotio	Asco	16	15	21	15	67	GCA_001599675.1	RIKEN Center for Life Science Technologies, Division of Genomic Technologies
873	*Mycena citricolor*	Agarico	Basidio	12	24	12	18	66	GCA_003987915.1	Universidade de Sao Paulo
874	*Penicillium sp. MA 6040*	Eurotio	Asco	12	28	15	11	66	GCA_003138025.1	University of Natural Resources and Life Sciences, Vienna
875	*Penicillium rubens*	Eurotio	Asco	12	28	15	11	66	GCA_902636305.1	IMPERIAL COLLEGE LONDON
876	*Penicillium vulpinum*	Eurotio	Asco	13	24	15	14	66	GCA_002072255.1	Chalmers University of Technology
877	*Penicillium chrysogenum*	Eurotio	Asco	12	28	15	11	66	GCA_000710275.1	Ruhr-Univeristaet Bochum
878	*Penicillium sp. HKF2*	Eurotio	Asco	12	28	15	11	66	GCA_002000375.1	CSIR-NEERI
879	*Crepidotus sp. BD-2015*	Agarico	Basidio	11	19	19	17	66	GCA_001179765.1	Royal Botanic Gardens, Kew
880	*Cyanodermella asteris*	Lecanoro	Asco	14	25	14	13	66	GCA_900618795.1	CEBITEC
881	*Trichothecium ovalisporum*	Sordario	Asco	15	18	21	12	66	GCA_003012195.1	USDA, ARS, NCAUR
882	*Dothistroma pini*	Dothideo	Asco	12	25	14	15	66	GCA_002116355.1	Canada's Michael Smith Genome Sciences Centre
883	*Dothidotthia symphoricarpi*	Dothideo	Asco	12	30	11	13	66	GCA_010015815.1	DOE Joint Genome Institute
884	*Venturia carpophila*	Dothideo	Asco	14	28	10	14	66	GCA_001990985.1	USDA-ARS
885	*Scedosporium boydii*	Sordario	Asco	14	21	17	14	66	GCA_002221725.1	GEIHP, UPRES EA 3142
886	*Sporothrix schenckii*	Sordario	Asco	14	23	17	12	66	GCA_000961545.1	LNCC
887	*Sporothrix brasiliensis*	Sordario	Asco	14	22	18	12	66	GCA_000820605.1	LNCC
888	*Penicillium sp. CF05*	Eurotio	Asco	13	27	14	11	65	GCA_002916455.1	INAIL-Research Area
889	*Penicillium nalgiovense*	Eurotio	Asco	13	27	14	11	65	GCA_000577395.2	INRA-LIPM
890	*Arthrobotrys entomopaga*	Orbilio	Asco	12	23	18	12	65	GCA_012184315.1	Laboratory for Conservation and Utilization of Bio-Resources and Key Laboratory for Microbial Diversity in Southwest China, Ministry of Education
891	*Hypoxylon fragiforme*	Sordario	Asco	13	22	16	14	65	GCA_902806515.1	CEBITEC

892	*Neurospora sp. CHS-2018c*	Sordario	Asco	13	21	17	14	65	GCA_009806235.1	University of California, Berkeley
893	*Friedmanniomyces simplex*	Dothideo	Asco	14	21	17	13	65	GCA_005059865.1	University of California, Riverside
894	*Scedosporium apiospermum*	Sordario	Asco	16	19	16	14	65	GCA_000732125.1	LUNAM - Angers University
895	*Arthrobotrys oligospora*	Orbilio	Asco	12	25	17	11	65	GCA_004768765.1	Academia Sinica
896	*Arthrobotrys flagrans*	Orbilio	Asco	12	24	16	13	65	GCA_004000055.1	Institute for Applied Biosciences
897	*Panaeolus cyanescens*	Agarico	Basidio	12	19	21	13	65	GCA_002938355.1	Ohio State University
898	*Rhizodiscina lignyota*	Dothideo	Asco	11	30	12	11	64	GCA_010015805.1	DOE Joint Genome Institute
899	*Xenoacremonium recifei*	Sordario	Asco	12	28	13	11	64	GCA_012184525.1	Belgian Coordinated Collection of Microorganisms, BCCM
900	*Neurospora terricola*	Sordario	Asco	13	21	18	12	64	GCA_009805285.1	University of California, Berkeley
901	*Neurospora tetraspora*	Sordario	Asco	13	20	17	14	64	GCA_009806155.1	University of California, Berkeley
902	*Patellaria atrata*	Dothideo	Asco	13	20	16	15	64	GCA_010093705.1	DOE Joint Genome Institute
903	*Auriculariopsis ampla*	Agarico	Basidio	14	24	13	13	64	GCA_007026445.1	DOE Joint Genome Institute
904	*Blumeriella jaapii*	Leotio	Asco	14	26	11	13	64	GCA_009599575.1	Michigan State University
905	*Venturia effusa*	Dothideo	Asco	12	27	11	14	64	GCA_001901625.1	USDA-ARS
906	*Marssonina coronariae*	Leotio	Asco	12	28	9	15	64	GCA_002204255.1	Nanjing Forestry University
907	*Marssonina rosae*	Leotio	Asco	12	28	10	14	64	GCA_011750715.1	Nanjing Forestry University
908	*Trichoderma koningiopsis*	Sordario	Asco	11	23	19	10	63	GCA_002246955.1	INBIOMIS
909	*Trichoderma erinaceum*	Sordario	Asco	11	23	19	10	63	GCA_013365115.1	None
910	*Trichoderma atroviride*	Sordario	Asco	11	23	19	10	63	GCA_001599035.1	RIKEN Center for Life Science Technologies, Division of Genomic Technologies
911	*Acremonium chrysogenum*	Sordario	Asco	15	21	16	11	63	GCA_000769265.1	Ruhr-Universitaet Bochum
912	*Mycosphaerella arachidis*	Dothideo	Asco	13	25	10	15	63	GCA_001297265.1	USDA
913	*Cercospora zeina*	Dothideo	Asco	11	25	14	13	63	GCA_002844615.1	University of Pretoria
914	*Sordaria macrospora*	Sordario	Asco	14	20	15	14	63	GCA_008692325.1	Ruhr-University Bochum
915	*Hyphodiscus sp. D1413*	Leotio	Asco	11	25	14	13	63	GCA_003988895.1	Manaaki Whenua Landcare Research
916	*Stereum hirsutum*	Agarico	Basidio	13	22	13	15	63	GCA_000264905.1	JGI

917	*Neurospora crassa*	Sordario	Asco	12	23	15	13	63	GCA_007478085.1	Ronin Genetics
918	*Tothia fuscella*	Dothideo	Asco	13	26	11	13	63	GCA_010583015.1	DOE Joint Genome Institute
919	*Dactylella cylindrospora*	Orbilio	Asco	12	24	14	13	63	GCA_012184295.1	Laboratory for Conservation and Utilization of Bio-Resources and Key Laboratory for Microbial Diversity in Southwest China, Ministry of Education
920	*Pterula gracilis*	Agarico	Basidio	15	19	14	15	63	GCA_004369125.1	DOE Joint Genome Institute
921	*Pleurotus salmoneostramineus*	Agarico	Basidio	14	21	13	15	63	GCA_002933715.1	Applied Bioscience, Kindai University, Faculty of Agriculture
922	*Sphaerobolus stellatus*	Agarico	Basidio	13	14	18	18	63	GCA_000827215.1	DOE Joint Genome Institute
923	*Epichloe glyceriae*	Sordario	Asco	11	22	19	10	62	GCA_000225285.2	University of Kentucky, Dept of Plant Pathology
924	*Trichoderma hamatum*	Sordario	Asco	11	23	18	10	62	GCA_000331835.2	University of Exeter
925	*Trichoderma gamsii*	Sordario	Asco	11	22	19	10	62	GCA_002894205.1	CSIRO
926	*Talaromyces borbonicus*	Eurotio	Asco	9	24	20	9	62	GCA_002916415.1	University of Naples "Federico II"
927	*Penicillium griseofulvum*	Eurotio	Asco	13	22	15	12	62	GCA_001561935.1	University of Turin
928	*Scytalidium lignicola*	Leotio	Asco	12	22	16	12	62	GCA_002812745.2	IHI Zittau / TU Dresden
929	*Scedosporium dehoogii*	Sordario	Asco	14	18	16	14	62	GCA_002812735.1	GEIHP, UPRES EA 3142
930	*Scedosporium sp. IMV 00882*	Sordario	Asco	14	18	16	14	62	GCA_001931805.1	Jet Propulsion Laboratory, California Institute of Technology
931	*Neurospora tetrasperma*	Sordario	Asco	12	22	15	13	62	GCA_000213195.1	US DOE Joint Genome Institute (JGI-PGF)
932	*Coriolopsis trogii*	Agarico	Basidio	15	20	10	17	62	GCA_007896425.1	Kunming University of Science and Technology
933	*Neurospora sp. CHS-2018b*	Sordario	Asco	13	19	17	13	62	GCA_009806015.1	University of California, Berkeley
934	*Aspergillus sclerotialis*	Eurotio	Asco	11	23	15	12	61	GCA_003589665.1	University of Natural Resources and Life Sciences, Vienna
935	*Mycosphaerella populi*	Dothideo	Asco	12	24	12	13	61	GCA_002153405.1	Canada's Michael Smith Genome Sciences Centre
936	*Talaromyces piceae*	Eurotio	Asco	10	23	16	12	61	GCA_001657655.1	Tianjin Institute of Biotechnology, CAS

937	*Rachicladosporium antarcticum*	Dothideo	Asco	13	17	18	13	61	GCA_002077065.1	University of California, Riverside
938	*Polytolypa hystricis*	Eurotio	Asco	14	18	17	12	61	GCA_002573605.1	Broad Institute
939	*Fragosphaeria purpurea*	Sordario	Asco	15	18	15	13	61	GCA_002778095.1	University of Montana
940	*Polyporus brumalis*	Agarico	Basidio	13	20	12	16	61	GCA_001792895.1	National Institute of Forest Science
941	*Raffaelea lauricola*	Sordario	Asco	14	21	13	13	61	GCA_014183025.1	University of Florida
942	*Polyporus arcularius*	Agarico	Basidio	13	20	12	16	61	GCA_004369055.1	DOE Joint Genome Institute
943	*Dictyopanus pusillus*	Agarico	Basidio	13	16	17	15	61	GCA_013387415.1	INRS-Institut Armand-Frappier
944	*Baeospora myosura*	Agarico	Basidio	13	19	13	16	61	GCA_001179705.1	Royal Botanic Gardens, Kew
945	*Grammothele lineata*	Agarico	Basidio	13	20	13	15	61	GCA_002150815.3	Molecular Biology Lab
946	*Aspergillus chevalieri*	Eurotio	Asco	13	22	12	14	61	GCA_001599875.1	RIKEN Center for Life Science Technologies, Division of Genomic Technologies
947	*Periglandula ipomoeae*	Sordario	Asco	11	22	16	11	60	GCA_000222875.2	University of Kentucky, Dept of Plant Pathology
948	*Ramularia endophylla*	Dothideo	Asco	12	26	11	11	60	GCA_002116395.1	Canada's Michael Smith Genome Sciences Centre
949	*Aspergillus clavatus*	Eurotio	Asco	13	20	16	11	60	GCA_000002715.1	J. Craig Venter Institute
950	*Esteya vermicola*	Sordario	Asco	14	18	15	13	60	GCA_002778215.1	University of Montana
951	*Neurospora discreta*	Sordario	Asco	12	21	15	12	60	GCA_009805215.1	University of California, Berkeley
952	*Dicyma pulvinata*	Sordario	Asco	14	24	12	10	60	GCA_006538405.1	National Agriculture and Food Research Organization
953	*Raffaelea sp. RL272*	Sordario	Asco	13	22	11	14	60	GCA_002777955.1	University of Montana
954	*Aspergillus tritici*	Eurotio	Asco	15	19	13	13	60	GCA_009812425.1	Universidad de Antioquia
955	*Teratosphaeria gauchensis*	Dothideo	Asco	12	25	10	13	60	GCA_007113925.1	University of Pretoria
956	*Arthonia radiata*	Arthonio	Asco	11	22	17	10	60	GCA_002989075.1	Stanford University
957	*Tricholomella constricta*	Agarico	Basidio	13	21	11	15	60	GCA_013368375.1	Lund University
958	*Aspergillus heteromorphus*	Eurotio	Asco	13	27	8	12	60	GCA_003184545.1	DOE Joint Genome Institute
959	*Drechslerella brochopaga*	Orbilio	Asco	11	22	15	12	60	GCA_012184305.1	Laboratory for Conservation and Utilization of Bio-Resources and Key Laboratory for Microbial Diversity in Southwest China, Ministry of Education

960	*Phyllachora maydis*	Sordario	Asco	13	20	13	14	60	GCA_011801745.1	Purdue University, Botany and Plant Pathology
961	*Armillaria borealis*	Agarico	Basidio	12	19	13	16	60	GCA_013427175.2	Genome Research and Education Center, Siberian Federal University
962	*Armillaria ostoyae*	Agarico	Basidio	12	22	12	14	60	GCA_900157425.1	Technische Universitat Munchen - WZW
963	*Trichoderma atrobrunneum*	Sordario	Asco	13	18	17	11	59	GCA_003439915.1	Institute of Sciences of Food Production (ISPA)
964	*Trichoderma guizhouense*	Sordario	Asco	13	18	17	11	59	GCA_002022785.1	Nanjing Agricultural University
965	*Rachicladosporium sp. CCFEE 5018*	Dothideo	Asco	12	17	18	12	59	GCA_002077045.2	University of California, Riverside
966	*Aspergillus saccharolyticus*	Eurotio	Asco	13	24	9	13	59	GCA_003184585.1	DOE Joint Genome Institute
967	*Lophium mytilinum*	Dothideo	Asco	14	18	12	15	59	GCA_010093605.1	DOE Joint Genome Institute
968	*Ploettnerulaceae sp. D365*	Leotio	Asco	13	21	11	14	59	GCA_003988805.1	Manaaki Whenua Landcare Research
969	*Teratosphaeria zuluensis*	Dothideo	Asco	12	24	10	13	59	GCA_007113905.1	University of Pretoria
970	*Pseudophaeomoniella oleicola*	Eurotio	Asco	10	27	10	12	59	GCA_003868215.1	University of Bari "A. Moro"
971	*Dothistroma septosporum*	Dothideo	Asco	12	19	14	14	59	GCA_002236755.2	Massey University
972	*Talaromyces marneffei*	Eurotio	Asco	10	24	14	11	59	GCA_009650675.1	Broad Institute
973	*Sporothrix insectorum*	Sordario	Asco	14	17	15	13	59	GCA_001636815.1	Shanghai Institutes for Biological Sciences, CAS
974	*Amesia nigricolor*	Sordario	Asco	14	17	14	14	59	GCA_004802645.1	Qingdao University
975	*Sporormia fimetaria*	Dothideo	Asco	14	14	19	12	59	GCA_010093795.1	DOE Joint Genome Institute
976	*Aeminium ludgeri*	Dothideo	Asco	14	24	11	10	59	GCA_004216415.1	Centre for Functional Diversity
977	*Dendrothele bispora*	Agarico	Basidio	15	18	11	15	59	GCA_004369135.1	DOE Joint Genome Institute
978	*Armillaria solidipes*	Agarico	Basidio	12	19	12	16	59	GCA_002307675.1	DOE Joint Genome Institute
979	*Trichoderma asperellum*	Sordario	Asco	11	19	18	10	58	GCA_013423425.1	Chubu university
980	*Trichoderma harzianum*	Sordario	Asco	13	17	17	11	58	GCA_010015525.1	The University of Adelaide
981	*Nothophaeocryptopus gaeumannii*	Dothideo	Asco	11	27	6	14	58	GCA_002116385.1	Canada's Michael Smith Genome Sciences Centre
982	*Penicillium coprophilum*	Eurotio	Asco	12	19	15	12	58	GCA_002072405.1	Chalmers University of Technology

983	*Trametes villosa*	Agarico	Basidio	13	16	13	16	58	GCA_002964805.1	Universidade Federal de Minas Gerais
984	*Ganoderma tsugae*	Agarico	Basidio	12	19	12	15	58	GCA_003057275.1	Biotechnology and Nuclear Technology Research Institute
985	*Passalora sequoiae*	Dothideo	Asco	11	23	10	14	58	GCA_013248845.1	USDA
986	*Pseudonectria buxi*	Sordario	Asco	13	25	9	11	58	GCA_003693545.1	USDA-ARS
987	*Tinctoporellus epimiltinus*	Agarico	Basidio	12	20	12	14	58	GCA_900155495.1	BIOTECHNOLOGY RESEARCH INSTITUTE
988	*Aulographum hederae*	Dothideo	Asco	12	22	11	13	58	GCA_010015705.1	DOE Joint Genome Institute
989	*Microcyclosporella mali*	Dothideo	Asco	11	21	15	11	58	GCA_002785985.1	Northwest A&F University
990	*Exutisphaerella laricina*	Dothideo	Asco	13	19	13	13	58	GCA_000504385.2	Canada's Michael Smith Genome Sciences Centre
991	*Delphinella strobiligena*	Dothideo	Asco	11	25	7	15	58	GCA_009982845.1	DOE Joint Genome Institute
992	*Chlorencoelia torta*	Leotio	Asco	12	19	14	13	58	GCA_003988815.1	Manaaki Whenua Landcare Research
993	*Dichomitus squalens*	Agarico	Basidio	12	20	10	16	58	GCA_004307925.1	DOE Joint Genome Institute
994	*Agrocybe cylindracea*	Agarico	Basidio	13	17	14	14	58	GCA_013376435.1	Beijing Institute of Genomics, CAS
995	*Trichocladium griseum*	Sordario	Asco	14	16	16	12	58	GCA_011316235.1	Embrapa Agroenergia
996	*Scedosporium aurantiacum*	Sordario	Asco	14	15	15	14	58	GCA_000812075.1	Australian National University
997	*Flammulina velutipes*	Agarico	Basidio	13	17	16	12	58	GCA_011800155.1	Fujian Agriculture and Forestry University
998	*Coprinellus micaceus*	Agarico	Basidio	13	13	17	15	58	GCA_004369175.1	DOE Joint Genome Institute
999	*Pleurotus pulmonarius*	Agarico	Basidio	13	18	13	14	58	GCA_012980535.1	Academia Sinica
1000	*Ceratobasidium theobromae*	Agarico	Basidio	13	22	10	13	58	GCA_009078325.1	Beltsville Agricultural Research Center
1001	*Armillaria cepistipes*	Agarico	Basidio	12	19	13	14	58	GCA_900157415.1	Technische Universitat Munchen - WZW
1002	*Aspergillus cristatus*	Eurotio	Asco	12	21	12	13	58	GCA_001693355.1	Shandong Freda Pharmaceutical Group Corporation
1003	*Rhizoctonia solani*	Agarico	Basidio	16	20	7	15	58	GCA_001899475.2	Universiti Kebangsaan Malaysia
1004	*Ascobolus immersus*	Pezizo	Asco	14	15	18	11	58	GCA_003788565.2	DOE Joint Genome Institute
1005	*Tulasnella calospora*	Agarico	Basidio	12	19	14	13	58	GCA_000827465.1	DOE Joint Genome Institute

1006	*Armillaria fuscipes*	Agarico	Basidio	14	16	11	17	58	GCA_001679825.1	Forestry and Agricultural Biotechnology Institute, University of Pretoria
1007	*Cantharellus appalachiensis*	Agarico	Basidio	10	21	18	8	57	GCA_003314335.1	Kunming University of Science and Technology
1008	*Trichoderma sp. IMV 00454*	Sordario	Asco	13	17	16	11	57	GCA_001931985.1	Jet Propulsion Laboratory, California Institute of Technology
1009	*Penicillium sp. OUCMDZ-019*	Eurotio	Asco	10	23	12	12	57	GCA_011750695.1	ocean university of China
1010	*[Talaromyces] leycettanus*	Eurotio	Asco	11	20	16	10	57	GCA_000787455.1	none
1011	*Aspergillus campestris*	Eurotio	Asco	12	19	15	11	57	GCA_002847485.1	DOE Joint Genome Institute
1012	*Lentinus tigrinus*	Agarico	Basidio	13	18	11	15	57	GCA_003813185.1	DOE Joint Genome Institute
1013	*Ganoderma boninense*	Agarico	Basidio	12	17	13	15	57	GCA_002900995.2	PT SMART Tbk
1014	*Teratosphaeria nubilosa*	Dothideo	Asco	11	24	10	12	57	GCA_010093825.1	DOE Joint Genome Institute
1015	*Chlorociboria aeruginascens*	Leotio	Asco	12	17	16	12	57	GCA_002276475.2	IHI Zittau / TU Dresden
1016	*Zymoseptoria pseudotritici*	Dothideo	Asco	11	23	14	9	57	GCA_000223685.2	University of Aarhus, Bioinformatics Research Center
1017	*Amylostereum areolatum*	Agarico	Basidio	13	19	10	15	57	GCA_012932865.1	Beijing Forestry University
1018	*Ophiostoma piceae*	Sordario	Asco	12	22	12	11	57	GCA_000410735.1	University of British Columbia
1019	*Myriangium duriaei*	Dothideo	Asco	12	21	12	12	57	GCA_010093895.1	DOE Joint Genome Institute
1020	*Trametes pubescens*	Agarico	Basidio	13	17	11	16	57	GCA_001895945.1	CBS-KNAW Fungal Biodiversity Centre Institute of the Royal Netherlands Academy of Arts and Science
1021	*Galerina marginata*	Agarico	Basidio	12	17	13	15	57	GCA_000697645.1	DOE Joint Genome Institute
1022	*Agrocybe pediades*	Agarico	Basidio	12	16	15	14	57	GCA_013053245.1	Lund University
1023	*Leucocalocybe mongolica*	Agarico	Basidio	13	16	12	16	57	GCA_013420905.1	Jilin Agricultural University
1024	*Trichoderma lentiforme*	Sordario	Asco	13	16	16	11	56	GCA_011066345.1	Embrapa Agroenergia
1025	*Talaromyces atroroseus*	Eurotio	Asco	9	24	13	10	56	GCA_001907595.1	Technical University of Denmark
1026	*Ramularia collo-cygni*	Dothideo	Asco	11	19	13	13	56	GCA_900074925.1	Technische Universitat Munchen - WZW
1027	*Ganoderma multipileum*	Agarico	Basidio	12	18	11	15	56	GCA_000338015.1	Ganoderma lucidum Research Consortium

1028	*Aspergillus taichungensis*	Eurotio	Asco	12	19	14	11	56	GCA_002850765.1	DOE Joint Genome Institute
1029	*Mycosphaerella sp. Ston1*	Dothideo	Asco	11	23	7	15	56	GCA_000504405.2	Canada's Michael Smith Genome Sciences Centre
1030	*Geosmithia putterillii*	Sordario	Asco	12	18	10	16	56	GCA_900188575.1	UNIVERSITY OF NEW HAMPSHIRE
1031	*Hortaea thailandica*	Dothideo	Asco	12	24	9	11	56	GCA_005059885.1	University of California, Riverside
1032	*Aspergillus rambellii*	Eurotio	Asco	12	21	13	10	56	GCA_000986645.1	USDA-ARS-SRRC
1033	*Sphaerulina musiva*	Dothideo	Asco	10	25	7	14	56	GCA_000320565.2	JGI
1034	*Trametes polyzona*	Agarico	Basidio	13	16	12	15	56	GCA_001939255.1	Universidad Nacional Agraria La Molina,
1035	*Ganoderma sinense*	Agarico	Basidio	13	16	11	16	56	GCA_002760635.1	The Institute of Medicinal Plant Development, China
1036	*Aspergillus coremiiformis*	Eurotio	Asco	11	25	9	11	56	GCA_009193565.1	DOE Joint Genome Institute
1037	*Ganoderma lucidum*	Agarico	Basidio	12	18	11	15	56	GCA_000271565.1	The Institute of Medicinal Plant Development
1038	*Lentinus polychrous*	Agarico	Basidio	12	18	13	13	56	GCA_000787475.1	none
1039	*Aspergillus glaucus*	Eurotio	Asco	11	23	8	14	56	GCA_001890805.1	DOE Joint Genome Institute
1040	*Rhizophlyctis rosea*	Chytridio	Chytridio	14	17	14	11	56	GCA_002214945.1	DTU
1041	*Fibularhizoctonia sp. CBS 109695*	Agarico	Basidio	12	15	13	15	55	GCA_001630335.1	DOE Joint Genome Institute
1042	*Rasamsonia emersonii*	Eurotio	Asco	10	21	13	11	55	GCA_000968595.1	DSM Bio-based Products & Services B.V.
1043	*Teratosphaeria pseudoeucalypti*	Dothideo	Asco	11	23	9	12	55	GCA_013403795.1	University of Pretoria
1044	*Zymoseptoria brevis*	Dothideo	Asco	11	21	14	9	55	GCA_000966595.1	Christian-Albrechts University of Kiel
1045	*Zymoseptoria tritici*	Dothideo	Asco	11	21	13	10	55	GCA_900184105.1	ETH
1046	*Sodiomyces alkalinus*	Sordario	Asco	13	13	17	12	55	GCA_003711515.1	DOE Joint Genome Institute
1047	*Aspergillus ochraceoroseus*	Eurotio	Asco	12	21	12	10	55	GCA_002846915.2	DOE Joint Genome Institute
1048	*Trametes versicolor*	Agarico	Basidio	13	16	11	15	55	GCA_000271585.1	JGI
1049	*Trametes sp. AH28-2*	Agarico	Basidio	13	16	10	16	55	GCA_001304625.1	Anhui University
1050	*Mycosphaerella sp. PB-2012b*	Dothideo	Asco	10	23	8	14	55	GCA_002116345.1	Canada's Michael Smith Genome Sciences Centre
1051	*Moniliophthora roreri*	Agarico	Basidio	13	16	12	14	55	GCA_001466705.1	Purdue University
1052	*Morchella sp. a71*	Pezizo	Asco	15	20	9	11	55	GCA_013407065.1	Chongqing Normal University
1053	*Coccinonectria pachysandricola*	Sordario	Asco	12	23	9	11	55	GCA_013283235.1	USDA-ARS

1054	*Phlebiopsis gigantea*	Agarico	Basidio	13	14	12	16	55	GCA_000832265.1	DOE Joint Genome Institute
1055	*Heterobasidion irregulare*	Agarico	Basidio	12	18	11	14	55	GCA_000320585.2	JGI
1056	*Chondrostereum purpureum*	Agarico	Basidio	13	16	11	15	55	GCA_004354395.1	Internationales Hochschulinstitut Zittau
1057	*Hydnomerulius pinastri*	Agarico	Basidio	15	15	10	15	55	GCA_000827185.1	DOE Joint Genome Institute
1058	*Ophiostoma novo-ulmi*	Sordario	Asco	13	20	10	12	55	GCA_000317715.1	Association of Biomolecular Resource Facilities
1059	*Crucibulum laeve*	Agarico	Basidio	14	14	13	14	55	GCA_004379715.1	DOE Joint Genome Institute
1060	*Gymnopus androsaceus*	Agarico	Basidio	10	18	15	12	55	GCA_009733575.1	DOE Joint Genome Institute
1061	*Lepista sordida*	Agarico	Basidio	12	15	12	16	55	GCA_900168675.1	UNIVERSITY OF BRISTOL
1062	*Porodaedalea pini*	Agarico	Basidio	12	17	11	15	55	GCA_002794775.1	Academia Sinica
1063	*Pleurotus tuoliensis*	Agarico	Basidio	12	17	11	15	55	GCA_003243755.1	Northeast Normal University
1064	*Trichoderma virens*	Sordario	Asco	12	14	17	11	54	GCA_001835465.1	Bhabha Atomic Research Centre
1065	*Lecanosticta acicola*	Dothideo	Asco	11	18	11	14	54	GCA_002441625.1	Mississippi State University
1066	*Saccharata proteae*	Dothideo	Asco	11	23	7	13	54	GCA_010015785.1	DOE Joint Genome Institute
1067	*Pseudonectria foliicola*	Sordario	Asco	11	24	9	10	54	GCA_002911195.1	USDA
1068	*Teratosphaeria destructans*	Dothideo	Asco	11	23	9	11	54	GCA_010367415.1	Forestry and Agricultural Biotechnology Institute (FABI), University of Pretoria
1069	*Taxomyces andreanae*	0	Asco	13	15	11	15	54	GCA_001969225.1	Fraunhofer
1070	*Trametes sanguinea*	Agarico	Basidio	13	15	10	16	54	GCA_008973685.1	School of Bioscience and Technology
1071	*Trametes coccinea*	Agarico	Basidio	13	15	10	16	54	GCA_002092935.1	DOE Joint Genome Institute
1072	*Penicillium digitatum*	Eurotio	Asco	11	23	10	10	54	GCA_012295545.1	Huazhong Agricultural University
1073	*Trametes hirsuta*	Agarico	Basidio	12	16	12	14	54	GCA_001302255.2	A.N. Bach Institute of Biochemistry
1074	*Agaricales sp. HM26-F1*	Agarico	Basidio	12	19	10	13	54	GCA_002718315.1	Shanghai Academy of Agricultural Sciences
1075	*Gymnopilus dilepis*	Agarico	Basidio	14	12	14	14	54	GCA_002938385.1	Ohio State University
1076	*Pleurotus citrinopileatus*	Agarico	Basidio	12	16	10	16	54	GCA_003314595.1	Kunming University of Science and Technology

1077	*Pleurotus eryngii*	Agarico	Basidio	12	18	11	13	54	GCA_001717165.1	Shanghai Academy of Agricultural Sciences
1078	*Lyophyllum shimeji*	Agarico	Basidio	13	14	10	16	53	GCA_001950515.1	RIKEN Center for Life Science Technologies, Division of Genomic Technologies
1079	*Zymoseptoria ardabiliae*	Dothideo	Asco	11	19	13	10	53	GCA_000223765.2	University of Aarhus, Bioinformatics Research Center
1080	*Cenococcum geophilum*	Dothideo	Asco	13	15	10	15	53	GCA_001692895.1	DOE Joint Genome Institute
1081	*Agrocybe aegerita*	Agarico	Basidio	12	14	13	14	53	GCA_902728275.1	Integrative Pilzforschung
1082	*Lineolata rhizophorae*	Dothideo	Asco	14	10	16	13	53	GCA_010093515.1	DOE Joint Genome Institute
1083	*Phanerochaete chrysosporium*	Agarico	Basidio	13	12	12	16	53	GCA_001910725.1	National Institute of Forest Science
1084	*Fusarium ventricosum*	Sordario	Asco	11	20	10	12	53	GCA_013623725.1	US Department of Agriculture, Agriculture Research Service
1085	*Heterobasidion parviporum*	Agarico	Basidio	12	17	10	14	53	GCA_002994785.1	University of Helsinki
1086	*Trichodelitschia bisporula*	Dothideo	Asco	10	25	8	10	53	GCA_010356995.1	DOE Joint Genome Institute
1087	*Heterobasidion annosum*	Agarico	Basidio	12	17	10	14	53	GCA_000633895.1	Seoul National University
1088	*Phanerochaete carnosa*	Agarico	Basidio	12	12	14	15	53	GCA_000300595.1	DOE Joint Genome Institute
1089	*Pleurotus ostreatus*	Agarico	Basidio	11	16	13	13	53	GCA_003313235.2	Chinese Academy of Agricultural Sciences
1090	*Gymnopus luxurians*	Agarico	Basidio	12	17	11	13	53	GCA_000827265.1	DOE JGI
1091	*Leucoagaricus leucothites*	Agarico	Basidio	12	14	12	15	53	GCA_013368445.1	Lund University
1092	*Trichoderma pleuroti*	Sordario	Asco	13	13	15	11	52	GCA_001721665.1	University of Pecs
1093	*Ganoderma sp. BRIUMSc*	Agarico	Basidio	12	16	10	14	52	GCA_008694245.1	Universiti Malaysia Sabah
1094	*Penicillium italicum*	Eurotio	Asco	12	21	8	11	52	GCA_000769765.1	Center for Genomic Regulation (CRG)
1095	*Lyophyllum decastes*	Agarico	Basidio	13	16	9	14	52	GCA_001950495.1	RIKEN Center for Life Science Technologies, Division of Genomic Technologies
1096	*Hericium coralloides*	Agarico	Basidio	12	13	12	15	52	GCA_003675405.1	Tibet Vocational Technical College
1097	*Trametes cinnabarina*	Agarico	Basidio	13	14	10	15	52	GCA_000787515.1	Aalborg University
1098	*Coprinellus angulatus*	Agarico	Basidio	11	11	15	15	52	GCA_013368325.1	Lund University
1099	*Phlebia tremellosa*	Agarico	Basidio	14	10	11	17	52	GCA_011032875.1	Ningxia University

1100	*Steccherinum ochraceum*	Agarico	Basidio	12	13	12	15	52	GCA_004332605.1	The Federal Research Centre "Fundamentals of Biotechnology" of the Russian Academy of Sciences
1101	*Thielaviopsis ethacetica*	Sordario	Asco	12	17	12	11	52	GCA_001599055.1	RIKEN Center for Life Science Technologies, Division of Genomic Technologies
1102	*Macrolepiota dolichaula*	Agarico	Basidio	12	13	12	15	52	GCA_003315915.1	Kunming University of Science and Technology
1103	*Cantharellus cibarius*	Agarico	Basidio	8	20	16	7	51	GCA_003521295.1	Kunming University of Science and Technology
1104	*Trichoderma viride*	Sordario	Asco	11	13	16	11	51	GCA_007896495.1	Institute of Biology, Shnaodong Academy of Science, China
1105	*Trichoderma sp. TW21990_1*	Sordario	Asco	12	12	16	11	51	GCA_010015515.1	The University of Adelaide
1106	*Friedmanniomyces endolithicus*	Dothideo	Asco	14	13	14	10	51	GCA_005059855.1	University of California, Riverside
1107	*Penicillium paneum*	Eurotio	Asco	12	18	10	11	51	GCA_000577715.1	INRA-LIPM
1108	*Zymoseptoria passerinii*	Dothideo	Asco	11	17	13	10	51	GCA_000223825.2	University of Aarhus, Bioinformatics Research Center
1109	*Lignosus rhinocerotis*	Agarico	Basidio	12	14	11	14	51	GCA_000743315.1	University of Malaya
1110	*Raffaelea quercivora*	Sordario	Asco	10	20	8	13	51	GCA_002778125.1	University of Montana
1111	*Dentipellis fragilis*	Agarico	Basidio	12	14	11	14	51	GCA_004679275.1	IHI Zittau / TU Dresden
1112	*Baudoinia panamericana*	Dothideo	Asco	11	16	13	11	51	GCA_000338955.1	JGI
1113	*Irpex lacteus*	Agarico	Basidio	13	13	11	14	51	GCA_001986395.2	Anhui university
1114	*Sphaerosporella brunnea*	Pezizo	Asco	14	15	8	14	51	GCA_008704415.1	DOE Joint Genome Institute
1115	*Raffaelea arxii*	Sordario	Asco	12	16	10	12	50	GCA_002778165.1	University of Montana
1116	*Mytilinidion resinicola*	Dothideo	Asco	13	12	10	15	50	GCA_010093595.1	DOE Joint Genome Institute
1117	*Aspergillus sp. HF37*	Eurotio	Asco	12	16	11	11	50	GCA_003698115.1	University of Natural Resources and Life Sciences, Vienna
1118	*Thermoascaceae sp. COH1141*	Eurotio	Asco	8	20	9	13	50	GCA_003123655.1	Jet Propulsion Laboratory, California Institute of Technology
1119	*Pleurotus ostreatoroseus*	Agarico	Basidio	13	14	9	14	50	GCA_005298045.1	Amazonian macromicets: knowing their diversity and evaluating their biotechnological

											potential in a sustainable way
1120	*Gloeophyllum trabeum*	Agarico	Basidio	11	16	10	13	50	GCA_000344685.1	JGI
1121	*Gloeostereum incarnatum*	Agarico	Basidio	11	14	10	15	50	GCA_010588315.1	Jilin Agricultural University
1122	*Stropharia rugosoannulata*	Agarico	Basidio	13	10	12	15	50	GCA_003314255.1	Kunming University of Science and Technology
1123	*Grosmannia penicillata*	Sordario	Asco	10	21	7	12	50	GCA_002778075.1	University of Montana
1124	*Obba rivulosa*	Agarico	Basidio	12	11	11	16	50	GCA_001687445.1	DOE Joint Genome Institute
1125	*Hypsizygus marmoreus*	Agarico	Basidio	13	14	10	13	50	GCA_013433165.1	Fujian Agriculture and Forestry University
1126	*Neonothopanus nambi*	Agarico	Basidio	12	10	12	16	50	GCA_003987895.1	Universidade de Sao Paulo
1127	*Armillaria gallica*	Agarico	Basidio	12	14	10	14	50	GCA_012064365.1	Yunnan Minzu University
1128	*Hypholoma sublateritium*	Agarico	Basidio	12	10	14	14	50	GCA_000827495.1	JGI
1129	*Serendipita vermifera*	Agarico	Basidio	14	14	11	11	50	GCA_003073295.1	DOE Joint Genome Institute
1130	*Trichoderma arundinaceum*	Sordario	Asco	12	12	15	10	49	GCA_003012105.1	USDA, ARS, NCAUR
1131	*Trichoderma reesei*	Sordario	Asco	12	10	16	11	49	GCA_000167675.2	DOE Joint Genome Institute
1132	*Trichoderma longibrachiatum*	Sordario	Asco	12	10	15	12	49	GCA_000332775.1	Shandong University
1133	*Trichoderma koningii*	Sordario	Asco	12	10	15	12	49	GCA_001950475.1	RIKEN Center for Life Science Technologies, Division of Genomic Technologies
1134	*Penicillium roqueforti*	Eurotio	Asco	11	19	9	10	49	GCA_001599855.1	RIKEN Center for Life Science Technologies, Division of Genomic Technologies
1135	*Dentipellis sp. KUC8613*	Agarico	Basidio	12	14	10	13	49	GCA_002286715.1	Korea University
1136	*Penicilliopsis zonata*	Eurotio	Asco	10	19	11	9	49	GCA_001890105.1	DOE Joint Genome Institute
1137	*Coniophora puteana*	Agarico	Basidio	13	14	8	14	49	GCA_000271625.1	DOE Joint Genome Institute
1138	*Ochroconis constricta*	Dothideo	Asco	12	16	9	12	49	GCA_000611715.1	UNIVERSITY OF MALAYA
1139	*Plicaturopsis crispa*	Agarico	Basidio	12	16	6	15	49	GCA_000827205.1	DOE Joint Genome Institute
1140	*Leptographium lundbergii*	Sordario	Asco	10	22	5	12	49	GCA_001455505.1	University of Pretoria
1141	*Exophiala lecanii-corni*	Eurotio	Asco	10	16	9	14	49	GCA_003955835.1	Naval Research Laboratory
1142	*Grifola frondosa*	Agarico	Basidio	13	12	8	16	49	GCA_001683735.1	Korea University
1143	*Psathyrella aberdarensis*	Agarico	Basidio	12	7	15	15	49	GCA_004126415.1	IHI Zittau / TU Dresden

1144	*Thielaviopsis punctulata*	Sordario	Asco	11	16	10	12	49	GCA_002925815.1	Forestry and Agricultural Biotechnology Institute
1145	*Pochonia chlamydosporia*	Sordario	Asco	11	13	12	12	48	GCA_001653235.2	The Institute of Vegetables and Flowers CAAS
1146	*Trichoderma parareesei*	Sordario	Asco	11	11	16	10	48	GCA_001050175.1	Vienna University of Technology
1147	*Byssochlamys sp. AF001*	Eurotio	Asco	7	23	9	9	48	GCA_002914405.1	University of Oklahoma
1148	*Choiromyces venosus*	Pezizo	Asco	12	17	7	12	48	GCA_003788595.2	DOE Joint Genome Institute
1149	*Gelatoporia subvermispora*	Agarico	Basidio	12	11	10	15	48	GCA_000320605.2	DOE Joint Genome Institute
1150	*Pseudogymnoascus destructans*	Leotio	Asco	10	21	8	9	48	GCA_001641265.1	US Forest Service
1151	*Verruconis gallopava*	Dothideo	Asco	9	19	7	13	48	GCA_000836295.1	Broad Institute
1152	*Thielaviopsis euricoi*	Sordario	Asco	10	17	11	10	48	GCA_001599615.1	RIKEN Center for Life Science Technologies, Division of Genomic Technologies
1153	*Macrocybe gigantea*	Agarico	Basidio	11	14	9	14	48	GCA_011319805.1	Yunnan Agricultural University
1154	*Leucoagaricus sp. SymC.cos*	Agarico	Basidio	11	11	12	14	48	GCA_001563735.1	BGI
1155	*Bretziella fagacearum*	Sordario	Asco	12	15	9	12	48	GCA_002018255.1	Forestry and Agricultural Biotechnology Institute
1156	*Pleurotus tuber-regium*	Agarico	Basidio	13	11	10	14	48	GCA_014058305.1	Huazhong Agricultural University
1157	*Termitomyces sp. JCM 13351*	Agarico	Basidio	12	9	12	15	48	GCA_001972325.1	RIKEN Center for Life Science Technologies, Division of Genomic Technologies
1158	*Trichoderma brevicompactum*	Sordario	Asco	12	11	14	10	47	GCA_003012085.1	USDA, ARS, NCAUR
1159	*Penicillium carneum*	Eurotio	Asco	11	18	8	10	47	GCA_000577495.1	INRA-LIPM
1160	*Byssochlamys nivea*	Eurotio	Asco	8	20	9	10	47	GCA_003116535.1	Cornell University
1161	*Geosmithia flava*	Sordario	Asco	10	17	7	13	47	GCA_900188565.1	UNIVERSITY OF NEW HAMPSHIRE
1162	*Hericium erinaceus*	Agarico	Basidio	12	14	7	14	47	GCA_006506795.2	Institute of Bast Fiber Crops, Chinese Academy of Agricultural Sciences
1163	*Chaetomium thermophilum*	Sordario	Asco	11	13	14	9	47	GCA_000221225.1	EMBL
1164	*Heliocybe sulcata*	Agarico	Basidio	9	16	11	11	47	GCA_004369045.1	DOE Joint Genome Institute
1165	*Raffaelea quercus-mongolicae*	Sordario	Asco	10	17	7	13	47	GCA_002215975.1	Seoul National University
1166	*Hericium alpestre*	Agarico	Basidio	12	12	8	15	47	GCA_004681135.1	IHI Zittau / TU Dresden
1167	*Serendipita indica*	Agarico	Basidio	12	13	10	12	47	GCA_000313545.1	Institute of Bioinformatics and

										Systems Biology, Helmholtz Zentrum München, Neuherberg, Germany
1168	*Psilocybe cyanescens*	Agarico	Basidio	11	9	12	15	47	GCA_002938375.1	Ohio State University
1169	*Mycosphaerella populicola*	Dothideo	Asco	8	20	6	12	46	GCA_000291705.1	University of British Columbia
1170	*Saitozyma sp. JCM 24511*	Tremello	Basidio	5	22	12	7	46	GCA_001600855.1	RIKEN Center for Life Science Technologies, Division of Genomic Technologies
1171	*Coprinopsis cinerea*	Agarico	Basidio	11	8	15	12	46	GCA_000182895.1	Broad Institute
1172	*Pholiota adiposa*	Agarico	Basidio	11	11	11	13	46	GCA_009935795.1	Dezhou University
1173	*Ophiostoma ips*	Sordario	Asco	8	20	8	10	46	GCA_002917055.1	University of Pretoria
1174	*Sarcomyxa edulis*	Agarico	Basidio	10	14	9	13	46	GCA_009761415.1	Engineering Research Center of Chinese Ministry of Education for Edible and Medicinal Fungi
1175	*Thielaviopsis musarum*	Sordario	Asco	12	13	9	12	46	GCA_001513885.1	University of Pretoria
1176	*Coniferiporia sulphurascens*	Agarico	Basidio	13	10	9	14	46	GCA_002794785.1	Biodiversity Research Center, Academia Sinica
1177	*Gymnopus confluens*	Agarico	Basidio	11	13	9	13	46	GCA_013368555.1	Lund University
1178	*Calcarisporium arbuscula*	Sordario	Asco	10	12	12	11	45	GCA_009828645.1	Zhejiang University
1179	*Grosmannia clavigera*	Sordario	Asco	10	19	4	12	45	GCA_000143105.2	Michael Smith Laboratories at UBC
1180	*Exophiala xenobiotica*	Eurotio	Asco	9	14	10	12	45	GCA_000835505.1	Broad Institute
1181	*Bondarzewia mesenterica*	Agarico	Basidio	12	9	7	17	45	GCA_004802705.1	IHI Zittau / TU Dresden
1182	*Knoxdaviesia proteae*	Sordario	Asco	13	7	13	12	45	GCA_001510565.1	University of Stellenbosch
1183	*Fomitopsis palustris*	Agarico	Basidio	10	12	10	13	45	GCA_001937815.1	National Institute of Forest Science
1184	*Botryobasidium botryosum*	Agarico	Basidio	14	6	11	14	45	GCA_000697705.1	JGI
1185	*Athelia rolfsii*	Agarico	Basidio	10	16	9	10	45	GCA_002940785.1	Southwest University
1186	*Coprinopsis marcescibilis*	Agarico	Basidio	11	7	13	14	45	GCA_004369085.1	DOE Joint Genome Institute
1187	*Volvariella volvacea*	Agarico	Basidio	12	9	11	13	45	GCA_001691835.3	Harvard University
1188	*Berkeleyomyces basicola*	Sordario	Asco	11	15	6	13	45	GCA_003671435.1	Forestry and Agricultural Biotechnology Institute
1189	*Jaapia argillacea*	Agarico	Basidio	14	9	8	14	45	GCA_000697665.1	DOE Joint Genome Institute
1190	*Fomitiporia mediterranea*	Agarico	Basidio	12	11	6	16	45	GCA_000271605.1	JGI
1191	*Termitomyces sp. MG148*	Agarico	Basidio	13	10	9	13	45	GCA_003313785.1	Kunming University of Science and Technology

1192	*Simplicillium aogashimaense*	Sordario	Asco	11	12	9	12	44	GCA_012273805.1	AgResearch
1193	*Phialophora attinorum*	Eurotio	Asco	9	10	12	13	44	GCA_001299255.1	CBS-KNAW Fungal Biodiversity Centre
1194	*Cladosporiaceae sp. IMV 00236*	Dothideo	Asco	7	17	7	13	44	GCA_001931875.2	Jet Propulsion Laboratory, California Institute of Technology
1195	*Malbranchea cinnamomea*	Eurotio	Asco	10	16	9	9	44	GCA_900128795.2	CHALMERS UNIVERSITY OF TECHNOLOGY
1196	*Knoxdaviesia capensis*	Sordario	Asco	12	6	15	11	44	GCA_001510575.1	University of Stellenbosch
1197	*Eurotiomycetes sp.*	Eurotio	Asco	9	18	7	10	44	GCA_002917005.1	Jill Banfield's Lab at Berkeley
1198	*Schizopora paradoxa*	Agarico	Basidio	13	9	8	14	44	GCA_001020605.1	DOE Joint Genome Institute
1199	*Sistotremastrum suecicum*	Agarico	Basidio	14	3	11	16	44	GCA_001632355.1	DOE Joint Genome Institute
1200	*Termitomyces sp. MG145*	Agarico	Basidio	11	8	11	14	44	GCA_003313055.1	Kunming University of Science and Technology
1201	*Termitomyces eurrhizus*	Agarico	Basidio	13	8	9	14	44	GCA_003316525.1	Kunming University of Science and Technology
1202	*Purpureocillium lilacinum*	Sordario	Asco	9	13	10	11	43	GCA_001468795.1	The Energy and Resources Institute
1203	*Cyphellophora europaea*	Eurotio	Asco	9	10	12	12	43	GCA_000365145.2	Broad Institute
1204	*Saitozyma podzolica*	Tremello	Basidio	5	20	12	6	43	GCA_003942215.1	Karlsruhe Institute of Technology
1205	*Phialophora verrucosa*	Eurotio	Asco	8	11	13	11	43	GCA_002099365.1	Peking University First Hospital
1206	*Amorphotheca resinae*	Leotio	Asco	7	18	8	10	43	GCA_001270345.1	East China University of Science and Technology
1207	*Phialophora americana*	Eurotio	Asco	9	8	15	11	43	GCA_000835435.1	Broad Institute
1208	*Knufia petricola*	Eurotio	Asco	9	17	7	10	43	GCA_002319055.1	University of Natural Resources and Life Sciences
1209	*Phlebia centrifuga*	Agarico	Basidio	13	6	7	17	43	GCA_001913855.2	CBS-KNAW Fungal Biodiversity Centre Institute of the Royal Netherlands Academy of Arts and Science
1210	*Coprinopsis strossmayeri*	Agarico	Basidio	11	6	13	13	43	GCA_900156845.1	UNIVERSITY OF BRISTOL
1211	*Davidsoniella australis*	Sordario	Asco	10	15	6	12	43	GCA_009806335.1	Forestry and Agricultural Biotechnology Institute
1212	*Davidsoniella neocaledoniae*	Sordario	Asco	10	16	5	12	43	GCA_009806295.1	Forestry and Agricultural Biotechnology Institute

1213	*Davidsoniella virescens*	Sordario	Asco	10	16	5	12	43	GCA_001513805.1	University of Pretoria, Forestry and Agricultural Biotechnology Institute
1214	*Davidsoniella eucalypti*	Sordario	Asco	10	15	5	13	43	GCA_004009845.1	Forestry and Agricultural Biotechnology Institute
1215	*Termitomyces sp. J132*	Agarico	Basidio	11	7	11	14	43	GCA_001263195.1	BGI
1216	*Collybia sp. MG36*	Agarico	Basidio	10	11	7	15	43	GCA_003313185.1	Kunming University of Science and Technology
1217	*Sanghuangporus baumii*	Agarico	Basidio	12	9	6	16	43	GCA_001481415.2	China University of Petroleum
1218	*Podosphaera xanthii*	Leotio	Asco	8	13	8	13	42	GCA_010015925.1	National Academy of Agricultural Science, Rural Development Adminstration
1219	*Epichloe uncinata*	Sordario	Asco	9	10	11	12	42	GCA_001043855.1	University of Kentucky
1220	*Claviceps fusiformis*	Sordario	Asco	8	13	12	9	42	GCA_000223055.1	University of Kentucky, Dept of Plant Pathology
1221	*Penicillium decumbens*	Eurotio	Asco	8	16	6	12	42	GCA_002072245.1	Chalmers University of Technology
1222	*Cladophialophora carrionii*	Eurotio	Asco	8	10	13	11	42	GCA_000365165.2	Broad Institute
1223	*Cladophialophora yegresii*	Eurotio	Asco	8	11	11	12	42	GCA_000585515.1	Broad Institute
1224	*Pluteus cervinus*	Agarico	Basidio	13	7	7	15	42	GCA_004369065.1	DOE Joint Genome Institute
1225	*Ceratocystis manginecans*	Sordario	Asco	10	15	6	11	42	GCA_006408425.1	University of Pretoria
1226	*Ceratocystis eucalypticola*	Sordario	Asco	10	15	6	11	42	GCA_001513815.1	University of pretoria
1227	*Ceratocystis cacaofunesta*	Sordario	Asco	10	15	6	11	42	GCA_002776505.1	University of Campinas
1228	*Ceratocystis platani*	Sordario	Asco	10	15	6	11	42	GCA_000978885.1	University of Neuchatel
1229	*Ceratocystis albifundus*	Sordario	Asco	10	15	6	11	42	GCA_002742255.2	University of Pretoria
1230	*Ceratocystis fimbriata*	Sordario	Asco	10	15	6	11	42	GCA_009914735.1	Universidade Federal de Vicosa
1231	*Ceratocystis harringtonii*	Sordario	Asco	10	15	6	11	42	GCA_002018265.1	Forestry and Agricultural Biotechnology Institute
1232	*Macrocystidia cucumis*	Agarico	Basidio	12	9	7	14	42	GCA_001179725.1	Royal Botanic Gardens, Kew
1233	*Rickenella mellea*	Agarico	Basidio	11	12	3	16	42	GCA_004355085.1	DOE Joint Genome Institute
1234	*Sistotremastrum niveocremeum*	Agarico	Basidio	14	3	10	15	42	GCA_001630475.1	DOE Joint Genome Institute
1235	*Clitocybe nebularis*	Agarico	Basidio	11	9	8	14	42	GCA_900068955.1	Royal Botanic Gardens, Kew
1236	*Lecanicillium psalliotae*	Sordario	Asco	10	12	8	11	41	GCA_002796755.1	Western Sydney University
1237	*Thermoascus crustaceus*	Eurotio	Asco	9	13	9	10	41	GCA_001599835.1	RIKEN Center for Life Science Technologies,

										Division of Genomic Technologies
1238	*Geosmithia morbida*	Sordario	Asco	10	14	5	12	41	GCA_900108815.1	UNIVERSITY OF NEW HAMPSHIRE
1239	*Leptographium procerum*	Sordario	Asco	10	18	2	11	41	GCA_000806385.1	University of Pretoria
1240	*Fomitopsis pinicola*	Agarico	Basidio	9	12	10	10	41	GCA_000344655.2	DOE Joint Genome Institute
1241	*Antrodiella citrinella*	Agarico	Basidio	12	7	10	12	41	GCA_004802725.1	IHI Zittau / TU Dresden
1242	*Daedalea quercina*	Agarico	Basidio	9	10	11	11	41	GCA_001632345.1	DOE Joint Genome Institute
1243	*Exophiala sp.*	Eurotio	Asco	8	13	8	12	41	GCA_004026505.1	Jill Banfield's Lab at Berkeley
1244	*Pholiota microspora*	Agarico	Basidio	10	9	10	12	41	GCA_003314615.1	Kunming University of Science and Technology
1245	*Pecoramyces ruminatium*	Neocallimastigo	Chytridio	10	15	16	0	41	GCA_000412615.1	Oklahoma State University
1246	*Chalaropsis thielavioides*	Sordario	Asco	10	14	5	12	41	GCA_001599435.1	RIKEN Center for Life Science Technologies, Division of Genomic Technologies
1247	*Lepiota venenata*	Agarico	Basidio	11	8	8	14	41	GCA_004296355.1	Kunming Institute of Botany
1248	*Aspergillus tanneri*	Eurotio	Asco	11	16	2	11	40	GCA_003426965.1	JCVI
1249	*Exophiala oligosperma*	Eurotio	Asco	8	10	11	11	40	GCA_000835515.1	Broad Institute
1250	*Claviceps paspali*	Sordario	Asco	7	12	11	10	40	GCA_013435705.1	INIA
1251	*Eremomyces bilateralis*	Dothideo	Asco	8	13	8	11	40	GCA_010015585.1	DOE Joint Genome Institute
1252	*Epichloe bromicola*	Sordario	Asco	8	11	10	11	40	GCA_002319005.1	University of Kentucky
1253	*Fomitopsis rosea*	Agarico	Basidio	10	9	9	12	40	GCA_004679265.1	IHI Zittau / TU Dresden
1254	*Byssochlamys spectabilis*	Eurotio	Asco	6	16	7	11	40	GCA_003990805.1	Kweichow moutai Co.,Ltd.
1255	*Neolentinus lepideus*	Agarico	Basidio	9	11	9	11	40	GCA_001632425.1	DOE Joint Genome Institute
1256	*Neocallimastix californiae*	Neocallimastigo	Chytridio	10	15	15	0	40	GCA_002104975.1	DOE Joint Genome Institute
1257	*Termitomyces heimii*	Agarico	Basidio	13	6	7	14	40	GCA_003313675.1	Kunming University of Science and Technology
1258	*Laetiporus sulphureus*	Agarico	Basidio	10	11	9	10	40	GCA_001632365.1	DOE Joint Genome Institute
1259	*Fibroporia radiculosa*	Agarico	Basidio	10	10	8	12	40	GCA_000313525.1	Forest Products, Mississippi State University
1260	*Phellinidium pouzarii*	Agarico	Basidio	12	7	6	15	40	GCA_004802695.1	IHI Zittau / TU Dresden
1261	*Exophiala aquamarina*	Eurotio	Asco	9	9	8	13	39	GCA_000709125.1	Broad Institute

1262	*Lepidopterella palustris*	Dothideo	Asco	9	12	7	11	39	GCA_001692735.1	DOE Joint Genome Institute
1263	*Raffaelea sulphurea*	Sordario	Asco	10	15	3	11	39	GCA_002778055.1	University of Montana
1264	*Exophiala sideris*	Eurotio	Asco	7	13	9	10	39	GCA_000835395.1	Broad Institute
1265	*Claviceps arundinis*	Sordario	Asco	8	13	9	9	39	GCA_004016465.1	Agriculture and Agri-Food Canada
1266	*Claviceps spartinae*	Sordario	Asco	8	13	9	9	39	GCA_004016175.1	Agriculture and Agri-Food Canada
1267	*Epichloe aotearoae*	Sordario	Asco	8	10	11	10	39	GCA_000729855.1	University of Kentucky
1268	*Claviceps humidiphila*	Sordario	Asco	8	13	9	9	39	GCA_004016155.1	Agriculture and Agri-Food Canada
1269	*Grosmannia galeiformis*	Sordario	Asco	10	13	4	12	39	GCA_004028395.1	University of Pretoria
1270	*Atkinsonella hypoxylon*	Sordario	Asco	7	13	10	9	39	GCA_000729835.1	University of Kentucky
1271	*Claviceps cf. purpurea*	Sordario	Asco	8	13	9	9	39	GCA_004016475.1	Agriculture and Agri-Food Canada
1272	*Moesziomyces sp. F16C1*	Ustilagino	Basidio	6	15	10	8	39	GCA_003004725.1	Universidade de Sao Paulo
1273	*Endoconidiophora polonica*	Sordario	Asco	10	15	3	11	39	GCA_001856765.1	Forestry and Agricultural Biotechnology Institute
1274	*Endoconidiophora laricicola*	Sordario	Asco	10	15	3	11	39	GCA_001640655.1	Forestry and Agricultural Biotechnology Institute
1275	*Ceratocystis adiposa*	Sordario	Asco	9	13	6	11	39	GCA_001640685.1	Forestry and Agricultural Biotechnology Institute
1276	*Gymnopilus junonius*	Agarico	Basidio	11	3	10	15	39	GCA_900068935.1	Royal Botanic Gardens, Kew
1277	*Ceratocystis smalleyi*	Sordario	Asco	9	14	6	10	39	GCA_003449175.1	Forestry and Agricultural Biotechnology Institute
1278	*Moniliophthora perniciosa*	Agarico	Basidio	9	8	8	14	39	GCA_000183025.1	Universidade Estadual de Campinas (UNICAMP), Brazil
1279	*Omphalotus olearius*	Agarico	Basidio	12	7	4	16	39	GCA_000296255.1	University of Minnesota
1280	*Lentinula edodes*	Agarico	Basidio	11	10	3	15	39	GCA_001562095.1	Chungbuk National University
1281	*Calocybe gambosa*	Agarico	Basidio	8	7	11	13	39	GCA_900068965.1	Royal Botanic Gardens, Kew
1282	*Umbelopsis isabellina*	Umbelopsido	Mucoro	6	18	7	7	38	GCA_000697415.1	IGS
1283	*Symbiotaphrina buchneri*	Xylono	Asco	7	15	6	10	38	GCA_001599915.1	RIKEN Center for Life Science Technologies, Division of Genomic Technologies
1284	*fungal sp. No.11243*	0	0	9	14	5	10	38	GCA_000836255.1	Tsukuba Biotechnology Research Center, Astellas Pharma Inc.,

1285	*Calocera cornea*	Dacry	Basidio	7	11	11	9	38	GCA_001632435.1	DOE Joint Genome Institute
1286	*Epichloe brachyelytri*	Sordario	Asco	8	10	10	10	38	GCA_000222915.1	University of Kentucky, Dept of Plant Pathology
1287	*Epichloe festucae*	Sordario	Asco	8	10	10	10	38	GCA_002318955.1	University of Kentucky
1288	*Epichloe baconii*	Sordario	Asco	8	10	10	10	38	GCA_000729845.1	University of Kentucky
1289	*Epichloe elymi*	Sordario	Asco	8	10	10	10	38	GCA_002591845.1	Massey University
1290	*Claviceps purpurea*	Sordario	Asco	8	13	8	9	38	GCA_004016095.1	Agriculture and Agri-Food Canada
1291	*Aciculosporium take*	Sordario	Asco	8	11	8	11	38	GCA_000222935.2	University of Kentucky, Dept of Plant Pathology
1292	*Rhinocladiella mackenziei*	Eurotio	Asco	8	10	9	11	38	GCA_000835555.1	Broad Institute
1293	*Postia placenta*	Agarico	Basidio	10	10	8	10	38	GCA_002117355.1	DOE Joint Genome Institute
1294	*Moesziomyces sp. F5C1*	Ustilagino	Basidio	6	14	10	8	38	GCA_003004715.1	Universidade de Sao Paulo
1295	*Huntiella omanensis*	Sordario	Asco	10	12	4	12	38	GCA_000833645.1	FABI
1296	*Megacollybia marginata*	Agarico	Basidio	10	7	7	14	38	GCA_003313965.1	Kunming University of Science and Technology
1297	*Sanghuangporus sanghuang*	Agarico	Basidio	12	6	6	14	38	GCA_009806525.1	Anhui Provincial Key Laboratory of Microbial Pest Control
1298	*Piromyces sp. E2*	Neocallimastigo	Chytridio	9	14	15	0	38	GCA_002157105.1	JGI
1299	*Infundibulicybe gibba*	Agarico	Basidio	12	8	4	14	38	GCA_900068925.1	Royal Botanic Gardens, Kew
1300	*Torrubiella hemipterigena*	Sordario	Asco	9	10	6	12	37	GCA_000825705.1	HKI JENA
1301	*Lecanicillium sp. MT-2017a*	Sordario	Asco	7	12	8	10	37	GCA_003056605.1	Northern Arizona University
1302	*Akanthomyces lecanii*	Sordario	Asco	9	10	6	12	37	GCA_001636795.1	Shanghai Institutes for Biological Sciences, CAS
1303	*Cladophialophora immunda*	Eurotio	Asco	6	9	12	10	37	GCA_000835495.1	Broad Institute
1304	*Epichloe sylvatica*	Sordario	Asco	8	10	9	10	37	GCA_001008265.1	University of Kentucky
1305	*Epichloe gansuensis*	Sordario	Asco	9	9	9	10	37	GCA_000222895.2	University of Kentucky, Dept of Plant Pathology
1306	*Exophiala dermatitidis*	Eurotio	Asco	8	8	10	11	37	GCA_010883545.1	Research Center for Medical Mycology - Peking University First Hospital
1307	*Sporothrix phasma*	Sordario	Asco	11	9	7	10	37	GCA_011037845.1	University of Pretoria
1308	*Epichloe typhina*	Sordario	Asco	8	10	9	10	37	GCA_000308955.1	University of Kentucky
1309	*Exophiala alcalophila*	Eurotio	Asco	8	7	10	12	37	GCA_001599775.1	RIKEN Center for Life Science Technologies, Division of Genomic Technologies

1310	*Epichloe sp. AL9924*	Sordario	Asco	8	10	9	10	37	GCA_000729825.1	University of Kentucky
1311	*Moesziomyces aphidis*	Ustilagino	Basidio	5	14	10	8	37	GCA_000517465.1	Fraunhofer IGB
1312	*Balansia obtecta*	Sordario	Asco	8	11	8	10	37	GCA_000709145.1	University of Kentucky
1313	*Fistulina hepatica*	Agarico	Basidio	10	9	4	14	37	GCA_000934395.1	DOE Joint Genome Institute
1314	*Huntiella savannae*	Sordario	Asco	9	13	4	11	37	GCA_001483325.1	Forestry and Agricultural Biotechnology Institute
1315	*Wolfiporia cocos*	Agarico	Basidio	9	9	8	11	37	GCA_000344635.1	DOE Joint Genome Institute
1316	*Huntiella decipiens*	Sordario	Asco	9	13	4	11	37	GCA_003032515.1	Forestry and Agricultural Biotechnology Institute
1317	*Huntiella moniliformis*	Sordario	Asco	9	13	4	11	37	GCA_000712465.1	Forestry and Agricultural Biotechnology Institute
1318	*Cryomyces minteri*	Dothideo	Asco	7	9	7	13	36	GCA_005059845.1	University of California, Riverside
1319	*Epichloe inebrians*	Sordario	Asco	9	8	9	10	36	GCA_000309355.1	University of Kentucky
1320	*Atkinsonella texensis*	Sordario	Asco	7	12	8	9	36	GCA_001008035.1	University of Kentucky
1321	*Apiotrichum porosum*	Tremello	Basidio	6	15	6	9	36	GCA_003942205.1	Karlsruhe Institute of Technology
1322	*Basidioascus undulatus*	Geminibasidio	Basidio	8	12	4	12	36	GCA_000826855.1	University of Ottawa
1323	*Epichloe amarillans*	Sordario	Asco	7	10	10	9	36	GCA_000223075.2	University of Kentucky, Dept of Plant Pathology
1324	*Tuber brumale*	Pezizo	Asco	10	12	3	11	36	GCA_014065205.1	INRAE
1325	*Tuber umbilicatum*	Pezizo	Asco	10	12	2	12	36	GCA_003313605.1	Kunming University of Science and Technology
1326	*Moesziomyces antarcticus*	Ustilagino	Basidio	6	12	10	8	36	GCA_900322835.1	Technische Universitat Munchen - WZW
1327	*Ambrosiella xylebori*	Sordario	Asco	9	11	5	11	36	GCA_002778035.1	University of Montana
1328	*Huntiella bhutanensis*	Sordario	Asco	9	12	4	11	36	GCA_002018275.1	Forestry and Agricultural Biotechnology Institute
1329	*Verruconis sp.*	Dothideo	Asco	9	11	5	11	36	GCA_004026245.1	Jill Banfield's Lab at Berkeley
1330	*Calocera viscosa*	Dacry	Basidio	7	10	9	9	35	GCA_001630345.1	DOE Joint Genome Institute
1331	*Tuber magnatum*	Pezizo	Asco	10	10	4	11	35	GCA_003182015.1	INRA
1332	*Sporisorium reilianum*	Ustilagino	Basidio	7	11	9	8	35	GCA_900162835.1	Technische Universitat Munchen - WZW
1333	*Agaricus bisporus*	Agarico	Basidio	11	2	7	15	35	GCA_008271545.1	Jilin Agricultural Unversity
1334	*Pterula multifida*	Agarico	Basidio	11	6	4	14	35	GCA_900068985.1	Royal Botanic Gardens, Kew
1335	*Tolypocladium sp. Sup5 PDA-1*	Sordario	Asco	9	9	6	10	34	GCA_000750105.3	OMRF
1336	*Ustilaginoidea virens*	Sordario	Asco	8	10	5	11	34	GCA_000965225.2	Computational Biology Research Center (CBRC),

										National Institute of Advanced Industrial Science and Technology (AIST)
1337	*Fonsecaea multimorphosa*	Eurotio	Asco	6	8	11	9	34	GCA_000836435.1	Broad Institute
1338	*Exophiala phaeomuriformis*	Eurotio	Asco	7	8	9	10	34	GCA_010883475.1	Research Center for Medical Mycology - Peking University First Hospital
1339	*Exophiala spinifera*	Eurotio	Asco	8	6	9	11	34	GCA_010882955.1	Research Center for Medical Mycology - Peking University First Hospital
1340	*Eurotiomycetes sp. MA 6038*	Eurotio	Asco	8	9	5	12	34	GCA_003004525.2	University of Natural Resources and Life Sciences, Vienna
1341	*Pseudozyma hubeiensis*	Ustilagino	Basidio	6	12	8	8	34	GCA_000403515.1	Kitami Institute of Technology
1342	*Pseudozyma tsukubaensis*	Ustilagino	Basidio	5	14	7	8	34	GCA_001736125.1	RWTH Aachen University
1343	*Tuber indicum*	Pezizo	Asco	10	11	3	10	34	GCA_006112555.1	College of Pharmacy and Biological Engineering
1344	*Dacryopinax primogenitus*	Dacry	Basidio	7	8	10	9	34	GCA_000292625.1	DOE Joint Genome Institute
1345	*Tuber melanosporum*	Pezizo	Asco	10	10	3	11	34	GCA_000151645.1	The French-Italian Tuber Genome Consortium
1346	*Boletus edulis*	Agarico	Basidio	11	5	5	13	34	GCA_003316165.1	Kunming University of Science and Technology
1347	*Drechslerella stenobrocha*	Orbilio	Asco	10	8	7	9	34	GCA_000525045.1	Institute of Microbiology, Chinese Academy of Sciences
1348	*Lecanicillium fungicola*	Sordario	Asco	8	9	5	11	33	GCA_900169235.1	UNIVERSITY OF BRISTOL
1349	*Metarhizium robertsii*	Sordario	Asco	10	5	6	12	33	GCA_000187425.2	Metarhizium genome sequencing Consortium
1350	*Microcyclospora tardicrescens*	Dothideo	Asco	7	10	8	8	33	GCA_003012245.1	USDA, ARS, NCAUR
1351	*Coniosporium apollinis*	Dothideo	Asco	8	8	4	13	33	GCA_000281105.1	Broad Institute
1352	*Ramichloridium luteum*	Dothideo	Asco	6	7	8	12	33	GCA_002368545.1	Northwest A&F University
1353	*Tuber microsphaerosporum*	Pezizo	Asco	9	12	2	10	33	GCA_003521225.1	Kunming University of Science and Technology
1354	*Fonsecaea pugnacius*	Eurotio	Asco	6	7	10	10	33	GCA_011800825.1	FEDERAL UNIVERSITY OF PARANA
1355	*Apiotrichum laibachii*	Tremello	Basidio	6	11	7	9	33	GCA_001600735.1	RIKEN Center for Life Science Technologies,

										Division of Genomic Technologies
1356	*Apiotrichum veenhuisii*	Tremello	Basidio	7	12	6	8	33	GCA_001600595.1	RIKEN Center for Life Science Technologies, Division of Genomic Technologies
1357	*Fonsecaea erecta*	Eurotio	Asco	6	8	10	9	33	GCA_001651985.1	Federal University of Parana
1358	*Eurotiomycetes sp. MA 6039*	Eurotio	Asco	8	10	4	11	33	GCA_003004485.1	University of Natural Resources and Life Sciences, Vienna
1359	*Pseudozyma sp. F8B2*	Ustilagino	Basidio	5	12	8	8	33	GCA_003004685.1	Universidade de Sao Paulo
1360	*Papiliotrema flavescens*	Tremello	Basidio	7	10	6	10	33	GCA_000442785.1	OSU/OARDC
1361	*Ustilago cynodontis*	Ustilagino	Basidio	6	11	7	9	33	GCA_001736195.1	RWTH Aachen University
1362	*Sparassis latifolia*	Agarico	Basidio	10	5	6	12	33	GCA_009812315.1	Chosun University
1363	*Chiua virens*	Agarico	Basidio	11	6	5	11	33	GCA_003316485.1	Kunming University of Science and Technology
1364	*Dioszegia aurantiaca*	Tremello	Basidio	6	13	7	7	33	GCA_001600655.1	RIKEN Center for Life Science Technologies, Division of Genomic Technologies
1365	*Coprinus comatus*	Agarico	Basidio	11	3	6	13	33	GCA_003316025.1	Kunming University of Science and Technology
1366	*Paecilomyces hepiali*	Sordario	Asco	7	8	6	11	32	GCA_001455915.2	Beijing Normal University
1367	*Metarhizium guizhouense*	Sordario	Asco	9	5	6	12	32	GCA_000814955.1	Shanghai Insititutes for Biological Sciences, CAS
1368	*Cladophialophora psammophila*	Eurotio	Asco	6	7	9	10	32	GCA_000585535.1	Broad Institute
1369	*Pascua guehoae*	Tremello	Basidio	7	10	7	8	32	GCA_001600415.1	RIKEN Center for Life Science Technologies, Division of Genomic Technologies
1370	*Cryomyces antarcticus*	Dothideo	Asco	7	7	5	13	32	GCA_000504465.1	VIBT Extremophile Center, University of Natural Resources and Life Sciences Vienna, Austria
1371	*Cutaneotrichosporon mucoides*	Tremello	Basidio	5	11	8	8	32	GCA_003116955.1	RIKEN Center for Life Science Technologies, Division of Genomic Technologies
1372	*Apiotrichum gamsii*	Tremello	Basidio	6	12	7	7	32	GCA_001600315.1	RIKEN Center for Life Science Technologies,

1373	*Anthracocystis flocculosa*	Ustilagino	Basidio	6	14	5	7	32	GCA_900322975.1	Division of Genomic Technologies Technische Universitat Munchen - WZW
1374	*Sporisorium graminicola*	Ustilagino	Basidio	4	13	8	7	32	GCA_005498985.1	University of Liverpool, Institute of Integrative Biology
1375	*Kalmanozyma brasiliensis*	Ustilagino	Basidio	5	11	7	9	32	GCA_000497045.1	Laboratorio Nacional de Ciencia e Tecnologia do Bioetanol
1376	*Ustilago tritici*	Ustilagino	Basidio	6	11	7	8	32	GCA_002993085.1	University of Sao Paulo
1377	*Sparassis crispa*	Agarico	Basidio	9	6	6	11	32	GCA_003851025.1	Department of Pediatric Cardiology, Tokyo Women's Medical University
1378	*Ustilago xerochloae*	Ustilagino	Basidio	5	10	8	9	32	GCA_001736075.1	RWTH Aachen University
1379	*Ascodesmis nigricans*	Pezizo	Asco	11	6	4	11	32	GCA_004786065.1	DOE Joint Genome Institute
1380	*Suillus pictus*	Agarico	Basidio	10	3	6	13	32	GCA_003313085.1	Kunming University of Science and Technology
1381	*Megacollybia platyphylla*	Agarico	Basidio	10	4	7	11	32	GCA_900068915.1	Royal Botanic Gardens, Kew
1382	*Bifiguratus adelaidae*	Endogono	Mucoro	7	11	6	7	31	GCA_002261195.1	University of California, Riverside
1383	*Metarhizium anisopliae*	Sordario	Asco	9	4	6	12	31	GCA_013305495.1	Chonbuk National University
1384	*Cordyceps farinosa*	Sordario	Asco	7	7	6	11	31	GCA_000733625.1	CSIR- Institute of Microbial technology (IMTECH)
1385	*Metarhizium majus*	Sordario	Asco	9	5	5	12	31	GCA_000814945.1	Shanghai Insititutes for Biological Sciences, CAS
1386	*Metarhizium brunneum*	Sordario	Asco	9	4	6	12	31	GCA_000814965.1	Shanghai Insititutes for Biological Sciences, CAS
1387	*Beauveria rudraprayagi*	Sordario	Asco	9	5	4	13	31	GCA_000733645.1	CSIR- Institute of Microbial technology (IMTECH)
1388	*Cladophialophora bantiana*	Eurotio	Asco	6	6	9	10	31	GCA_000835475.1	Broad Institute
1389	*Apiotrichum akiyoshidainum*	Tremello	Basidio	7	10	6	8	31	GCA_002973495.1	PROIMI - CONICET
1390	*Ceratocystiopsis minuta*	Sordario	Asco	7	11	3	10	31	GCA_001676865.1	University of Pretoria
1391	*Polychaeton citri*	Dothideo	Asco	6	10	5	10	31	GCA_010093785.1	DOE Joint Genome Institute
1392	*Monascus purpureus*	Eurotio	Asco	7	10	4	10	31	GCA_011319195.1	Beijing Technology and Business University

1393	*Tuber borchii*	Pezizo	Asco	9	10	2	10	31	GCA_003070745.1	DOE Joint Genome Institute
1394	*Capronia coronata*	Eurotio	Asco	8	5	6	12	31	GCA_000585585.1	Broad Institute
1395	*Cutaneotrichosporon daszewskae*	Tremello	Basidio	5	10	8	8	31	GCA_002335585.1	RIKEN Center for Life Science Technologies, Division of Genomic Technologies
1396	*Sporisorium iseilematis-ciliati*	Ustilagino	Basidio	4	11	8	8	31	GCA_001748505.1	RWTH Aachen University
1397	*Violaceomyces palustris*	Ustilagino	Basidio	6	11	4	10	31	GCA_003144235.1	DOE Joint Genome Institute
1398	*Kockovaella imperatae*	Tremello	Basidio	6	10	8	7	31	GCA_002102565.1	DOE Joint Genome Institute
1399	*Takashimella koratensis*	Tremello	Basidio	5	12	8	6	31	GCA_003116875.1	RIKEN Center for Life Science Technologies, Division of Genomic Technologies
1400	*Serpula lacrymans*	Agarico	Basidio	10	6	5	10	31	GCA_002891385.1	University of Oslo
1401	*Gonapodya prolifera*	Monoblepharido	Chytridio	6	15	1	9	31	GCA_001574975.1	DOE Joint Genome Institute
1402	*Xerocomus impolitus*	Agarico	Basidio	10	5	4	12	31	GCA_003316335.1	Kunming University of Science and Technology
1403	*Powellomyces hirtus*	Chytridio	Chytridio	10	10	1	10	31	GCA_006536005.1	Agriculture and Agri-Food Canada
1404	*Piromyces finnis*	Neocallimastigo	Chytridio	9	12	10	0	31	GCA_002104945.1	DOE Joint Genome Institute
1405	*Cordyceps javanica*	Sordario	Asco	7	7	5	11	30	GCA_006981985.1	The Institute of Vegetables and Flowers CAAS
1406	*Beauveria sp. IMV 00265*	Sordario	Asco	9	7	3	11	30	GCA_001931865.2	Jet Propulsion Laboratory, California Institute of Technology
1407	*Beauveria pseudobassiana*	Sordario	Asco	9	6	3	12	30	GCA_003267905.1	National Institute of Horticultural and Herbal Science, Rural Development Administration
1408	*Tolypocladium sp. Salcha MEA-2*	Sordario	Asco	7	7	6	10	30	GCA_000750145.2	INPART
1409	*Tolypocladium inflatum*	Sordario	Asco	7	7	6	10	30	GCA_003945705.1	University of Minnesota
1410	*Beauveria brongniartii*	Sordario	Asco	9	7	3	11	30	GCA_001636735.1	Shanghai Institutes for Biological Sciences, CAS
1411	*Acaromyces ingoldii*	Exobasidio	Basidio	4	13	4	9	30	GCA_003144295.1	DOE Joint Genome Institute
1412	*Leucosporidium scottii*	Microbotryo	Basidio	6	10	4	10	30	GCA_003054985.1	UCIBIO-REQUIMTE, Faculdade de Ciencias e

										Tecnologia, Universidade Nova da Lisboa
1413	*Albatrellus ellisii*	Agarico	Basidio	10	5	4	11	30	GCA_003314395.1	Kunming University of Science and Technology
1414	*Ustilago bromivora*	Ustilagino	Basidio	4	10	8	8	30	GCA_900101485.1	Institute of Bioinformatics and Systems Biology
1415	*Amanita thiersii*	Agarico	Basidio	9	5	4	12	30	GCA_002554575.1	JGI
1416	*Leucoagaricus gongylophorus*	Agarico	Basidio	9	2	7	12	30	GCA_000382605.1	UW-Madison
1417	*Cordyceps pruinosa*	Sordario	Asco	7	7	4	11	29	GCA_003025255.1	National Institute of Horticultural and Herbal Science, Rural Development Administration
1418	*Paecilomyces penicillatus*	Sordario	Asco	9	8	4	8	29	GCA_005765155.1	Biotechnology and Nuclear Technology Research Institute
1419	*Beauveria bassiana*	Sordario	Asco	9	5	3	12	29	GCA_003337105.1	Biotechnology Institute-National University of Colombia- Bogota
1420	*Fonsecaea pedrosoi*	Eurotio	Asco	5	6	10	8	29	GCA_000835455.1	Broad Institute
1421	*Apiotrichum mycotoxinovorans*	Tremello	Basidio	7	8	6	8	29	GCA_013177335.1	Nanjing TECH University
1422	*Escovopsis sp. TC*	Sordario	Asco	8	6	7	8	29	GCA_003055185.1	University of East Anglia
1423	*Herpotrichiellaceae sp. UM238*	Eurotio	Asco	9	6	1	13	29	GCA_000315175.1	UNIVERSITY OF MALAYA
1424	*Fonsecaea nubica*	Eurotio	Asco	5	7	9	8	29	GCA_001646965.1	Federal University of Parana
1425	*Cutaneotrichosporon dermatis*	Tremello	Basidio	5	10	7	7	29	GCA_003116895.1	RIKEN Center for Life Science Technologies, Division of Genomic Technologies
1426	*Exophiala mesophila*	Eurotio	Asco	9	4	5	11	29	GCA_000836275.1	Broad Institute
1427	*Peltaster fructicola*	Dothideo	Asco	4	11	6	8	29	GCA_001592805.2	Northwest A&F University
1428	*Gyalolechia flavorubescens*	Lecanoro	Asco	8	6	5	10	29	GCA_000442125.1	Seoul National University
1429	*Dioszegia crocea*	Tremello	Basidio	5	10	9	5	29	GCA_001600615.1	RIKEN Center for Life Science Technologies, Division of Genomic Technologies
1430	*Arthrocladium fulminans*	Eurotio	Asco	8	8	4	9	29	GCA_003614865.1	Westerdijk Fungal Biodiversity Institute
1431	*Terfezia boudieri*	Pezizo	Asco	8	9	3	9	29	GCA_003788615.2	DOE Joint Genome Institute
1432	*Paxillus involutus*	Agarico	Basidio	8	5	4	12	29	GCA_000827475.1	JGI

1433	*Solicoccozyma phenolica*	Tremello	Basidio	5	11	6	7	29	GCA_001600015.1	RIKEN Center for Life Science Technologies, Division of Genomic Technologies
1434	*Boletus sp. MG55*	Agarico	Basidio	9	5	4	11	29	GCA_003313885.1	Kunming University of Science and Technology
1435	*Retiboletus ornatipes*	Agarico	Basidio	8	3	6	12	29	GCA_003316065.1	Kunming University of Science and Technology
1436	*Tolypocladium paradoxum*	Sordario	Asco	8	5	5	10	28	GCA_002916505.1	Oregon State University
1437	*Fonsecaea monophora*	Eurotio	Asco	5	6	9	8	28	GCA_001642475.1	Federal University of Parana
1438	*Monascus ruber*	Eurotio	Asco	6	10	3	9	28	GCA_002976275.1	Fujian Institute of Microbiology
1439	*Bullera alba*	Tremello	Basidio	4	10	8	6	28	GCA_001600095.1	RIKEN Center for Life Science Technologies, Division of Genomic Technologies
1440	*Sporisorium scitamineum*	Ustilagino	Basidio	4	11	6	7	28	GCA_000772675.1	Key Lab of Sugarcane Biology and Genetic Breeding, Ministry of Agriculture, P.R.China
1441	*Kwoniella heveanensis*	Tremello	Basidio	6	9	5	8	28	GCA_000507405.3	Broad Institute
1442	*Albatrellus sp. MG142*	Agarico	Basidio	8	2	5	13	28	GCA_003314695.1	Kunming University of Science and Technology
1443	*Suillus alpinus*	Agarico	Basidio	8	4	5	11	28	GCA_003316505.1	Kunming University of Science and Technology
1444	*Suillellus subvelutipes*	Agarico	Basidio	9	2	3	14	28	GCA_003316035.1	Kunming University of Science and Technology
1445	*Ustilago hordei*	Ustilagino	Basidio	4	9	8	7	28	GCA_003012045.1	Jawaharlal Nehru Centre for Advanced Scientific Research
1446	*Neoboletus brunneissimus*	Agarico	Basidio	9	3	4	12	28	GCA_003316195.1	Kunming University of Science and Technology
1447	*Hypomyces rosellus*	Sordario	Asco	7	4	8	8	27	GCA_011799845.1	Jilin Agricultural University
1448	*Metarhizium acridum*	Sordario	Asco	7	5	3	12	27	GCA_000187405.1	Metarhizium genome sequencing Consortium
1449	*Tolypocladium ophioglossoides*	Sordario	Asco	7	6	5	9	27	GCA_001189435.1	Oregon State University
1450	*Microcyclospora pomicola*	Dothideo	Asco	6	6	8	7	27	GCA_002786065.1	Northwest A&F University
1451	*Usnea hakonensis*	Lecanoro	Asco	9	5	1	12	27	GCA_013423325.1	Swedish Museum of Natural History
1452	*Gongronella sp. w5*	Mucoro	Mucoro	4	10	5	8	27	GCA_001650995.1	Anhui University
1453	*Cladonia uncialis*	Lecanoro	Asco	8	4	3	12	27	GCA_002927785.1	University of Manitoba

1454	*Tuber calosporum*	Pezizo	Asco	8	8	1	10	27	GCA_003316355.1	Kunming University of Science and Technology
1455	*Ustilago trichophora*	Ustilagino	Basidio	4	11	4	8	27	GCA_900323505.1	Technische Universitat Munchen - WZW
1456	*Taiwanofungus camphoratus*	Agarico	Basidio	9	3	6	9	27	GCA_000766995.1	Academia Sinica
1457	*Naganishia sp. IF7SW-B1*	Tremello	Basidio	5	9	6	7	27	GCA_012922685.1	Jet Propulsion Laboratory, California Institute of Technology
1458	*Naganishia adeliensis*	Tremello	Basidio	5	9	6	7	27	GCA_012922715.1	Jet Propulsion Laboratory, California Institute of Technology
1459	*Tylopilus plumbeoviolaceoides*	Agarico	Basidio	8	2	5	12	27	GCA_003316375.1	Kunming University of Science and Technology
1460	*Rhizopogon vesiculosus*	Agarico	Basidio	8	3	5	11	27	GCA_001882365.1	University of Florida
1461	*Auricularia polytricha*	Agarico	Basidio	8	2	5	12	27	GCA_003316125.1	Kunming University of Science and Technology
1462	*Papiliotrema laurentii*	Tremello	Basidio	5	9	6	7	27	GCA_012922615.1	Jet Propulsion Laboratory, California Institute of Technology
1463	*Rhizopogon sp. AM-2018a*	Agarico	Basidio	8	4	5	10	27	GCA_002995095.1	Oregon State University
1464	*Suillus sp. MG131*	Agarico	Basidio	8	5	5	9	27	GCA_003313855.1	Kunming University of Science and Technology
1465	*Butyriboletus roseoflavus*	Agarico	Basidio	8	3	4	12	27	GCA_003315995.1	Kunming University of Science and Technology
1466	*Boletus speciosus*	Agarico	Basidio	8	3	5	11	27	GCA_003316055.1	Kunming University of Science and Technology
1467	*Floccularia luteovirens*	Agarico	Basidio	9	3	5	10	27	GCA_009739215.1	Zhejiang University
1468	*Hypomyces perniciosus*	Sordario	Asco	7	5	6	8	26	GCA_008477525.1	Jilin Agricultural University
1469	*Albophoma yamanashiensis*	Sordario	Asco	7	6	3	10	26	GCA_001600555.1	RIKEN Center for Life Science Technologies, Division of Genomic Technologies
1470	*Cordyceps cicadae*	Sordario	Asco	6	5	5	10	26	GCA_010211705.1	Anhui Agricultural University
1471	*Metarhizium rileyi*	Sordario	Asco	7	5	3	11	26	GCA_007866325.1	Embrapa
1472	*Metarhizium album*	Sordario	Asco	7	5	4	10	26	GCA_000804445.1	Shanghai Institutes for Biological Sciences, CAS
1473	*Hirsutella thompsonii*	Sordario	Asco	7	3	3	13	26	GCA_000472145.1	Institute of Microbial Technology
1474	*Hirsutella minnesotensis*	Sordario	Asco	8	3	3	12	26	GCA_000956045.1	Institute of Microbiology, Chinese Academy of Sciences
1475	*Ceratocystiopsis brevicomis*	Sordario	Asco	5	9	2	10	26	GCA_002778105.1	University of Montana

1476	*Capronia epimyces*	Eurotio	Asco	7	4	4	11	26	GCA_000585565.1	Broad Institute
1477	*Thermomyces lanuginosus*	Eurotio	Asco	8	3	3	12	26	GCA_000315935.1	Durban University of Technology
1478	*Ascosphaera apis*	Eurotio	Asco	2	16	2	6	26	GCA_001636715.1	Shanghai Institutes for Biological Sciences, CAS
1479	*Ustilago maydis*	Ustilagino	Basidio	5	6	6	9	26	GCA_001599495.1	RIKEN Center for Life Science Technologies, Division of Genomic Technologies
1480	*Ustilago vetiveriae*	Ustilagino	Basidio	4	9	7	6	26	GCA_001735935.1	RWTH Aachen University
1481	*Cryptococcus amylolentus*	Tremello	Basidio	4	10	5	7	26	GCA_001720205.1	Broad Institute
1482	*Cryptococcus floricola*	Tremello	Basidio	4	10	5	7	26	GCA_006352305.1	Duke University
1483	*Cryptococcus wingfieldii*	Tremello	Basidio	4	10	5	7	26	GCA_001720155.1	Broad Institute
1484	*Kwoniella shandongensis*	Tremello	Basidio	5	9	4	8	26	GCA_008629635.1	Broad Institute
1485	*Rhizopogon vinicolor*	Agarico	Basidio	8	2	5	11	26	GCA_001658105.1	DOE Joint Genome Institute
1486	*Boletus sp. MG95*	Agarico	Basidio	7	5	3	11	26	GCA_003313155.1	Kunming University of Science and Technology
1487	*Rhizopogon hawkerae*	Agarico	Basidio	8	4	4	10	26	GCA_002995035.1	Oregon State University
1488	*Kwoniella bestiolae*	Tremello	Basidio	5	9	4	8	26	GCA_000512585.2	Broad Institute
1489	*Tricholoma matsutake*	Agarico	Basidio	11	2	2	11	26	GCA_002939025.2	Korea University
1490	*Cladobotryum protrusum*	Sordario	Asco	7	3	7	8	25	GCA_004303015.1	Jilin Agricultural University
1491	*Cordyceps tenuipes*	Sordario	Asco	6	5	4	10	25	GCA_003025305.1	National Institute of Horticultural and Herbal Science, Rural Development Administration
1492	*Hirsutella rhossiliensis*	Sordario	Asco	6	4	4	11	25	GCA_004142005.1	Chinese Academy of Sciences
1493	*Cordyceps sp. RAO-2017*	Sordario	Asco	8	3	3	11	25	GCA_002591385.1	Utrecht University
1494	*Trichosporon ovoides*	Tremello	Basidio	6	4	5	10	25	GCA_009833065.1	Heilongjiang bayi agricultural university
1495	*Escovopsis weberi*	Sordario	Asco	7	4	6	8	25	GCA_003055145.1	University of East Anglia
1496	*Rusavskia elegans*	Lecanoro	Asco	8	5	2	10	25	GCA_011316305.1	Yunnan Academy of Forestry
1497	*Evernia prunastri*	Lecanoro	Asco	8	4	2	11	25	GCA_003184365.1	Senckenberg
1498	*Prillingera fragicola*	Tremello	Basidio	5	6	6	8	25	GCA_002335605.1	RIKEN Center for Life Science Technologies, Division of Genomic Technologies
1499	*Letharia columbiana*	Lecanoro	Asco	8	4	3	10	25	GCA_014066305.1	Eastern Washington University
1500	*Emmonsia crescens*	Eurotio	Asco	6	5	4	10	25	GCA_002572855.1	Broad Institute

1501	*Naganishia liquefaciens*	Tremello	Basidio	5	9	4	7	25	GCA_013423385.1	School of Life Science and Technology, Tokyo Institute of Technology
1502	*Boletus bicolor*	Agarico	Basidio	8	2	5	10	25	GCA_003316205.1	Kunming University of Science and Technology
1503	*Rhizopogon parksii*	Agarico	Basidio	8	4	3	10	25	GCA_002994865.1	Oregon State University
1504	*Zoophagus insidians*	Zoopago	Zoopago	1	17	2	5	25	GCA_004114325.1	University of Michigan
1505	*Rhizopogon rudus*	Agarico	Basidio	8	4	3	10	25	GCA_002995055.1	Oregon State University
1506	*Caloboletus calopus*	Agarico	Basidio	9	2	3	11	25	GCA_003316085.1	Kunming University of Science and Technology
1507	*Ramaria sp. MG151*	Agarico	Basidio	9	2	2	12	25	GCA_003314545.1	Kunming University of Science and Technology
1508	*Cordyceps fumosorosea*	Sordario	Asco	6	4	4	10	24	GCA_001636725.1	Shanghai Institutes for Biological Sciences, CAS
1509	*Cordyceps militaris*	Sordario	Asco	7	4	3	10	24	GCA_003332165.1	pangugene
1510	*Trichosporon coremiiforme*	Tremello	Basidio	6	4	3	11	24	GCA_001752605.1	RIKEN Center for Life Science Technologies, Division of Genomic Technologies
1511	*Dirinaria sp. GBRC AP01*	Lecanoro	Asco	8	3	2	11	24	GCA_013315955.1	Gujarat Biotechnology Research Centre
1512	*Leptoxyphium fumago*	Dothideo	Asco	6	5	4	9	24	GCA_001660795.1	International Institute Zittau - TU Dresden
1513	*Pseudevernia furfuracea*	Lecanoro	Asco	7	3	3	11	24	GCA_003184345.1	Senckenberg
1514	*Cutaneotrichosporon cutaneum*	Tremello	Basidio	5	5	6	8	24	GCA_001600715.1	RIKEN Center for Life Science Technologies, Division of Genomic Technologies
1515	*Ophiocordyceps australis*	Sordario	Asco	7	4	3	10	24	GCA_002591415.1	Utrecht University
1516	*Thecaphora thlaspeos*	Ustilagino	Basidio	6	10	1	7	24	GCA_900260175.1	RWTH Aachen University
1517	*Cladonia metacorallifera*	Lecanoro	Asco	7	5	3	9	24	GCA_000482085.2	Seoul National University
1518	*Moniliella sp. 'wahieum'*	Moniliello	Basidio	4	9	0	11	24	GCA_003971905.1	Kyungpook National University
1519	*Suillus luteus*	Agarico	Basidio	7	3	5	9	24	GCA_000827255.1	DOE Joint Genome Institute
1520	*Tricholoma saponaceum*	Agarico	Basidio	9	2	2	11	24	GCA_003313625.1	Kunming University of Science and Technology
1521	*Rhizopogon salebrosus*	Agarico	Basidio	9	2	2	11	24	GCA_002995475.1	Oregon State University
1522	*Naematelia encephala*	Tremello	Basidio	4	8	5	7	24	GCA_002105065.1	DOE Joint Genome Institute
1523	*Entoloma clypeatum*	Agarico	Basidio	9	4	1	10	24	GCA_900068945.1	Royal Botanic Gardens, Kew
1524	*Tricholoma terreum*	Agarico	Basidio	8	3	2	11	24	GCA_003316345.1	Kunming University of Science and Technology

1525	*Escovopsis sp. AC*	Sordario	Asco	7	4	4	8	23	GCA_003055955.1	University of East Anglia
1526	*Blastomyces emzantsi*	Eurotio	Asco	6	4	4	9	23	GCA_003206725.1	National Institute for Communicable Diseases
1527	*Trichosporon asahii*	Tremello	Basidio	6	4	3	10	23	GCA_001972365.1	RIKEN Center for Life Science Technologies, Division of Genomic Technologies
1528	*Apiotrichum gracile*	Tremello	Basidio	7	4	4	8	23	GCA_001600335.1	RIKEN Center for Life Science Technologies, Division of Genomic Technologies
1529	*Tolypocladium capitatum*	Sordario	Asco	6	4	4	9	23	GCA_002901185.1	Oregon State University
1530	*Trichosporon faecale*	Tremello	Basidio	6	4	3	10	23	GCA_001752585.1	RIKEN Center for Life Science Technologies, Division of Genomic Technologies
1531	*Endocarpon pusillum*	Eurotio	Asco	7	3	1	12	23	GCA_000611755.1	Seoul National University
1532	*Elaphomyces granulatus*	Eurotio	Asco	6	4	3	10	23	GCA_002240705.1	Oregon State University
1533	*Letharia lupina*	Lecanoro	Asco	8	3	2	10	23	GCA_014066315.1	Eastern Washington University
1534	*Jimgerdemannia lactiflua*	Endogono	Mucoro	6	6	2	9	23	GCA_003951145.1	Oregon State University
1535	*Cladonia rangiferina*	Lecanoro	Asco	7	4	2	10	23	GCA_006146055.1	Lake Superior State University
1536	*Chaetothyriales sp. CBS 134920*	Eurotio	Asco	8	3	2	10	23	GCA_003693665.1	Westerdijk Fungal Biodiversity Institute
1537	*Cutaneotrichosporon oleaginosum*	Tremello	Basidio	5	5	5	8	23	GCA_008065305.1	Ruhr-University Bochum
1538	*Ramalina peruviana*	Lecanoro	Asco	7	4	2	10	23	GCA_001956345.1	Yunnan Academy of Forestry
1539	*Lichtheimia ramosa*	Mucoro	Mucoro	4	9	3	7	23	GCA_000945115.1	HKI JENA
1540	*Cutaneotrichosporon arboriformis*	Tremello	Basidio	5	4	4	10	23	GCA_002335565.1	RIKEN Center for Life Science Technologies, Division of Genomic Technologies
1541	*Naganishia randhawae*	Tremello	Basidio	5	7	3	8	23	GCA_013461525.1	Wits University
1542	*Rhizopogon vulgaris*	Agarico	Basidio	8	1	3	11	23	GCA_002995295.1	Oregon State University
1543	*Hebeloma cylindrosporum*	Agarico	Basidio	8	3	2	10	23	GCA_000827355.1	DOE Joint Genome Institute
1544	*Solicoccozyma terricola*	Tremello	Basidio	5	8	3	7	23	GCA_001600875.1	RIKEN Center for Life Science Technologies, Division of Genomic Technologies
1545	*Suillus brevipes*	Agarico	Basidio	7	3	4	9	23	GCA_011800875.1	DOE Joint Genome Institute

1546	*Synchytrium microbalum*	Chytridio	Chytridio	8	4	1	10	23	GCA_006535985.1	Agriculture and Agri-Food Canada
1547	*Rhizopogon villosulus*	Agarico	Basidio	7	2	4	10	23	GCA_002995315.1	Oregon State University
1548	*Escovopsis sp. Ae720*	Sordario	Asco	7	3	4	8	22	GCA_003055925.1	University of East Anglia
1549	*Escovopsis sp. Ae733*	Sordario	Asco	7	3	4	8	22	GCA_003055945.1	University of East Anglia
1550	*Escovopsis sp. Ae724*	Sordario	Asco	7	3	4	8	22	GCA_003055165.1	University of East Anglia
1551	*Moelleriella libera*	Sordario	Asco	5	4	4	9	22	GCA_001636675.1	Shanghai Institutes for Biological Sciences, CAS
1552	*Ophiocordyceps sinensis*	Sordario	Asco	6	2	2	12	22	GCA_002077885.1	Institute of Microbiology, Chinese Academy of Sciences
1553	*Neohortaea acidophila*	Dothideo	Asco	5	7	3	7	22	GCA_010093505.1	DOE Joint Genome Institute
1554	*Umbilicaria muehlenbergii*	Lecanoro	Asco	8	2	2	10	22	GCA_000611775.1	Seoul National University
1555	*Tremella fuciformis*	Tremello	Basidio	5	3	7	7	22	GCA_000987905.1	Fujian Agriculture and Forestry University
1556	*Leucosporidium creatinivorum*	Microbotryo	Basidio	5	7	2	8	22	GCA_001600635.1	RIKEN Center for Life Science Technologies, Division of Genomic Technologies
1557	*Lasallia pustulata*	Lecanoro	Asco	8	2	2	10	22	GCA_008636195.1	University of Frankfurt
1558	*Syncephalastrum sp. IA-2019*	Mucoro	Mucoro	4	9	2	7	22	GCA_013461545.1	University of Melbourne
1559	*Mrakia frigida*	Tremello	Basidio	4	9	3	6	22	GCA_001600395.1	RIKEN Center for Life Science Technologies, Division of Genomic Technologies
1560	*Emergomyces orientalis*	Eurotio	Asco	6	4	2	10	22	GCA_002110485.1	Beijing Institute of Radiation Medicine
1561	*Nilaparvata lugens yeast-like symbiont*	0	Asco	6	3	3	10	22	GCA_000758425.1	Nilaparvata lugens Genome Consortium
1562	*Ustilago esculenta*	Ustilagino	Basidio	5	6	3	8	22	GCA_000819925.1	Zhejiang Provincial Key Laboratory of Biometrology and Inspection & Quarantine
1563	*Lasallia hispanica*	Lecanoro	Asco	8	2	2	10	22	GCA_003254425.1	Senckenberg
1564	*Blastomyces percursus*	Eurotio	Asco	6	5	2	9	22	GCA_001883805.1	Broad Institute
1565	*Naganishia albida*	Tremello	Basidio	5	6	4	7	22	GCA_001599735.1	RIKEN Center for Life Science Technologies, Division of Genomic Technologies
1566	*Russula aff. compacta*	Agarico	Basidio	7	0	2	13	22	GCA_003313875.1	Kunming University of Science and Technology
1567	*Filobasidium wieringae*	Tremello	Basidio	5	6	5	6	22	GCA_001600055.1	RIKEN Center for Life Science Technologies,

										Division of Genomic Technologies
1568	*Ramaria cf. rubripermanens*	Agarico	Basidio	9	0	2	11	22	GCA_003316465.1	Kunming University of Science and Technology
1569	*Hygrophorus pudorinus*	Agarico	Basidio	6	4	1	11	22	GCA_003314045.1	Kunming University of Science and Technology
1570	*Trichosporon inkin*	Tremello	Basidio	6	4	2	9	21	GCA_001752625.1	RIKEN Center for Life Science Technologies, Division of Genomic Technologies
1571	*Dissoconium aciculare*	Dothideo	Asco	5	3	5	8	21	GCA_010015565.1	DOE Joint Genome Institute
1572	*Drechmeria coniospora*	Sordario	Asco	6	5	1	9	21	GCA_001625195.1	Biotechnology Research Institute, Chinese Academy of Agricultural Sciences
1573	*Ramalina intermedia*	Lecanoro	Asco	6	4	2	9	21	GCA_003073195.1	Yunnan Academy of Forestry
1574	*Syncephalastrum monosporum*	Mucoro	Mucoro	4	9	2	6	21	GCA_000697355.1	IGS
1575	*Chaetothyriales sp. CBS 135597*	Eurotio	Asco	7	3	1	10	21	GCA_003709825.1	Westerdijk Fungal Biodiversity Institute
1576	*Cladonia macilenta*	Lecanoro	Asco	5	5	3	8	21	GCA_000444155.1	Seoul National University
1577	*Cerataphis brasiliensis yeast-like symbiont*	0	Asco	6	3	2	10	21	GCA_000372705.1	University of Arizona
1578	*Rhodotorula paludigena*	Microbotryo	Basidio	5	3	2	11	21	GCA_005281665.1	Suranaree University of Technology
1579	*Emergomyces pasteurianus*	Eurotio	Asco	7	2	2	10	21	GCA_001883825.1	Broad Institute
1580	*Paxillus rubicundulus*	Agarico	Basidio	6	3	4	8	21	GCA_000827395.1	DOE Joint Genome Institute
1581	*Blastomyces silverae*	Eurotio	Asco	6	4	2	9	21	GCA_001014755.1	Broad Institute
1582	*Phlebopus portentosus*	Agarico	Basidio	7	2	1	11	21	GCA_000766925.2	Yunnan University
1583	*Blastomyces gilchristii*	Eurotio	Asco	7	3	2	9	21	GCA_000003855.2	Broad Institute
1584	*Neoboletus magnificus*	Agarico	Basidio	7	1	2	11	21	GCA_003316145.1	Kunming University of Science and Technology
1585	*Melampsora pinitorqua*	Puccinio	Basidio	5	10	3	3	21	GCA_000464645.1	Tree Aggressors Identification using Genomic Approaches
1586	*Sarcodon aspratus*	Agarico	Basidio	6	2	4	9	21	GCA_003313825.1	Kunming University of Science and Technology
1587	*Kwoniella dejecticola*	Tremello	Basidio	4	7	3	7	21	GCA_000512565.2	Broad Institute
1588	*Hypocrella siamensis*	Sordario	Asco	5	3	3	9	20	GCA_000731825.1	CSIR- Institute of Microbial technology (IMTECH)
1589	*Quambalaria eucalypti*	Exobasidio	Basidio	3	8	1	8	20	GCA_004016185.1	University of Pretoria

1590	*Jimgerdemannia flammicorona*	Endogono	Mucoro	4	4	3	9	20	GCA_003990745.1	DOE Joint Genome Institute
1591	*Lichtheimia corymbifera*	Mucoro	Mucoro	3	8	2	7	20	GCA_000697175.1	IGS
1592	*Syncephalastrum racemosum*	Mucoro	Mucoro	4	9	1	6	20	GCA_002105135.1	DOE Joint Genome Institute
1593	*Hygrocybe conica*	Agarico	Basidio	6	5	2	7	20	GCA_900068975.1	Royal Botanic Gardens, Kew
1594	*Jaminaea rosea*	Exobasidio	Basidio	3	8	1	8	20	GCA_003144245.1	DOE Joint Genome Institute
1595	*Blastomyces parvus*	Eurotio	Asco	6	4	2	8	20	GCA_002572885.1	Broad Institute
1596	*Antarctomyces pellizariae*	Leotio	Asco	8	4	0	8	20	GCA_010623925.1	UFMG - Universidade Federal de Minas Gerais
1597	*Blastomyces dermatitidis*	Eurotio	Asco	6	3	2	9	20	GCA_000003525.2	Broad Institute
1598	*Pseudomicrostroma glucosiphilum*	Exobasidio	Basidio	5	3	4	8	20	GCA_003144135.1	DOE Joint Genome Institute
1599	*Rhizopus oryzae*	Mucoro	Mucoro	6	6	0	8	20	GCA_000697195.1	IGS
1600	*Lactarius trivialis*	Agarico	Basidio	7	0	1	12	20	GCA_003315845.1	Kunming University of Science and Technology
1601	*Russula griseocarnosa*	Agarico	Basidio	6	0	1	13	20	GCA_004801975.1	Research Institute of Tropical Forestry, Chinese Academy of Forestry
1602	*Holtermanniella nyarrowii*	Tremello	Basidio	4	8	3	5	20	GCA_001600035.1	RIKEN Center for Life Science Technologies, Division of Genomic Technologies
1603	*Tricholoma sp. MG77*	Agarico	Basidio	7	2	0	11	20	GCA_003314665.1	Kunming University of Science and Technology
1604	*Lactifluus pinguis*	Agarico	Basidio	7	0	0	13	20	GCA_003313945.1	Kunming University of Science and Technology
1605	*Lactifluus rugatus*	Agarico	Basidio	6	1	1	12	20	GCA_003315895.1	Kunming University of Science and Technology
1606	*Lactifluus hygrophoroides*	Agarico	Basidio	6	0	1	13	20	GCA_003314055.1	Kunming University of Science and Technology
1607	*Gomphus bonarii*	Agarico	Basidio	8	0	1	11	20	GCA_003316585.1	Kunming University of Science and Technology
1608	*Erysiphe pulchra*	Leotio	Asco	4	7	3	6	20	GCA_002918395.1	USDA-ARS
1609	*Takashimella tepidaria*	Tremello	Basidio	4	6	3	7	20	GCA_003116915.1	RIKEN Center for Life Science Technologies, Division of Genomic Technologies
1610	*Gomphus sp. MG54*	Agarico	Basidio	8	0	0	12	20	GCA_003314385.1	Kunming University of Science and Technology
1611	*Pyrrhoderma noxium*	Agarico	Basidio	7	3	1	9	20	GCA_002287475.2	Academia Sinica

1612	*Ophiocordyceps polyrhachis-furcata*	Sordario	Asco	4	4	5	6	19	GCA_001633055.2	National Center for Genetic Engineering and Biotechnology (BIOTEC)
1613	*Oph. camponoti-leonardi (nom. inval.)*	Sordario	Asco	5	4	3	7	19	GCA_003339455.1	National Center for Genetic Engineering and Biotechnology
1614	*Alectoria sarmentosa*	Lecanoro	Asco	6	3	2	8	19	GCA_009733775.1	Universidad Complutense de Madrid
1615	*Rhizoclosmatium globosum*	Chytridio	Chytridio	6	5	0	8	19	GCA_002104985.1	DOE Joint Genome Institute
1616	*Cutaneotrichosporon curvatum*	Tremello	Basidio	5	3	4	7	19	GCA_001600275.1	RIKEN Center for Life Science Technologies, Division of Genomic Technologies
1617	*Cutaneotrichosporon cyanovorans*	Tremello	Basidio	5	2	4	8	19	GCA_002335625.1	RIKEN Center for Life Science Technologies, Division of Genomic Technologies
1618	*Rhizomucor pusillus*	Mucoro	Mucoro	4	8	1	6	19	GCA_900175165.2	CHALMERS UNIVERSITY OF TECHNOLOGY
1619	*Rhodotorula graminis*	Microbotryo	Basidio	4	4	3	8	19	GCA_001329695.1	DOE Joint Genome Institute
1620	*Suillus placidus*	Agarico	Basidio	6	2	3	8	19	GCA_003313645.1	Kunming University of Science and Technology
1621	*Rhizopus delemar*	Mucoro	Mucoro	6	5	0	8	19	GCA_000149305.1	Broad Institute
1622	*Phycomyces blakesleeanus*	Mucoro	Mucoro	5	5	1	8	19	GCA_001638985.2	JGI-PSF
1623	*Lactarius echinatus*	Agarico	Basidio	6	1	1	11	19	GCA_003315975.1	Kunming University of Science and Technology
1624	*Laccaria amethystina*	Agarico	Basidio	7	1	1	10	19	GCA_000827195.1	DOE Joint Genome Institute
1625	*Apiotrichum montevideense*	Tremello	Basidio	6	2	3	7	18	GCA_001598995.1	RIKEN Center for Life Science Technologies, Division of Genomic Technologies
1626	*Apiotrichum domesticum*	Tremello	Basidio	5	2	3	8	18	GCA_001599015.1	RIKEN Center for Life Science Technologies, Division of Genomic Technologies
1627	*Rhizomucor miehei*	Mucoro	Mucoro	3	7	2	6	18	GCA_000611695.1	Department of Biotechnology, College of Food Science and Nutritional Engineering, China Agricultural University

1628	*Lactarius indigo*	Agarico	Basidio	5	1	1	11	18	GCA_003313985.1	Kunming University of Science and Technology
1629	*Ceraceosorus guamensis*	Exobasidio	Basidio	3	5	1	9	18	GCA_003144195.1	DOE Joint Genome Institute
1630	*Cystobasidiopsis lactophilus*	Agaricostilbo	Basidio	7	1	1	9	18	GCA_001599975.1	RIKEN Center for Life Science Technologies, Division of Genomic Technologies
1631	*Chytriomyces confervae*	Chytridio	Chytridio	3	6	2	7	18	GCA_006535975.1	Agriculture and Agri-Food Canada
1632	*Symmetrospora coprosmae*	Cystobasidio	Basidio	4	4	2	8	18	GCA_008802785.1	University College Dublin
1633	*Lactarius hatsudake*	Agarico	Basidio	5	1	1	11	18	GCA_003315955.1	Kunming University of Science and Technology
1634	*Erythrobasidium yunnanense*	Cystobasidio	Basidio	4	3	3	8	18	GCA_001600175.1	RIKEN Center for Life Science Technologies, Division of Genomic Technologies
1635	*Cetradonia linearis*	Lecanoro	Asco	5	2	1	10	18	GCA_003521265.1	The New York Botanical Garden
1636	*Protomyces macrosporus*	Taphrino	Asco	4	7	2	5	18	GCA_003717175.1	University of Helsinki
1637	*Lactarius sp. MG121*	Agarico	Basidio	5	1	1	11	18	GCA_003315925.1	Kunming University of Science and Technology
1638	*Thelephora aurantiotincta*	Agarico	Basidio	6	0	2	10	18	GCA_003316405.1	Kunming University of Science and Technology
1639	*Pulveroboletus ravenelii*	Agarico	Basidio	5	2	2	9	18	GCA_003316545.1	Kunming University of Science and Technology
1640	*Laccaria bicolor*	Agarico	Basidio	6	1	0	11	18	GCA_000143565.1	Laccaria Genome Consortium
1641	*Russula virescens*	Agarico	Basidio	5	0	1	12	18	GCA_003316435.1	Kunming University of Science and Technology
1642	*Microbotryum silenes-acaulis*	Microbotryo	Basidio	4	4	1	9	18	GCA_003665825.1	Universite Paris Sud
1643	*Microbotryum silenes-dioicae*	Microbotryo	Basidio	5	3	1	9	18	GCA_900120095.1	ESE
1644	*Phaeotremella skinneri*	Tremello	Basidio	5	4	2	7	18	GCA_001599695.1	RIKEN Center for Life Science Technologies, Division of Genomic Technologies
1645	*Tricholoma bakamatsutake*	Agarico	Basidio	8	0	1	9	18	GCA_003313665.1	Kunming University of Science and Technology
1646	*Chroogomphus rutilus*	Agarico	Basidio	6	0	2	10	18	GCA_003314275.1	Kunming University of Science and Technology
1647	*Ophiocordyceps camponoti-rufipedis*	Sordario	Asco	3	4	3	7	17	GCA_002591395.1	Utrecht University
1648	*Ophiocordyceps camponoti-floridani*	Sordario	Asco	3	4	3	7	17	GCA_012980515.1	University of Central Florida

1649	*Ophiocordyceps unilateralis*	Sordario	Asco	4	3	3	7	17	GCA_001272575.2	Utrecht University
1650	*Chaetothyriales sp. CBS 134916*	Eurotio	Asco	6	0	2	9	17	GCA_003709845.1	Westerdijk Fungal Biodiversity Institute
1651	*Tilletiopsis washingtonensis*	Exobasidio	Basidio	2	6	2	7	17	GCA_003144115.1	DOE Joint Genome Institute
1652	*Apiotrichum brassicae*	Tremello	Basidio	4	2	4	7	17	GCA_001600295.1	RIKEN Center for Life Science Technologies, Division of Genomic Technologies
1653	*Podosphaera leucotricha*	Leotio	Asco	4	3	2	8	17	GCA_013170925.1	Washington State University
1654	*Salmacisia buchloeana*	Exobasidio	Basidio	7	2	0	8	17	GCA_001990185.1	Pennsylvania State University
1655	*Thermomucor indicae-seudaticae*	Mucoro	Mucoro	3	5	3	6	17	GCA_000787465.1	Aalborg University
1656	*Mucor racemosus*	Mucoro	Mucoro	4	3	1	9	17	GCA_000697255.1	IGS
1657	*Tilletiaria anomala*	Exobasidio	Basidio	4	4	1	8	17	GCA_000711695.1	DOE Joint Genome Institute
1658	*Paracoccidioides lutzii*	Eurotio	Asco	4	2	3	8	17	GCA_000150705.2	Broad Institute
1659	*Rhizopus stolonifer*	Mucoro	Mucoro	5	3	1	8	17	GCA_000697035.1	IGS
1660	*Russula foetens*	Agarico	Basidio	5	0	1	11	17	GCA_003316565.1	Kunming University of Science and Technology
1661	*Rhizopus azygosporus*	Mucoro	Mucoro	6	2	0	9	17	GCA_003325435.1	University of California, Riverside
1662	*Rhizopus microsporus*	Mucoro	Mucoro	6	2	0	9	17	GCA_006680115.1	TGen-North
1663	*Choanephora cucurbitarum*	Mucoro	Mucoro	4	4	0	9	17	GCA_001683725.1	Korea University
1664	*Rhizopogon fuscorubens*	Agarico	Basidio	6	1	2	8	17	GCA_002995455.1	Oregon State University
1665	*Microbotryum intermedium*	Microbotryo	Basidio	4	3	1	9	17	GCA_900096595.1	ESE
1666	*Tricholoma flavovirens*	Agarico	Basidio	5	0	2	10	17	GCA_003313805.1	Kunming University of Science and Technology
1667	*Tricholoma sp. MG99*	Agarico	Basidio	8	0	1	8	17	GCA_003521275.1	Kunming University of Science and Technology
1668	*Wallemia hederae*	Wallemio	Basidio	7	2	0	8	17	GCA_004918325.1	Biotechnical Faculty, University of Ljubljana
1669	*Phellinus lamaoensis*	Agarico	Basidio	6	1	1	9	17	GCA_002794735.1	Academia Sinica
1670	*Chrysosporium queenslandicum*	Eurotio	Asco	3	1	2	10	16	GCA_001430955.1	UC Berkeley
1671	*Ophiocordyceps camponoti-saundersi (nom. inval.)*	Sordario	Asco	4	3	3	6	16	GCA_003339415.1	National Center for Genetic Engineering and Biotechnology
1672	*Vanrija humicola*	Tremello	Basidio	4	3	2	7	16	GCA_008065275.1	Ruhr-University Bochum
1673	*Basidiobolus meristosporus*	Basidiobolo	Zoopago	5	2	0	9	16	GCA_002104905.1	DOE Joint Genome Institute
1674	*Chaetothyriales sp. CBS 132003*	Eurotio	Asco	6	0	1	9	16	GCA_003709865.1	Westerdijk Fungal Biodiversity Institute

1675	*Aspergillus cejpii*	Eurotio	Asco	5	3	0	8	16	GCA_004769165.1	Guangdong Institute of Microbiology
1676	*Lactarius sp. MG50*	Agarico	Basidio	5	0	1	10	16	GCA_003314065.1	Kunming University of Science and Technology
1677	*Tilletia caries*	Exobasidio	Basidio	3	4	1	8	16	GCA_001645005.2	Agriculture and Agri-Food Canada
1678	*Xeromyces bisporus*	Eurotio	Asco	5	3	1	7	16	GCA_900006255.1	UPPSALA UNIVERSITET
1679	*Rhodotorula diobovata*	Microbotryo	Basidio	4	2	2	8	16	GCA_006352295.1	University of Manitoba
1680	*Mucor velutinosus*	Mucoro	Mucoro	4	1	2	9	16	GCA_000696895.1	IGS
1681	*Mrakia psychrophila*	Tremello	Basidio	3	4	4	5	16	GCA_001889225.1	Institute Of Microbiology Chinese Academy of Sciences
1682	*Histoplasma capsulatum*	Eurotio	Asco	4	3	2	7	16	GCA_000313325.1	Taipei Medical University
1683	*Rhodotorula toruloides*	Microbotryo	Basidio	4	1	2	9	16	GCA_001255795.1	UCB
1684	*Cystobasidiaceae sp. HBUAS51001*	Cystobasidio	Basidio	5	1	3	7	16	GCA_003351005.1	Hubei University of Arts and Science
1685	*Protomyces lactucaedebilis*	Taphrino	Asco	5	5	1	5	16	GCA_002105105.1	DOE Joint Genome Institute
1686	*Caulochytrium protostelioides*	Chytridio	Chytridio	5	1	1	9	16	GCA_003615045.1	DOE Joint Genome Institute
1687	*Piloderma croceum*	Agarico	Basidio	7	2	0	7	16	GCA_000827315.1	DOE Joint Genome Institute
1688	*Endogone sp. FLAS-F59071*	Endogono	Mucoro	4	3	1	8	16	GCA_003990785.1	DOE Joint Genome Institute
1689	*Protomyces inouyei*	Taphrino	Asco	5	5	1	5	16	GCA_003717155.1	University of Helsinki
1690	*Lactarius deliciosus*	Agarico	Basidio	5	0	0	11	16	GCA_006680135.1	UCSI University
1691	*Microbotryum saponariae*	Microbotryo	Basidio	5	2	1	8	16	GCA_900102585.1	ESE
1692	*Cryptococcus neoformans*	Tremello	Basidio	3	4	2	7	16	GCA_002216725.1	Washington University in St. Louis
1693	*Lactarius volemus*	Agarico	Basidio	4	0	1	11	16	GCA_003315835.1	Kunming University of Science and Technology
1694	*Russula abietina*	Agarico	Basidio	4	0	1	11	16	GCA_003313715.1	Kunming University of Science and Technology
1695	*Russula lepida*	Agarico	Basidio	5	0	1	10	16	GCA_003316425.1	Kunming University of Science and Technology
1696	*Lactarius piperatus*	Agarico	Basidio	5	0	0	11	16	GCA_003315875.1	Kunming University of Science and Technology
1697	*Scleroderma citrinum*	Agarico	Basidio	4	1	3	8	16	GCA_000827425.1	DOE Joint Genome Institute
1698	*Hygrophorus russula*	Agarico	Basidio	4	1	2	9	16	GCA_003314125.1	Kunming University of Science and Technology
1699	*Mortierella alpina*	Mortierello	Mucoro	3	3	2	7	15	GCA_000507065.1	IGS
1700	*Tilletia controversa*	Exobasidio	Basidio	3	3	1	8	15	GCA_001645045.2	Agriculture and Agri-Food Canada

1701	*Ceraceosorus bombacis*	Exobasidio	Basidio	3	3	1	8	15	GCA_900000165.1	IPF
1702	*Mucor ambiguus*	Mucoro	Mucoro	5	1	0	9	15	GCA_000950595.1	National Institute of Advanced Industrial Science and Technology (AIST)
1703	*Emmonsia sp. CAC-2015a*	Eurotio	Asco	3	3	2	7	15	GCA_001660665.1	Broad Institute
1704	*Tilletia walkeri*	Exobasidio	Basidio	3	3	1	8	15	GCA_009428295.1	Agriculture and Agri-Food Canada
1705	*Xylona heveae*	Xylono	Asco	5	1	0	9	15	GCA_001619985.1	DOE Joint Genome Institute
1706	*Paracoccidioides brasiliensis*	Eurotio	Asco	3	2	3	7	15	GCA_000150475.2	Broad Institute
1707	*Mucor irregularis*	Mucoro	Mucoro	4	1	1	9	15	GCA_000587855.1	Nanjing Biozeron
1708	*Actinomucor elegans*	Mucoro	Mucoro	5	1	0	9	15	GCA_001599635.1	RIKEN Center for Life Science Technologies, Division of Genomic Technologies
1709	*Mrakia blollopis*	Tremello	Basidio	4	2	4	5	15	GCA_000950635.1	National Institute of Advanced Industrial Science and Technology (AIST)
1710	*Erythrobasidium hasegawianum*	Cystobasidio	Basidio	4	2	2	7	15	GCA_001972285.1	RIKEN Center for Life Science Technologies, Division of Genomic Technologies
1711	*Parasitella parasitica*	Mucoro	Mucoro	4	1	1	9	15	GCA_000938895.1	FRIEDRICH SCHILLER UNIVERSITY JENA
1712	*Protomyces pachydermus*	Taphrino	Asco	5	5	0	5	15	GCA_003717275.1	University of Helsinki
1713	*Saitoella complicata*	0	Asco	5	2	1	7	15	GCA_000227095.3	Saitoella complicata genome sequencing consortium
1714	*Protomyces sp. C29*	Taphrino	Asco	5	5	0	5	15	GCA_003568695.1	University of Helsinki
1715	*Rhodotorula sp. ZM1*	Microbotryo	Basidio	4	0	2	9	15	GCA_009806315.1	Zhejiang Normal University
1716	*Golovinomyces cichoracearum*	Leotio	Asco	4	3	2	6	15	GCA_003611215.1	University of Maryland
1717	*Synchytrium endobioticum*	Chytridio	Chytridio	5	3	0	7	15	GCA_006536045.1	Agriculture and Agri-Food Canada
1718	*Cryptococcus gattii VGII*	Tremello	Basidio	3	3	1	8	15	GCA_003011995.1	Jawaharlal Nehru Centre for Advanced Scientific Research
1719	*Amanita brunnescens*	Agarico	Basidio	4	3	1	7	15	GCA_001691785.2	Harvard University
1720	*Amanita muscaria*	Agarico	Basidio	5	1	0	9	15	GCA_000827485.1	DOE Joint Genome Institute
1721	*Amauroascus mutatus*	Eurotio	Asco	3	2	1	8	14	GCA_001430935.1	UC Berkeley

1722	*Tilletia indica*	Exobasidio	Basidio	2	2	2	8	14	GCA_001689995.1	ICAR-Indian Institute of Wheat and Barley Research, Karnal
1723	*Mortierella verticillata*	Mortierello	Mucoro	3	3	0	8	14	GCA_000739165.1	Broad Institute
1724	*Mucor indicus*	Mucoro	Mucoro	4	1	0	9	14	GCA_000697295.1	IGS
1725	*Mucor lusitanicus*	Mucoro	Mucoro	4	1	0	9	14	GCA_010203745.1	DOE Joint Genome Institute
1726	*Mucor circinelloides*	Mucoro	Mucoro	4	1	0	9	14	GCA_001599575.1	RIKEN Center for Life Science Technologies, Division of Genomic Technologies
1727	*Basidiobolus heterosporus*	Basidiobolo	Zoopago	5	0	1	8	14	GCA_000697455.1	IGS
1728	*Taphrina betulina*	Taphrino	Asco	3	3	2	6	14	GCA_008802775.1	University College Dublin
1729	*Taphrina confusa*	Taphrino	Asco	3	4	1	6	14	GCA_005281535.1	Northwest A&F University
1730	*Protomyces gravidus*	Taphrino	Asco	4	4	0	6	14	GCA_003717255.1	University of Helsinki
1731	*Glaciozyma antarctica*	Microbotryo	Basidio	4	1	1	8	14	GCA_002917775.1	Malaysia Genome Institute
1732	*Microbotryum violaceum*	Microbotryo	Basidio	4	2	1	7	14	GCA_900015485.1	INRA-LIPM
1733	*Cryptococcus neoformans AD hybrid*	Tremello	Basidio	4	2	0	8	14	GCA_006992865.1	Weill Cornell Medicine
1734	*Sarcodon sp. MG97*	Agarico	Basidio	4	2	1	7	14	GCA_003313065.1	Kunming University of Science and Technology
1735	*Amanita phalloides*	Agarico	Basidio	5	0	0	9	14	GCA_001983385.1	Michigan State University
1736	*Kwoniella mangrovensis*	Tremello	Basidio	3	3	2	6	14	GCA_000507485.3	Broad Institute
1737	*Amauroascus niger*	Eurotio	Asco	2	2	1	8	13	GCA_001430945.1	UC Berkeley
1738	*Mortierella elongata*	Mortierello	Mucoro	2	3	1	7	13	GCA_001651415.1	DOE Joint Genome Institute
1739	*fungal sp. Mo6-1*	0	0	3	2	2	6	13	GCA_002939055.1	University of Montana
1740	*Nannizzia gypsea*	Eurotio	Asco	4	1	0	8	13	GCA_000150975.2	Broad Institute
1741	*Holtermannia corniformis*	Tremello	Basidio	4	2	1	6	13	GCA_001599935.1	RIKEN Center for Life Science Technologies, Division of Genomic Technologies
1742	*Taphrina pruni*	Taphrino	Asco	2	5	1	5	13	GCA_005281585.1	Northwest A&F University
1743	*Blumeria graminis*	Leotio	Asco	3	2	1	7	13	GCA_900237765.1	MAX PLANCK INSTITUTE FOR PLANT BREEDING RESEARCH
1744	*Taphrina flavorubra*	Taphrino	Asco	2	5	1	5	13	GCA_000836175.1	University of Miyazaki
1745	*Saksenaea vasiformis*	Mucoro	Mucoro	5	0	0	8	13	GCA_000697055.1	IGS
1746	*Pisolithus tinctorius*	Agarico	Basidio	4	0	1	8	13	GCA_000827335.1	DOE Joint Genome Institute

1747	*Pisolithus microcarpus*	Agarico	Basidio	3	2	1	7	13	GCA_000827275.1	JGI
1748	*Blyttiomyces helicus*	Chytridio	Chytridio	4	2	0	7	13	GCA_003614705.1	DOE Joint Genome Institute
1749	*Rhizophagus clarus*	Glomero	Mucoro	3	1	0	9	13	GCA_003203555.1	National Institute for Basic Biology
1750	*Arthroderma uncinatum*	Eurotio	Asco	3	1	1	7	12	GCA_011692745.1	Chinese Academy of Medical Science and Peking Union Medical College
1751	*Absidia glauca*	Mucoro	Mucoro	3	1	1	7	12	GCA_900079185.1	FRIEDRICH SCHILLER UNIVERSITY JENA
1752	*Piedraia hortae*	Dothideo	Asco	2	2	4	4	12	GCA_010093745.1	DOE Joint Genome Institute
1753	*Cunninghamella bertholletiae*	Mucoro	Mucoro	3	2	0	7	12	GCA_000697215.1	IGS
1754	*Cunninghamella elegans*	Mucoro	Mucoro	3	2	0	7	12	GCA_000697015.1	IGS
1755	*Taphrina communis*	Taphrino	Asco	2	4	1	5	12	GCA_005281525.1	Northwest A&F University
1756	*Cokeromyces recurvatus*	Mucoro	Mucoro	4	0	0	8	12	GCA_000697235.1	IGS
1757	*Gigaspora rosea*	Glomero	Mucoro	3	2	0	7	12	GCA_003550325.1	INRA
1758	*Allomyces macrogynus*	Blastocladio	Blastocladio	1	4	3	4	12	GCA_000151295.1	Broad Institute
1759	*Gigaspora margarita*	Glomero	Mucoro	4	2	0	6	12	GCA_009809945.1	CNR
1760	*Taphrina sp. SM11*	Taphrino	Asco	3	3	2	4	12	GCA_004000165.1	University of Helsinki
1761	*Cryptococcus gattii VGI*	Tremello	Basidio	3	1	1	7	12	GCA_000836355.1	Broad Institute
1762	*Cryptococcus gattii VGIV*	Tremello	Basidio	3	1	1	7	12	GCA_000835755.1	Broad Institute
1763	*Oidium heveae*	Leotio	Asco	3	2	0	7	12	GCA_003957845.1	Hainan University
1764	*Golubevia sp. BC0902*	Exobasidio	Basidio	3	2	0	7	12	GCA_012976205.1	Wageningen UR
1765	*Amanita inopinata*	Agarico	Basidio	4	1	0	7	12	GCA_001691775.3	Harvard University
1766	*Amanita pseudoporphyria*	Agarico	Basidio	5	0	0	7	12	GCA_003316615.1	Kunming University of Science and Technology
1767	*Microsporum canis*	Eurotio	Asco	2	2	1	6	11	GCA_000151145.1	Broad Institute
1768	*Absidia repens*	Mucoro	Mucoro	2	1	0	8	11	GCA_002105175.1	DOE Joint Genome Institute
1769	*Mortierella sp. BCC40632*	Mortierello	Mucoro	3	1	0	7	11	GCA_011634665.1	National Center for Genetic Engineering and Biotechnology
1770	*Hesseltinella vesiculosa*	Mucoro	Mucoro	3	0	0	8	11	GCA_002104935.1	DOE Joint Genome Institute
1771	*Uncinocarpus reesii*	Eurotio	Asco	3	1	1	6	11	GCA_000003515.2	Broad Institute
1772	*Saksenaea oblongispora*	Mucoro	Mucoro	4	0	0	7	11	GCA_000697495.1	IGS
1773	*Rhodotorula sp. JG-1b*	Microbotryo	Basidio	2	2	1	6	11	GCA_001541205.1	DOE Joint Genome Institute
1774	*Taphrina deformans*	Taphrino	Asco	3	2	1	5	11	GCA_005281805.1	Northwest A&F University
1775	*Conidiobolus incongruus*	Entomophthoro	Zoopago	2	1	1	7	11	GCA_000697335.1	IGS

1776	*Glomus cerebriforme*	Glomero	Mucoro	2	1	0	8	11	GCA_003833025.1	University of Ottawa
1777	*Cryptococcus cf. gattii*	Tremello	Basidio	2	2	1	6	11	GCA_009650685.1	Broad Institute
1778	*Cryptococcus gattii VGIII*	Tremello	Basidio	2	2	1	6	11	GCA_000836335.1	Broad Institute
1779	*Golubevia sp. BC0812*	Exobasidio	Basidio	3	2	0	6	11	GCA_012976215.1	Wageningen UR
1780	*Golubevia sp. BC0850*	Exobasidio	Basidio	3	2	0	6	11	GCA_012976225.1	Wageningen UR
1781	*Oidium neolycopersici*	Leotio	Asco	4	1	0	6	11	GCA_003610855.1	University of Maryland
1782	*Oehlia diaphana*	Glomero	Mucoro	2	1	0	8	11	GCA_003833135.1	University of Ottawa
1783	*Rhizophagus irregularis*	Glomero	Mucoro	2	1	0	8	11	GCA_002897155.1	NIBB core research facilities, National Institues for Basic Biology
1784	*Rhizophagus sp. MUCL 43196*	Glomero	Mucoro	2	1	0	8	11	GCA_003549995.1	INRA
1785	*Amanita polypyramis*	Agarico	Basidio	3	0	0	8	11	GCA_001691755.2	Harvard University
1786	*Trichophyton mentagrophytes*	Eurotio	Asco	2	2	0	6	10	GCA_003664465.1	North-Western State Medical University named after I.I. Mechnicov
1787	*Byssoonygena ceratinophila*	Eurotio	Asco	3	1	0	6	10	GCA_001430925.1	UC Berkeley
1788	*Trichophyton benhamiae*	Eurotio	Asco	2	2	0	6	10	GCA_001858085.1	Swiss Institute of Bioinformatics
1789	*Rhodotorula taiwanensis*	Microbotryo	Basidio	2	1	1	6	10	GCA_002922495.1	Lawrence Livermore National Laboratory
1790	*Coccidioides immitis*	Eurotio	Asco	2	1	1	6	10	GCA_004115165.2	TGen North
1791	*Apophysomyces trapeziformis*	Mucoro	Mucoro	4	0	0	6	10	GCA_000696975.1	IGS
1792	*Apophysomyces elegans*	Mucoro	Mucoro	4	0	0	6	10	GCA_000696995.1	IGS
1793	*Rhodotorula mucilaginosa*	Microbotryo	Basidio	2	2	0	6	10	GCA_003055205.1	Indian Institute Of Chemical biology
1794	*Taphrina wiesneri*	Taphrino	Asco	2	3	0	5	10	GCA_005281515.1	Northwest A&F University
1795	*Linderina pennispora*	Kickxello	Zoopago	1	3	1	5	10	GCA_002104995.1	DOE Joint Genome Institute
1796	*Malassezia furfur*	Malassezio	Basidio	2	1	0	7	10	GCA_002551515.1	University of Utrecht
1797	*Wallemia ichthyophaga*	Wallemio	Basidio	4	1	0	5	10	GCA_004918895.1	Biotechnical Faculty, University of Ljubljana
1798	*Diversispora epigaea*	Glomero	Mucoro	2	1	0	7	10	GCA_003547095.1	Cornell University
1799	*Neolecta irregularis*	Neolecto	Asco	2	2	1	5	10	GCA_001929475.1	University of California, Riverside
1800	*Malassezia obtusa*	Malassezio	Basidio	2	1	0	7	10	GCA_001264985.1	Genome Institute of Singapore
1801	*Kwoniella pini*	Tremello	Basidio	2	2	2	4	10	GCA_000512605.2	Broad Institute
1802	*Golubevia pallescens*	Exobasidio	Basidio	3	2	0	5	10	GCA_001599655.1	RIKEN Center for Life Science Technologies, Division of Genomic Technologies

1803	*Malassezia globosa*	Malassezio	Basidio	3	0	0	7	10	GCA_001264805.1	Genome Institute of Singapore
1804	*Trichophyton rubrum*	Eurotio	Asco	2	1	0	6	9	GCA_000616845.1	Broad Institute
1805	*Trichophyton soudanense*	Eurotio	Asco	2	1	0	6	9	GCA_000616865.1	Broad Institute
1806	*Trichophyton interdigitale*	Eurotio	Asco	2	1	0	6	9	GCA_000622975.1	Broad Institute
1807	*Trichophyton kuryangei*	Eurotio	Asco	2	1	0	6	9	GCA_012184535.1	Belgian Coordinated Collection of Microorganisms, BCCM
1808	*Trichophyton equinum*	Eurotio	Asco	2	1	0	6	9	GCA_000151175.1	Broad Institute
1809	*Trichophyton tonsurans*	Eurotio	Asco	2	1	0	6	9	GCA_000151455.1	Broad Institute
1810	*Trichophyton violaceum*	Eurotio	Asco	2	1	0	6	9	GCA_001651435.1	Chinese Academy of Medical Science & Peking Union Medical College
1811	*Trichophyton yaoundei*	Eurotio	Asco	2	1	0	6	9	GCA_012184575.1	Belgian Coordinated Collection of Microorganisms, BCCM
1812	*Trichophyton verrucosum*	Eurotio	Asco	2	1	0	6	9	GCA_000151505.1	Arthroderma Genome Sequencing Consortium
1813	*Onygena corvina*	Eurotio	Asco	2	1	0	6	9	GCA_000812245.1	Aalborg University
1814	*Rhodotorula sp. CCFEE 5036*	Microbotryo	Basidio	2	1	0	6	9	GCA_005059875.1	University of California, Riverside
1815	*Apophysomyces variabilis*	Mucoro	Mucoro	3	0	0	6	9	GCA_002749535.1	Postgraduate Institute of medical education and research
1816	*Ophidiomyces ophiodiicola*	Eurotio	Asco	3	0	0	6	9	GCA_002167195.1	University of Arizona
1817	*Taphrina populina*	Taphrino	Asco	2	3	0	4	9	GCA_000836195.1	University of Miyazaki
1818	*Lobosporangium transversale*	Mortierello	Mucoro	2	1	0	6	9	GCA_002105155.1	DOE Joint Genome Institute
1819	*fungal sp. ARF18*	0	0	2	1	0	6	9	GCA_002224055.1	University of Arkansas
1820	*Entomophthora muscae*	Entomophthoro	Zoopago	2	0	0	7	9	GCA_900018355.1	UNIVERSITY OF COPENHAGEN
1821	*Conidiobolus coronatus*	Entomophthoro	Zoopago	2	1	0	6	9	GCA_001566745.1	JGI-PSF
1822	*Puccinia triticina*	Puccinio	Basidio	4	0	0	5	9	GCA_013090125.1	University of Sydney
1823	*Malassezia cuniculi*	Malassezio	Basidio	3	0	0	6	9	GCA_001264635.1	Genome Institute of Singapore
1824	*Erysiphe necator*	Leotio	Asco	3	0	0	6	9	GCA_000798715.1	University of California, Davis
1825	*Massospora cicadina*	Entomophthoro	Zoopago	2	0	0	7	9	GCA_006912075.1	University of Michigan
1826	*Cystobasidium pallidum*	Cystobasidio	Basidio	2	0	2	5	9	GCA_001599955.1	RIKEN Center for Life Science Technologies, Division of Genomic Technologies
1827	*Erysiphe pisi*	Leotio	Asco	3	1	0	5	9	GCA_000208805.1	Max-Planck-Institute for Plant Breeding Research

1828	*Malassezia caprae*	Malassezio	Basidio	3	0	0	6	9	GCA_001264625.1	Genome Institute of Singapore
1829	*Puccinia striiformis*	Puccinio	Basidio	4	1	1	3	9	GCA_011750755.1	Australian National University
1830	*Wallemia mellicola*	Wallemio	Basidio	4	0	0	5	9	GCA_004919005.1	Biotechnical Faculty, University of Ljubljana
1831	*Mixia osmundae*	Mixio	Basidio	2	0	0	6	8	GCA_000708205.1	DOE Joint Genome Institute
1832	*Spizellomyces sp. 'palustris'*	Chytridio	Chytridio	4	0	0	4	8	GCA_006535965.1	Agriculture and Agri-Food Canada
1833	*Coemansia reversa*	Kickxello	Zoopago	1	2	0	5	8	GCA_002705745.1	JGI
1834	*Zoopage sp. CT-All*	Zoopago	Zoopago	1	0	3	4	8	GCA_004114245.1	University of Michigan
1835	*Sporidiobolus pararoseus*	Microbotryo	Basidio	2	0	1	5	8	GCA_010758995.1	Shenyang Agricultral University
1836	*Puccinia novopanici*	Puccinio	Basidio	3	0	0	5	8	GCA_004348175.1	Noble Research Institute
1837	*Amanita bisporigera*	Agarico	Basidio	2	0	0	6	8	GCA_001983365.1	Michigan State University
1838	*Malassezia japonica*	Malassezio	Basidio	2	0	0	6	8	GCA_001600795.1	RIKEN Center for Life Science Technologies, Division of Genomic Technologies
1839	*Malassezia equina*	Malassezio	Basidio	2	0	0	6	8	GCA_001264685.1	Genome Institute of Singapore
1840	*Malassezia sympodialis*	Malassezio	Basidio	2	0	0	6	8	GCA_001264715.1	Genome Institute of Singapore
1841	*Malassezia dermatis*	Malassezio	Basidio	2	0	0	6	8	GCA_001600775.1	RIKEN Center for Life Science Technologies, Division of Genomic Technologies
1842	*Malassezia restricta*	Malassezio	Basidio	2	0	0	6	8	GCA_001264725.1	Genome Institute of Singapore
1843	*Phaeotremella fagi*	Tremello	Basidio	1	0	3	4	8	GCA_001599715.1	RIKEN Center for Life Science Technologies, Division of Genomic Technologies
1844	*Spizellomyces punctatus*	Chytridio	Chytridio	3	0	0	4	7	GCA_000182565.2	Broad Institute
1845	*Puccinia coronata*	Puccinio	Basidio	3	0	0	4	7	GCA_002873275.1	University of Minnesota
1846	*Puccinia sorghi*	Puccinio	Basidio	3	0	0	4	7	GCA_001263375.1	BIA - FCEN - UBA
1847	*Malassezia slooffiae*	Malassezio	Basidio	2	0	0	5	7	GCA_010577765.1	Jawaharlal Nehru Centre for Advanced Scientific Research
1848	*Amanita jacksonii*	Agarico	Basidio	2	0	0	5	7	GCA_000497225.1	University of Toronto
1849	*Puccinia horiana*	Puccinio	Basidio	3	0	0	4	7	GCA_001624995.1	National Academy of Agricultural Science

1850	*Cryptococcus depauperatus*	Tremello	Basidio	2	0	1	4	7	GCA_001720245.1	Broad Institute
1851	*Malassezia nana*	Malassezio	Basidio	2	0	0	5	7	GCA_001600835.1	RIKEN Center for Life Science Technologies, Division of Genomic Technologies
1852	*Malassezia vespertilionis*	Malassezio	Basidio	1	0	0	6	7	GCA_002818225.1	US Forest Service
1853	*Malassezia pachydermatis*	Malassezio	Basidio	2	0	0	5	7	GCA_001264975.1	Genome Institute of Singapore
1854	*Malassezia sp.*	Malassezio	Basidio	2	0	0	5	7	GCA_004026415.1	Jill Banfield's Lab at Berkeley
1855	*Malassezia yamatoensis*	Malassezio	Basidio	1	0	0	6	7	GCA_001264885.1	Genome Institute of Singapore
1856	*Homolaphlyctis polyrhiza*	Chytridio	Chytridio	1	0	0	5	6	GCA_000235945.1	University of Idaho, Initiative for Bioinformatics and Evolutionary Studies
1857	*Syncephalis pseudoplumigaleata*	Zoopago	Zoopago	2	1	0	3	6	GCA_003614755.1	DOE Joint Genome Institute
1858	*Smittium mucronatum*	Harpello	Zoopago	0	2	1	3	6	GCA_001953115.1	University of Toronto
1859	*Catenaria anguillulae*	Blastocladio	Blastocladio	1	1	1	3	6	GCA_002102555.1	DOE Joint Genome Institute
1860	*Massospora platypediae*	Entomophthoro	Zoopago	3	0	0	3	6	GCA_006912095.1	University of California, Riverside
1861	*Puccinia graminis*	Puccinio	Basidio	2	0	0	4	6	GCA_008522505.1	University of Minnesota
1862	*Phaffia rhodozyma*	Tremello	Basidio	1	1	0	4	6	GCA_001600435.1	RIKEN Center for Life Science Technologies, Division of Genomic Technologies
1863	*uncultured Malassezia*	Malassezio	Basidio	2	0	0	4	6	GCA_903798065.1	EBI
1864	*Puccinia hordei*	Puccinio	Basidio	3	0	0	3	6	GCA_007896445.1	University of Sydney
1865	*Cochlonema odontosperma*	Zoopago	Zoopago	1	0	0	4	5	GCA_004114315.1	University of Michigan
1866	*Zancudomyces culisetae*	Harpello	Zoopago	1	1	0	3	5	GCA_001969505.1	University of Toronto
1867	*Acaulopage tetraceros*	Zoopago	Zoopago	1	0	0	4	5	GCA_004114255.1	University of Michigan
1868	*Thamnocephalis sphaerospora*	Zoopago	Zoopago	0	0	1	3	4	GCA_003614735.1	DOE Joint Genome Institute
1869	*Dimargaris cristalligena*	Dimargarito	Zoopago	1	1	0	2	4	GCA_003614675.1	DOE Joint Genome Institute
1870	*Batrachochytrium dendrobatidis*	Chytridio	Chytridio	1	0	0	3	4	GCA_003595275.1	University of Otago
1871	*Smittium culicis*	Harpello	Zoopago	1	0	0	3	4	GCA_001970855.1	University of Toronto
1872	*Stylopage hadra*	Zoopago	Zoopago	0	1	0	3	4	GCA_005111325.1	University of Michigan
1873	*Smittium megazygosporum*	Harpello	Zoopago	0	0	0	3	3	GCA_003086715.1	University of Toronto
1874	*Schizosaccharomyces japonicus*	Schizosaccharo	Asco	0	1	0	2	3	GCA_000149845.2	Broad Institute
1875	*Smittium simulii*	Harpello	Zoopago	0	0	0	3	3	GCA_003086735.1	University of Toronto
1876	*Schizosaccharomyces octosporus*	Schizosaccharo	Asco	0	1	0	2	3	GCA_000150505.2	Broad Institute

1877	*Batrachochytrium salamandrivorans*	Chytridio	Chytridio	0	0	0	3	3	GCA_002006685.1	Broad Institute
1878	*Piptocephalis cylindrospora*	Zoopago	Zoopago	1	0	0	2	3	GCA_003614145.1	DOE Joint Genome Institute
1879	*Schizosaccharomyces cryophilus*	Schizosaccharo	Asco	0	1	0	2	3	GCA_000004155.2	Broad Institute
1880	*Cronartium ribicola*	Puccinio	Basidio	1	0	0	2	3	GCA_000500245.1	Tree Aggressors Identification using Genomic Approaches
1881	*Herpomyces periplanetae*	Laboulbenio	Asco	2	0	0	1	3	GCA_009733715.1	University of Warsaw
1882	*Tremella mesenterica*	Tremello	Basidio	0	0	0	3	3	GCA_004117975.1	Broad Institute
1883	*Smittium angustum*	Harpello	Zoopago	0	0	0	2	2	GCA_003097675.1	University of Toronto
1884	*Furculomyces boomerangus*	Harpello	Zoopago	0	0	0	2	2	GCA_003086725.1	University of Toronto
1885	*Capniomyces stellatus*	Harpello	Zoopago	0	0	0	2	2	GCA_001661515.1	University of Toronto
1886	*Schizosaccharomyces pombe*	Schizosaccharo	Asco	0	0	0	2	2	GCA_003086255.1	Uppsala University
1887	*Rozella allomycis*	0	Crypto	0	0	0	2	2	GCA_000442015.1	University of Michigan
1888	*Melampsora medusae*	Puccinio	Basidio	1	1	0	0	2	GCA_002157035.1	BC Cancer Agency, Canada's Michael Smith Genome Sciences Centre
1889	*Nosema bombycis*	0	Microsporidia	1	0	1	0	2	GCA_000383075.1	The Institute of Sericulture and Systems Biology (ISSB) of Southwest University (SWU)
1890	*Amphiamblys sp. WSBS2006*	0	Microsporidia	1	0	0	1	2	GCA_001875675.1	A.N. Belozersky Institute of Physico-Chemical Biology, Lomonosov Moscow State University
1891	*Uromyces viciae-fabae*	Puccinio	Basidio	2	0	0	0	2	GCA_000785685.1	Universitaet Hohenheim
1892	*Melampsora occidentalis*	Puccinio	Basidio	1	0	0	0	1	GCA_002157085.1	BC Cancer Agency, Canada's Michael Smith Genome Sciences Centre
1893	*Melampsora abietis-canadensis*	Puccinio	Basidio	1	0	0	0	1	GCA_002157025.1	BC Cancer Agency, Canada's Michael Smith Genome Sciences Centre
1894	*Melampsora larici-populina*	Puccinio	Basidio	1	0	0	0	1	GCA_000204055.1	US DOE Joint Genome Institute (JGI-PGF)
1895	*Cronartium comandrae*	Puccinio	Basidio	0	0	0	1	1	GCA_000464975.1	Tree Aggressors Identification using Genomic Approaches
1896	*Meira miltonrushii*	Exobasidio	Basidio	0	0	0	1	1	GCA_003144205.1	DOE Joint Genome Institute
1897	*Endocronartium harknessii*	Puccinio	Basidio	0	1	0	0	1	GCA_000500795.1	Tree Aggressors Identification using Genomic Approaches

1898	*Mitosporidium daphniae*	0	Microsporidia	0	0	0	1	1	GCA_000760515.2	Illinois Institute of Technology
1899	*Melampsora allii-populina*	Puccinio	Basidio	0	0	0	1	1	GCA_002157005.1	BC Cancer Agency, Canada's Michael Smith Genome Sciences Centre
1900	*Microbotryum lychnidis-dioicae*	Microbotryo	Basidio	0	1	0	0	1	GCA_001244265.1	INRA-LIPM
1901	*Pseudoloma neurophilia*	0	Microsporidia	1	0	0	0	1	GCA_001432165.1	University of Ottawa
1902	*Melampsora aecidioides*	Puccinio	Basidio	0	0	0	1	1	GCA_002157015.1	BC Cancer Agency, Canada's Michael Smith Genome Sciences Centre
1903	*Paramicrosporidium saccamoebae*	0	Crypto	0	0	0	0	0	GCA_002794465.1	University of Michigan
1904	*Nosema ceranae*	0	Microsporidia	0	0	0	0	0	GCA_004919615.1	Jiangxi Agricultural University
1905	*Vavraia culicis*	0	Microsporidia	0	0	0	0	0	GCA_000192795.1	Broad Institute
1906	*Tubulinosema ratisbonensis*	0	Microsporidia	0	0	0	0	0	GCA_004000155.1	Universite Clermont Auvergne, CNRS UMR 6023
1907	*Edhazardia aedis*	0	Microsporidia	0	0	0	0	0	GCA_000230595.3	Broad Institute
1908	*Nematocida displodere*	0	Microsporidia	0	0	0	0	0	GCA_001642395.1	University of California San Diego
1909	*Hamiltosporidium tvaerminnensis*	0	Microsporidia	0	0	0	0	0	GCA_004325075.1	Illinois Institute of Technology
1910	*Austropuccinia psidii*	Puccinio	Basidio	0	0	0	0	0	GCA_902702905.1	UNIVERSITY OF SYDNEY
1911	*Trachipleistophora hominis*	0	Microsporidia	0	0	0	0	0	GCA_000316135.1	Institute for Cell and Molecular Biosciences, Newcastle University
1912	*Hamiltosporidium magnivora*	0	Microsporidia	0	0	0	0	0	GCA_004325065.1	Illinois Institute of Technology
1913	*Encephalitozoon intestinalis*	0	Microsporidia	0	0	0	0	0	GCA_000146465.1	Keeling lab, University of British Columbia
1914	*Nematocida parisii*	0	Microsporidia	0	0	0	0	0	GCA_000190615.1	Broad Institute
1915	*Enterocytozoon bieneusi*	0	Microsporidia	0	0	0	0	0	GCA_000209485.1	Tufts Cummings School of Veterinary Medicine
1916	*Vittaforma corneae*	0	Microsporidia	0	0	0	0	0	GCA_000231115.1	Broad Institute
1917	*Encephalitozoon hellem*	0	Microsporidia	0	0	0	0	0	GCA_000277815.3	University of British Columbia
1918	*Encephalitozoon romaleae*	0	Microsporidia	0	0	0	0	0	GCA_000280035.2	University of Ottawa
1919	*Nematocida sp. 1*	0	Microsporidia	0	0	0	0	0	GCA_000738915.1	Broad Institute
1920	*Ordospora colligata*	0	Microsporidia	0	0	0	0	0	GCA_000803265.1	University of British Columbia
1921	*Encephalitozoon cuniculi*	0	Microsporidia	0	0	0	0	0	GCA_001078035.1	University of Ottawa
1922	*Nematocida sp. ERTm5*	0	Microsporidia	0	0	0	0	0	GCA_001642415.1	University of California San Diego

1923	*Hemileia vastatrix*	Puccinio	Basidio	0	0	0	0	0	GCA_004125335.1	Universidade Federal de Lavras (UFLA)
1924	*Antonospora locustae*	0	Microsporidia	0	0	0	0	0	GCA_007674295.1	Zhengzhou Normal University
1925	*Cronartium quercuum*	Puccinio	Basidio	0	0	0	0	0	GCA_000500775.1	Tree Aggressors Identification using Genomic Approaches
1926	*Pneumocystis jirovecii*	Pneumocystido	Asco	0	0	0	0	0	GCA_001477535.1	Broad Institute
1927	*Hepatospora eriocheir*	0	Microsporidia	0	0	0	0	0	GCA_002087885.1	University of Exeter
1928	*Metchnikovella incurvata*	0	Microsporidia	0	0	0	0	0	GCA_003600395.1	SINGEK
1929	*Enterocytozoon hepatopenaei*	0	Microsporidia	0	0	0	0	0	GCA_003709115.1	Korea Research Institute of Bioscience & Biotechnology
1930	*Pneumocystis carinii*	Pneumocystido	Asco	0	0	0	0	0	GCA_001477545.1	Broad Institute
1931	*Meira nashicola*	Exobasidio	Basidio	0	0	0	0	0	GCA_001600355.1	RIKEN Center for Life Science Technologies, Division of Genomic Technologies
1932	*Enterospora canceri*	0	Microsporidia	0	0	0	0	0	GCA_002087915.1	University of Exeter

Table S3. Categorization of “Family;Function” data (EC number in relation to substrate associations. The Table displays the data in two ways: Two left columns lists the order according to ascending EC number; The four columns to the right lists the “Family;Function” organized per substrate. The term GENERAL indicates what the family generally is associated to a certain substrate, in case the identified group does not have a known molecular function.

Function;family	Substrate	Function:family substrate annotations encoded CAZymes			
		Cellulose	Pectin	Xylan	Lignin
GENERAL;AA1	Lignin	GENERAL;AA3	3.1.1.11;CE0	GENERAL;AA14	1.10.3.*;AA1
1.10.3.2;AA1	Lignin	1.1.99.18;AA3	GENERAL;CE8	1.*.*.*;AA14	GENERAL;AA2
1.10.3.*;AA1	Lignin	1.1.3.4;AA3	3.1.1.11;CE8	3.1.1.72;CE1	1.11.1.13;AA2
GENERAL;AA2	Lignin	1.1.3.7;AA3	3.1.1.*;CE12	GENERAL;CE2	1.11.1.16;AA2
1.11.1.13;AA2	Lignin	1.1.3.13;AA3	GENERAL;CE13	3.1.1.72;CE2	1.11.1.14;AA2
1.11.1.16;AA2	Lignin	1.1.3.10;AA3	3.1.1.*;CE13	GENERAL;CE3	1.11.1.*;AA2
1.11.1.14;AA2	Lignin	GENERAL;AA9	3.2.1.23;GH0	3.1.1.72;CE3	1.11.1.5;AA2
1.11.1.*;AA2	Lignin	1.14.99.54;AA9	3.2.1.23;GH1	3.1.1.72;CE4	GENERAL;AA3
1.11.1.5;AA2	Lignin	1.14.99.56;AA9	3.2.1.23;GH2	3.1.1.72;CE5	1.1.99.18;AA3
GENERAL;AA3	Cellulose;Lignin	GENERAL;AA10	3.2.1.31;GH2	GENERAL;CE6	1.1.3.4;AA3
1.1.99.18;AA3	Cellulose;Lignin	1.14.99.54;AA10	3.2.1.55;GH2	3.1.1.72;CE6	1.1.3.7;AA3
1.1.3.4;AA3	Cellulose;Lignin	1.14.99.56;AA10	3.2.1.*;GH2	GENERAL;CE7	1.1.3.13;AA3
1.1.3.7;AA3	Cellulose;Lignin	1.14.99.53;AA10	3.2.1.*;GH2	3.1.1.72;CE7	1.1.3.10;AA3
1.1.3.13;AA3	Cellulose;Lignin	GENERAL;AA15	3.2.1.55;GH3	3.1.1.72;CE12	GENERAL;AA4
1.1.3.10;AA3	Cellulose;Lignin	1.14.99.54;AA15	3.2.1.164;GH5	GENERAL;CE15	1.1.3.38;AA4
GENERAL;AA4	Lignin	GENERAL;AA16	3.2.1.181;GH16	3.1.1.*;CE15	GENERAL;AA5
1.1.3.38;AA4	Lignin	1.14.99.54;AA16	3.2.1.22;GH27	GENERAL;CE16	1.1.3.*;AA5
GENERAL;AA5	Lignin	3.2.1.4;GH0	GENERAL;GH28	3.1.1.6;CE16	1.1.3.9;AA5
1.1.3.*;AA5	Lignin	3.2.1.21;GH0	3.2.1.15;GH28	3.2.1.37;GH1	1.2.3.15;AA5
1.1.3.9;AA5	Lignin	3.2.1.21;GH1	3.2.1.40;GH28	3.2.1.31;GH2	1.1.3.13;AA5
1.2.3.15;AA5	Lignin	3.2.1.*;GH1	3.2.1.67;GH28	3.2.1.55;GH2	GENERAL;AA6
1.1.3.13;AA5	Lignin	3.2.1.21;GH3	3.2.1.82;GH28	3.2.1.37;GH2	1.6.5.6;AA6
GENERAL;AA6	Lignin	3.2.1.74;GH3	3.2.1.171;GH28	3.2.1.37;GH3	GENERAL;AA8
1.6.5.6;AA6	Lignin	3.2.1.4;GH5	3.2.1.173;GH28	3.2.1.55;GH3	GENERAL;AA9
GENERAL;AA8	Lignin	3.2.1.21;GH5	3.2.1.*;GH28	3.2.1.8;GH5	1.14.99.54;AA9
GENERAL;AA9	Cellulose;Lignin	3.2.1.74;GH5	3.2.1.164;GH30	3.2.1.8;GH8	1.14.99.56;AA9

1.14.99.54;AA9	Cellulose;Lignin	3.2.1.91;GH5	3.2.1.*;GH33	3.2.1.156;GH8
1.14.99.56;AA9	Cellulose;Lignin	GENERAL;GH6	3.2.1.23;GH35	3.2.1.8;GH10
GENERAL;AA10	Cellulose	3.2.1.4;GH6	3.2.1.*;GH35	3.2.1.32;GH10
1.14.99.54;AA10	Cellulose	3.2.1.91;GH6	3.2.1.23;GH42	2.4.2.*;GH10
1.14.99.56;AA10	Cellulose	3.2.1.4;GH7	3.2.1.*;GH42	GENERAL;GH11
1.14.99.53;AA10	Cellulose	3.2.1.176;GH7	3.2.1.55;GH43	3.2.1.8;GH11
GENERAL;AA14	Xylan	3.2.1.4;GH8	3.2.1.*;GH43	3.2.1.32;GH11
1.*.*.*;AA14	Xylan	3.2.1.4;GH9	3.2.1.99;GH43	3.2.1.32;GH26
GENERAL;AA15	Cellulose	3.2.1.21;GH9	3.2.1.145;GH43	3.2.1.8;GH30
1.14.99.54;AA15	Cellulose	3.2.1.73;GH9	3.2.1.55;GH51	3.2.1.37;GH30
GENERAL;AA16	Cellulose	3.2.1.74;GH9	GENERAL;GH53	3.2.1.136;GH30
1.14.99.54;AA16	Cellulose	3.2.1.91;GH9	3.2.1.89;GH53	3.2.1.*;GH30
3.1.1.11;CE0	Pectin	3.2.1.4;GH10	3.2.1.55;GH54	3.2.1.177;GH31
3.1.1.72;CE1	Xylan	3.2.1.4;GH12	3.2.1.23;GH59	3.2.1.37;GH39
GENERAL;CE2	Xylan	3.2.1.21;GH30	3.2.1.55;GH62	3.2.1.37;GH43
3.1.1.72;CE2	Xylan	3.2.1.4;GH44	GENERAL;GH78	3.2.1.55;GH43
GENERAL;CE3	Xylan	3.2.1.4;GH45	3.2.1.40;GH78	3.2.1.8;GH43
3.1.1.72;CE3	Xylan	3.2.1.176;GH48	3.2.1.174;GH78	3.2.1.*;GH43
3.1.1.72;CE4	Xylan	3.2.1.4;GH48	3.2.1.*;GH78	GENERAL;GH51
3.1.1.72;CE5	Xylan	3.2.1.4;GH51	GENERAL;GH93	3.2.1.8;GH51
GENERAL;CE6	Xylan	3.2.1.4;GH74	3.2.1.*;GH93	3.2.1.37;GH51
3.1.1.72;CE6	Xylan	3.2.1.21;GH116	3.2.1.*;GH95	3.2.1.55;GH51
GENERAL;CE7	Xylan	GENERAL;GH124	3.2.1.172;GH105	GENERAL;GH52
3.1.1.72;CE7	Xylan	3.2.1.4;GH124	3.2.1.*;GH105	3.2.1.37;GH52
GENERAL;CE8	Pectin		GENERAL;GH106	GENERAL;GH54
3.1.1.11;CE8	Pectin		3.2.1.40;GH106	3.2.1.55;GH54
3.1.1.*;CE12	Pectin		3.2.1.174;GH106	3.2.1.37;GH54
3.1.1.72;CE12	Xylan		3.2.1.185;GH127	GENERAL;GH62
GENERAL;CE13	Pectin		3.2.1.*;GH127	3.2.1.55;GH62
3.1.1.*;CE13	Pectin		3.2.1.185;GH137	GENERAL;GH67
GENERAL;CE15	Xylan		3.2.1.185;GH137	3.2.1.139;GH67

3.1.1.*;CE15	Xylan	GENERAL;GH138	3.2.1.131;GH67
GENERAL;CE16	Xylan	3.2.1.173;GH138	3.2.1.8;GH98
3.1.1.6;CE16	Xylan	GENERAL;GH139	GENERAL;GH115
3.2.1.23;GH0	Pectin	3.2.1.*;GH139	3.2.1.131;GH115
3.2.1.4;GH0	Cellulose	GENERAL;GH140	3.2.1.*;GH115
3.2.1.21;GH0	Cellulose	3.2.1.*;GH140	3.2.1.37;GH116
3.2.1.21;GH1	Cellulose	3.2.1.51;GH141	GENERAL;GH120
3.2.1.23;GH1	Pectin	GENERAL;GH142	3.2.1.37;GH120
3.2.1.37;GH1	Xylan	3.2.1.185;GH142	3.2.1.8;GH141
3.2.1.*;GH1	Cellulose	GENERAL;GH143	
3.2.1.23;GH2	Pectin	3.2.1.*;GH143	
3.2.1.31;GH2	Xylan	GENERAL;GH145	
3.2.1.31;GH2	Pectin	3.2.1.*;GH145	
3.2.1.55;GH2	Xylan	GENERAL;GH146	
3.2.1.55;GH2	Pectin	3.2.1.185;GH146	
3.2.1.*;GH2	Pectin	3.2.1.185;GH146	
3.2.1.*;GH2	Pectin	GENERAL;GH147	
3.2.1.37;GH2	Xylan	3.2.1.23;GH147	
3.2.1.21;GH3	Cellulose	GENERAL;GH160	
3.2.1.37;GH3	Xylan	3.2.1.*;GH160	
3.2.1.55;GH3	Xylan	GENERAL;GH165	
3.2.1.55;GH3	Pectin	3.2.1.23;GH165	
3.2.1.74;GH3	Cellulose	GENERAL;PL1	
3.2.1.4;GH5	Cellulose	4.2.2.2;PL1	
3.2.1.8;GH5	Xylan	4.2.2.9;PL1	
3.2.1.21;GH5	Cellulose	4.2.2.10;PL1	
3.2.1.74;GH5	Cellulose	GENERAL;PL2	
3.2.1.91;GH5	Cellulose	4.2.2.2;PL2	
3.2.1.164;GH5	Pectin	4.2.2.9;PL2	
GENERAL;GH6	Cellulose	GENERAL;PL3	
3.2.1.4;GH6	Cellulose	4.2.2.2;PL3	

3.2.1.91;GH6	Cellulose	GENERAL;PL4
3.2.1.4;GH7	Cellulose	4.2.2.23;PL4
3.2.1.176;GH7	Cellulose	GENERAL;PL9
3.2.1.4;GH8	Cellulose	4.2.2.2;PL9
3.2.1.8;GH8	Xylan	4.2.2.9;PL9
3.2.1.156;GH8	Xylan	4.2.2.23;PL9
3.2.1.4;GH9	Cellulose	GENERAL;PL10
3.2.1.21;GH9	Cellulose	4.2.2.2;PL10
3.2.1.73;GH9	Cellulose	GENERAL;PL11
3.2.1.74;GH9	Cellulose	4.2.2.23;PL11
3.2.1.91;GH9	Cellulose	4.2.2.24;PL11
3.2.1.8;GH10	Xylan	GENERAL;PL22
3.2.1.32;GH10	Xylan	4.2.2.6;PL22
2.4.2.*;GH10	Xylan	GENERAL;PL26
3.2.1.4;GH10	Cellulose	4.2.2.24;PL26
GENERAL;GH11	Xylan	GENERAL;PL27
3.2.1.8;GH11	Xylan	4.2.2.*;PL27
3.2.1.32;GH11	Xylan	
3.2.1.4;GH12	Cellulose	
3.2.1.181;GH16	Pectin	
3.2.1.32;GH26	Xylan	
3.2.1.22;GH27	Pectin	
GENERAL;GH28	Pectin	
3.2.1.15;GH28	Pectin	
3.2.1.40;GH28	Pectin	
3.2.1.67;GH28	Pectin	
3.2.1.82;GH28	Pectin	
3.2.1.171;GH28	Pectin	
3.2.1.173;GH28	Pectin	
3.2.1.*;GH28	Pectin	
3.2.1.8;GH30	Xylan	

3.2.1.21;GH30	Cellulose
3.2.1.37;GH30	Xylan
3.2.1.136;GH30	Xylan
3.2.1.164;GH30	Pectin
3.2.1.*;GH30	Xylan
3.2.1.177;GH31	Xylan
3.2.1.*;GH33	Pectin
3.2.1.23;GH35	Pectin
3.2.1.*;GH35	Pectin
3.2.1.37;GH39	Xylan
3.2.1.23;GH42	Pectin
3.2.1.*;GH42	Pectin
3.2.1.37;GH43	Xylan
3.2.1.55;GH43	Pectin
3.2.1.55;GH43	Xylan
3.2.1.8;GH43	Xylan
3.2.1.*;GH43	Xylan
3.2.1.*;GH43	Pectin
3.2.1.99;GH43	Pectin
3.2.1.145;GH43	Pectin
3.2.1.4;GH44	Cellulose
3.2.1.4;GH45	Cellulose
3.2.1.176;GH48	Cellulose
3.2.1.4;GH48	Cellulose
GENERAL;GH51	Xylan
3.2.1.4;GH51	Cellulose
3.2.1.8;GH51	Xylan
3.2.1.37;GH51	Xylan
3.2.1.55;GH51	Xylan
3.2.1.55;GH51	Pectin
GENERAL;GH52	Xylan

3.2.1.37;GH52	Xylan
GENERAL;GH53	Pectin
3.2.1.89;GH53	Pectin
GENERAL;GH54	Xylan
3.2.1.55;GH54	Xylan
3.2.1.55;GH54	Pectin
3.2.1.37;GH54	Xylan
3.2.1.23;GH59	Pectin
GENERAL;GH62	Xylan
3.2.1.55;GH62	Xylan
3.2.1.55;GH62	Pectin
GENERAL;GH67	Xylan
3.2.1.139;GH67	Xylan
3.2.1.131;GH67	Xylan
3.2.1.4;GH74	Cellulose
GENERAL;GH78	Pectin
3.2.1.40;GH78	Pectin
3.2.1.174;GH78	Pectin
3.2.1.*;GH78	Pectin
GENERAL;GH93	Pectin
3.2.1.*;GH93	Pectin
3.2.1.*;GH95	Pectin
3.2.1.8;GH98	Xylan
3.2.1.172;GH105	Pectin
3.2.1.*;GH105	Pectin
GENERAL;GH106	Pectin
3.2.1.40;GH106	Pectin
3.2.1.174;GH106	Pectin
GENERAL;GH115	Xylan
3.2.1.131;GH115	Xylan
3.2.1.*;GH115	Xylan

3.2.1.21;GH116	Cellulose
3.2.1.37;GH116	Xylan
GENERAL;GH120	Xylan
3.2.1.37;GH120	Xylan
GENERAL;GH124	Cellulose
3.2.1.4;GH124	Cellulose
3.2.1.185;GH127	Pectin
3.2.1.*;GH127	Pectin
3.2.1.185;GH137	Pectin
3.2.1.185;GH137	Pectin
GENERAL;GH138	Pectin
3.2.1.173;GH138	Pectin
GENERAL;GH139	Pectin
3.2.1.*;GH139	Pectin
GENERAL;GH140	Pectin
3.2.1.*;GH140	Pectin
3.2.1.51;GH141	Pectin
3.2.1.8;GH141	Xylan
GENERAL;GH142	Pectin
3.2.1.185;GH142	Pectin
GENERAL;GH143	Pectin
3.2.1.*;GH143	Pectin
GENERAL;GH145	Pectin
3.2.1.*;GH145	Pectin
GENERAL;GH146	Pectin
3.2.1.185;GH146	Pectin
3.2.1.185;GH146	Pectin
GENERAL;GH147	Pectin
3.2.1.23;GH147	Pectin
GENERAL;GH160	Pectin
3.2.1.*;GH160	Pectin

GENERAL;GH165	Pectin
3.2.1.23;GH165	Pectin
GENERAL;PL1	Pectin
4.2.2.2;PL1	Pectin
4.2.2.9;PL1	Pectin
4.2.2.10;PL1	Pectin
GENERAL;PL2	Pectin
4.2.2.2;PL2	Pectin
4.2.2.9;PL2	Pectin
GENERAL;PL3	Pectin
4.2.2.2;PL3	Pectin
GENERAL;PL4	Pectin
4.2.2.23;PL4	Pectin
GENERAL;PL9	Pectin
4.2.2.2;PL9	Pectin
4.2.2.9;PL9	Pectin
4.2.2.23;PL9	Pectin
GENERAL;PL10	Pectin
4.2.2.2;PL10	Pectin
GENERAL;PL11	Pectin
4.2.2.23;PL11	Pectin
4.2.2.24;PL11	Pectin
GENERAL;PL22	Pectin
4.2.2.6;PL22	Pectin
GENERAL;PL26	Pectin
4.2.2.24;PL26	Pectin
GENERAL;PL27	Pectin
4.2.2.*;PL27	Pectin
